# Unlock lasing in two-dimensional metal halide perovskites by tuning ns^2^ lone pairs

**DOI:** 10.1126/sciadv.aee5631

**Published:** 2026-08-05

**Authors:** Xinyu Li, Mingyuan Li, Shixuan Zheng, Rui Wen, Meng Zhang, Songhao Guo, Yu Tao, Yuhong Mao, Xiaofan Jiang, Jiazhen Gu, Tianhao Zhang, Yan Guan, Chen Li, Xujie Lü, Yongping Fu

**Affiliations:** ^1^ Beijing National Laboratory for Molecular Science, State Key Laboratory of Rare Earth Materials Chemistry and Applications, College of Chemistry and Molecular Engineering, Peking University, Beijing 100871, China.; ^2^ Center for High Pressure Science and Technology Advanced Research, Shanghai 201203, China.

## Abstract

Despite the proliferation of lasing reports in three-dimensional (3D) lead halide perovskites, robust lasing in their single-layer two-dimensional (2D) counterparts, (LA)_2_PbX_4_ (LA = spacer cation; X = halide), has remained elusive. Here, we uncover the critical role of metal ns^2^-lone-pair stereochemistry in modulating excitonic properties and enabling lasing in these materials. Using a library of (LA)_2_BI_4_ [B = Pb (lead), Sn (tin), or Ge (germanium)], we identify key structural descriptors that link lone pair activity to exciton-phonon coupling and exciton-exciton annihilation. Within a given B-cation series, rigid frameworks with shorter B─I bonds suppress lone pair activity and favor free exciton emission, while enhanced lone pair activity—tuned by the spacer and B-cation—can localize excitons, increase dielectric screening, and suppress exciton-exciton annihilation at high excitation fluences. By balancing these effects through cation selection, we demonstrate lasing in a newly synthesized Pb-based (2FBMZ)_2_PbI_4_ (2FBMZ = 5,6-difluoro-1*H*-benzimidazole cation) and achieve the most thermally stable lasing in its Sn-based analog. These insights provide design principles for high-brightness 2D perovskite photonic devices.

## Teaser

Tuning ns^2^ lone pairs suppresses exciton annihilation and establishes a design principle for lasing in 2D perovskites.

## INTRODUCTION

The ns^2^ electron–derived lone pairs in heavy main group cations are known to drive a wide range of material properties, from spontaneous lattice polarization in ferroelectrics to second-harmonic generation in nonlinear optical materials (*1*–*3*). Recently, their role in developing favorable optoelectronic properties has drawn increasing attention, spurred by the success of lead halide perovskites in photovoltaics (*4*). In these materials, the stereochemical expression of the 6s^2^ lone pair is suggested to contribute to defect tolerance by generating a large static dielectric constant that effectively screens the Coulomb interaction between carriers and charged defects or impurities (*5*–*7*). These insights have sparked growing interest in a broader class of lone pair–bearing semiconductors (*7*–*9*). Comparative studies across several series of lead-based perovskites show that enhanced lone pair activity leads to stronger exciton-phonon coupling and substantially affects their optical properties (*10*–*12*). Yet, a comprehensive understanding of how ns^2^ lone pair stereochemistry governs these optical properties has yet to be established (*10*, *11*, *13*–*15*).

Two-dimensional (2D) halide perovskites, with the general formula (LA)_2_BX_4_ [LA = organic spacer cation; B = Pb (lead), Sn (tin), or Ge (germanium)], offer a versatile platform to probe the effects of lone pair activity on the optical properties (*5*, *11*, *16*). Their intrinsic quantum and reduced dielectric confinement results in tightly bound excitons due to enhanced Coulomb interactions, making them attractive for light-emitting applications (*17*, *18*). Organic spacer cation engineering has been shown to suppress exciton-phonon coupling, enabling high photoluminescence (PL) quantum yields (*11*, *19*, *20*). Despite these advances, lasing in Pb-based (LA)_2_PbX_4_ remains unexpectedly rare, especially given the extensive demonstrations of 3D lead halide perovskites over the past decade (*21*–*25*). An early study reported lasing from (C_6_H_13_NH_3_)_2_PbI_4_ at 16 K (*26*), but a recent photophysical study suggests that the observed narrow emission peaks may originate from cavity or waveguiding effects rather than stimulated emission (*27*). Similarly, while some studies have claimed lasing in the prototypical (BA)_2_PbI_4_ (BA = butylammonium) (*28*) or (PEA)_2_PbI_4_ (PEA = phenylethylammonium) (*29*), others reported its absence, attributing the lack of lasing to strong exciton-phonon coupling or competing Auger recombination (*20*, *27*, *30*, *31*). Robust lasing has been demonstrated in Sn-based (LA)_2_SnI_4_, including (PEA)_2_SnI_4_, under cryogenic conditions across several research groups (*32*–*34*). It has been shown that incorporation of rigid conjugated spacers in (LA)_2_SnI_4_ enhances the lasing performance by suppressing exciton-phonon coupling (*33*).

Here, we identify the structural descriptors that control lasing in 2D halide perovskites. We uncover the critical role of ns^2^ lone pair stereochemistry in modulating exciton-phonon coupling and exciton-exciton annihilation (EEA) and thereby determining their lasing performance. EEA is a dominant nonradiative decay pathway at high exciton densities and is often considered as a fundamental bottleneck for achieving lasing in 2D semiconductors more broadly (*35*–*39*). Through a systematic study of the (LA)_2_BX_4_ library of >140 compounds (including 20 newly synthesized structures) with varied spacers and B-cations, we show that, within a given B-cation series, rigid inorganic frameworks with short B─I bonds generally suppress lone pair activity and exciton-phonon coupling, enabling efficient and bright free exciton (FE) emission. At the same time, lone pair activity, modulated by both the spacer cation and B-cation, also affects exciton localization and dielectric screening and therefore influences EEA (*40*, *41*). In particular, when comparing Pb- and Sn-based structures, the stronger lone pair effect in Sn^2+^ enhances exciton localization and dielectric screening, both of which help suppress EEA, whereas Ge-based structures typically lie in a much more strongly self-trapped regime. By balancing structural rigidity and lone pair activity via spacer cation and B-cation selection, we demonstrate lasing in a newly synthesized (2FBMZ)_2_PbI_4_ (2FBMZ = 5,6-difluoro-1*H*-benzimidazole cation), in contrast to the absence of lasing in the benchmark (PEA)_2_PbI_4_. Furthermore, we achieve robust lasing up to near room temperature in its Sn-based analog (2FBMZ)_2_SnI_4_, outperforming other (LA)_2_SnI_4_. These results uncover fundamental structure-property relationships connecting lone pair stereochemistry to excitonic dynamics, offering design principles of 2D perovskites for high-brightness optoelectronic and photonic applications.

## RESULTS

### Tuning lone pair activity by the spacer and B-cation

**Fig. 1.**
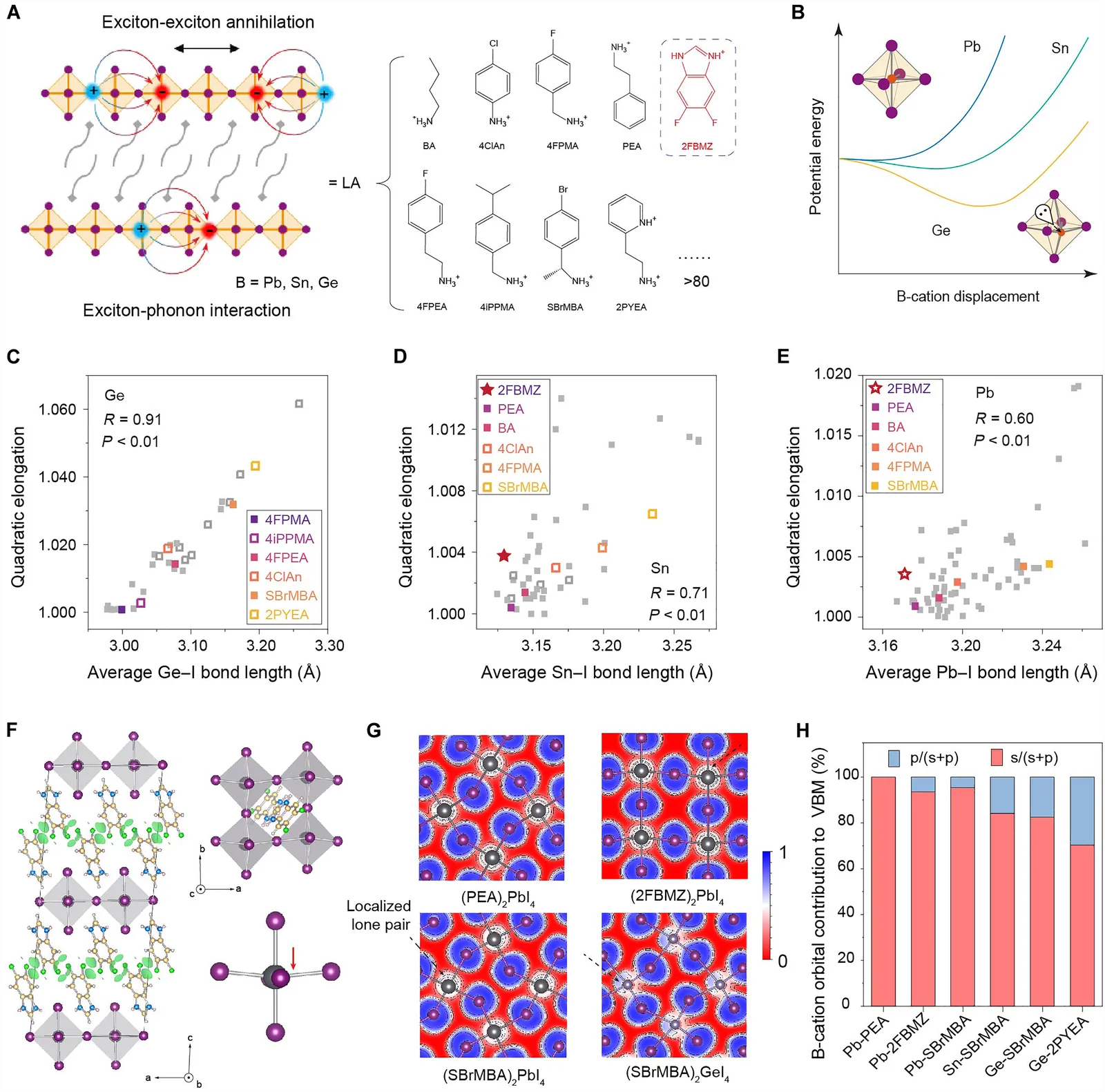
Structural distortion and lone pair activity in 2D perovskites. (**A**) Schematic crystal structure of 2D perovskites (LA)_2_BI_4_, showing the layered inorganic framework templated by organic spacer cations (representative LA cations are shown at right). Exciton-phonon coupling and EEA are also illustrated. (**B**) Schematic diagram of lone pair expression on the B-cation. As lone pair activity increases from Pb to Ge, the PES (potential energy surface) evolves from an anharmonic profile to a double-well profile, leading to octahedral dictation with off-center B-cation displacement. (**C** to **E**) Structural correlation map of quadratic elongation (〈λ〉) versus average bond length ($\bar{d}$) for Ge-based (C), Sn-based (D), and Pb-based (E) structures, revealing strong correlation between 〈λ〉 and $\bar{d}$. The numbers of crystal structures surveyed in each panel are 31, 45, and 68, respectively. Empty points (including empty squares and stars) are our newly synthesized structures. Selected spacer cations for detailed studies are highlighted; others are shown in fig. S1. (**F**) Crystal structure of (2FBMZ)_2_PbI_4_, highlighting the C─F···H hydrogen bonding, F···π halogen bonding, and F···F intermolecular interaction between spacer cations, shown as green regions representing the IGMH (independent gradient model based on Hirshfeld partition) isosurfaces at δg^inter^ = 0.005 a.u. (arbitrary units). The cavity is stretched along the **a** + **b** direction, and the octahedra are distorted to accommodate the anisotropic and oversized imidazolium headgroups in 2FBMZ. (**G**) ELF maps of (PEA)_2_PbI_4_, (2FBMZ)_2_PbI_4_, (SBrMBA)_2_PbI_4_, and (SBrMBA)_2_GeI_4_, visualizing increasing lone pair localization from nearly spherical to crescent-shaped electron density around the Pb atoms, as well as from the Pb structure to the Ge structure. The dashed arrows indicate localized lone pair density. (**H**) Orbital contribution of B-cation s and p orbitals to the VBM for representative structures, highlighting enhanced sp mixing with increasing $\bar{d}$ or 〈λ〉 within the same B-cation series or by changing the B-cation from Pb to Ge.

We start with the trends of lone pair activity in (LA)_2_BI_4_ as a function of spacers and B-cations by combining crystallographic analysis with theoretical calculations. The crystal structure of (LA)_2_BI_4_ comprises single layers of corner-sharing BI_6_^4−^ octahedra separated by bilayers of organic spacer cations (Fig. 1A). These spacer cations template the interoctahedral spacing and modulate intraoctahedral distortion (*5*, *11*, *16*, *18*). The lone pair activity on the B-cation arises from a second-order Jahn-Teller distortion, driven by sp mixing between a filled ground state with partial s-orbital character and a low-lying excited state derived from p orbitals, enabled by off-centering of the B-cation (i.e., inversion symmetry breaking of the octahedra) (*1*, *2*, *5*). This interaction leads to a localized ns^2^ electron density around the B-cation to different degrees depending on the B-cation (Pb < Sn < Ge), as illustrated in Fig. 1B. To assess its impact on the excitonic properties within a unified framework, we systematically studied a library of (LA)_2_BI_4_ with various B-cations and 94 spacer cations (fig. S1), including 124 from the crystallographic database and 20 newly synthesized structures by us (table S1). This comprehensive structural survey reveals how both the metal and organic spacer cations modulate lone pair activity, enabling us to identify the key structural features that govern the excitonic properties and eventual lasing performance.

For each B-cation series, we evaluated the lone pair activity using two structural descriptors: average B─I bond length ($\bar{d}$) and intraoctahedral distortion (*16*, *42*). The latter was characterized using two parameters: the quadratic elongation, $\langle \lambda \rangle =\sum_{i=1}^{6}{\left(d_{i}/\bar{d}\right)}^{2}/6$, and the bond angle variance, $\sigma_{\theta }^{2}=\sum_{i=1}^{12}{\left(\theta_{i}-90{}^{\circ}\right)}^{2}/11$, where $d_{i}$ is the individual B─I bond length, and $\theta_{i}$ is the I─B─I bond angle. As $\sigma_{\theta }^{2}$ strongly correlates with 〈λ〉 (fig. S2), we focus on 〈λ〉 in the main discussion. These two descriptors are chosen on the basis of the two following considerations (*16*, *42*). First, theoretical calculations have shown that the potential energy surfaces (PESs) associated with the B-cation off-center displacement evolve from a single-well shape to a double-well shape as the metal halide bond length increases. Therefore, a longer $\bar{d}$ generally indicates a reduced restoring force for B-cation displacement and a greater propensity for lone pair activity. Second, once the lone pair becomes stereochemically active, the resulting off-centering distorts the local octahedral coordination environment, increasing the octahedral distortion parameters. A summary of $\bar{d}$, 〈λ〉, and $\sigma_{\theta }^{2}$ of all the studied structures is listed in table S2. Figure 1 (C to E) shows the structural correlation maps between the 〈λ〉 and $\bar{d}$ structural parameters for the Ge-, Sn-, and Pb-based 2D perovskite series. The observed $\bar{d}$ ranges are 2.95 to 3.27 Å for Ge─I, 3.12 to 3.28 Å for Sn─I, and 3.16 to –3.27 Å for Pb─I, and these variations in each series are caused by different packing distances between the spacer cations. A strong correlation between 〈λ〉 and ${\bar{d}}_{\text{Ge}─I}$ is observed in (LA)_2_GeI_4_ (Pearson *R* = 0.91, *P* < 0.01), with slightly weaker correlation in (LA)_2_SnI_4_ (*R* = 0.71, *P* < 0.01). For (LA)_2_PbI_4_, the correlation is ambiguous at shorter Pb─I bond lengths (${\bar{d}}_{\text{Pb}─I}$ within 3.16 to 3.20 Å) but becomes statistically significant at longer bond lengths, ${\bar{d}}_{\text{Pb}─I}$ > 3.20 Å.

To understand the above structural trends, we compared the PES for B-cation displacement in model structures Cs_2_BI_4_ (fig. S3) with different B-cations (*16*). By manually varying the B─I bond length, we simulated the templating effect of different spacer cations on the bond length of the inorganic [BI_4_]^2−^ framework. As $\bar{d}$ increases, the PES evolves into an anharmonic shape and, once a certain bond length is exceeded, develops a local minimum corresponding to an off-center displacement of the B-cation; this displacement then continuously grows with a further increase in $\bar{d}$ (fig. S3B). Consequently, 〈λ〉 of the minimum-energy structures remains constant below the bond length threshold but markedly increases above it (fig. S3C). This simple model effectively captures the observed correlation between $\bar{d}$ and 〈λ〉, particularly for (LA)_2_GeI_4_ series.

However, at a short $\bar{d}$—especially in (LA)_2_PbI_4_ series—lone pair–induced distortion would be expected to be suppressed, yet notable exceptions are observed. One notable example is our newly synthesized (2FBMZ)_2_PbI_4_, which features one of the shortest $\bar{d}$ values but a large 〈λ〉 with off-center Pb^2+^ displacement (Fig. 1F). In this structure, the imidazolium headgroup in 2FBMZ is slightly oversized and anisotropic relative to the cavity formed by four [PbI_6_]^4−^ octahedra, inducing diagonal stretching and octahedral distortion of the inorganic framework, which would typically lead to bond length elongation. However, the packing of 2FBMZ features strong interlayer and intralayer intermolecular interactions (Fig. 1F and fig. S4), which compress the inorganic lattice and result in a short $\bar{d}$. These observations suggest that, beyond the packing distance, the specific geometry of the spacer cation and organic-inorganic interactions could also modulate the PES, influencing lone pair activity (e.g., 〈λ〉) among structures with similar $\bar{d}$. Furthermore, similar structural characteristics are also found in (2FBMZ)_2_SnI_4_, whereas (2FBMZ)_2_GeI_4_ behaves differently (fig. S5), suggesting that the structural response to the 2FBMZ spacer also depends on the identity of the metal cation.

The depth of the potential well follows the order Cs_2_GeI_4_ > Cs_2_SnI_4_ > Cs_2_PbI_4_, suggesting that the tendency to exhibit octahedral distortion increases from Pb to Ge, consistent with the more active ns^2^ electron pair (Fig. 1B and fig. S3B). The deepest potential well in Ge structures indicates that the PES is less perturbed by spacer cation geometry, leading to the strongest correlation between $\bar{d}$ and 〈λ〉 (Fig. 1C). In contrast, the shallower PES in Sn structures makes their octahedral distortion more sensitive to the specific organic-inorganic interactions, leading to greater variability in 〈λ〉 and a weaker correlation between $\bar{d}$ and 〈λ〉 (Fig. 1D). For Pb structures with short Pb─I bond lengths, the PES is anharmonic, suggesting that the octahedral distortion in this regime is influenced by other LA geometry–induced perturbations (Fig. 1E).

As the lone pair activity originates from sp mixing under octahedral symmetry breaking (*12*, *43*), we further investigated its trend through electronic band structure calculations on representative compounds. We analyzed the electron localization function (ELF), a real-space descriptor of electron localization based on the Pauli principle and local kinetic energy density (*44*, *45*). ELF values range from 0 to 1, with values near 1 indicating strongly localized electrons and a value of 0.5 corresponding to a more delocalized, electron gas–like distribution. The ELF maps are obtained from ground-state density functional theory calculations on the basis of the experimentally determined single-crystal structures and therefore reflect the experimentally averaged structural configuration. In general, enhanced lone pair localization is manifested by ELF values slightly above 0.5 on the side opposite to the B-cation displacement or on the side associated with the longest B─I bond.

Lone pair localization as a function of 〈λ〉, $\bar{d}$, and B-cation identity can thus be analyzed by ELF maps, as shown in Fig. 1G and figs. S6 to S8. Within each B-cation series, we compared structures with varying $\bar{d}$ as well as those with similar $\bar{d}$ but different 〈λ〉. For example, (PEA)_2_PbI_4_, which has nearly regular octahedra (〈λ〉 = 1.0009) and a short $\bar{d}$ (3.176 Å), shows a nearly spherical ELF around the Pb atom, indicative of delocalized lone pairs. In contrast, (SBrMBA)_2_PbI_4_ [SBrMBA = *S*-1-(4-bromophenyl)ethan-1-ammonium] with a longer $\bar{d}$ (3.243 Å) and distorted octahedra (〈λ〉 = 1.0044) shows crescent-shaped ELF contours, signifying lone pair localization. (2FBMZ)_2_PbI_4_, which combines a short $\bar{d}$ (3.173 Å) similar to (PEA)_2_PbI_4_ and a large 〈λ〉 (1.0036) similar to (SBrMBA)_2_PbI_4_, exhibits very weak electron density localization. The ELF around Pb remains nearly spherical, with only a slight asymmetry relative to (PEA)_2_PbI_4_, and this subtle difference becomes apparent only upon closer inspection of the ELF value of 0.5 contours (fig. S9). The weak localization despite a large 〈λ〉 arises from the fact that the longest Pb─I bond in (2FBMZ)_2_PbI_4_ remains relatively short (3.173 Å).

To further probe the underlying sp mixing, we analyzed the orbital projected density of states (PDOS) and the valence electron density just below the Fermi energy (Fig. 1H and figs. S6 to S8) (*43*). Structures with regular octahedra (i.e., 〈λ〉 close to 1) show that the valence band maximum (VBM) contributed by the B-cation is dominated by ns orbitals [e.g., (PEA)_2_PbI_4_], while distorted octahedra show notable mixing of np orbitals [e.g., (2FBMZ)_2_PbI_4_ and (SBrMBA)_2_PbI_4_]. Both (2FBMZ)_2_PbI_4_ and (SBrMBA)_2_PbI_4_ exhibit similar degrees of sp mixing, although the former exhibits much weaker lone pair localization. This is because the shorter $\bar{d}$ in (2FBMZ)_2_PbI_4_ facilitates a stronger interaction between Pb 6s and I 5p orbitals, leading to a more delocalized lone pair despite comparable distortion. These trends are consistent across the Ge-, Sn-, and Pb-based series (figs. S6 to S8). For structures with the same spacer cation, the degree of lone pair localization follows the trend Ge > Sn > Pb, correlating with decreasing sp mixing. For example, the relative ns to np orbital contributions at the VBM are 4:1 in (SBrMBA)_2_GeI_4_, 5:1 in (SBrMBA)_2_SnI_4_, and 20:1 in (SBrMBA)_2_PbI_4_ (fig. S10).

### Exciton-phonon coupling

**Fig. 2.**
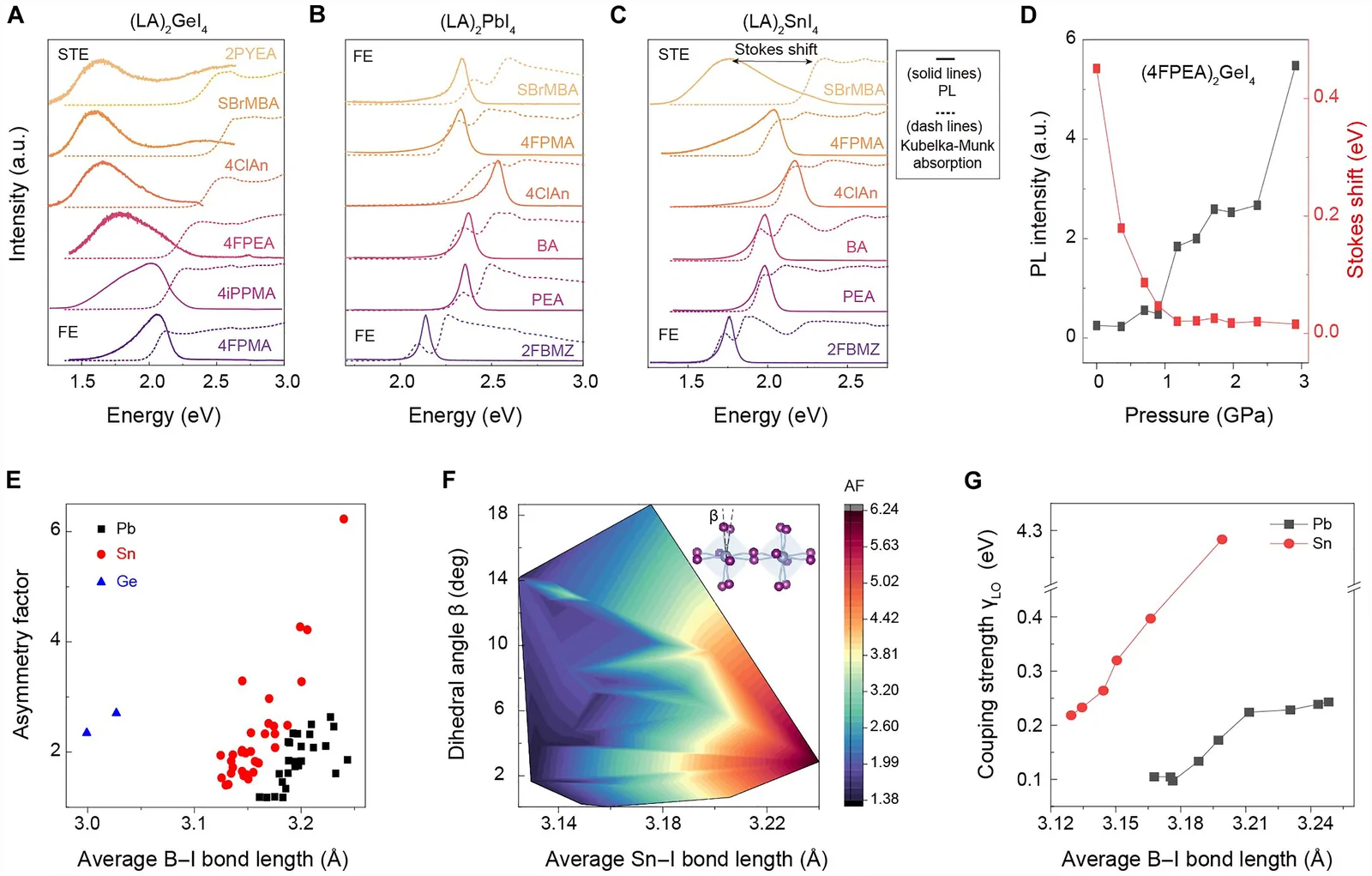
Impact of lone pair activity on exciton self-trapping and exciton-phonon coupling. (**A** to **C**) PL and Kubelka-Munk absorption spectra of representative (A) (LA)_2_GeI_4_, (B) (LA)_2_PbI_4_, and (C) (LA)_2_SnI_4_ structures, corresponding to selected points on the structural distortion map in Fig. 1C. 4FPMA, 4-fluorophenylmethylammonium; 4iPPMA, 4-isopropylbenzylammonium; 4FPEA, 4-fluorophenylethylammonium; 4ClAn, 4-chloroaniline cation; 2PYEA, 2-pyridylethylammonium. (**D**) Integrated PL emission intensity and Stokes shift of (4FPEA)_2_GeI_4_ under pressure, showing that lattice rigidification suppresses exciton self-trapping and promotes FE emission. (**E**) Correlation between FE emission AF (asymmetry factor) and average B─I bond length ($\bar{d}$), indicating enhanced exciton-phonon coupling with increasing $\bar{d}$ across the three B-cation series. (**F**) 2D color map of the AF value as a function of the average out-of-plane octahedral tilting angle and average Sn─I bond length for the Sn-based structures. The inset illustrates the definition of the apical I─Sn─Sn─I dihedral angle used to quantify the out-of-plane octahedral tilting. (**G**) Fitted exciton-phonon coupling strength ($\gamma_{\text{LO}}$) from temperature-dependent PL linewidth analysis as a function of the average metal iodide bond length for representative Pb- and Sn-based structures.

We next examined the trends of exciton-phonon coupling in selected (LA)_2_BI_4_ compounds and its impact on optical properties and correlation with structural parameters. Most (LA)_2_GeI_4_ compounds exhibit broadband emission with large Stokes shifts (Fig. 2A and fig. S11A), characteristic of self-trapped exciton (STE) arising from strong exciton-phonon coupling. In contrast, our examined 27 (LA)_2_PbI_4_ structures exhibit narrow PL peaks with small Stokes shifts, indicative of FE emission and weaker exciton-phonon coupling (Fig. 2B; see fig. S11B for PL spectra of all the structures). The (LA)_2_SnI_4_ series behaves as an intermediate case: While most of structures (30 of 36 examined structures) show FE emission, several with a larger ${\bar{d}}_{\text{Sn}─I}$ exhibit broadband STE emission (Fig. 2C; see fig. S11C for PL spectra of all the structures).

When comparing the same spacer across different B-cations in the FE regime, (LA)_2_SnI_4_ typically exhibits a larger PL peak width and more asymmetric PL shape (see fig. S12 for multiple representative spacers). The PL asymmetry can be quantified by asymmetry factor (AF), defined as the ratio of integrated PL intensity on the low-energy side to the high-energy side; larger AF values generally indicate stronger exciton-phonon coupling (*11*, *46*–*48*). Furthermore, a stronger tendency toward exciton localization from Pb to Sn to Ge is evident in the Stokes shifts. For example, in the SBrMBA series, the Stokes shift increases from 0.1 eV in (SBrMBA)_2_PbI_4_ to 0.7 eV in (SBrMBA)_2_SnI_4_ and 1 eV in (SBrMBA)_2_GeI_4_. These observations indicate that, although (LA)_2_SnI_4_ exhibit predominant FE emission, they display a stronger degree of localization than the Pb-based analogs while not reaching the strong STE regime characteristic of the Ge-based structures.

Within each B-cation series, we find that the B─I bond length is a primary structural factor governing exciton-phonon coupling. In the Ge series, while most compounds exhibit STE emission, certain structures—such as (4FPMA)_2_GeI_4_ (4FPMA = 4-fluorophenylmethylammonium)—display relatively narrow FE emission. These structures feature the shortest ${\bar{d}}_{\text{Ge}─I}$ and the most regular octahedra with Ge^2+^ centered within the octahedron (*16*, *49*). A systematic increase in Stokes shift and PL quenching is observed with increasing ${\bar{d}}_{\text{Ge}─I}$. This correlation is further supported by high-pressure PL studies on (4FPEA)_2_GeI_4_ (4FPEA = 4-fluorophenylethylammonium), which exhibits broad emission at ambient pressure. Such broad emission can be regulated by applying external pressure, which therefore is assigned to the formation of STE instead of permanent defects (*50*). Upon applying pressure up to 2 GPa, the PL emission transitions from STE to FE, accompanied by a 10-fold increase in PL intensity, a reduction in Stokes shift, and a more symmetric PL emission peak (Fig. 2D and fig. S13). These results indicate that lattice rigidification—achieved by shortening B─I bonds—effectively suppresses exciton self-trapping and promotes FE emission. This behavior can be understood from two perspectives. First, as shown in previous calculations, excited-state structures in halide perovskites exhibit strong first-order Jahn-Teller distortion of the metal halide octahedra, with notable elongation or compression of metal-halide bonds relative to the ground state (*51*). A more rigid inorganic framework with shorter B─I bonds would resist such distortion, thereby suppressing excited-state structural relaxation and reducing exciton-phonon coupling. Second, for structures with lone pair distortion in the ground state, sp mixing is eliminated in the excited state, favoring more symmetric octahedra. Thus, these would undergo greater structural relaxation, leading to stronger exciton-phonon coupling, as observed in metal halide polyhedral units (*52*, *53*).

Similar behavior of B─I bond length governing exciton-phonon coupling is observed in Sn- and Pb-based systems. In these systems, structures with a shorter $\bar{d}$ generally show smaller AF values (Fig. 2E and fig. S14), indicating weaker exciton-phonon coupling. In addition, we find that larger out-of-plane octahedral tilting—characterized by the apical I─B─B─I dihedral angle (β)—also increases AF in structures with a short $\bar{d}$ (Fig. 2F). However, the influence of out-of-plane tilting on exciton-phonon coupling is less pronounced than that of $\bar{d}$. To further quantify exciton-phonon coupling strength, we analyzed the temperature dependence of PL linewidths in representative Pb- and Sn-based structures (fig. S15 and tables S3 to S5) using the model $\Gamma \left(T\right)=\Gamma_{0}+\Gamma_{\text{LO}}=\Gamma_{0}+\gamma_{\text{LO}}/\left(e^{E_{\text{LO}}/k_{B}T}-1\right)$ (*54*), where $\Gamma_{0}$ is inhomogeneous broadening due to structural disorder, and $\Gamma_{\text{LO}}$ is the homogeneous broadening arising from longitudinal optical (LO) phonon scattering, characterized by coupling strength ($\gamma_{\text{LO}}$) and average phonon energy ($E_{\text{LO}}$). We find that linewidth broadening with temperature increases more rapidly in structures with a larger $\bar{d}$, and the fitted $\gamma_{\text{LO}}$ correlated positively with $\bar{d}$ (Fig. 2G), consistent with the AF trends. Furthermore, $\gamma_{\text{LO}}$ values of (LA)_2_SnI_4_ are generally higher than their Pb counterparts, reflecting enhanced exciton-phonon coupling in Sn-based structures. Together, these observations show that exciton-phonon coupling and exciton localization systematically increase either when progressing from Pb to Sn to Ge or when the B─I bond length increases within a given B-cation series.

### Exciton annihilation and diffusion

The above results establish that a rigid inorganic framework with a short $\bar{d}$ and minimal out-of-plane tilting suppresses exciton-phonon coupling and PL quenching, thereby enabling efficient FE emission. 2D perovskites incorporating PEA derivatives exhibit these structural features and have therefore been widely used as benchmarks for light-emitting devices (*19*, *29*, *55*). As a result, suppressed exciton-phonon coupling is commonly invoked to explain the better lasing performance (*20*, *33*). However, for high-brightness light-emitting diodes and lasers, EEA becomes the dominant nonradiative loss mechanism that limits light-emitting efficiency at high exciton densities (*40*, *56*, *57*). To investigate how lone pair activity influences EEA, we focused on four representative structures—(2FBMZ)_2_PbI_4_, (2FBMZ)_2_SnI_4_, (PEA)_2_PbI_4_, and (PEA)_2_SnI_4_—which feature short $\bar{d}$ values but differ in lone pair activity, lattice polarizability, and exciton dynamics.

**Fig. 3.**
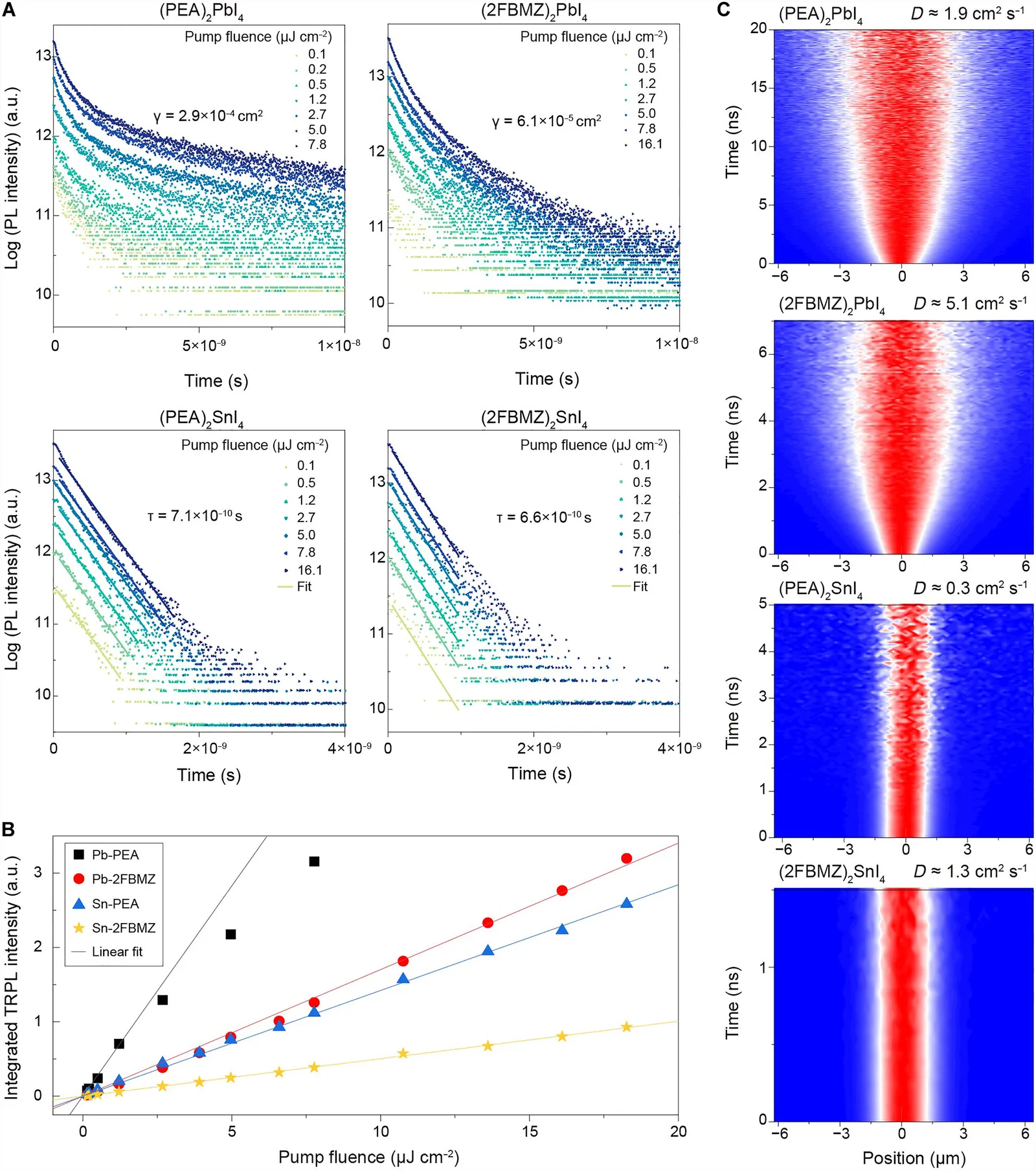
Impact of lone pair activity on exciton localization and annihilation at 80 K. (**A**) Power-dependent TRPL spectra plotted with PL intensity on a logarithmic scale and time on a linear scale of (PEA)_2_PbI_4_, (2FBMZ)_2_PbI_4_, (PEA)_2_SnI_4_, and (2FBMZ)_2_SnI_4_. Exciton recombination kinetics at high fluences are modeled using $\frac{\mathrm{dn}}{\mathrm{dt}}=-\frac{n}{\tau }-\gamma n^{2}$, where τ is the first-order exciton recombination lifetime and γ is the EEA rate. The fitting γ values are 2.9 × 10^−4^ and 6.1 × 10^−5^ cm^2^ s^−1^ for (PEA)_2_PbI_4_ and (2FBMZ)_2_PbI_4_, with corresponding τ values of 27 and 1.0 ns, respectively (fig. S16). The solid lines in TRPL of (PEA)_2_SnI_4_ and (2FBMZ)_2_SnI_4_ represent single-exponential fits $\left(\frac{\mathrm{dn}}{\mathrm{dt}}=-\frac{n}{\tau }\right)$ with τ values of 0.71 and 0.66 ns, respectively. (**B**) Integrated TRPL intensity versus pump fluence for (PEA)_2_PbI_4_, (2FBMZ)_2_PbI_4_, (PEA)_2_SnI_4_, and (2FBMZ)_2_SnI_4_. Solid lines denote linear fits constrained through the origin; the low-fluence regime is used for (PEA)_2_PbI_4_ because of sublinear behavior at high fluence. (**C**) Normalized spatially resolved PL and TRPL intensity profiles for (PEA)_2_PbI_4_, (2FBMZ)_2_PbI_4_, (PEA)_2_SnI_4_, and (2FBMZ)_2_SnI_4_ at various time delays. Exciton diffusivity (*D*) was extracted by fitting the temporal broadening of the PL intensity profile, revealing lower exciton diffusivity in Sn-based structures compared to Pb-based analogs. Note that broadening of the PL intensity profile is only observable within the first few hundred picoseconds, and this time window was used to extract *D* in (2FBMZ)_2_SnI_4_ (fig. S20).

Figure 3A and fig. S16 show power-dependent time-resolved PL (TRPL) spectra for (PEA)_2_PbI_4_, (2FBMZ)_2_PbI_4_, (PEA)_2_SnI_4_, and (2FBMZ)_2_SnI_4_, and the corresponding integrated PL intensity as a function of pump fluence is shown in Fig. 3B (corresponding to an exciton density of 10^11^ to 10^13^ cm^−2^). In (PEA)_2_PbI_4_, the integrated PL increases linearly at low fluences (i.e., monomolecular recombination dominant regime) but becomes sublinear above 0.5 × 10^13^ cm^−2^, indicating the onset of EEA, which is consistent with the decreased relative PL quantum yield for (PEA)_2_PbI_4_ at high fluences (fig. S17). The kinetic model at high fluences can be fitted by the following equation

$$\frac{\mathrm{dn}}{\mathrm{dt}}=-\frac{n}{\tau }-\gamma n^{2}$$

where n is the exciton density, τ is the first-order exciton recombination lifetime, and γ is the EEA rate constant. Fitting yields γ = 2.9 × 10^−4^ cm^2^ s^−1^ for (PEA)_2_PbI_4_, consistent with previous reports (figs. S16 and S18) (*40*, *41*). In stark contrast, (2FBMZ)_2_PbI_4_ exhibits a linear PL intensity increase and less change in PL lifetime across the same fluence range, suggesting suppressed EEA. Fitting the high-fluence data gives a much smaller γ = 6.1 × 10^−5^ cm^2^ s^−1^ (figs. S16 and S18). Femtosecond transient absorption spectroscopy on thin-film samples further supports this picture, showing a similar much weaker dependence of the decay kinetics on pump fluence in (2FBMZ)_2_PbI_4_ (fig. S19). The Sn-based structures exhibit markedly different exciton recombination dynamics from their Pb-based analogs. Both (2FBMZ)_2_SnI_4_ and (PEA)_2_SnI_4_ exhibit a linear PL intensity increase with increasing pump fluence. In addition, their TRPL spectra show characteristic of first-order recombination kinetics, with nearly unchanged PL lifetime as the pump fluence increases. These results indicate that EEA is strongly suppressed in the Sn-based structures.

EEA is a bimolecular process in which two excitons interact and one exciton recombines nonradiatively. In general, the EEA rate is governed not only by the exciton diffusion coefficient (*D*), which determines how frequently excitons encounter one another, but also by the effective annihilation radius (*R*), which sets the critical distance below which annihilation can occur (*40*, *56*, *58*–*61*). The relative importance of these two factors can vary across material systems, because the dielectric environment and exciton character also affect the effective interaction range (*56*, *58*, *62*–*64*). Because *R* is influenced by dielectric screening, both the exciton diffusion and dielectric response are considered in understanding the different EEA behavior.

We measured the exciton diffusion coefficients of the four structures using space-resolved PL and TRPL microscopy at 80 K, obtaining *D* values of 1.9 cm^2^ s^−1^ for (PEA)_2_PbI_4_, 5.1 cm^2^ s^−1^ for (2FBMZ)_2_PbI_4_, 0.3 cm^2^ s^−1^ for (PEA)_2_SnI_4_, and 1.3 cm^2^ s^−1^ for (2FBMZ)_2_SnI_4_ (Fig. 3C, fig. S20, and table S6). These values are reproducible across multiple independent samples and are on the same order of magnitude as reports on related compounds obtained using similar optical methods under cryogenic conditions (*55*, *65*, *66*). To minimize the possible influence of defects on the Sn-based structures (fig. S21), the synthesis, exfoliation, and transfer of the samples into the cryostat were all carried out in a nitrogen-filled glovebox. In addition, the excitation-density dependence of *D* was examined within the linear regime (fig. S20 and table S7). At a low excitation density, trap states more strongly affect the evolution of the PL intensity spatial profile, whereas at a higher excitation density, these trap states become filled (*67*, *68*), allowing the initial linear expansion regime to be resolved more clearly and the intrinsic exciton diffusion to be extracted more reliably. The Sn-based structures exhibit smaller *D* than their Pb-based analogs. However, band structure calculations reveal smaller carrier effective masses in Sn-based structures, which would normally imply higher exciton diffusivity (fig. S22 and table S8). The observed reduced *D* is therefore attributed to stronger exciton localization in the Sn-based structures, consistent with the exciton-phonon coupling results discussed above. This interpretation is further supported by electroabsorption spectroscopy and computational studies (*69*–*71*), which showed that excitons in Sn-based perovskites are more Frenkel-like, with smaller radii [e.g., 0.5 nm for (PEA)_2_SnI_4_ and 1.8 nm for (PEA)_2_PbI_4_], as a consequence of small polaron formation.

In addition to reduced exciton diffusion, dielectric screening also plays an important role in determining EEA. A larger dielectric constant more effectively screens exciton-exciton interactions and therefore reduces the effective annihilation radius (*40*, *56*, *58*, *63*). We calculated both the high frequency and static dielectric constants (ε) of the four structures. The influence of lone pair activity on the dielectric response is reflected in the difference between the high-frequency and static dielectric constants (*5*). The results show that (PEA)_2_SnI_4_ (static ε ∼ 13) and (2FBMZ)_2_SnI_4_ (static ε ∼ 51) have a larger static dielectric constant than (PEA)_2_PbI_4_ (static ε ∼ 8) and (2FBMZ)_2_PbI_4_ (static ε ∼ 13), respectively (table S9). The higher static ε in Sn structures is attributed to enhanced lattice polarizability arising from stronger lone pair activity. It should be noted that, although the Sn- and Pb-based structures exhibit similar octahedral distortions, the lone pair activity is stronger in the Sn compounds because the Pb 6s^2^ electron pair is more inert than the Sn 5s^2^ electron pair (*5*, *15*, *72*). Thus, both the reduced *D* and *R* contribute to the suppressed EEA in Sn-based structures.

By contrast, for the comparison between (2FBMZ)_2_BI_4_ and (PEA)_2_BI_4_, the 2FBMZ-based structures exhibit a higher *D* than the PEA-based analogs, likely due to their lower bandgaps. Their lower EEA rates therefore cannot be attributed to reduced diffusion. Instead, we attribute the suppressed EEA to the larger static dielectric constants of the 2FBMZ-based structures, which enhance dielectric screening and weaken exciton-exciton interactions. The difference in the high-frequency dielectric constant between (2FBMZ)_2_BI_4_ and (PEA)_2_BI_4_ is relatively small, whereas the difference in the static dielectric constant is much larger, indicating that the enhanced dielectric response of the 2FBMZ-based structures arises mainly from increased lattice polarization associated with stronger lone pair activity.

### Superior lasing performance

**Fig. 4.**
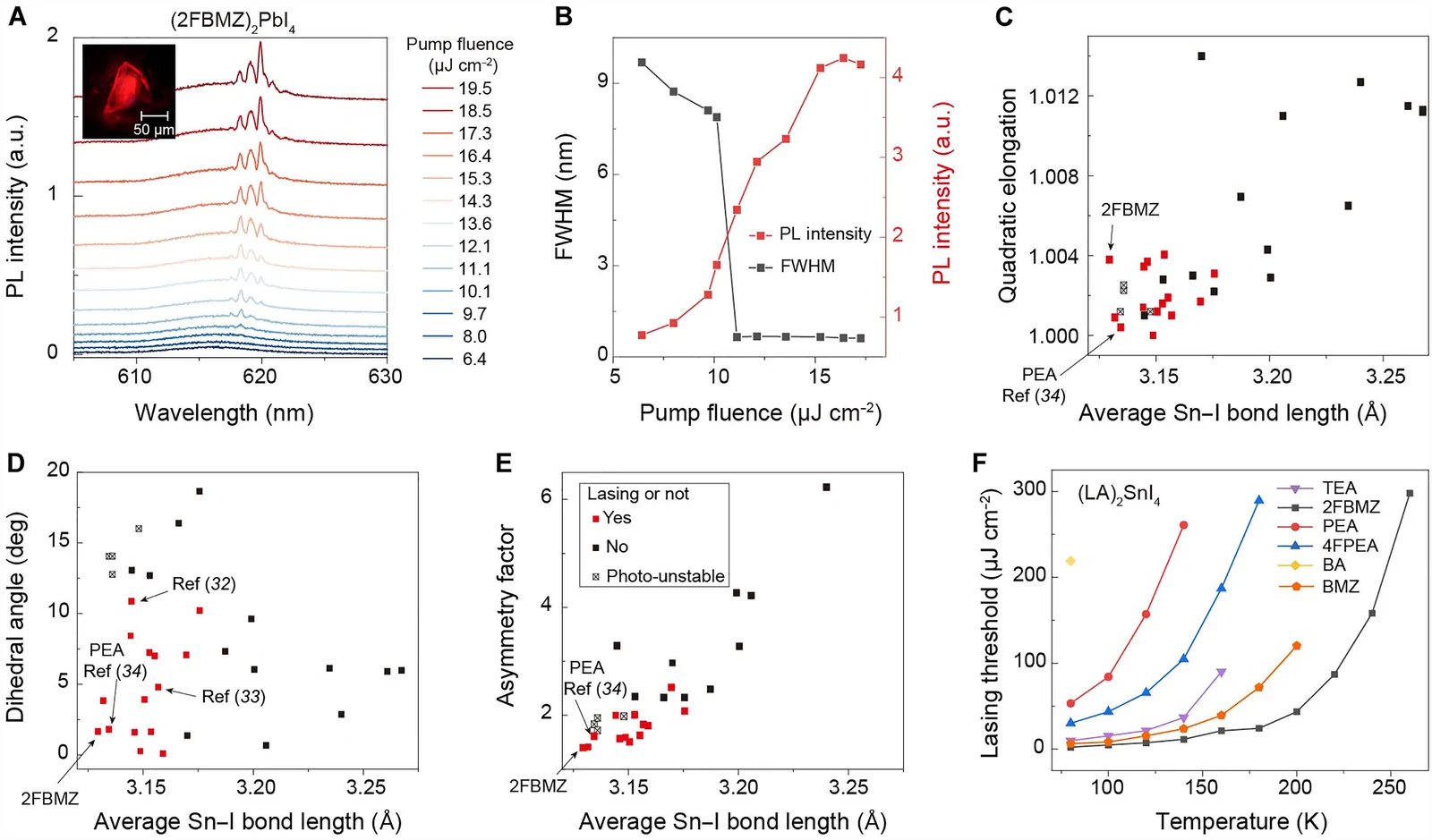
Superior lasing enabled by suppressed EEA in 2D perovskites. (**A**) Emission spectra of (2FBMZ)_2_PbI_4_ under increasing pump fluences at 80 K, showing a transition from spontaneous to lasing emission. The inset shows an optical image of the microcrystal above the lasing threshold. (**B**) FWHM and integrated PL intensity as a function of pump fluence at 80 K, revealing a lasing threshold at around 11 μJ cm^−2^. (**C**) Map of octahedral distortion versus Sn─I bond length, showing that lasing structures (red points) cluster in the region of short bond lengths. Black points indicate nonlasing structures, while empty crosses denote photo-unstable samples that degrade quickly even at 80 K, with only occasional transient lasing observed. (**D**) Map of out-of-plane octahedral tilting dihedral angle versus Sn─I bond length, with lasing structures located in the region with short bond lengths and small dihedral angles. (**E**) Map of PL AF versus Sn─I bond length, indicating that lasing structures cluster in the region with short bond lengths and low PL AFs. (**F**) Temperature-dependent lasing threshold of several representative (LA)_2_SnI_4_. (2FBMZ)_2_SnI_4_ demonstrates the most robust performance, sustaining lasing up to near room temperature.

We carried out lasing experiments on exfoliated single-crystal thin flakes using femtosecond pulsed laser excitation under cryogenic conditions. Under our experimental conditions, lasing was observed in (2FBMZ)_2_PbI_4_, whereas no lasing was detected in the benchmark (PEA)_2_PbI_4_ regardless of its structural morphology. Figure 4A presents a 2D color map of the PL spectra of (2FBMZ)_2_PbI_4_ under increasing pump fluences from 6 to 20 μJ cm^−2^ at 80 K. Figure 4B shows that the emission is broad below the lasing threshold (∼10 μJ cm^−2^), centered at ∼610 nm, with a full width at half maximum (FWHM) of ∼20 nm. The PL emission is red shifted relative to typical (LA)_2_PbI_4_. This is because the Pb─I bond lengths are short in (2FBMZ)_2_PbI_4_, and the in-plane and out-of-plane octahedral tilting is small, which enhances Pb─I orbital overlap and reduces the optical bandgap. Above the threshold, sharp lasing peaks emerge, accompanied by a steep increase in the integrated PL intensity and saturation of spontaneous emission—indicative of lasing (*33*). The dominant lasing peak appears at λ = 618.2 nm with an FWHM (δλ) of 0.6 nm, corresponding to a quality factor (*Q* = λ/δλ) of ∼940. We also tested several common (LA)_2_PbI_4_ structures, including (BA)_2_PbI_4_, (HA)_2_PbI_4_ (HA = hexylammonium), (PMA)_2_PbI_4_ (PMA = phenylmethylammonium), and (4FPEA)_2_PbI_4_, and none of them exhibited lasing. We attribute the lasing capability of (2FBMZ)_2_PbI_4_ to its unique combination of structural properties, which suppresses both exciton-phonon coupling and EEA.

Compared with the (LA)_2_PbI_4_ system, lasing is much more prevalent at 80 K among (LA)_2_SnI_4_, including the prototypical (BA)_2_SnI_4_ and (PEA)_2_SnI_4_ (fig. S23) Among the 32 (LA)_2_SnI_4_ we tested, 15 exhibited lasing, and these are predominantly located in the left-bottom region of structural maps with a short ${\bar{d}}_{\text{Sn}─I}$ (Fig. 4, C to E). A full list of examined structures, including two examples from the literature (*32*, *33*), is provided in table S10, and their PL spectra are shown in fig. S23. Despite stronger exciton-phonon coupling than their Pb counterparts (Fig. 2, E and G), Sn-based perovskites exhibit more localized FE and stronger dielectric screening that led to suppressed EEA, which we propose as the origin of their superior lasing performance. In addition, structures with a long ${\bar{d}}_{\text{Sn}─I}$ rarely lase, as they are associated with too much strong exciton-phonon coupling and PL quenching (Fig. 4C). This same limitation also prevents lasing in (LA)_2_GeI_4_. Furthermore, structures with a short ${\bar{d}}_{\text{Sn}─I}$ but large out-of-plane tilting (marked by black empty crosses) are less likely to lase because of severe photoinstability (Fig. 4D and fig. S24); they also exhibit larger AF values. When plotted as AF versus ${\bar{d}}_{\text{Sn}─I}$, lasing structures cluster in the region with both short ${\bar{d}}_{\text{Sn}─I}$ and low AF (Fig. 4E). Such prevalence in lasing in (LA)_2_SnI_4_ also correlates with the recent reports of lasing among various 2D Sn perovskites at cryogenic temperature (*32*–*34*), sometimes with thicker quantum wells (higher *n* value) (*31*).

We further measured the power-dependent PL spectra of (LA)_2_SnI_4_ (LA = PEA, 4FPEA, TEA, BA, 2FBMZ, and BMZ) at different temperatures (figs. S25 to S30). These structures display an increase in lasing threshold with rising temperature (Fig. 4F). Lasing typically ceases around 160 K because of thermal PL quenching arising from strong exciton-phonon coupling and associated nonradiative losses. We find that (2FBMZ)_2_SnI_4_ exhibits the most robust lasing that persists up to near room temperature, distinguishing it from other structures (*33*). The superior lasing performance is again attributed to its balancing of crystal structure rigidity (i.e., the shortest ${\bar{d}}_{\text{Sn}─I}$) and lone pair activity with enhanced dielectric screening, placing it in a unique structural regime similar to (2FBMZ)_2_PbI_4_ in the Pb-based system. In comparison, a structurally related compound, (BMZ)_2_SnI_4_, with similar octahedral distortion but longer ${\bar{d}}_{\text{Sn}─I}$ (due to differing packing distance of the spacer cation because of varying intermolecular interactions, as shown in fig. S31), shows slightly inferior lasing performance (fig. S26).

## DISCUSSION

In summary, we identify the key structural descriptors that control exciton behaviors in 2D perovskites and rationally design new 2D lead and tin iodide perovskites to enable optically pumped lasing. Within a given metal cation series, a rigid inorganic framework characterized by short metal-halide bonds generally suppresses lone pair activity and enables efficient FE emission. However, enhanced lone pair activity, modulated by the spacer cation and B-cation, can increase exciton localization and dielectric screening, thereby suppressing EEA, i.e., a major limiting factor for light-emitting performance at high exciton densities. By strategically balancing these two effects in a new rationally designed (2FBMZ)_2_PbI_4_, we achieved lasing in stark contrast to the benchmark (PEA)_2_PbI_4_ and further achieve superior lasing performance in its Sn-based analog including lasing up to near room temperature.

Our results show that exciton-phonon coupling alone is insufficient to account for lasing behavior in 2D perovskites. Instead, lone pair activity simultaneously modulates exciton-phonon coupling, exciton localization, and dielectric screening, and these effects together determine EEA and, consequently, lasing performance. This framework is particularly important for understanding the contrast between Pb- and Sn-based systems: Although Sn-based structures generally exhibit stronger exciton-phonon coupling, their enhanced exciton localization and dielectric screening more effectively suppress EEA, leading to improved lasing performance. These findings establish a unified structure-property framework for exciton dynamics in 2D perovskites and suggest that optimizing lasing requires balancing competing effects from tuning lone pair activity. For example, the spacer cations should be designed with an appropriate steric size to avoid excessive expansion of the inorganic framework while exhibiting strong intermolecular interactions to promote dense packing. Such packing can effectively compress the inorganic lattice, analogous to an internal pressure effect, thereby shortening the B─I bond length and suppressing excessive exciton-phonon coupling. In parallel, tuning lone pair activity offers an additional handle to enhance exciton localization and dielectric screening, reducing EEA and enabling high-efficiency lasing and other high-exciton-density photonic applications.

## MATERIALS AND METHODS

### Starting materials

Lead(II) iodide (PbI_2_; 99%, Macklin), tin(II) acetate [Sn(CH_3_COO)_2_; 95%, Bide Pharmatech Co., Ltd.], tin(II) iodide (SnI_2_; 99.99%, Liaoning Youxuan New Energy Technology Co., Ltd.), germanium oxide (GeO_2_; 99%, Macklin), hypophosphorous acid solution (H_3_PO_2_; 50 wt % in H_2_O, Macklin), hydriodic acid (HI) solution (55 wt % in H_2_O, stabilized with 1.5% hypophosphorous acid, Energy Chemical), *N*,*N*-dimethylformamide [DMF; high-performance liquid chromatography, Concord Technology (Tianjin) Co., Ltd.], and all amines—including 5,6-difluorobenzimidazole (95%, Bide Pharmatech Co., Ltd.), 2-phenylethylamine (98%, Adamas-beta), butylamine (99.5%, Sigma-Aldrich), 4-chloroaniline (98%, J&K Scientific), 4-fluorophenylmethylamine (97%, 9dingchem), (S)-1-(4bromophenyl)ethylamine (99.95%, Bidepharm), 4-fluorophenylethylamine (98%, Bide Pharmatech Co., Ltd.), 4-isopropylbenzylamine (98%, Rhawn), and 2-pyridylethylamine (98%, Rhawn), among others—were purchased commercially and used as received.

### Synthesis of 2D Ge-based perovskites

The GeI_2_ precursor solution was prepared by adding 0.5 mmol of GeO_2_ to 2.0 ml of a 1:1 mixture (volume ratio) of 55 wt % HI solution and 50 wt % H_3_PO_2_ solution in a capped vial. The mixture was heated to near boiling for 1 hour to reduce Ge^4+^ to Ge^2+^ and then allowed to cool to room temperature. The resulting GeI_2_ precursor appeared as a light-yellow solution. Subsequently, a stoichiometric amount of organic amine was added to the GeI_2_ precursor solution—1.0 mmol for the Ruddlesden-Popper phase with (LA)_2_GeI_4_ (LA is monoammonium) or 0.5 mmol for the Dion-Jacobson phase with (LA)GeI_4_ (LA is diammonium). The solution was then stirred and heated to 110°C until complete dissolution of all components. To grow crystals suitable for single-crystal x-ray diffraction measurements, the solution was kept at 70°C for 4 hours and then cooled to room temperature at a rate of 4°C per hour. Depending on the specific LA used, light-yellow to dark-red plate-like crystals were obtained. The synthesis was performed under ambient condition.

### Synthesis of 2D Sn-based perovskites

The SnI_2_ precursor solution was prepared by adding 0.5 mmol of Sn(CH_3_COO)_2_ to 2.0 ml of a 3:1 mixture (volume ratio) of 55 wt % HI solution and 50 wt % H_3_PO_2_ solution in a capped vial. The mixture was heated to near boiling until all solids completely dissolved and then cooled to room temperature. The SnI_2_ precursor solution appeared orange. A stoichiometric amount of the spacer amine was added to the SnI_2_ precursor solution, and the subsequent dissolution and crystallization steps were performed following the same procedure for 2D Ge-based perovskites. Depending on the specific amine used, plate-like crystals ranging in color from orange to near-black were obtained. Unless otherwise specified, the synthesis was carried out under ambient conditions. For the (PEA)_2_SnI_4_ and (2FBMZ)_2_SnI_4_ crystals used in the space-resolved PL and TRPL measurements, crystal growth was performed inside a nitrogen-filled acrylic glovebox equipped with an oxygen analyzer, with the oxygen concentration maintained below 200 ppm (parts per million).

### Synthesis of 2D Pb-based perovskites

PbI_2_ (0.5 mmol) and organic monoamine (1.0 mmol) were added to 2.0 ml of a 3:1 mixture (volume ratio) of 55 wt % HI solution and 50 wt % H_3_PO_2_ solution in a capped vial. The mixture was heated to 110°C until all reactants were completely dissolved. To grow crystals suitable for single-crystal x-ray diffraction, the solution was kept at 70°C for 4 hours and then cooled to room temperature at a rate of 4°C per hour. Depending on the specific organic amine used, plate-like crystals ranging from light yellow to red were obtained. The synthesis was carried out under ambient conditions. For the (PEA)_2_PbI_4_ and (2FBMZ)_2_PbI_4_ crystals used in the space-resolved PL and TRPL measurements, the samples were prepared by an evaporation-induced crystallization method in mixed aqueous-organic solvents, which has been shown to produce high-quality crystals suitable for probing exciton diffusion close to the intrinsic phonon-scattering limit (*73*). Specifically, 0.5 mmol of PbI_2_ were added to a mixture of 160 μl of HI solution and 2.0 ml of acetonitrile in a vial and then stirred and dissolved at room temperature. Then, 1.0 mmol of the target organic amine was added to the vial. The vial was then left open in a fume hood to allow solvent evaporation at room temperature for 24 hours, during which solvent evaporation led to crystal growth on the inner vial walls.

### Preparation of 2D perovskite thin films

First, we synthesized organic ammonium iodide salts: phenylethylamine hydroiodide (PEAI) and 5,6-difluorobenzimidazole hydroiodide (2FBMZI). To obtain PEAI and 2FBMZI, the corresponding amine was dissolved in 1.1 molar equivalents of HI. The solution was stirred and evaporated at 80°C, resulting in white precipitates for PEAI and gray precipitates for 2FBMZI. The powders were collected by underpressure distillation, washed with diethyl ether, and dried overnight in a vacuum oven. To prepare the (LA)_2_SnI_4_ (LA = PEA and 2FBMZ) thin films, 0.20 mmol of LAI and 0.10 mmol of SnI_2_ were dissolved in 1.0 ml of DMF and stirred for 2 hours at room temperature in a nitrogen-filled glovebox. The solution was then filtered through 0.22-μm polytetrafluoroethylene filters, spin coated on a glass substrate at 4000 rpm for 20 s, and annealed at 100°C for 5 min. For the (LA)_2_PbI_4_ (LA = PEA and 2FBMZ) thin films, 0.24 mmol of LAI and 0.12 mmol of PbI_2_ were dissolved in 1.0 ml of DMF and stirred for 1 hour at room temperature. The subsequent filtration, spin-coating, and annealing steps were identical for (LA)_2_SnI_4_ thin films. These thin films were used for steady-state transmission absorption and transient absorption measurements.

### Preparation and handling of 2D perovskite thin flakes

For the steady-state PL and reflectance spectral measurements of Sn- and Ge-based structures, the crystals were picked up by using a stick with a drop of oil, i.e., polybutylene, which directly transferred into the oil without exposure to the air. For the lasing experiments, the synthesized 2D perovskite crystals were separated from solution and dried through filter paper compression to remove residual solvent under ambient conditions. Subsequently, mechanical exfoliation was performed using Scotch tape to produce thin flakes with pristine, atomically flat surfaces. These exfoliated flakes were then transferred onto cryostat stages for the optically pumped lasing experiments. For the (PEA)_2_SnI_4_ and (2FBMZ)_2_SnI_4_ crystals used in the space-resolved PL and TRPL measurements, the exfoliation and transfer into the cryostat stage were performed inside a nitrogen-filled acrylic glovebox equipped with an oxygen analyzer, with the oxygen concentration maintained below 200 ppm.

### Single-crystal x-ray diffraction

Single-crystal x-ray diffraction experiments were performed at 290 K using an XtaLAB PRO 007HF (Mo) diffractometer equipped with Mo Kα radiation (λ = 0.71073 Å) operating at 50 kV and 24 mA. Data reduction and empirical absorption correction were performed using the CrysAlisPro program. The structures were solved by direct methods using the SHELXT program package in Olex2-1.5 software. All nonhydrogen atoms were located directly from the difference Fourier maps. Final structure refinements were performed using the SHELXL program in Olex2-1.5 software by minimizing the sum of squared deviations of *F*^2^ using a full-matrix technique. All structures passed full validation of CIF and structure factors via https://checkcif.iucr.org/.

### Steady-state Kubelka-Munk absorption spectra

Diffuse reflectance spectra of various powder samples were measured at room temperature using a Shimadzu UV-3600 ultraviolet-visible near-infrared spectrometer in the 300- to 900-nm region. BaSO_4_ was used as the 100% reflectance reference for the measurements. The absorption spectra were obtained by converting the reflectance data to absorption values using the Kubelka-Munk equation, $K/S=\frac{{\left(1-R\right)}^{2}}{2R}$, where R is the measured reflectance and K and S are the absorption and scattering coefficients, respectively.

### Steady-state PL spectra

The PL spectra of the samples were measured using a custom-built microscope system on the basis of an Olympus IX73 inverted microscope. A 405-nm continuous-wave laser (TEM-F-405nm) served as the excitation source and was focused onto the sample through an objective lens [40×, numerical aperture (NA) = 0.65, Olympus]. A 425-nm long-pass dichroic filter was used to direct the excitation light into the objective while blocking the reflected laser light. PL emission was collected by the same objective and directed through a set of achromatic lenses into a liquid nitrogen–cooled 2D charge-coupled device camera (Princeton Instruments, PyLoN-400BRX) equipped with a 150 g/mm grating. For temperature-dependent PL measurements, a cryostat (Scryo-S-300MS) was used to control the sample temperature in the range of 80 to 300 K.

### TRPL spectra and diffusion

TRPL spectroscopy and exciton diffusion measurements were performed on a modified Q2 Laser Scanning Confocal Microscope (ISS) setup equipped with a pulsed 405-nm laser (Omicron-Laserage QuixX 405-120; repetition rate, 20 MHz) as the excitation source. The 2D perovskite thin flake sample was placed in a cryostat (Scryo-S-300MS) and cooled to 80 K for low-temperature measurements. The excitation laser was focused onto the sample to the diffraction limit through a 40× achromatic objective lens (NA = 0.6, Nikon). The PL signal was collected through the same objective lens and then passed through the confocal microscope system (ISS) to achieve spatially resolved detection using Galvano mirrors for scanning. The PL signal was lastly detected by an avalanche photodiode (MPD PDM PD-100-CTE) connected to a time-correlated single-photon counting system for TRPL decay measurements. TRPL intensity data were collected in 50-ns windows with 1024 temporal bins.

#### Analysis of exciton diffusion

The spatiotemporal evolution of exciton density n(x,t) was modeled using a 1D diffusion-recombination equation: $\frac{\partial n\left(x,t\right)}{\partial t}=D\frac{\partial^{2}n\left(x,t\right)}{\partial x^{2}}-k_{\text{rc}}n\left(x,t\right)$, where D is the exciton diffusivity and $k_{\text{rc}}$ is the recombination rate. The solution is expressed as a convolution of the initial exciton distribution $n\left(\tilde{x},0\right)$ with a time-dependent Gaussian broadening function G(x,t): $n\left(x,t\right)\propto \int_{-\infty }^{\infty }n\left(\tilde{x},0\right)\cdot \frac{1}{\sqrt{4\pi \mathrm{Dt}}}\cdot \text{exp}\left[-\frac{{\left(x-\tilde{x}\right)}^{2}}{4\mathrm{Dt}}\right]d\tilde{x}$. Experimentally, the spatial PL profile was fitted using a Voigt function to account for both Gaussian broadening (from diffusion) and Lorentzian broadening (due to the diffraction limit of the optical components and the instability of the instrument) (*68*): n(x,t)∝L(x)∗G(x,t), where the Gaussian PL distribution $\sigma_{G}^{2}$ increases linearly with time: $\sigma_{G}^{2}\left(t\right)=\sigma_{G}^{2}\left(0\right)+2\mathrm{Dt}$. The exciton diffusivity D was extracted from the slope of $\sigma_{G}^{2}$ as a function of time.

#### Fitting of TRPL kinetics

The exciton recombination kinetics were analyzed using the model $\frac{\mathrm{dn}}{\mathrm{dt}}=-\frac{n}{\tau }-\gamma n^{2}$, where n is the exciton density, τ is the first-order exciton recombination lifetime, and γ is the EEA rate. The temporal PL intensity was assumed to be proportional to the exciton density. The initial PL intensity was found to scale linearly with the excitation laser power (P) and was used to obtain the conversion factor between the exciton density and the PL intensity. The exciton density (number per unit area per pulse) was estimated as $n=\frac{4P}{\pi d^{2}fE_{\text{photon}}}$, where f is the laser repetition rate (20 MHz), $E_{\text{photon}}$ is the photon energy (405 nm), and d is the excitation spot diameter. The spot diameter d was determined from the initial PL spatial profile, which follows a Gaussian distribution, by measuring the full width at $1/e^{2}$ maximum PL intensity.

### Transient absorption measurement

Transient absorption spectra were collected using an ultrafast transient absorption spectrometer (TIME-TECH SPECTRA). The output from a femtosecond laser (100 kHz) was split into two beams. One beam was directed through an optical parametric amplifier to generate the pump light at 400 nm. The other beam was frequency doubled by a β-barium borate crystal to 515 nm and then broadened into a broadband probe light (400 to 680 nm) using a sapphire crystal. A mechanical translation stage (PLX) was used to adjust the time delay of the probe relative to the pump. The pump light, modulated by a chopper (THORLABS), was cofocused with the probe light onto the sample using an objective lens (NA = 0.45, MITUTOYO). The transmitted signal, after passing through a 425-nm long-pass filter (THORLABS), was collected by another objective lens (NA = 0.45, MITUTOYO) and directed into a detector (SV230712, TIME-TECH SPECTRA) for measurement.

### Optically pumped lasing measurements

Lasing measurements on 2D perovskite thin flakes at low temperature were conducted with a cryostat (Scryo-S-300MS). The experiments were carried out with a custom-built microscope system on the basis of an Olympus IX73 inverted microscope. Excitation pulses at 400 nm (repetition rate: 200 Hz) were generated by an optical parametric amplifier (ORPHEUS-HP), which is seeded by a femtosecond laser (PHAROS-PH2-20 W, 1030 nm, 290 fs, 200 kHz). The excitation beam was expanded with a diameter of ∼67 μm using a lens before entering the microscope. The exciton power was adjusted with a neutral density filter and monitored with a power meter (THORLABS, PM100D). The PL emission spectra from the sample were collected through a set of achromatic lenses, directed through the entrance slit of a spectrometer (HRS-300S) equipped with a 1200 grooves/mm grating, and recorded by a liquid nitrogen–cooled 2D charge-coupled device camera (Princeton Instruments, PyLoN-400BRX).

### High-pressure optical measurements

The high-pressure environment was generated using symmetrical diamond anvil cells equipped with ultralow-fluorescence diamonds with a culet size of 400 μm. The high-pressure sample chamber was prepared by pre-indenting a T301 gasket to a thickness of about 50 μm and laser-drilling a central hole with a diameter of about 250 μm. The sample and a ruby ball (used for pressure calibration) were loaded into the chamber, and the pressure was determined using the ruby fluorescence method (*74*). Silicone oil was used as the pressure-transmitting medium. Pressure-dependent PL measurements were performed on a home-built spectroscopy system. A stabilized 488-nm continuous-wave laser (Cobolt) was used as the excitation source. The PL emission spectra were collected by a long working distance objective lens (20×, Mitutoyo, NA = 0.42), passed into a spectrograph (HRS-300S, Princeton Instruments), and recorded using a charge-coupled device camera (PIXIS: 100BR_eXcelon, Princeton Instruments). For the transmission absorption measurements, a stabilized tungsten-halogen lamp (SLS201L, Thorlabs) was used as the light source, and the spectra were collected by a spectrometer (Ideaoptics).

### Calculation methods

All calculations were performed using the Vienna Ab-initio Simulation Package (VASP) (*75*). The projector augmented wave formalism (*76*) and a planewave basis set with a cutoff energy of 500 eV were used. The Perdew-Burke-Ernzerhof (*77*) functional was used with D3 (*78*, *79*) correction to account for the van der Waals interaction. The valence electrons taken into consideration are as follows: Ge, 3d^10^4s^2^4p^2^; Sn, 4d^10^5s^2^5p^2^; Pb, 5d^10^6s^2^6p^2^; F, 2s^2^2p^5^; Br, 4s^2^4p^5^; I, 5s^2^5p^5^; Cs, 5s^2^5p^6^6s^1^; H, 1s^1^; C, 2s^2^2p^2^; N, 2s^2^2p^3^; O, 2s^2^2p^4^.

In the calculation of ELF, orbital PDOS, and band structures, we take the experimental crystal structures and relax the hydrogen atoms using VASP. The force tolerance was set to 0.02 eV/Å. The ELFs were calculated with Silvi and Savin’s recipe (*45*), as was implemented in VASP. In PDOS calculations, the Monkhorst-Pack *k*-mesh was generated using the VASPKIT package (*80*) with a *k*-space resolution of 0.03 Å^−1^ and the energy convergence criterion set to 10^−8^ eV.

The PES calculations were carried out as described in our previous study (*16*). Briefly, we used a simplified model of a 2D perovskite, where the organic spacer cation was replaced by an inorganic cesium cation, resulting in the structure Cs_2_BI_4_ (B = Ge^2+^, Sn^2+^, and Pb^2+^). This model assumes a tetragonal phase and no octahedral tilting, with out-of-plane tilting angles set to zero and linear bridging B─I─B bond angles of 180°. The lattice parameters were defined as *a* = *b* = $2d_{B─I}$ and *c* = $5d_{B─I}$, where $d_{B─I}$ is the B─I bond length. The PESs were computed by scanning the B-cation off-center displacement along the in-plane (110) direction from 0 to 0.700 Å in 0.050-Å increments for a range of bond lengths. Specifically, the bond length $d_{B─I}$ was varied from 2.95 to 3.23 Å (Cs_2_GeI_4_), 3.05 to 3.33 Å (Cs_2_SnI_4_), and 3.10 to 3.38 Å (Cs_2_PbI_4_) in 0.04-Å steps. For each bond length, the minimum-energy structure identified from the PES was then used to analyze the octahedral distortion as a function of bond length.

For the calculations of band structure and carrier effective mass, VASPKIT was used to generate high-symmetry paths and postprocessing data. For dielectric constant calculations, both atomic positions and the unit cell were fully relaxed with a tighter force convergence criterion of 0.005 eV/Å to minimize imaginary frequencies. The calculations were carried out using density functional perturbation theory (*81*, *82*) with a *k*-mesh resolution of 0.04 Å^−1^.

The intermolecular forces were calculated as follows: The Multiwfn program (*83*, *84*) was used to perform the IGMH analysis (*85*, *86*), in which the wave functions were derived using the CP2K code (*87*) with the Goedecker-Teter-Hutter pseudopotentials (*88*) and DZVP-MOLOPT-SR-GTH basis sets (*89*). In addition, the Hirshfeld surface analysis (*86*, *90*) is also performed using Multiwfn.

## References

[1] G. Laurita, R. Seshadri, Chemistry, structure, and function of lone pairs in extended solids. Acc. Chem. Res. 55, 1004–1014 (2022). 10.1021/acs.accounts.1c00741 35319202

[2] A. Walsh, D. J. Payne, R. G. Egdell, G. W. Watson, Stereochemistry of post-transition metal oxides: Revision of the classical lone pair model. Chem. Soc. Rev. 40, 4455–4463 (2011). 10.1039/c1cs15098g 21666920

[3] K. M. McCall, V. Morad, B. M. Benin, M. V. Kovalenko, Efficient lone-pair-driven luminescence: Structure–property relationships in emissive 5s^2^ metal halides. ACS Mater. Lett. 2, 1218–1232 (2020). 10.1021/acsmaterialslett.0c00211 PMC7491574 32954359

[4] A. K. Jena, A. Kulkarni, T. Miyasaka, Halide perovskite photovoltaics: Background, status, and future prospects. Chem. Rev. 119, 3036–3103 (2019). 10.1021/acs.chemrev.8b00539 30821144

[5] Y. Fu, S. Jin, X.-Y. Zhu, Stereochemical expression of ns^2^ electron pairs in metal halide perovskites. Nat. Rev. Chem. 5, 838–852 (2021). 10.1038/s41570-021-00335-9 37117392

[6] D. H. Fabini, R. Seshadri, M. G. Kanatzidis, The underappreciated lone pair in halide perovskites underpins their unusual properties. MRS Bull. 45, 467–477 (2020).

[7] I. Mosquera-Lois, Y.-T. Huang, H. Lohan, J. Ye, A. Walsh, R. L. Z. Hoye, Multifaceted nature of defect tolerance in halide perovskites and emerging semiconductors. Nat. Rev. Chem. 9, 287–304 (2025). 10.1038/s41570-025-00702-w 40195529

[8] T. Li, S. Luo, X. Wang, L. Zhang, Alternative lone-pair ns^2^-cation-based semiconductors beyond lead halide perovskites for optoelectronic applications. Adv. Mater. 33, e2008574 (2021). 10.1002/adma.202008574 34060151

[9] M. S. Hammer, H. Schlott, L. Lüer, C. J. Brabec, M. Sytnyk, J. Will, B. Meyer, W. Heiss, Bridging theory and experiment in defect-tolerant semiconductors for photovoltaics. Nat. Rev. Mater. 10, 311–325 (2025).

[10] X. Huang, X. Li, Y. Tao, S. Guo, J. Gu, H. Hong, Y. Yao, Y. Guan, Y. Gao, C. Li, X. Lü, Y. Fu, Understanding electron–phonon interactions in 3d lead halide perovskites from the stereochemical expression of 6s^2^ lone pairs. J. Am. Chem. Soc. 144, 12247–12260 (2022). 10.1021/jacs.2c03443 35767659

[11] J. Gu, Y. Tao, T. Fu, S. Guo, X. Jiang, Y. Guan, X. Li, C. Li, X. Lü, Y. Fu, Correlating photophysical properties with stereochemical expression of 6s^2^ lone pairs in two-dimensional lead halide perovskites. Angew. Chemie Int. Ed. Engl. 62, e202304515 (2023). 10.1002/anie.202304515 37235527

[12] X. Jiang, Y. Tao, J. Gu, L. Jin, C. Li, W. Zhang, Y. Fu, Broadband emission originating from the stereochemical expression of 6s^2^ lone pairs in two-dimensional lead bromide perovskites. Dalt. Trans. 52, 15489–15495 (2023). 10.1039/d3dt01627g 37552134

[13] Y. Fu, H. Lohan, M. Righetto, Y. T. Huang, S. R. Kavanagh, C. W. Cho, S. J. Zelewski, Y. W. Woo, H. Demetriou, M. A. McLachlan, S. Heutz, B. A. Piot, D. O. Scanlon, A. Rao, L. M. Herz, A. Walsh, R. L. Z. Hoye, Structural and electronic features enabling delocalized charge-carriers in CuSbSe_2_. Nat. Commun. 16, 65 (2025). 10.1038/s41467-024-55254-2 PMC11697385 39747002

[14] J. Han, Y. Li, P. Zhang, B. Xu, X. Xu, Z. Quan, Cooperative regulation of ns^2^ lone-pair expression realizes distinct excitonic emissions in hybrid germanium, tin, and lead halides. J. Am. Chem. Soc. 147, 1291–1299 (2025). 10.1021/jacs.4c15554 39688853

[15] D. H. Fabini, G. Laurita, J. S. Bechtel, C. C. Stoumpos, H. A. Evans, A. G. Kontos, Y. S. Raptis, P. Falaras, A. Van der Ven, M. G. Kanatzidis, R. Seshadri, Dynamic stereochemical activity of the Sn^2+^ lone pair in perovskite CsSnBr_3_. J. Am. Chem. Soc. 138, 11820–11832 (2016). 10.1021/jacs.6b06287 27583813

[16] X. Li, Y. Tao, X. Jiang, G. Cai, J. Gu, N. Zheng, Y. Guan, W. Zhang, X. Li, J. Su, Z. Liu, Z. Bian, J. Sun, C. Li, Y. Fu, Optical bandgap anomaly with tuning dimensionality in germanium perovskites: Interplay between quantum confinement and lone pair expression. Chem 10, 891–909 (2024).

[17] B. Saparov, D. B. Mitzi, Organic–inorganic perovskites: Structural versatility for functional materials design. Chem. Rev. 116, 4558–4596 (2016). 10.1021/acs.chemrev.5b00715 27040120

[18] X. Li, J. M. Hoffman, M. G. Kanatzidis, The 2D halide perovskite rulebook: How the spacer influences everything from the structure to optoelectronic device efficiency. Chem. Rev. 121, 2230–2291 (2021). 10.1021/acs.chemrev.0c01006 33476131

[19] X. Gong, O. Voznyy, A. Jain, W. Liu, R. Sabatini, Z. Piontkowski, G. Walters, G. Bappi, S. Nokhrin, O. Bushuyev, M. Yuan, R. Comin, D. McCamant, S. O. Kelley, E. H. Sargent, Electron–phonon interaction in efficient perovskite blue emitters. Nat. Mater. 17, 550–556 (2018). 10.1038/s41563-018-0081-x 29760510

[20] J. Y. Park, R. Song, J. Liang, L. Jin, K. Wang, S. Li, E. Shi, Y. Gao, M. Zeller, S. J. Teat, P. Guo, L. Huang, Y. S. Zhao, V. Blum, L. Dou, Thickness control of organic semiconductor-incorporated perovskites. Nat. Chem. 15, 1745–1753 (2023). 10.1038/s41557-023-01311-0 37653228

[21] H. Zhu, Y. Fu, F. Meng, X. Wu, Z. Gong, Q. Ding, M. V. Gustafsson, M. T. Trinh, S. Jin, X.-Y. Zhu, Lead halide perovskite nanowire lasers with low lasing thresholds and high quality factors. Nat. Mater. 14, 636–642 (2015). 10.1038/nmat4271 25849532

[22] G. Xing, N. Mathews, S. S. Lim, N. Yantara, X. Liu, D. Sabba, M. Grätzel, S. Mhaisalkar, T. C. Sum, Low-temperature solution-processed wavelength-tunable perovskites for lasing. Nat. Mater. 13, 476–480 (2014). 10.1038/nmat3911 24633346

[23] Q. Zhang, S. T. Ha, X. Liu, T. C. Sum, Q. Xiong, Room-temperature near-infrared high-Q perovskite whispering-gallery planar nanolasers. Nano Lett. 14, 5995–6001 (2014). 10.1021/nl503057g 25118830

[24] S. W. Eaton, M. Lai, N. A. Gibson, A. B. Wong, L. Dou, J. Ma, L.-W. Wang, S. R. Leone, P. Yang, Lasing in robust cesium lead halide perovskite nanowires. Proc. Natl. Acad. Sci. U.S.A. 113, 1993–1998 (2016). 10.1073/pnas.1600789113 PMC4776493 26862172

[25] R. Su, A. Fieramosca, Q. Zhang, H. S. Nguyen, E. Deleporte, Z. Chen, D. Sanvitto, T. C. H. Liew, Q. Xiong, Perovskite semiconductors for room-temperature exciton-polaritonics. Nat. Mater. 20, 1315–1324 (2021). 10.1038/s41563-021-01035-x 34211156

[26] T. Kondo, T. Azuma, T. Yuasa, R. Ito, Biexciton lasing in the layered perovskite-type material (C_6_H_13_NH_3_)_2_PbI_4_. Solid State Commun. 105, 253–255 (1998).

[27] W. K. Chong, K. Thirumal, D. Giovanni, T. W. Goh, X. Liu, N. Mathews, S. Mhaisalkar, T. C. Sum, Dominant factors limiting the optical gain in layered two-dimensional halide perovskite thin films. Phys. Chem. Chem. Phys. 18, 14701–14708 (2016). 10.1039/c6cp01955b 27184073

[28] C. M. Raghavan, T.-P. Chen, S.-S. Li, W.-L. Chen, C.-Y. Lo, Y.-M. Liao, G. Haider, C.-C. Lin, C.-C. Chen, R. Sankar, Y.-M. Chang, F.-C. Chou, C.-W. Chen, Low-threshold lasing from 2D homologous organic–inorganic hybrid Ruddlesden–Popper perovskite single crystals. Nano Lett. 18, 3221–3228 (2018). 10.1021/acs.nanolett.8b00990 29694049

[29] H. Zhang, Y. Hu, W. Wen, B. Du, L. Wu, Y. Chen, S. Feng, C. Zou, J. Shang, H. J. Fan, T. Yu, Room-temperature continuous-wave vertical-cavity surface-emitting lasers based on 2D layered organic–inorganic hybrid perovskites. APL Mater. 9, 071106 (2021).

[30] M. Gomez-Dominguez, V. Quirós-Cordero, E. Rojas-Gatjens, K. A. Koch, E. J. Kumar, C. A. R. R. Perini, N. Stingelin, C. Silva-Acuña, A. R. Srimath Kandada, V. Menon, J.-P. P. Correa-Baena, Multiple emission peaks challenge polariton condensation in phenethylammonium-based 2D perovskite microcavities. ACS Photonics 12, 2423–2431 (2025). 10.1021/acsphotonics.4c02065 PMC12100713 40416319

[31] Y. Liang, Q. Shang, Q. Wei, L. Zhao, Z. Liu, J. Shi, Y. Zhong, J. Chen, Y. Gao, M. Li, X. Liu, G. Xing, Q. Zhang, Lasing from mechanically exfoliated 2D homologous Ruddlesden–Popper perovskite engineered by inorganic layer thickness. Adv. Mater. 31, e1903030 (2019). 10.1002/adma.201903030 31408551

[32] W. Shao, J. H. Kim, J. Simon, Z. Nian, S.-D. Baek, Y. Lu, C. B. Fruhling, H. Yang, K. Wang, J. Y. Park, L. Huang, Y. Yu, A. Boltasseva, B. M. Savoie, V. M. Shalaev, L. Dou, Molecular templating of layered halide perovskite nanowires. Science 384, 1000–1006 (2024). 10.1126/science.adl0920 38815024

[33] Y. Li, H. Zhou, M. Xia, H. Shen, T. Wang, H. Gao, X. Sheng, Y. Han, Z. Chen, L. Dou, H. Zhu, E. Shi, Phase-pure 2D tin halide perovskite thin flakes for stable lasing. Sci. Adv. 9, eadh0517 (2023). 10.1126/sciadv.adh0517 PMC11801371 37556538

[34] A. L. Alvarado-Leaños, D. Cortecchia, C. N. Saggau, S. Martani, G. Folpini, E. Feltri, M. D. Albaqami, L. Ma, A. Petrozza, Lasing in two-dimensional tin perovskites. ACS Nano 16, 20671–20679 (2022). 10.1021/acsnano.2c07705 PMC9798858 36420860

[35] H. Kim, S. Z. Uddin, N. Higashitarumizu, E. Rabani, A. Javey, Inhibited nonradiative decay at all exciton densities in monolayer semiconductors. Science 373, 448–452 (2021). 10.1126/science.abi9193 34437119

[36] S. Z. Uddin, E. Rabani, A. Javey, Universal inverse scaling of exciton–exciton annihilation coefficient with exciton lifetime. Nano Lett. 21, 424–429 (2021). 10.1021/acs.nanolett.0c03820 33320011

[37] D. Sun, Y. Rao, G. A. Reider, G. Chen, Y. You, L. Brézin, A. R. Harutyunyan, T. F. Heinz, Observation of rapid exciton–exciton annihilation in monolayer molybdenum disulfide. Nano Lett. 14, 5625–5629 (2014). 10.1021/nl5021975 25171389

[38] Y. Yu, Y. Yu, C. Xu, A. Barrette, K. Gundogdu, L. Cao, Fundamental limits of exciton-exciton annihilation for light emission in transition metal dichalcogenide monolayers. Phys. Rev. B 93, 201111 (2016).

[39] T. J. Sheehan, S. Saris, W. A. Tisdale, Exciton transport in perovskite materials. Adv. Mater. 37, 2415757 (2025). 10.1002/adma.202415757 39737764

[40] S. Deng, E. Shi, L. Yuan, L. Jin, L. Dou, L. Huang, Long-range exciton transport and slow annihilation in two-dimensional hybrid perovskites. Nat. Commun. 11, 664 (2020). 10.1038/s41467-020-14403-z PMC6994693 32005840

[41] G. Delport, G. Chehade, F. Lédée, H. Diab, C. Milesi-Brault, G. Trippé-Allard, J. Even, J.-S. Lauret, E. Deleporte, D. Garrot, Exciton–exciton annihilation in two-dimensional halide perovskites at room temperature. J. Phys. Chem. Lett. 10, 5153–5159 (2019). 10.1021/acs.jpclett.9b01595 31415177

[42] X. Li, Y. Guan, X. Li, Y. Fu, Stereochemically active lone pairs and nonlinear optical properties of two-dimensional multilayered tin and germanium iodide perovskites. J. Am. Chem. Soc. 144, 18030–18042 (2022). 10.1021/jacs.2c07535 36134903

[43] D. B. Straus, H. E. Mitchell Warden, R. J. Cava, s–p mixing in stereochemically active lone pairs drives the formation of 1D chains of lead bromide square pyramids. Inorg. Chem. 60, 12676–12680 (2021). 10.1021/acs.inorgchem.1c01277 34375087

[44] R. Seshadri, N. A. Hill, Visualizing the role of Bi 6s “Lone Pairs” in the off-center distortion in ferromagnetic BiMnO_3_. Chem. Mater. 13, 2892–2899 (2001).

[45] B. Silvi, A. Savin, Classification of chemical bonds based on topological analysis of electron localization functions. Nature 371, 683–686 (1994).

[46] M. Schreiber, Y. Toyozawa, Numerical experiments on the absorption lineshape of the exciton under lattice vibrations. III. The Urbach rule. J. Physical Soc. Japan 51, 1544–1550 (1982).

[47] H. Sumi, Y. Toyozawa, Urbach-Martienseen rule and exciton trapped momentarily by lattice vibrations. J. Physical Soc. Japan 31, 342–358 (1971).

[48] S. Guo, W. Mihalyi-Koch, Y. Mao, X. Li, K. Bu, H. Hong, M. P. Hautzinger, H. Luo, D. Wang, J. Gu, Y. Zhang, D. Zhang, Q. Hu, Y. Ding, W. Yang, Y. Fu, S. Jin, X. Lü, Exciton engineering of 2D Ruddlesden–Popper perovskites by synergistically tuning the intra and interlayer structures. Nat. Commun. 15, 3001 (2024). 10.1038/s41467-024-47225-4 PMC11001939 38589388

[49] A. Morteza Najarian, F. Dinic, H. Chen, R. Sabatini, C. Zheng, A. Lough, T. Maris, M. I. Saidaminov, F. P. García de Arquer, O. Voznyy, S. Hoogland, E. H. Sargent, Homomeric chains of intermolecular bonds scaffold octahedral germanium perovskites. Nature 620, 328–335 (2023). 10.1038/s41586-023-06209-y 37438526

[50] X. Lü, C. Stoumpos, Q. Hu, X. Ma, D. Zhang, S. Guo, J. Hoffman, K. Bu, X. Guo, Y. Wang, C. Ji, H. Chen, H. Xu, Q. Jia, W. Yang, M. G. Kanatzidis, H.-K. Mao, Regulating off-centering distortion maximizes photoluminescence in halide perovskites. Natl. Sci. Rev. 8, nwaa288 (2021). 10.1093/nsr/nwaa288 PMC8433095 34691729

[51] X. Wang, W. Meng, W. Liao, J. Wang, R.-G. Xiong, Y. Yan, Atomistic mechanism of broadband emission in metal halide perovskites. J. Phys. Chem. Lett. 10, 501–506 (2019). 10.1021/acs.jpclett.8b03717 30642179

[52] V. Morad, Y. Shynkarenko, S. Yakunin, A. Brumberg, R. D. Schaller, M. V. Kovalenko, Disphenoidal zero-dimensional lead, tin, and germanium halides: Highly emissive singlet and triplet self-trapped excitons and X-ray scintillation. J. Am. Chem. Soc. 141, 9764–9768 (2019). 10.1021/jacs.9b02365 31244134

[53] H. Nikol, A. Becht, A. Vogler, Photoluminescence of germanium(II), tin(II), and lead(II) chloride complexes in solution. Inorg. Chem. 31, 3277–3279 (1992).

[54] A. D. Wright, C. Verdi, R. L. Milot, G. E. Eperon, M. A. Pérez-Osorio, H. J. Snaith, F. Giustino, M. B. Johnston, L. M. Herz, Electron–phonon coupling in hybrid lead halide perovskites. Nat. Commun. 7, 11755 (2016). 10.1038/ncomms11755 PMC4894981 27225329

[55] J. D. Ziegler, J. Zipfel, B. Meisinger, M. Menahem, X. Zhu, T. Taniguchi, K. Watanabe, O. Yaffe, D. A. Egger, A. Chernikov, Fast and anomalous exciton diffusion in two-dimensional hybrid perovskites. Nano Lett. 20, 6674–6681 (2020). 10.1021/acs.nanolett.0c02472 32786939

[56] A. J. Goodman, D.-H. Lien, G. H. Ahn, L. L. Spiegel, M. Amani, A. P. Willard, A. Javey, W. A. Tisdale, Substrate-dependent exciton diffusion and annihilation in chemically treated MoS_2_ and WS_2_. J. Phys. Chem. C 124, 12175–12184 (2020).

[57] L. Yuan, T. Wang, T. Zhu, M. Zhou, L. Huang, Exciton dynamics, transport, and annihilation in atomically thin two-dimensional semiconductors. J. Phys. Chem. Lett. 8, 3371–3379 (2017). 10.1021/acs.jpclett.7b00885 28661147

[58] J. P. Philbin, E. Rabani, Electron–hole correlations govern Auger recombination in nanostructures. Nano Lett. 18, 7889–7895 (2018). 10.1021/acs.nanolett.8b03715 30403875

[59] Y. Tamai, Y. Matsuura, H. Ohkita, H. Benten, S. Ito, One-dimensional singlet exciton diffusion in poly(3-hexylthiophene) crystalline domains. J. Phys. Chem. Lett. 5, 399–403 (2014). 10.1021/jz402299a 26270718

[60] A. Srivastava, J. Kono, Diffusion-limited exciton-exciton annihilation in single-walled carbon nanotubes: A time-dependent analysis. Phys. Rev. B 79, 205407 (2009).

[61] M. A. Stevens, C. Silva, D. M. Russell, R. H. Friend, Exciton dissociation mechanisms in the polymeric semiconductors poly(9,9-dioctylfluorene) and poly(9,9-dioctylfluorene-co-benzothiadiazole). Phys. Rev. B 63, 165213 (2001).

[62] S. Kumar, I. S. Dunn, S. Deng, T. Zhu, Q. Zhao, O. F. Williams, R. Tempelaar, L. Huang, Exciton annihilation in molecular aggregates suppressed through quantum interference. Nat. Chem. 15, 1118–1126 (2023). 10.1038/s41557-023-01233-x 37337112

[63] Y. Lee, J. D. S. Forte, A. Chaves, A. Kumar, T. T. Tran, Y. Kim, S. Roy, T. Taniguchi, K. Watanabe, A. Chernikov, J. I. Jang, T. Low, J. Kim, Boosting quantum yields in two-dimensional semiconductors via proximal metal plates. Nat. Commun. 12, 7095 (2021). 10.1038/s41467-021-27418-x PMC8651657 34876573

[64] Z. Li, D. F. Cordovilla Leon, W. Lee, K. Datta, Z. Lyu, J. Hou, T. Taniguchi, K. Watanabe, E. Kioupakis, P. B. Deotare, Dielectric engineering for manipulating exciton transport in semiconductor monolayers. Nano Lett. 21, 8409–8417 (2021). 10.1021/acs.nanolett.1c02990 34591493

[65] Q. Wei, Q. Yao, Y. Han, Z. Wang, Y. Shu, S. Liu, Z. Wang, X. Bai, Z. Meng, Y. Gao, W. Hu, S. Zhang, E. Shi, L. Yuan, Lattice control of exciton landscapes enhances energy transport in 2D perovskites. Laser Photon. Rev. 20, e03273 (2026).

[66] L. Jin, C. Mora Perez, Y. Gao, K. Ma, J. Y. Park, S. Li, P. Guo, L. Dou, O. Prezhdo, L. Huang, Superior phonon-limited exciton mobility in lead-free two-dimensional perovskites. Nano Lett. 24, 3638–3646 (2024). 10.1021/acs.nanolett.3c04895 38498912

[67] M. Seitz, M. Meléndez, N. Alcázar-Cano, D. N. Congreve, R. Delgado-Buscalioni, F. Prins, Mapping the trap-state landscape in 2D metal-halide perovskites using transient photoluminescence microscopy. Adv. Opt. Mater. 9, 2001875 (2021).

[68] M. Seitz, A. J. Magdaleno, N. Alcázar-Cano, M. Meléndez, T. J. Lubbers, S. W. Walraven, S. Pakdel, E. Prada, R. Delgado-Buscalioni, F. Prins, Exciton diffusion in two-dimensional metal-halide perovskites. Nat. Commun. 11, 2035 (2020). 10.1038/s41467-020-15882-w PMC7184754 32341361

[69] K. R. Hansen, C. E. McClure, D. Powell, H. Hsieh, L. Flannery, K. Garden, E. J. Miller, D. J. King, S. Sainio, D. Nordlund, J. S. Colton, L. Whittaker-Brooks, Low exciton binding energies and localized exciton–polaron states in 2D tin halide perovskites. Adv. Opt. Mater. 10, 2102698 (2022).

[70] K. R. Hansen, C. Y. Wong, C. E. McClure, B. Romrell, L. Flannery, D. Powell, K. Garden, A. Berzansky, M. Eggleston, D. J. King, C. M. Shirley, M. C. Beard, W. Nie, A. Schleife, J. S. Colton, L. Whittaker-Brooks, Mechanistic origins of excitonic properties in 2D perovskites: Implications for exciton engineering. Matter 6, 3463–3482 (2023).

[71] H. Ouhbi, F. Ambrosio, F. De Angelis, J. Wiktor, Strong electron localization in tin halide perovskites. J. Phys. Chem. Lett. 12, 5339–5343 (2021). 10.1021/acs.jpclett.1c01326 PMC8280731 34062062

[72] G. Laurita, D. H. Fabini, C. C. Stoumpos, M. G. Kanatzidis, R. Seshadri, Chemical tuning of dynamic cation off-centering in the cubic phases of hybrid tin and lead halide perovskites. Chem. Sci. 8, 5628–5635 (2017). 10.1039/c7sc01429e PMC5621007 28989600

[73] X. Jiang, J. Gu, S. Zheng, T. Zhang, X. Li, Y. Guan, C. Li, J. Wang, W. Zhang, Y. Fu, Mixed-solvent crystallization expands the chemical space of 2D perovskites and enables phonon-limited exciton transport. J. Am. Chem. Soc. 147, 32287–32298 (2025). 10.1021/jacs.5c13393 40845225

[74] H. K. Mao, J. Xu, P. M. Bell, Calibration of the ruby pressure gauge to 800 kbar under quasi-hydrostatic conditions. J. Geophys. Res. Solid Earth 91, 4673–4676 (1986).

[75] G. Kresse, J. Furthmüller, Efficient iterative schemes for ab initio total-energy calculations using a plane-wave basis set. Phys. Rev. B 54, 11169–11186 (1996). 10.1103/physrevb.54.11169 9984901

[76] P. E. Blöchl, Projector augmented-wave method. Phys. Rev. B 50, 17953–17979 (1994). 10.1103/physrevb.50.17953 9976227

[77] J. P. Perdew, K. Burke, M. Ernzerhof, Generalized gradient approximation made simple. Phys. Rev. Lett. 77, 3865–3868 (1996). 10.1103/PhysRevLett.77.3865 10062328

[78] S. Grimme, J. Antony, S. Ehrlich, H. Krieg, A consistent and accurate ab initio parametrization of density functional dispersion correction (DFT-D) for the 94 elements H-Pu. J. Chem. Phys. 132, 154104 (2010). 10.1063/1.3382344 20423165

[79] W. Reckien, F. Janetzko, M. F. Peintinger, T. Bredow, Implementation of empirical dispersion corrections to density functional theory for periodic systems. J. Comput. Chem. 33, 2023–2031 (2012). 10.1002/jcc.23037 22684689

[80] V. Wang, N. Xu, J.-C. Liu, G. Tang, W.-T. Geng, VASPKIT: A user-friendly interface facilitating high-throughput computing and analysis using VASP code. Comput. Phys. Commun. 267, 108033 (2021).

[81] M. Gajdoš, K. Hummer, G. Kresse, J. Furthmüller, F. Bechstedt, Linear optical properties in the projector-augmented wave methodology. Phys. Rev. B 73, 045112 (2006).

[82] S. Baroni, R. Resta, Ab initio calculation of the macroscopic dielectric constant in silicon. Phys. Rev. B 33, 7017–7021 (1986). 10.1103/physrevb.33.7017 9938028

[83] T. Lu, F. Chen, Multiwfn: A multifunctional wavefunction analyzer. J. Comput. Chem. 33, 580–592 (2012). 10.1002/jcc.22885 22162017

[84] T. Lu, A comprehensive electron wavefunction analysis toolbox for chemists, Multiwfn. J. Chem. Phys. 161, 082503 (2024). 10.1063/5.0216272 39189657

[85] T. Lu, Q. Chen, Independent gradient model based on Hirshfeld partition: A new method for visual study of interactions in chemical systems. J. Comput. Chem. 43, 539–555 (2022). 10.1002/jcc.26812 35108407

[86] T. Lu, F. Chen, Quantitative analysis of molecular surface based on improved Marching Tetrahedra algorithm. J. Mol. Graph. Model. 38, 314–323 (2012). 10.1016/j.jmgm.2012.07.004 23085170

[87] T. D. Kühne, M. Iannuzzi, M. Del Ben, V. V. Rybkin, P. Seewald, F. Stein, T. Laino, R. Z. Khaliullin, O. Schütt, F. Schiffmann, D. Golze, J. Wilhelm, S. Chulkov, M. H. Bani-Hashemian, V. Weber, U. Borštnik, M. Taillefumier, A. S. Jakobovits, A. Lazzaro, H. Pabst, T. Müller, R. Schade, M. Guidon, S. Andermatt, N. Holmberg, G. K. Schenter, A. Hehn, A. Bussy, F. Belleflamme, G. Tabacchi, A. Glöß, M. Lass, I. Bethune, C. J. Mundy, C. Plessl, M. Watkins, J. VandeVondele, M. Krack, J. Hutter, CP2K: An electronic structure and molecular dynamics software package—Quickstep: Efficient and accurate electronic structure calculations. J. Chem. Phys. 152, 194103 (2020). 10.1063/5.0007045 33687235

[88] C. Hartwigsen, S. Goedecker, J. Hutter, Relativistic separable dual-space Gaussian pseudopotentials from H to Rn. Phys. Rev. B 58, 3641–3662 (1998). 10.1103/physrevb.54.1703 9986014

[89] J. VandeVondele, J. Hutter, Gaussian basis sets for accurate calculations on molecular systems in gas and condensed phases. J. Chem. Phys. 127, 114105 (2007). 10.1063/1.2770708 17887826

[90] M. A. Spackman, D. Jayatilaka, Hirshfeld surface analysis. CrstEngComm 11, 19–32 (2009).

