# Supplementary material for: Unlock lasing in two-dimensional metal halide perovskites by tuning ns2 lone pairs
Source: Sci Adv. 2026 Aug 5;12(32):eaee5631. doi: 10.1126/sciadv.aee5631 (PMC13440396; doi:10.1126/sciadv.aee5631)
Supplement: Supplementary file 1 [file sciadv.aee5631_sm.pdf]

Science Advances
AAAS

Supplementary Materials for

Unlock lasing in two-dimensional metal halide perovskites by tuning ns^2 lone pairs

Xinyu Li *et al.*

Corresponding author: Yongping Fu, yfu@pku.edu.cn

Sci. Adv. **12**, eaee5631 (2026)
DOI: 10.1126/sciadv.aee5631

The PDF file includes:

Figs. S1 to S31
Tables S1 to S10
Legend for data S1

Other Supplementary Material for this manuscript includes the following:

Data S1

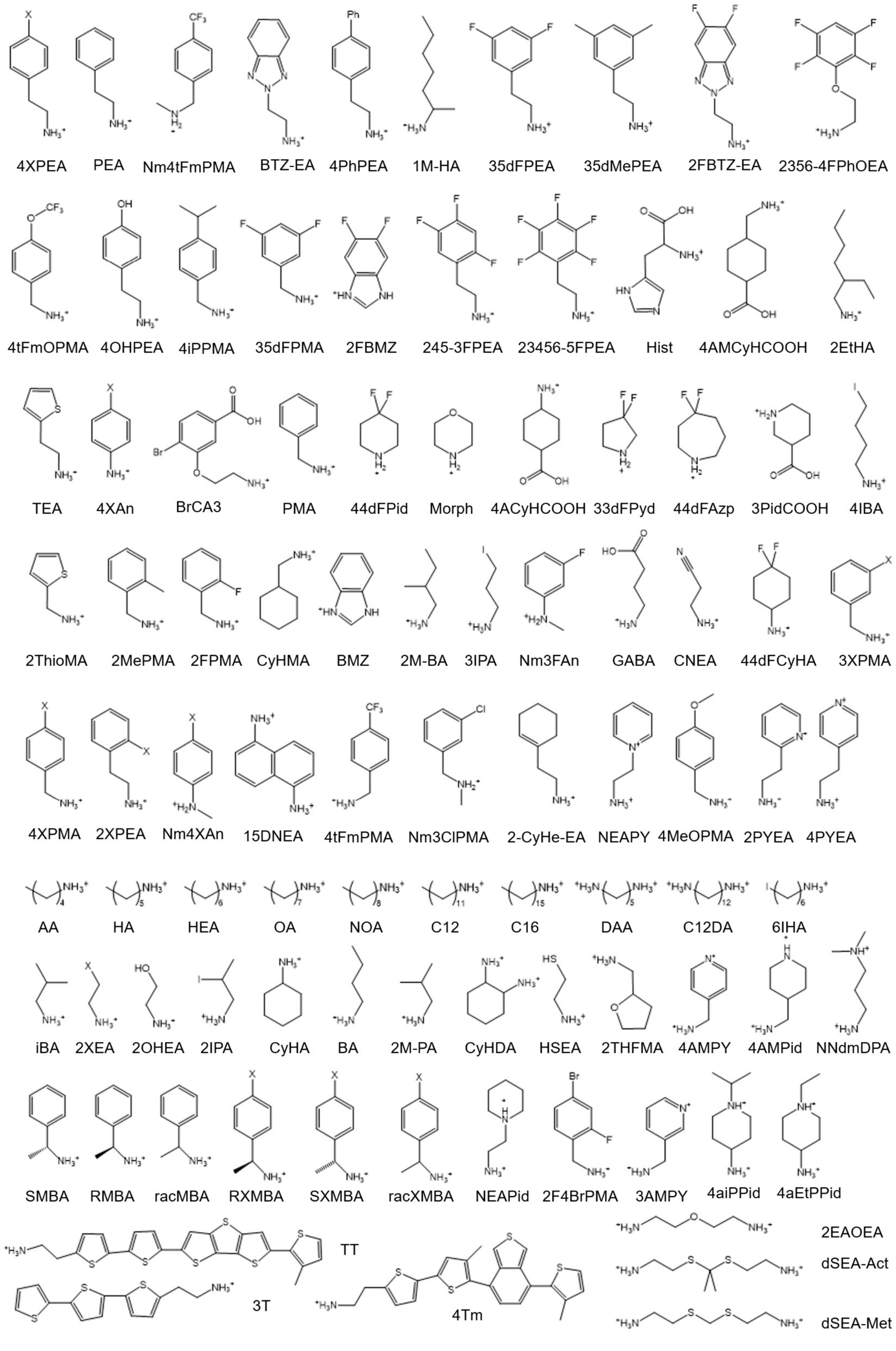

Fig. S1. Molecular structures of all LA-cations in the surveyed 2D perovskites. The abbreviations are consistent with those in Table S1.

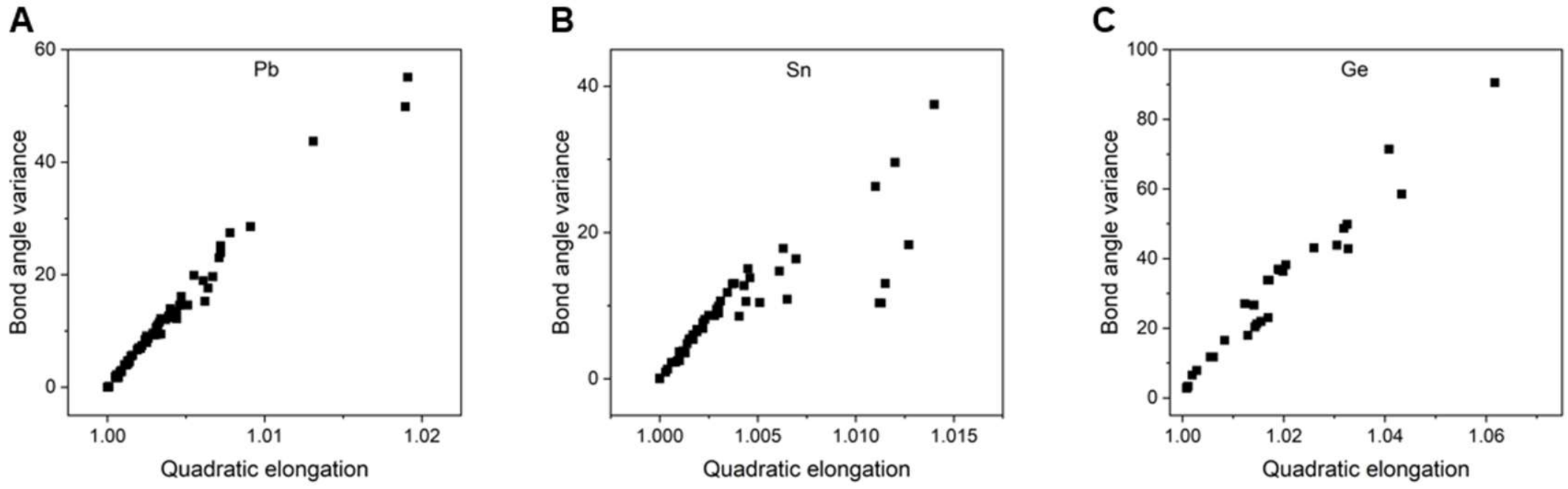

Fig. S2. Structural map of bond angle variance ($\boldsymbol{\sigma_\theta^2}$) and quadratic elongation ($\langle\boldsymbol{\lambda}\rangle$) for 2D **(A)** Pb-, **(B)** Sn-, and **(C)** Ge-based iodide perovskites, revealing strong correlation between $\boldsymbol{\sigma_\theta^2}$ and $\langle\boldsymbol{\lambda}\rangle$.

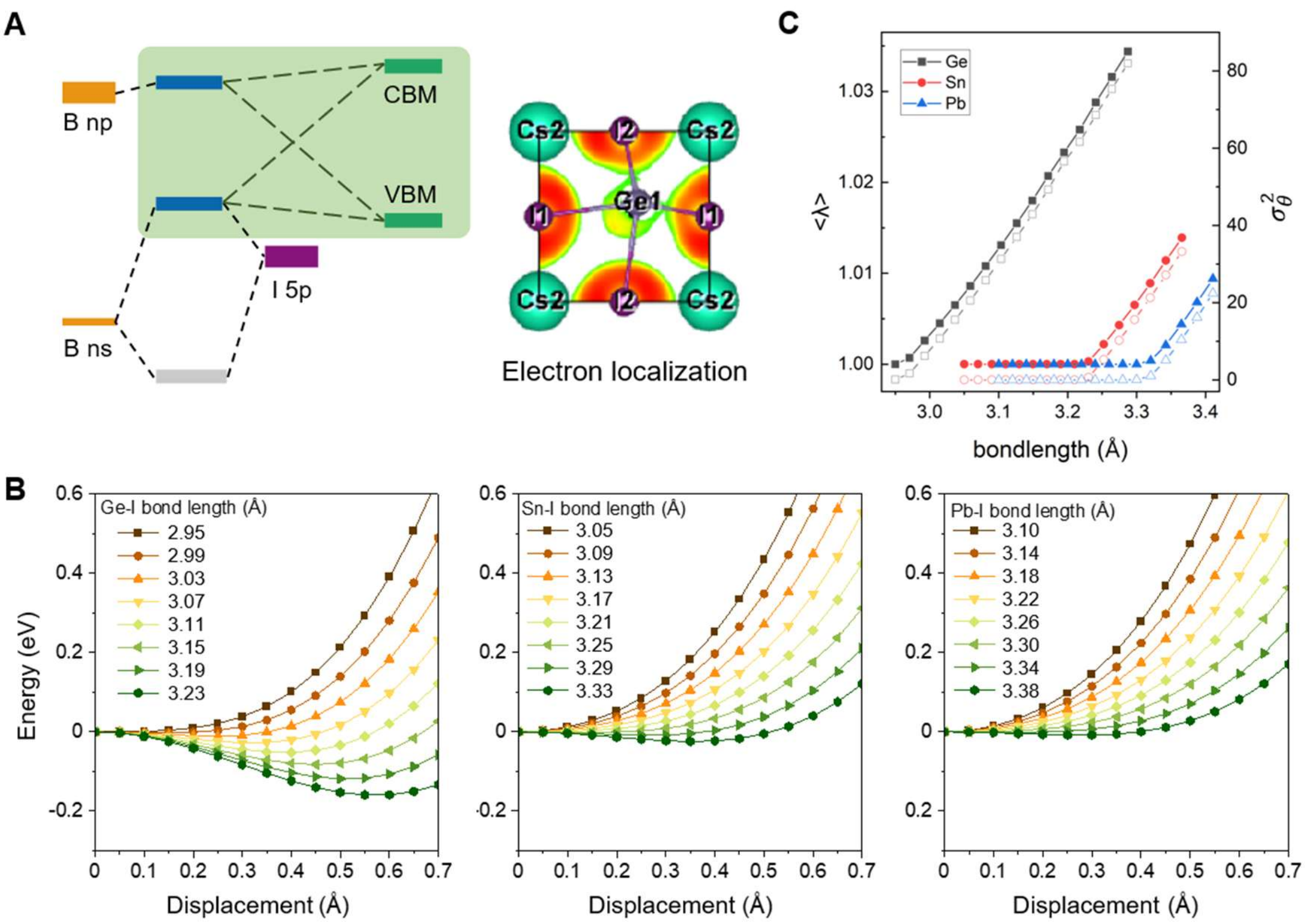

Fig. S3. Lone pair activity. **(A)** Schematic illustration of the orbital interactions underlying lone pair activity, i.e., a second-order Jahn-Teller distortion, driven by hybridization between the B-cation's *s*-orbital (contributing to the valence band maximum, VBM) and *p*-orbital (contributing to the conduction band minimum, CBM), mediated by iodide *p*-orbital. Electron localization function (ELF) of the model structure Cs_2GeI_4, showing lone pair localization opposite to the direction of Ge^{2+} displacement. **(B)** Calculated potential energy surface (PES) for B-cation displacement in model structures Cs_2BI_4 with different B-I bond lengths. B = Ge^{2+}, Sn^{2+}, and Pb^{2+}. **(C)** Structure distortion map showing intra-octahedral distortion parameters, $\langle\boldsymbol{\lambda}\rangle$ and $\boldsymbol{\sigma_\theta^2}$, as function of $\overline{\boldsymbol{d}}$ for Cs_2BI_4 model structures optimized at the global minima of their respective PES (from **B**). Filled symbols represent $\langle\boldsymbol{\lambda}\rangle$, and hollow symbols represent $\boldsymbol{\sigma_\theta^2}$.

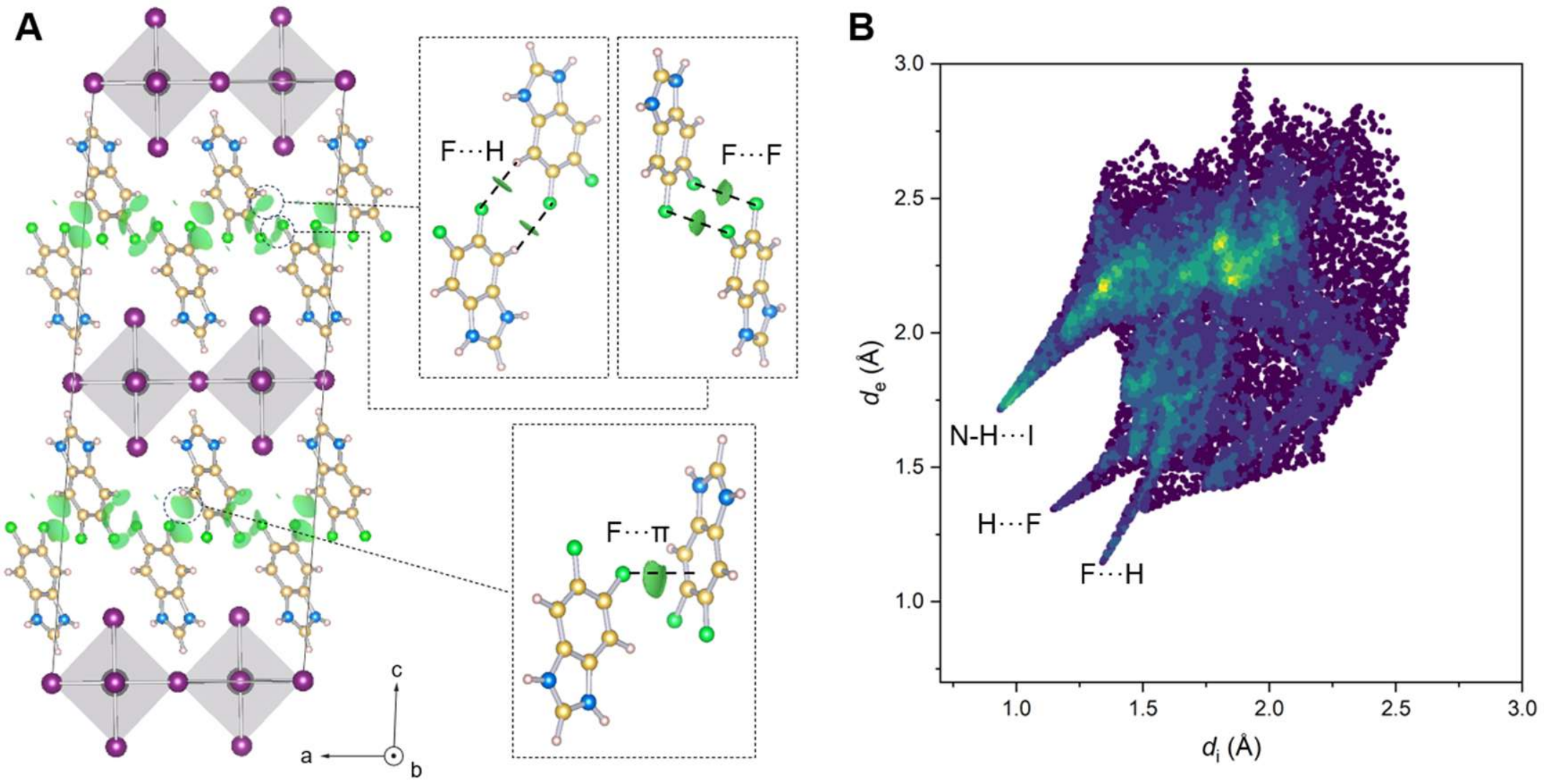

Fig. S4. Intermolecular forces in $(2FBMZ)_2PbI_4$. **(A)** Intermolecular forces in the organic spacer layers of $(2FBMZ)_2PbI_4$, shown as green regions representing the independent gradient model based on Hirshfeld partition (IGMH) isosurfaces at $\delta g^{inter} = 0.005$ a.u. The observed intermolecular interactions including F···π halogen bonding, F··· H hydrogen bonding, and F···F dispersion force. **(B)** Hirshfeld surface fingerprint plots of $(2FBMZ)_2PbI_4$, highlighting the intermolecular interactions. The *x*-axis (d_i) and the *y*-axis (d_e) represent the distances from a point on the Hirshfeld surface to the nearest nucleus internal and external to the surface, respectively. Each plot is color-mapped according to point density using a viridis color scale, with low-density regions appearing purple and high-density regions appearing yellow.

Type or paste caption here. Create a page break and paste in the Figure above the caption.

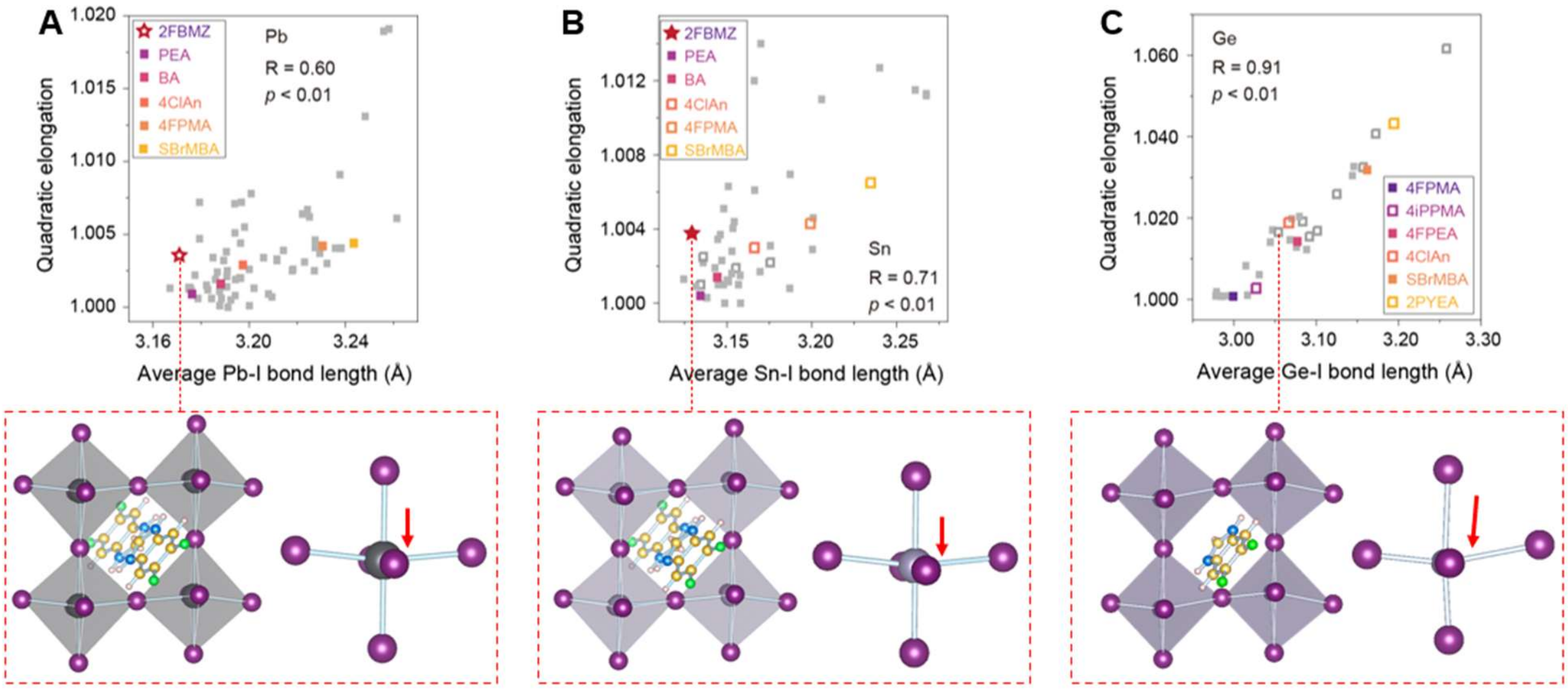

Fig. S5. Comparison of the crystal structures of $(2FBMZ)_2BI_4$ with different B cations. Structural correlation map of quadratic elongation ($\langle \boldsymbol{\lambda} \rangle$) versus average bond length ($\bar{\boldsymbol{d}}$) for $(LA)_2BI_4$ structures with B = **(A)** Pb, **(B)** Sn, and **(C)** Ge. The crystal structures of $(2FBMZ)_2BI_4$ are shown in red dashed box. Both $(2FBMZ)_2PbI_4$ and $(2FBMZ)_2SnI_4$ appear at similar locations in their respective maps, and feature a short average B–I bond length together with a large octahedral distortion. By contrast, $(2FBMZ)_2GeI_4$ falls in a distinct region, indicating a different structural response to the same spacer. This contrast suggests that the structural role of 2FBMZ is modulated by the metal cation, likely because the smaller Ge^{2+} alters the size of the half-perovskite cavity accommodating the spacer and gives rise to a different potential energy landscape.

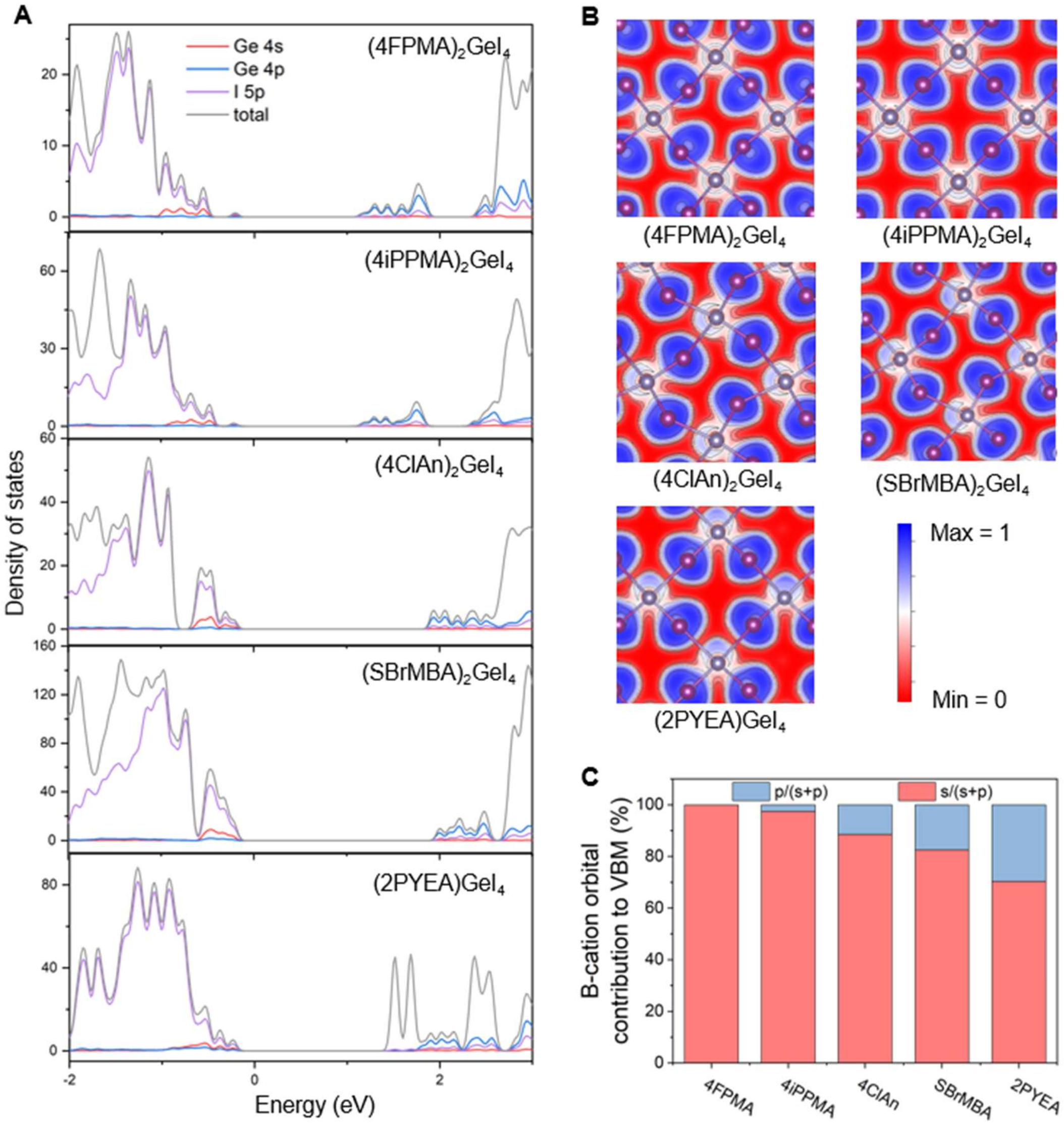

Fig. S6. Lone pair activity in Ge-based structures with different LA-cations. **(A)** Orbital-projected density of states (PDOS), **(B)** ELF, and **(C)** relative percentage orbital contribution of B-cation *s* and *p* orbitals to the VBM for $(4FPMA)_2GeI_4$, $(4iPPMA)_2GeI_4$, $(4ClAn)_2GeI_4$, $(SBrMBA)_2GeI_4$, and $(2PYEA)GeI_4$, showing increasing *sp* hybridization with increasing average Ge-I bond length.

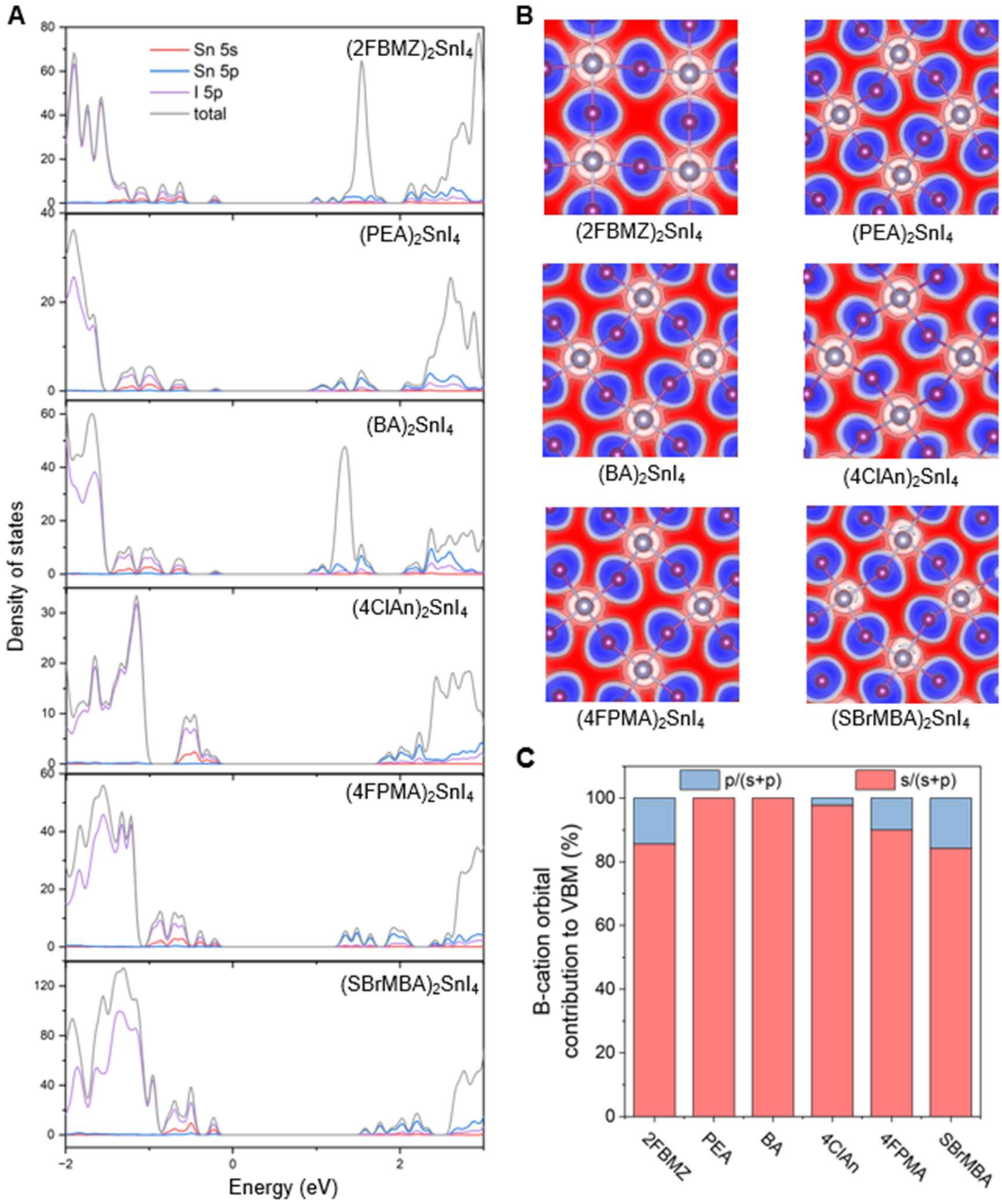

Fig. S7. Lone pair activity in Sn-based structures with different LA-cations. **(A)** PDOS, **(B)** ELF, and **(C)** relative percentage orbital contribution of B-cation *s* and *p* orbitals to the VBM for $(2FBMZ)_2SnI_4$, $(PEA)_2SnI_4$, $(BA)_2SnI_4$, $(4ClAn)_2SnI_4$, $(4FPMA)_2SnI_4$, and $(SBrMBA)_2SnI_4$.

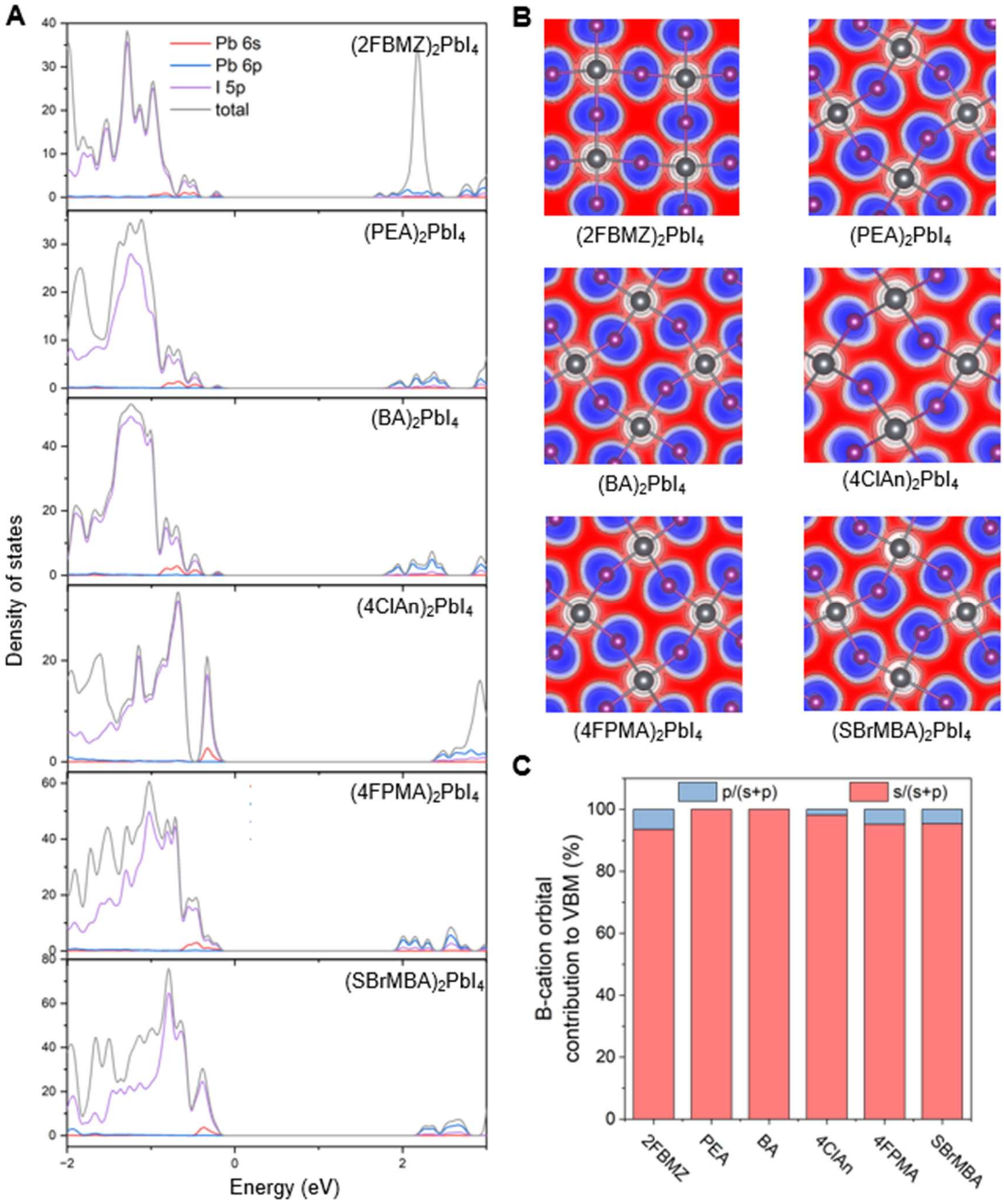

Fig. S8. Lone pair activity in Pb-based structures with different LA-cations. **(A)** PDOS, **(B)** ELF, and **(C)** relative percentage orbital contribution of B-cation *s* and *p* orbitals to the VBM for $(2FBMZ)_2PbI_4$, $(PEA)_2PbI_4$, $(BA)_2PbI_4$, $(4ClAn)_2PbI_4$, $(4FPMA)_2PbI_4$, and $(SBrMBA)_2PbI_4$.

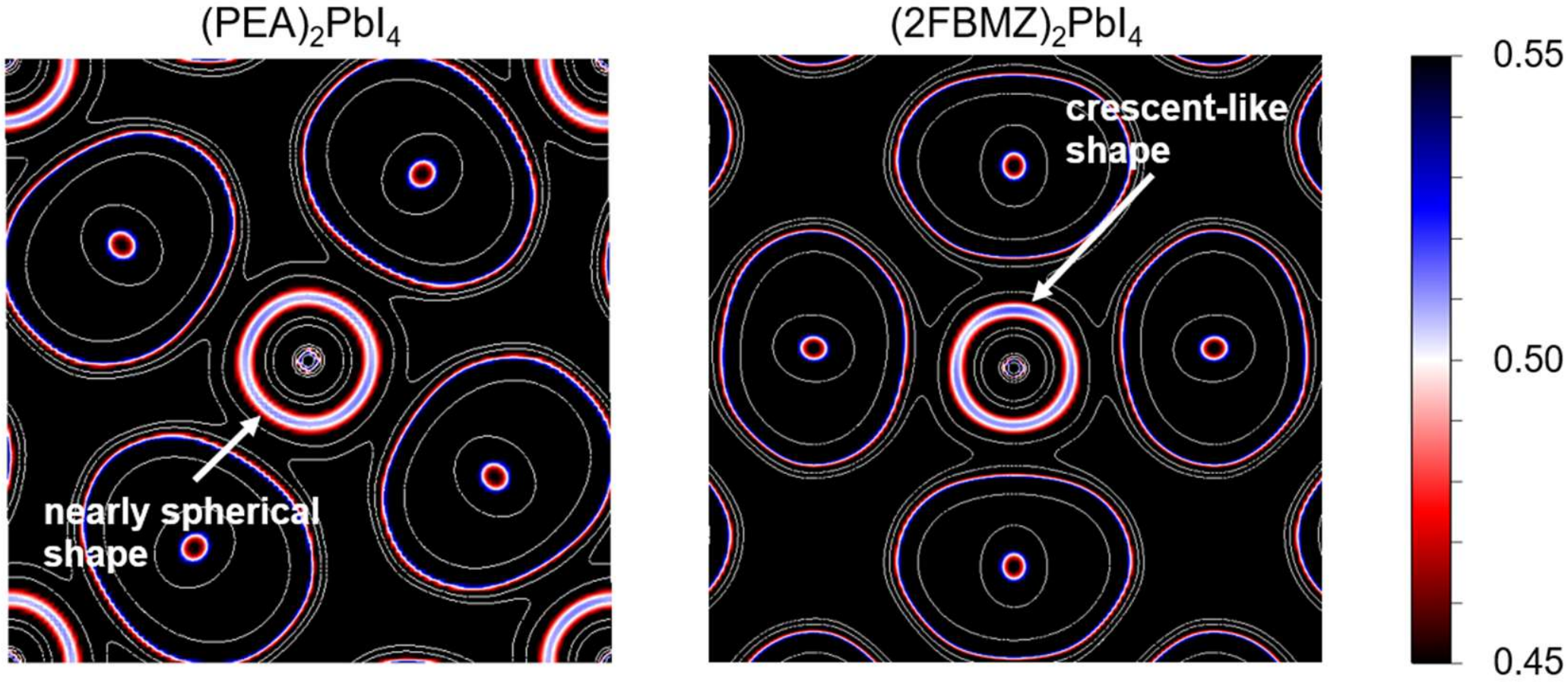

Fig. S9. The ELF maps of $(PEA)_2PbI_4$ and $(2FBMZ)_2PbI_4$ with scare bar of ELF values from 0.45 to 0.55, showing deviation from spherical symmetry of the electron density around the Pb atoms in $(2FBMZ)_2PbI_4$.

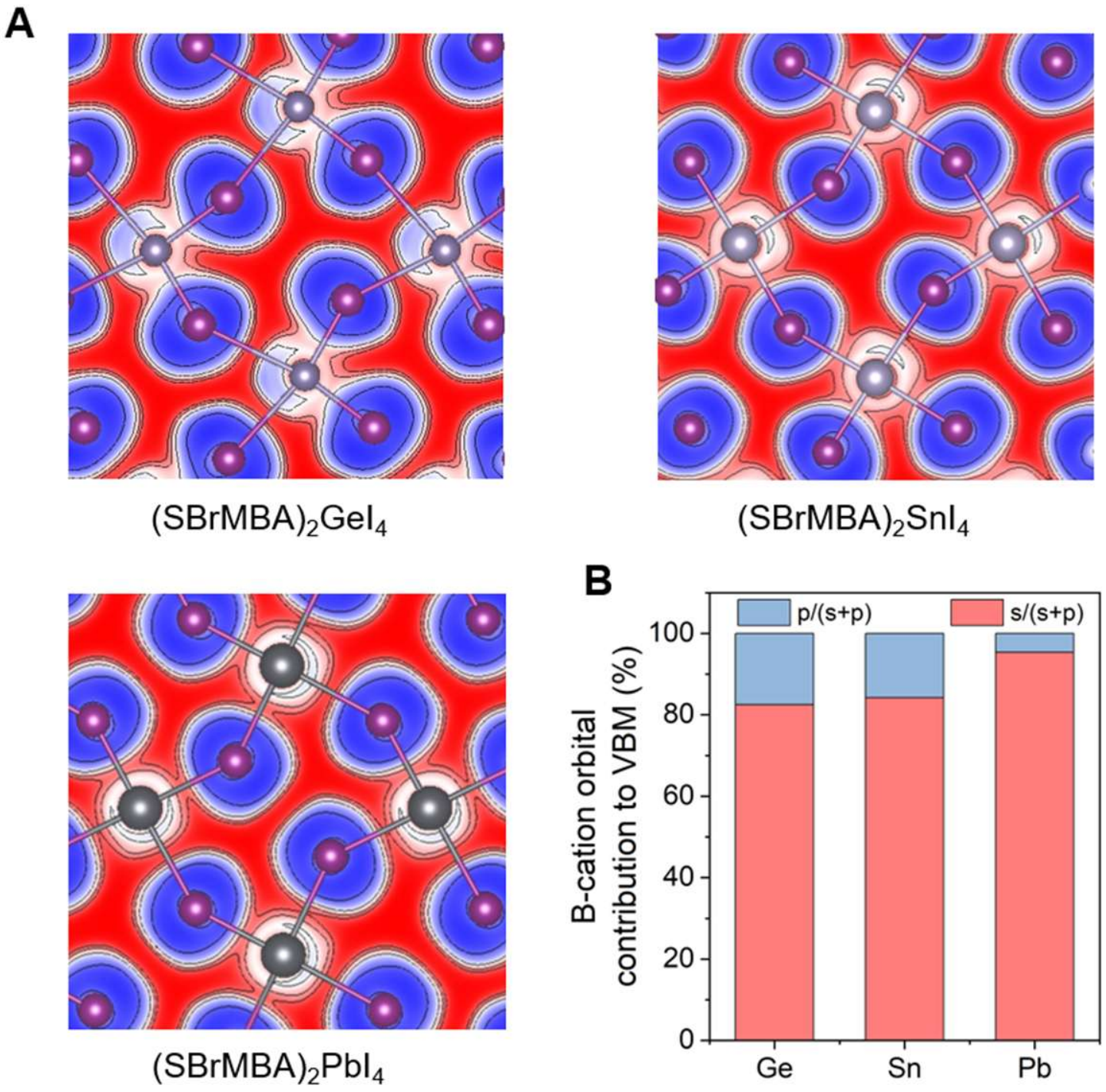

Fig. S10. Lone pair activity in Ge-, Sn-, and Pb-based structures with the same LA-cation. **(A)** ELF and **(B)** relative percentage orbital contribution of B-cation *s* and *p* orbitals to the VBM for $(SBrMBA)_2BI_4$. B = Ge^{2+}, Sn^{2+}, and Pb^{2+}.

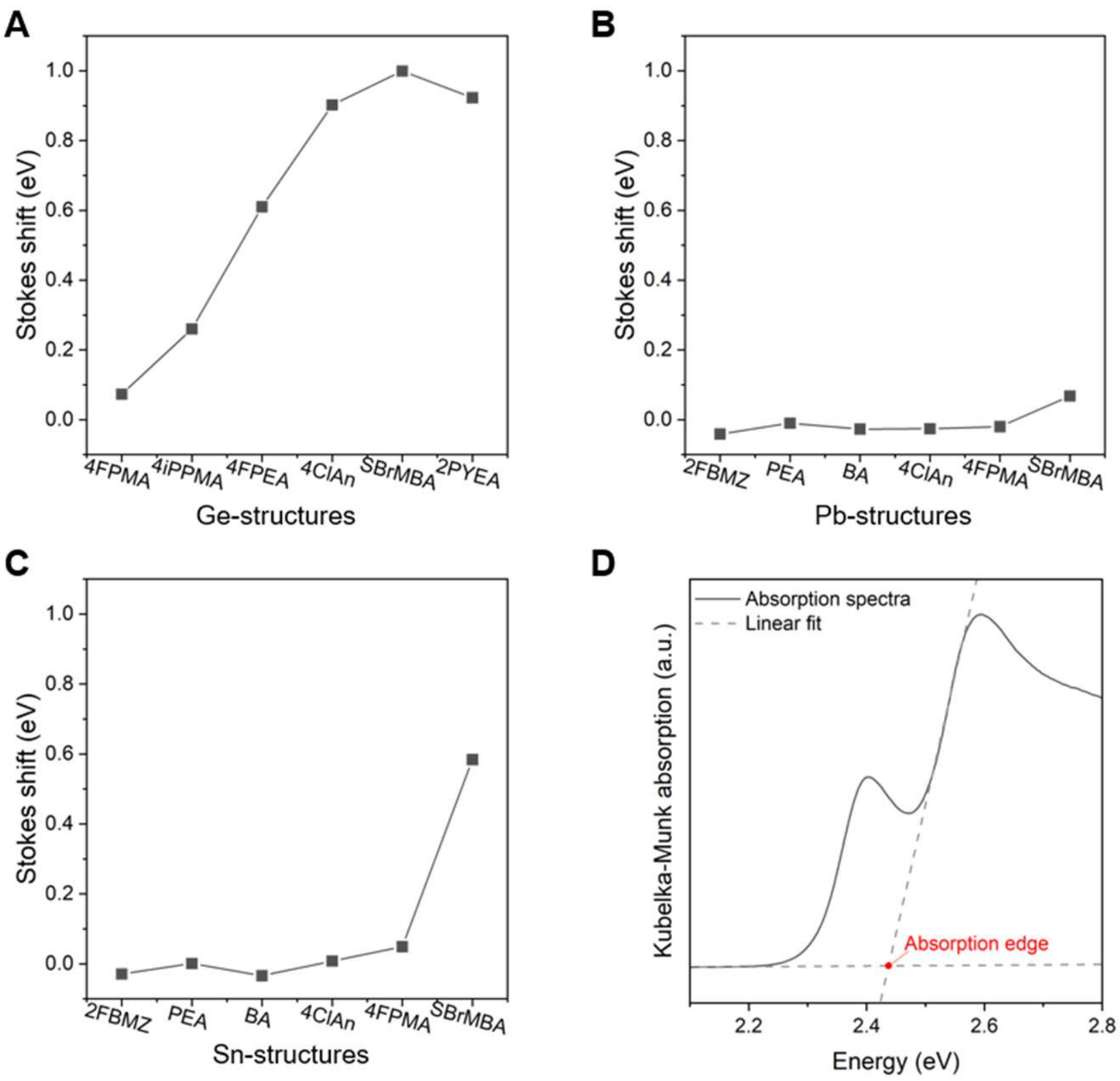

Fig. S11. The Stokes shift of **(A)** Ge-based, **(B)** Pb-based, and **(C)** Sn-based 2D perovskites. The Stokes shift is obtained by subtracting the energy of the emission peak from the energy of the absorption edge, where the definition of absorption edge is shown in **(D)**.

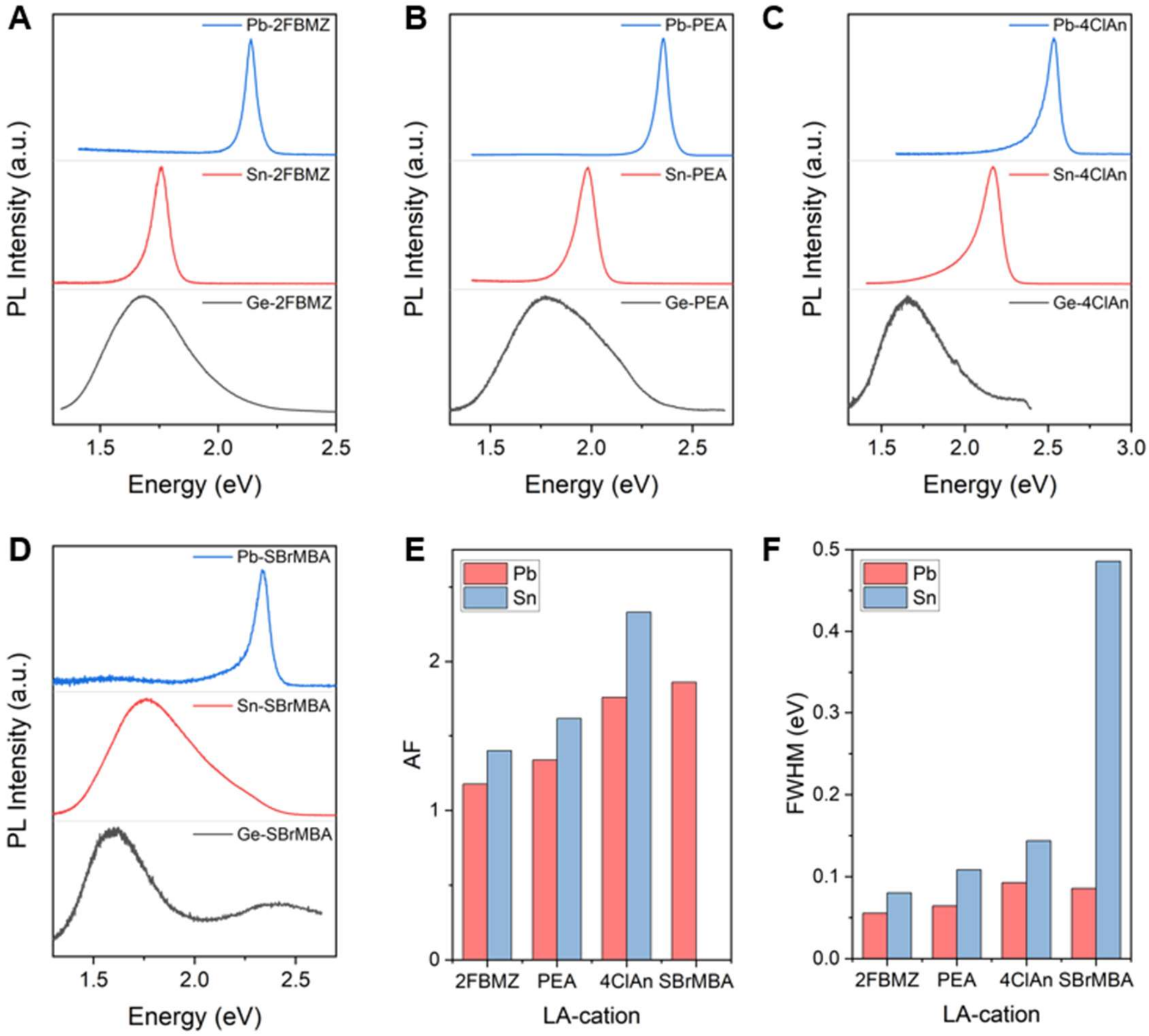

Fig. S12. PL spectra of **(A)** $(2FBMZ)_2BI_4$, **(B)** $(PEA)_2BI_4$, **(C)** $(4ClAn)_2BI_4$, and **(D)** $(SBrMBA)_2BI_4$ at 298 K, where B = Pb, Sn, and Ge. The **(E)** asymmetry factor (AF) and **(F)** full width at half maximum (FWHM) of the PL peaks for various $(LA)_2BI_4$ (B = Pb, Sn) extracted from **(A)**-**(D)**.

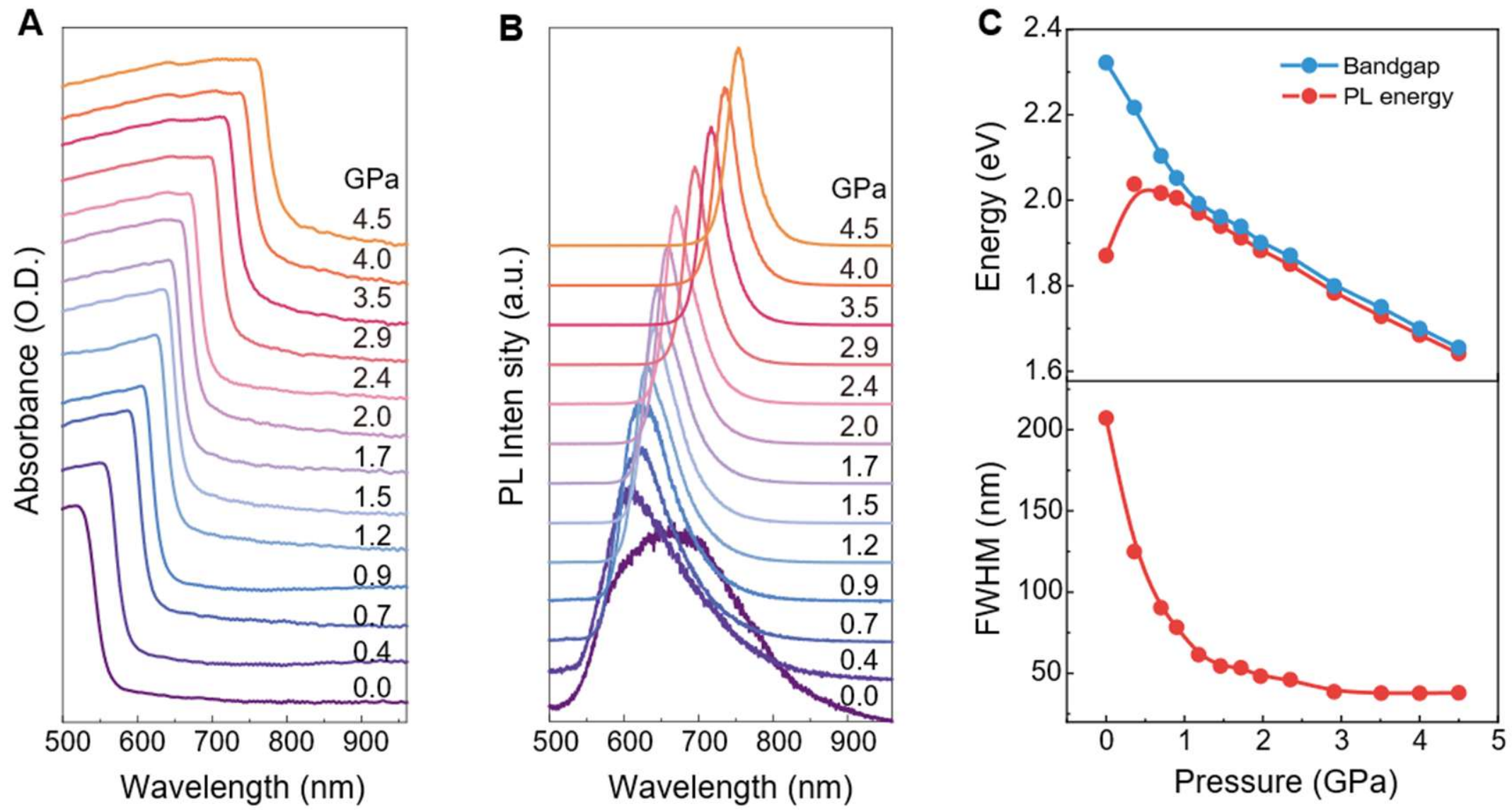

Fig. S13. High-pressure studies on $(4FPEA)_2GeI_4$. Pressure-dependent **(A)** absorption spectra and **(B)** photoluminescence (PL) measured over the pressure range from 0 to 4.5 GPa. **(C)** Evolution of optical bandgap determined from absorption spectra, PL emission peak energy, and FWHM as a function of pressure.

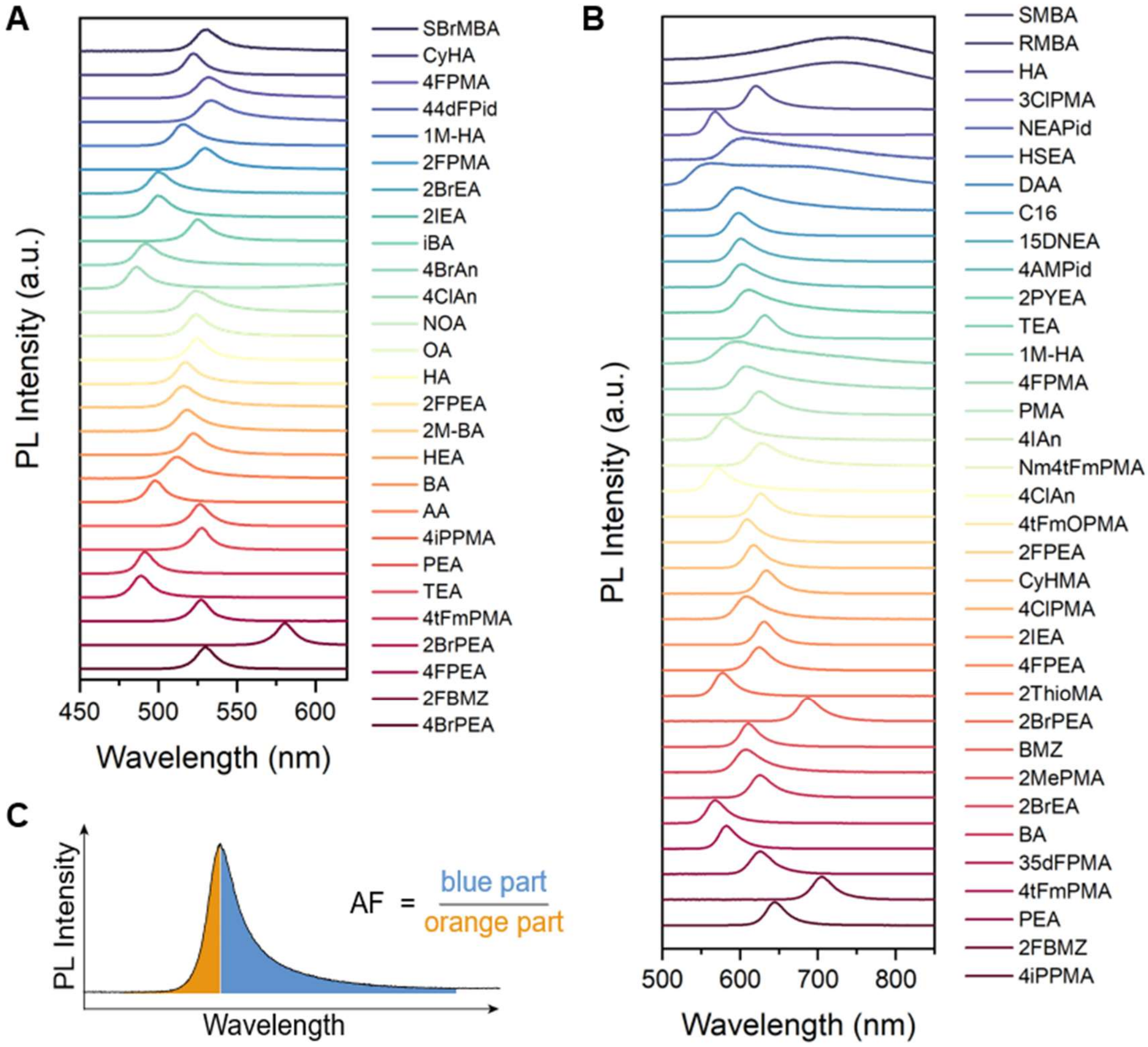

Fig. S14. PL spectra of various 2D perovskites. **(A)** $(LA)_2PbI_4$ and **(B)** $(LA)_2SnI_4$. **(C)** The schematic diagram of asymmetry factor (AF) obtained from the PL spectra.

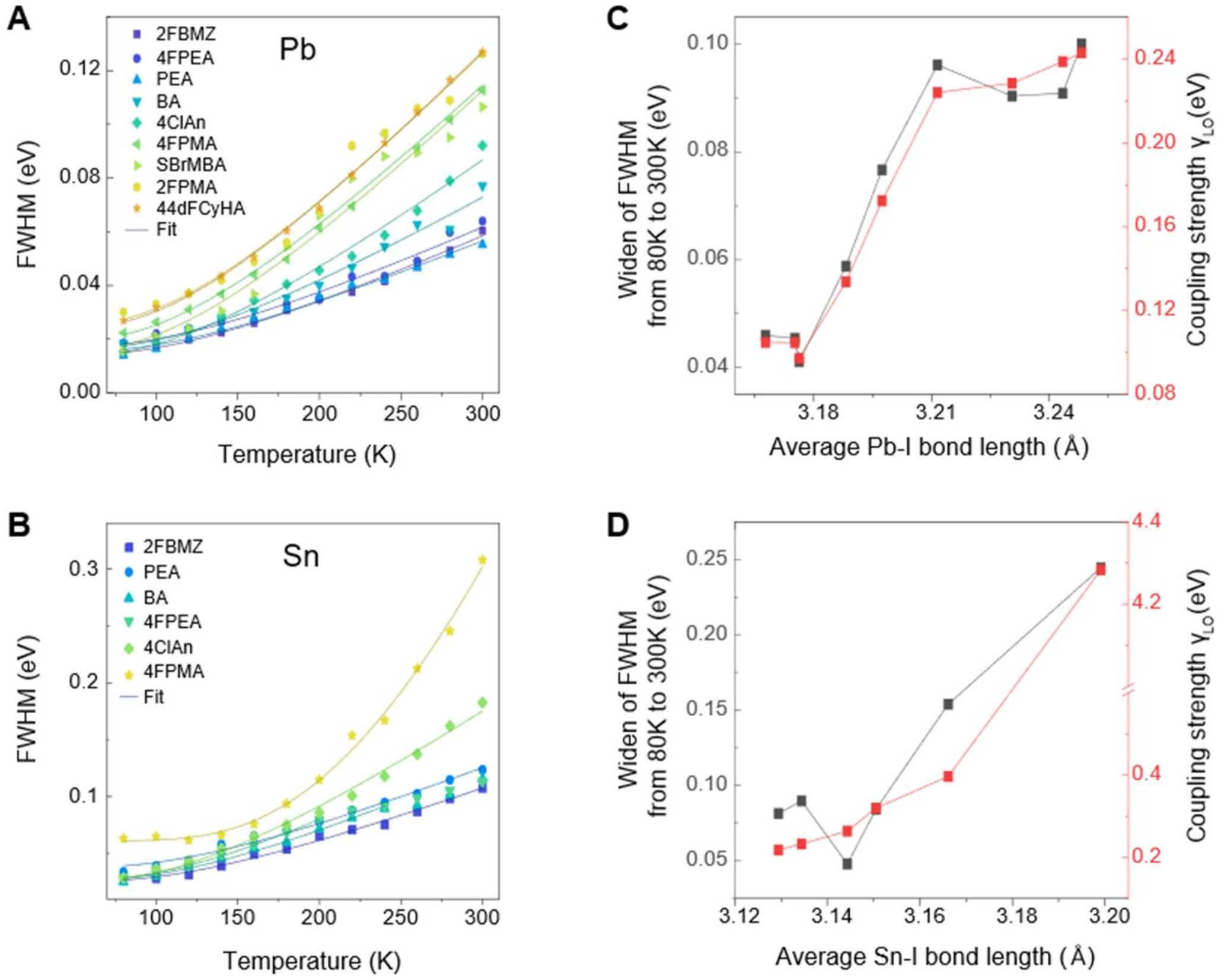

Fig. S15. Electron-phonon coupling parameters of various 2D perovskites. **(A-B)** PL peak width as a function of temperature for various **(A)** Pb-based and **(B)** Sn-based 2D perovskites. The temperature dependence of PL FWHM is fitted by the model: $\Gamma(T) = \Gamma_0 + \Gamma_{LO} = \Gamma_0 + \gamma_{LO}/\left(e^{E_{LO}/k_BT} - 1\right)$, where Γ_0 is inhomogeneous broadening due to structural disorder, and Γ_{LO} is the homogeneous broadening arising from longitudinal optical (LO) phonon scattering, characterized by coupling strength (γ_{LO}) and average phonon energy (E_{LO}). To reliably extract the three unknown parameters, the PL peak width must show a clear exponential increase with temperature, as observed in $(4FPMA)_2SnI_4$. However, for some other structures, this exponential behavior is not prominent. When both γ_{LO} and E_{LO} are treated as free parameters, the fitting can yield unmeaningful results because the two parameters trade off to produce seemingly reasonable fits. Given the similar inorganic frameworks for the same B-cation, we assumed E_{LO} to be similar and use it as a global fitting parameter. Therefore, a global fitting approach was used to extract the γ_{LO} for each structure. All Pb-based perovskites and Sn-based perovskites (except for $(4FPMA)_2SnI_4$, which was fitted individually) were analyzed using this global fit approach. All the FWHM data are listed in Tables S3-S4, and the fitting parameters are listed in Table S5. **(C-D)** The trends of γ_{LO} and the corresponding PL broadening from 80 to 300K as a function of average bond length for **(C)** Pb-based and **(D)** Sn-based 2D perovskites.

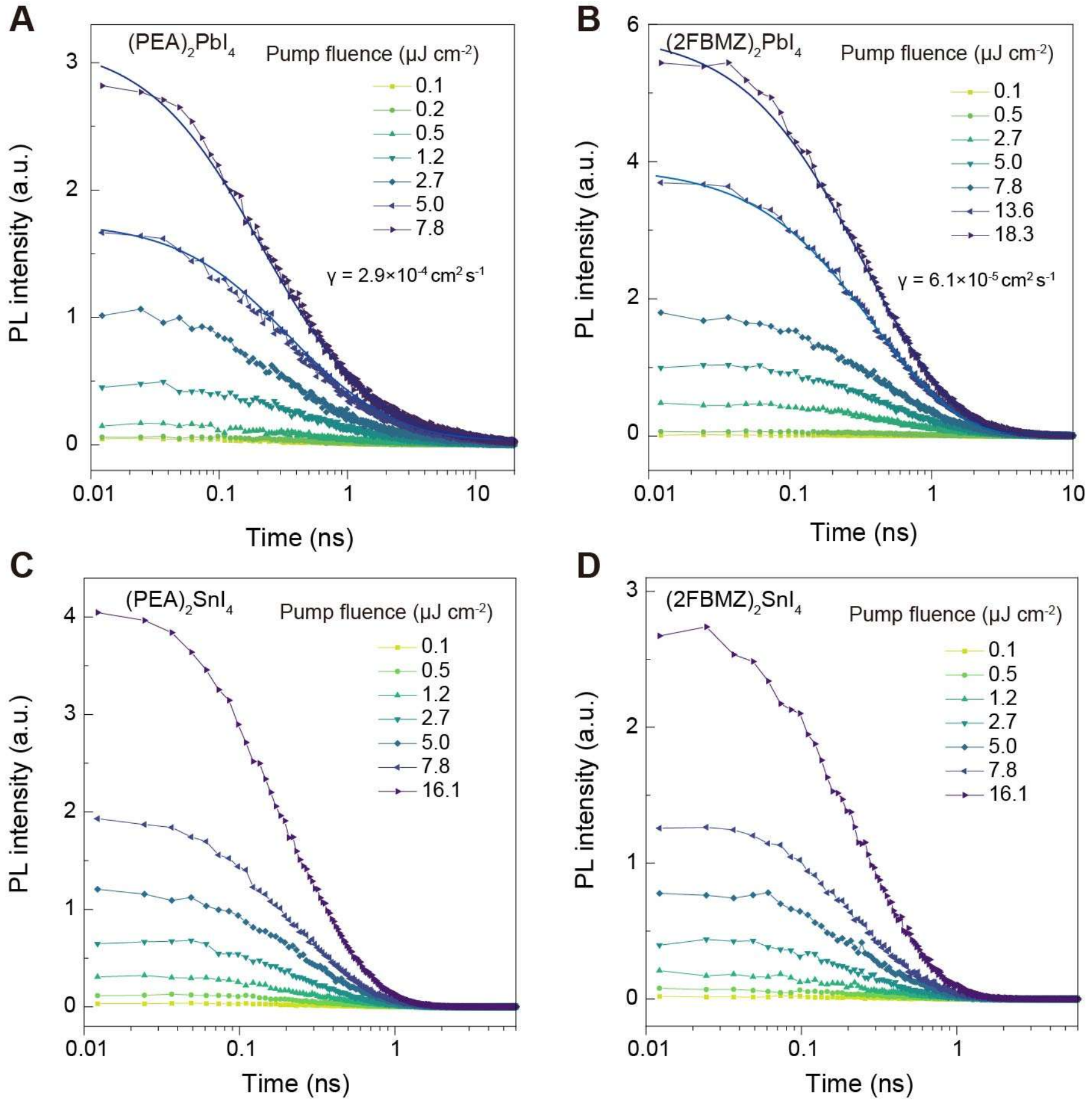

Fig. S16. TRPL spectra with different excitation fluences plotted with PL intensity on a linear scale and time on a logarithmic scale of **(A)** $(PEA)_2PbI_4$, **(B)** $(2FBMZ)_2PbI_4$, **(C)** $(PEA)_2SnI_4$ and **(D)** $(2FBMZ)_2SnI_4$. Exciton recombination kinetics at high pump fluence, fitted using the rate equation $\frac{dn}{dt} = -\frac{n}{\tau} - \gamma n^2$, where τ is the first-order exciton recombination lifetime, and γ is the EEA rate constant. The fitting γ are 2.9×10^{-4} cm^2s^{-1} and 6.1×10^{-5} cm^2s^{-1} for $(PEA)_2PbI_4$ and $(2FBMZ)_2PbI_4$, with corresponding τ values of 27 ns and 1.0 ns. These data correspond to those presented in Fig. 3A.

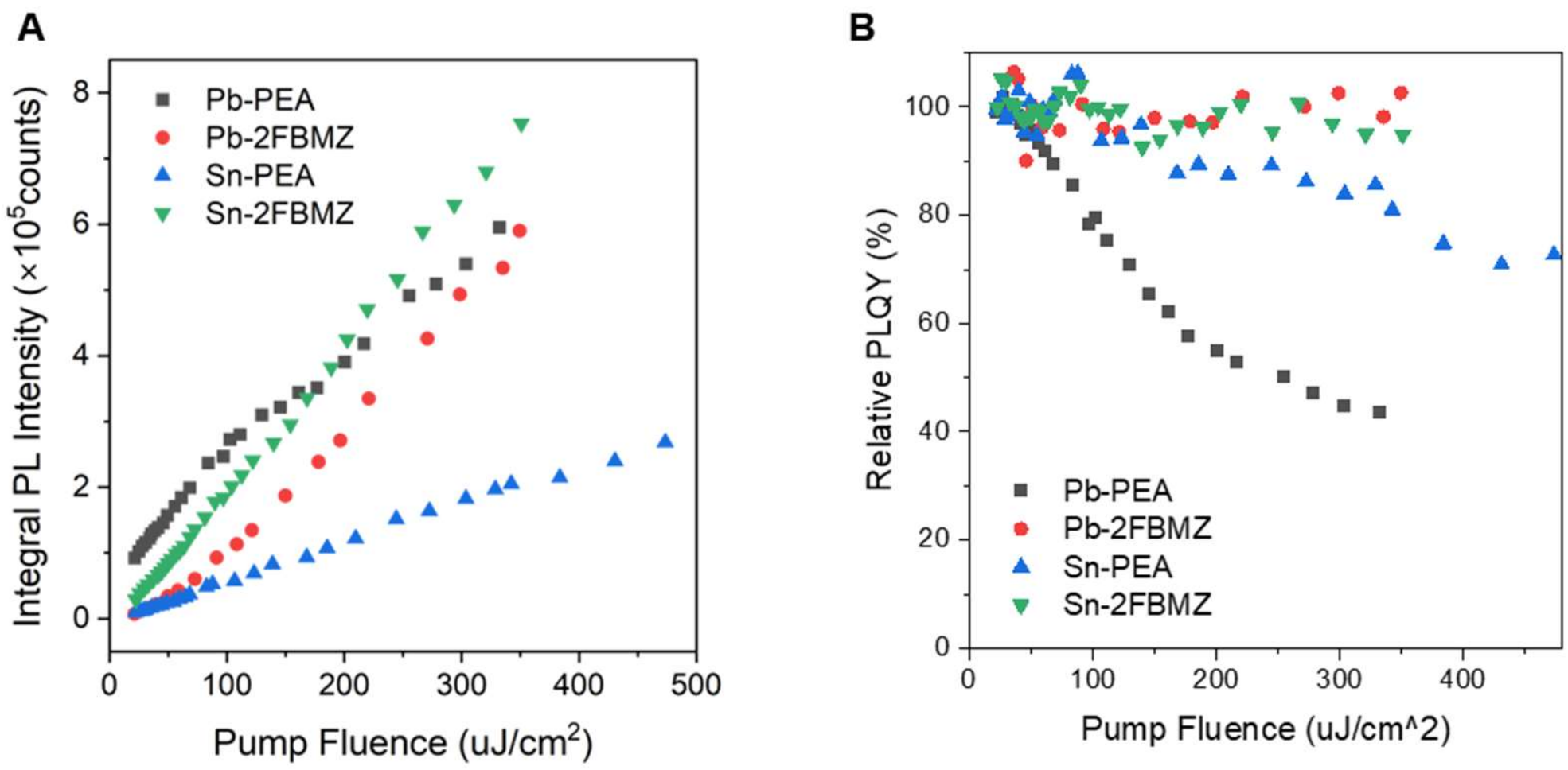

Fig. S17. Pump fluence-dependent PL intensity of $(LA)_2BI_4$ (LA = $2FBMZ^+$, PEA^+, B = Pb^{2+} and Sn^{2+}) under femtosecond laser excitation. **(A)** Integrated PL intensity as a function of pump fluence. Similar to Fig. 3B (measured under picosecond laser excitation), $(PEA)_2PbI_4$ exhibits a linear increase in integrated PL at low fluences, transitioning to a sublinear regime at high fluences (> 100 µJ cm^{-2}). Measurements were performed on single crystals at 80 K using a femtosecond laser excitation (400 nm, 200 Hz) with a spot diameter of 60 µm. The pulsed excitation was generated by an optical parametric amplifier (ORPHEUS-HP), seeded by a femtosecond laser (PHAROS-PH2-20W, 1030 nm, 290 fs, 200 kHz). The PL spectra were recorded to calculate the integrated PL intensity. **(B)** Relative PL quantum yield (PLQY) as function of pump fluence, showing a decrease in PLQY for $(PEA)_2PbI_4$ at high fluences.

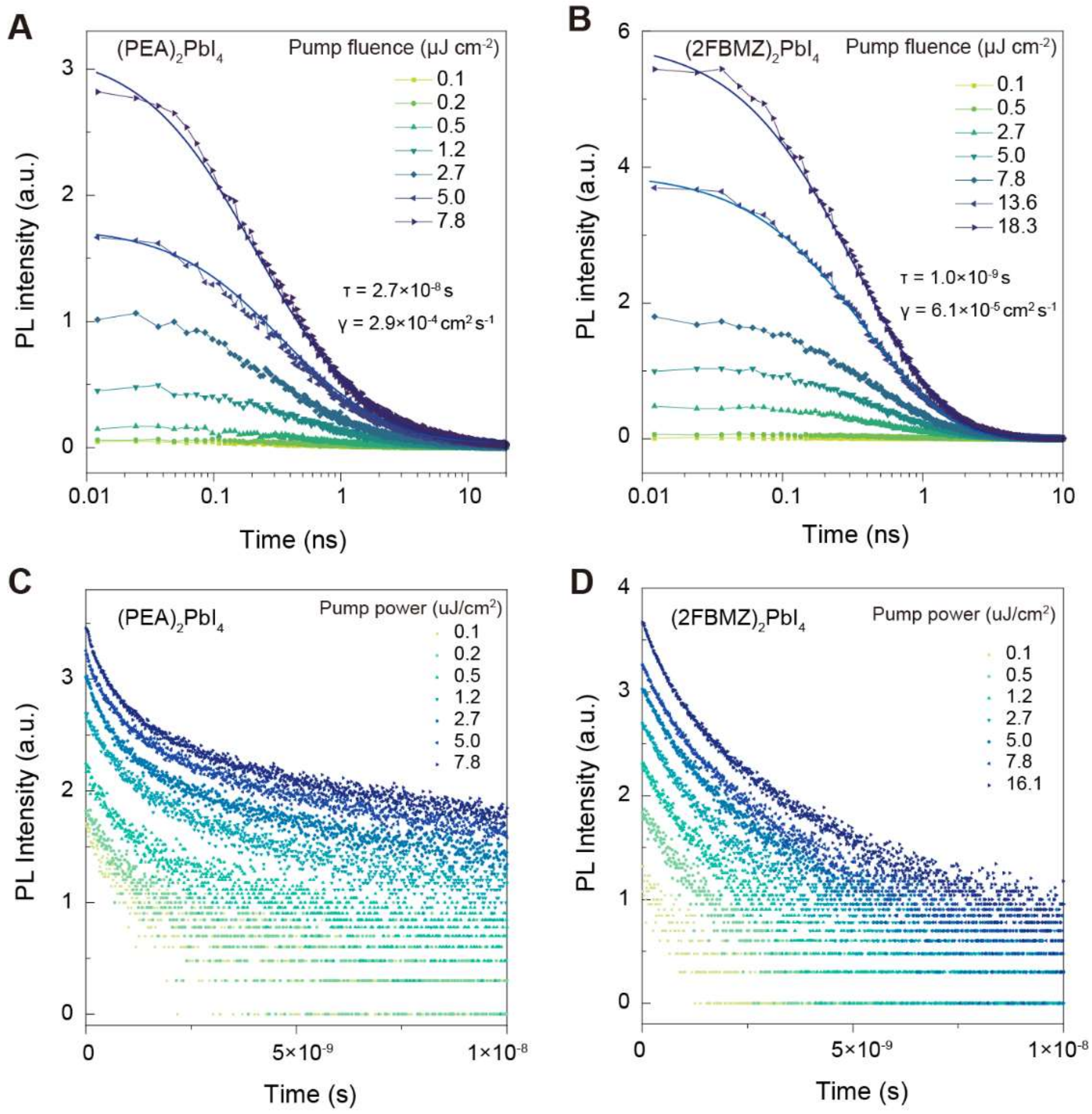

Fig. S18. Time-resolved PL (TRPL) spectra with different excitation fluences. **(A-B)** TRPL spectra plotted with PL intensity on a linear scale and time on a logarithmic scale and the fits of **(A)** $(PEA)_2PbI_4$ and **(B)** $(2FBMZ)_2PbI_4$. Exciton recombination kinetics at high pump fluence, fitted using the rate equation $\frac{dn}{dt} = -\frac{n}{\tau} - \gamma n^2$, where τ is the first-order exciton recombination lifetime, and γ is the EEA rate constant. **(C-D)** TRPL spectra plotted with PL intensity on a logarithmic scale and time on a linear scale of **(C)** $(PEA)_2PbI_4$ and **(D)** $(2FBMZ)_2PbI_4$. These data correspond to those presented in Fig. 3A.

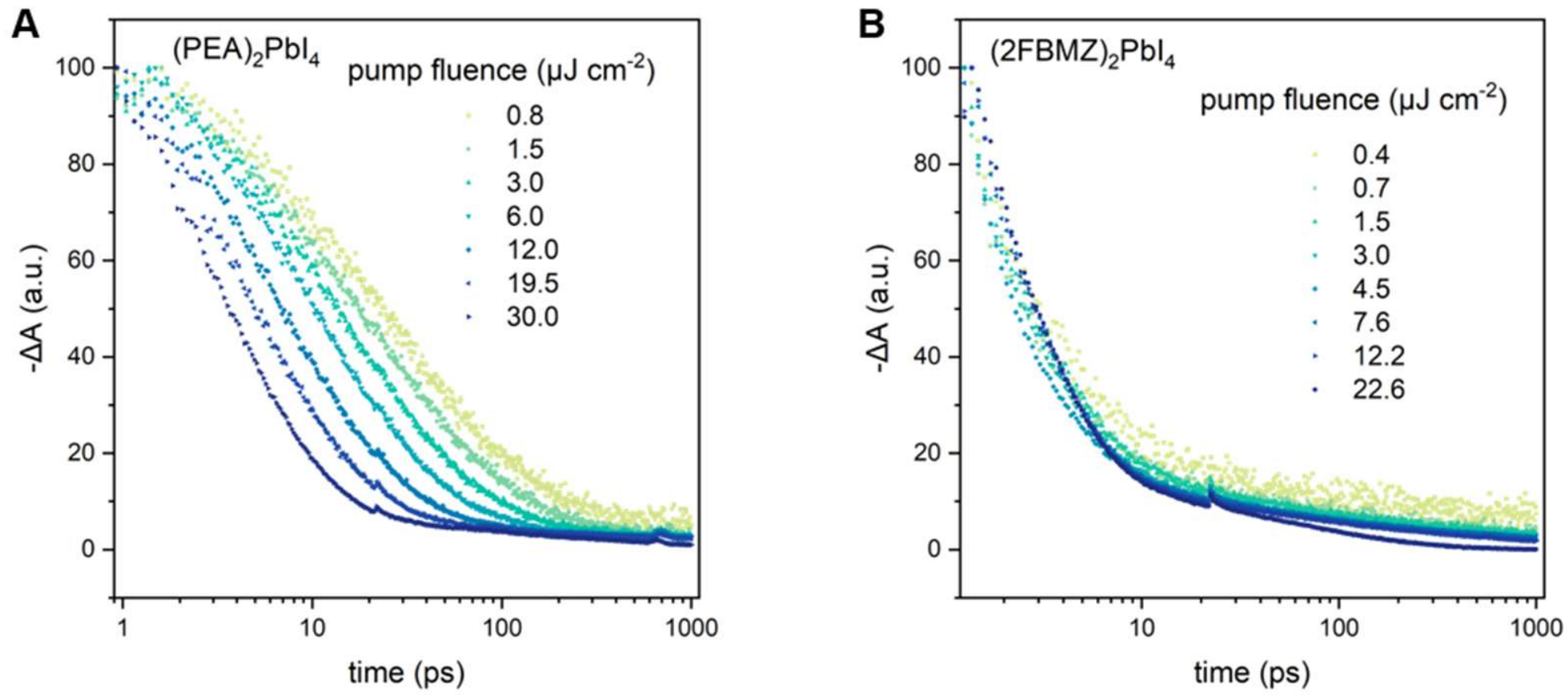

Fig. S19. Normalized transient absorption (TA) measurements for (A) $(PEA)_2PbI_4$ and (B) $(2FBMZ)_2PbI_4$. The TA kinetics of $(PEA)_2PbI_4$ exhibit pronounced pump-fluence-dependent decay dynamics, whereas $(2FBMZ)_2PbI_4$ shows nearly fluence-independent decay behavior, suggesting suppressed EEA in the latter. The ultrafast decay component (<10 ps) observed in TA at low pump fluence is likely associated with fast carrier trapping by surface defects in the film samples. This process is more readily captured by TA, which probes all photoexcited species, including non-radiative channels, whereas TRPL monitors only radiative recombination.

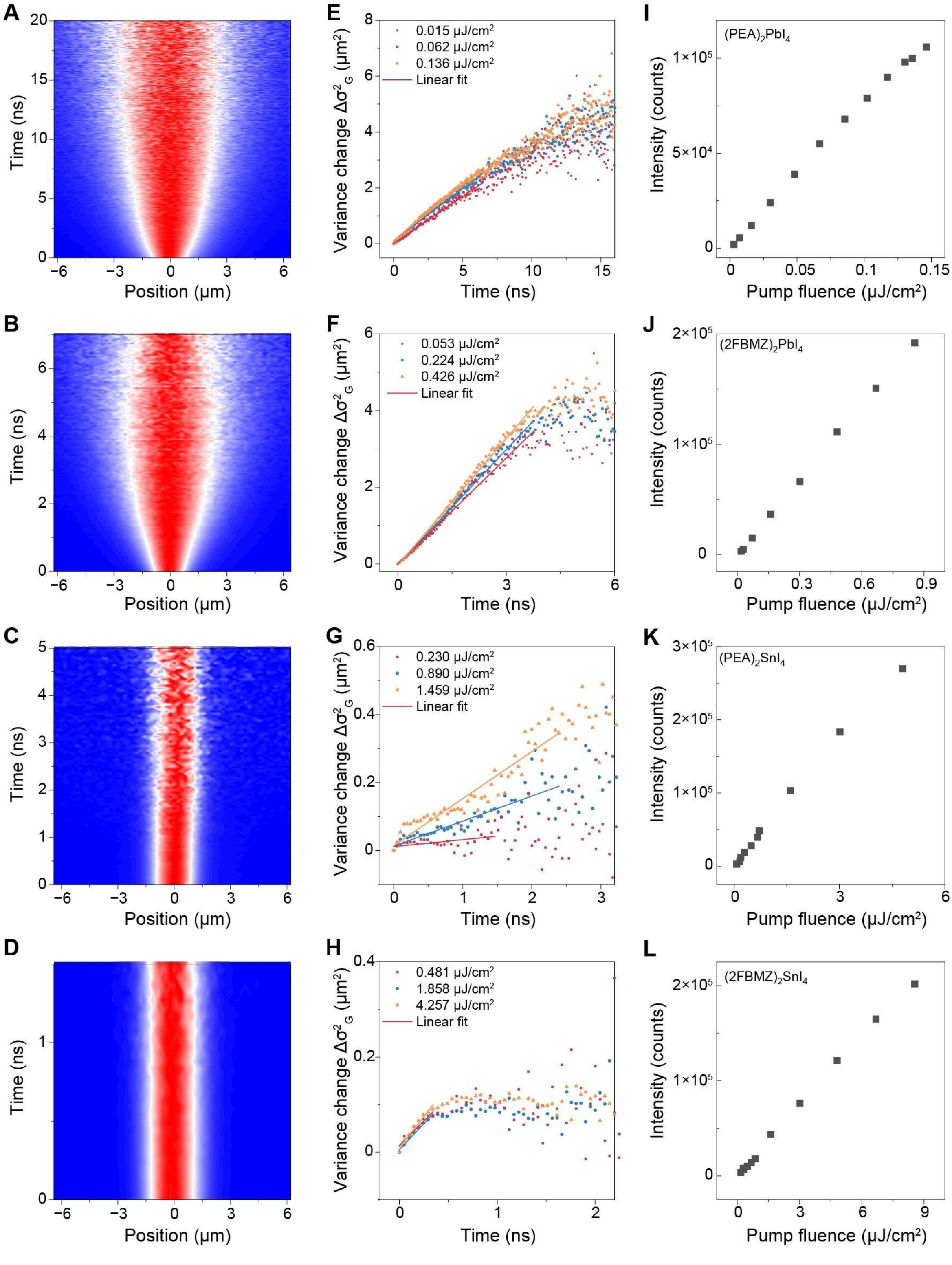

Fig. S20. Excitation-power dependence of exciton diffusion measurements. **(A-D)** Normalized spatial- and time-resolved PL intensity profiles at various time delays of **(A)** $(PEA)_2PbI_4$, **(B)**

$(2FBMZ)_2PbI_4$, **(C)** $(PEA)_2SnI_4$, and **(D)** $(2FBMZ)_2SnI_4$ at 80K. The spatial PL profiles were normalized at each time delay to visualize the temporal broadening of the emission spot. **(E-H)** Time evolution of the variance change $\Delta\sigma_G^2$ for **(E)** $(PEA)_2PbI_4$, **(F)** $(2FBMZ)_2PbI_4$, **(G)** $(PEA)_2SnI_4$, and **(H)** $(2FBMZ)_2SnI_4$ under three different pump fluences. The profiles were fitted with a Voigt function to extract the variance, and the increase in $\Delta\sigma_G^2 = \sigma_G^2(t) - \sigma_G^2(0)$ reflects the exciton diffusion. The exciton diffusivity D was determined from the slope of $\Delta\sigma_G^2$ versus time according to $\Delta\sigma_G^2 = 2Dt$. The $\Delta\sigma_G^2$ shows an initial linear expansion followed by contraction at later times, consistent with previous reports (*67*, *68*). The initial linear regime reflects intrinsic exciton diffusion, whereas the later deviation is associated with exciton trapping by defects. At higher excitation fluence, the initial linear broadening regime becomes more clearly resolved in some samples due to the trap filling. **(I-L)** Linear dependence of PL intensity on pump fluence for **(I)** $(PEA)_2PbI_4$, **(J)** $(2FBMZ)_2PbI_4$, **(K)** $(PEA)_2SnI_4$, and **(L)** $(2FBMZ)_2SnI_4$, confirming that the excitation conditions used for exciton diffusion measurements were within the linear response regime.

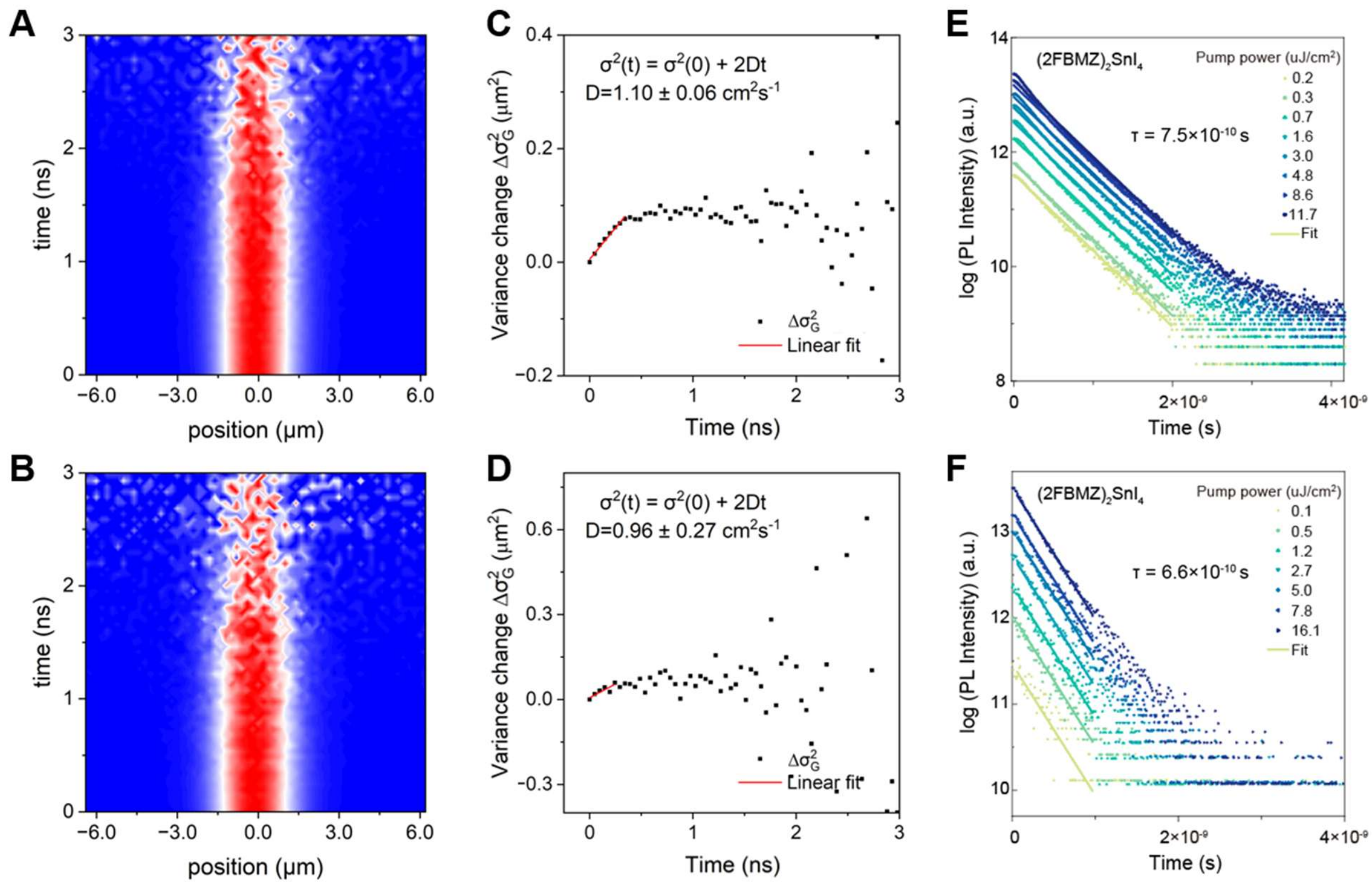

Fig. S21. Comparison between the optical properties of $(2FBMZ)_2SnI_4$ samples prepared under ambient conditions and under inert atmosphere. **(A, B)** Normalized spatial- and time-resolved PL intensity profiles at various time delays of $(2FBMZ)_2SnI_4$ under **(A)** N_2 atmosphere and **(B)** ambient conditions at 80 K. **(C, D)** The temporal broadening of the Gaussian PL distribution ($\Delta\sigma_G^2$) for $(2FBMZ)_2SnI_4$ prepared under **(C)** N_2 atmosphere and **(D)** ambient conditions. The exciton diffusivity (***D***) was extracted from the slope of $\Delta\sigma_G^2$ as a function of time, according to $\sigma_G^2(t) = \sigma_G^2(0) + 2Dt$. **(E, F)** TRPL spectra measured at different excitation fluences of $(2FBMZ)_2SnI_4$ prepared under **(E)** N_2 atmosphere and **(F)** ambient conditions, plotted with PL intensity on a logarithmic scale and time on a linear scale. The solid lines represent single-exponential fits, yielding lifetimes (τ) of 0.75 ns and 0.66 ns, respectively. Over all, the differences between the optical properties of Sn-based samples prepared under ambient conditions and inert atmosphere are not significant.

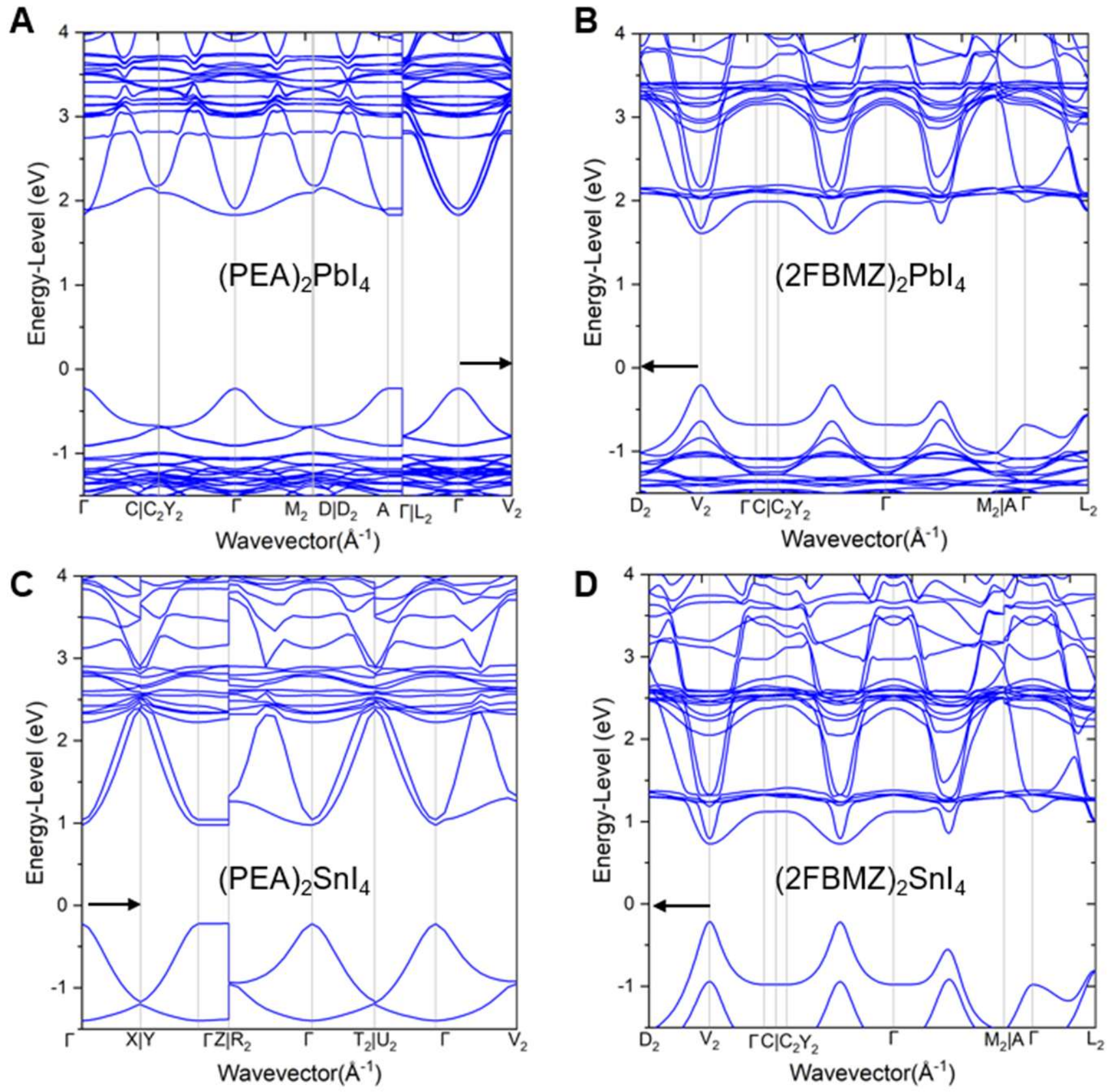

Fig. S22. Band structures of **(A)** $(PEA)_2PbI_4$, **(B)** $(2FBMZ)_2PbI_4$, **(C)** $(PEA)_2SnI_4$, and **(D)** $(2FBMZ)_2SnI_4$. The arrows indicate the K-paths used to calculate the carrier effective masses reported in Table S8.

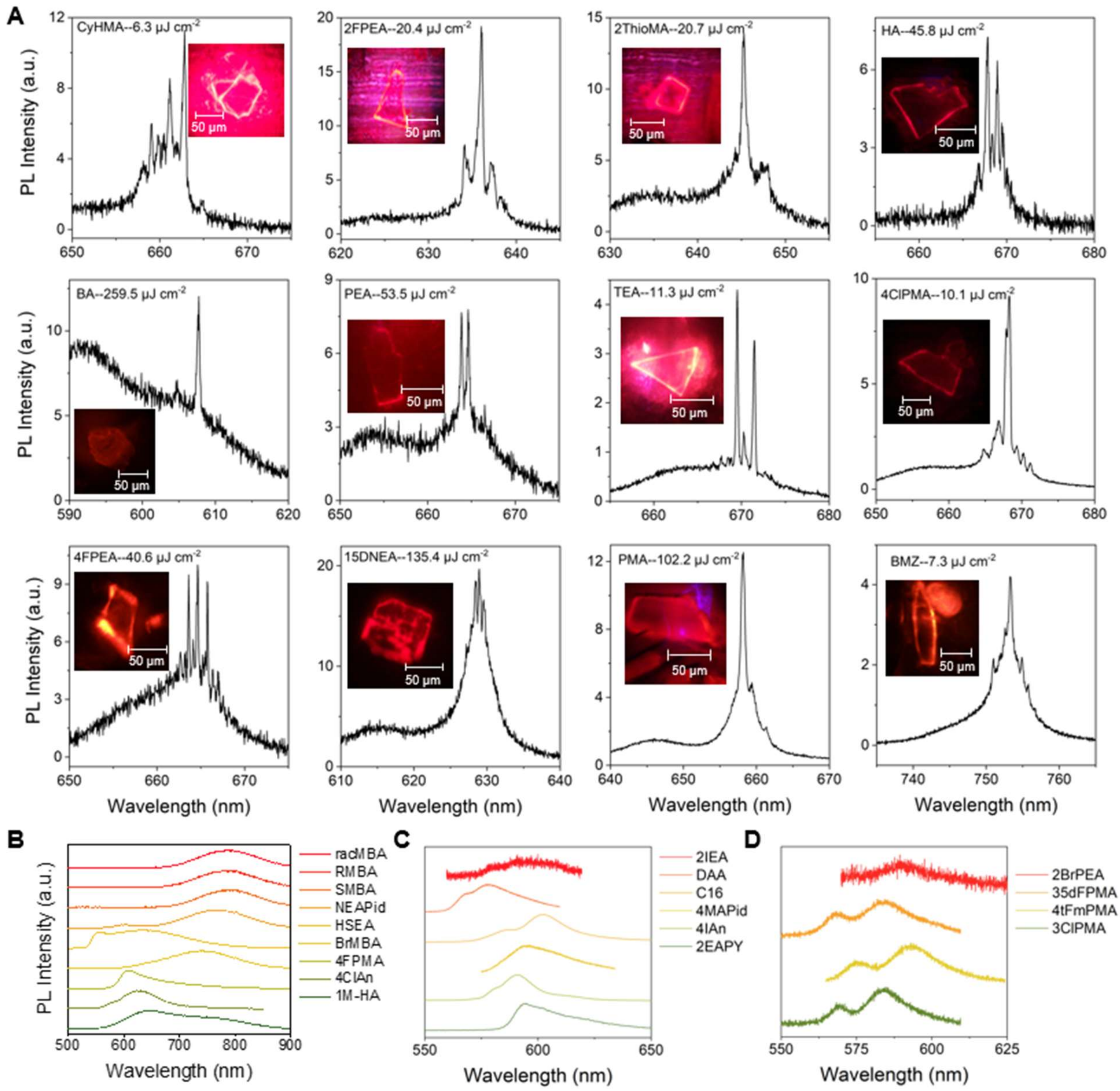

Fig. S23. PL spectra of various Sn-based 2D perovskites at 80 K under high pump fluence. **(A)** Lasing spectra of various Sn-based 2D perovskites exhibiting stimulated emission. Insets show optical images of the microcrystals above lasing threshold. **(B-D)** PL spectra under high pump fluence of various Sn-based 2D perovskites that do not lase, categorized into three groups: **(B)** structures showing self-trapped exciton emission or free exciton emission with large AF values; **(C)** structures exhibiting free exciton emission with moderately large AF values, some of which are near the boundary of the lasing region; **(D)** structures with large out-of-plane tilting and photo-instability, where only fleeting lasing is occasionally observed before rapid photodegradation.

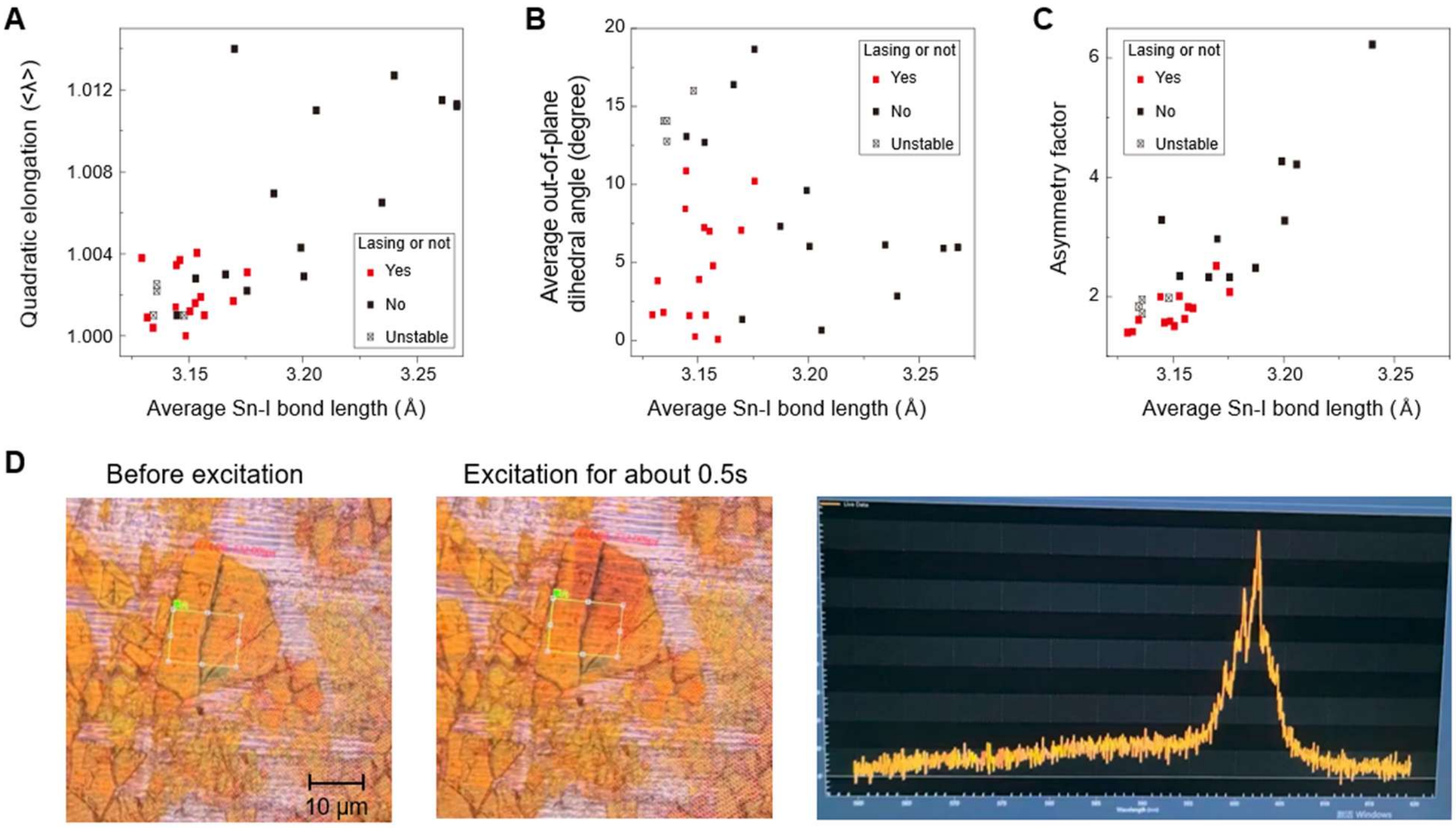

Fig. S24. Structure–lasing correlation in 2D Sn-based perovskites, highlighting the structural parameters of lasing-capable compounds. **(A)** Map of out-of-plane octahedral tilting dihedral angle versus Sn–I bond length, showing lasing structures located in the region with short bond lengths and small dihedral angles. **(B)** Map of octahedral distortion versus Sn–I bond length, with lasing structures concentrated in the region of short bond lengths. **(C)** Map of PL asymmetry factor versus Sn–I bond length, indicating that lasing structures cluster in the region with short bond lengths and low PL asymmetry factors. A list of structures we tested or have been reported in literatures is shown in Table S8, where the PL spectra of structures we tested are shown in Fig. S23. We note that $(3ClPMA)_2SnI_4$, $(35dFPMA)_2SnI_4$, $(4tFmPMA)_2SnI_4$, and $(2BrPEA)_2SnI_4$ (labeled as black empty squares) are very unstable under photoexcitation even at 80 K, as shown in **(D)**, where we only get a capture fleeting moment of lasing action. Although transient lasing can occasionally be observed, the samples degrade quickly. In **(D)**, the left panel displays the crystal before excitation, the middle panel shows the crystal after 0.5 seconds of excitation, where noticeable color deepening occurs at the irradiated spot. The corresponding spectrum is presented on the right. These compounds exhibit large out-of-plane octahedral tilting, which leads to higher PL asymmetry factors despite their relatively short Sn–I bond lengths. We believe that the Sn–I bond length plays a primary role in determining whether a Sn-based structure can lase. Under this condition, large out-of-plane octahedral tilting is generally unfavorable due to photostability issues and elevated asymmetry factors.

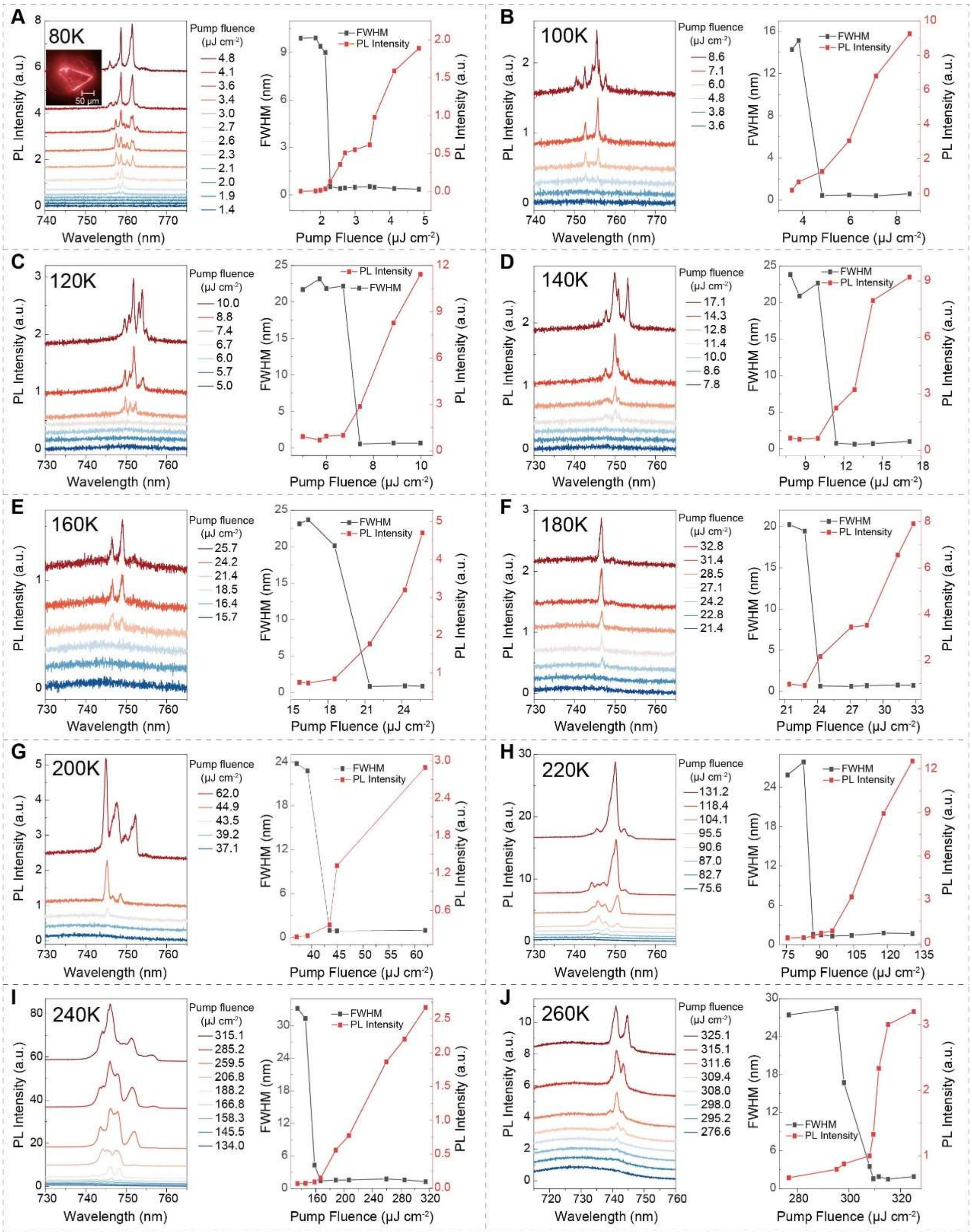

Fig. S25. Emission spectra of $(2FBMZ)_2SnI_4$ under increasing pump fluences, and corresponding FWHM and integrated PL intensity as functions of pump fluence at 80-260 K. **(A)** 80 K. Inset shows an optical image of the microcrystal above lasing threshold. **(B)** 100 K. **(C)** 120 K. **(D)** 140 K. **(E)** 160 K. **(F)** 180 K. **(G)** 200 K. **(H)** 220 K. **(I)** 240 K. **(J)** 260 K. These results revealing a lasing threshold increases from 2.3 μJ cm^{-2} at 80 K to 309.4 μJ cm^{-2} at 260 K.

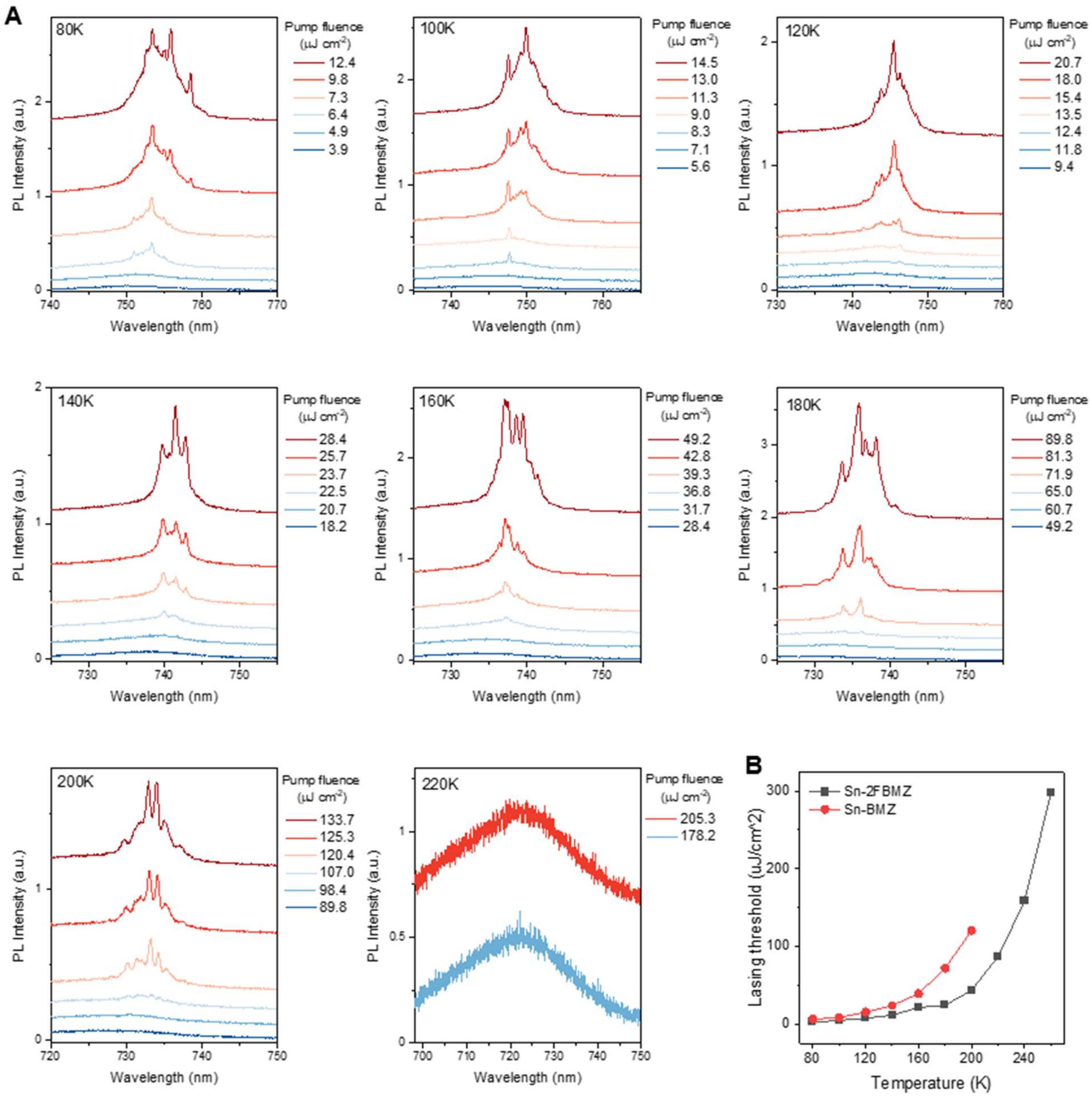

Fig. S26. Comparison of lasing performance between $(BMZ)_2SnI_4$ and $(2FBMZ)_2SnI_4$. **(A)** Emission spectra of $(BMZ)_2SnI_4$ under increasing pump fluences at 80-220 K, showing that lasing persists up to 200 K. **(B)** Comparison of temperature-dependent lasing thresholds of $(BMZ)_2SnI_4$ and $(2FBMZ)_2SnI_4$.

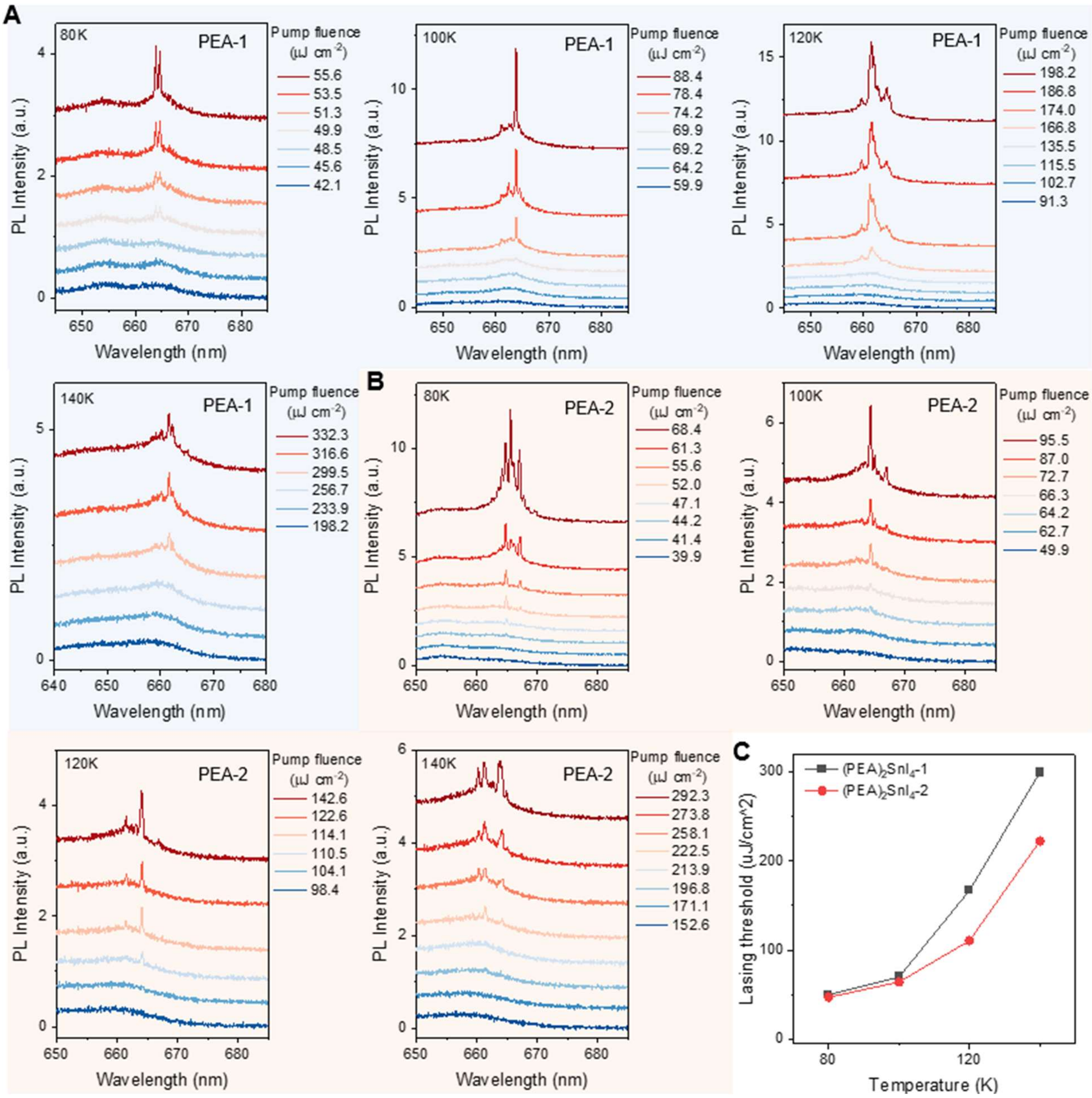

Fig. S27. Lasing performance of $(PEA)_2SnI_4$. **(A-B)** Emission spectra of two $(PEA)_2SnI_4$ samples under increasing pump fluences at 80-160 K. **(C)** Temperature-dependent lasing thresholds of the two samples we tested. The threshold of $(PEA)_2SnI_4$ in Fig. 4F comes from the average value of these two samples.

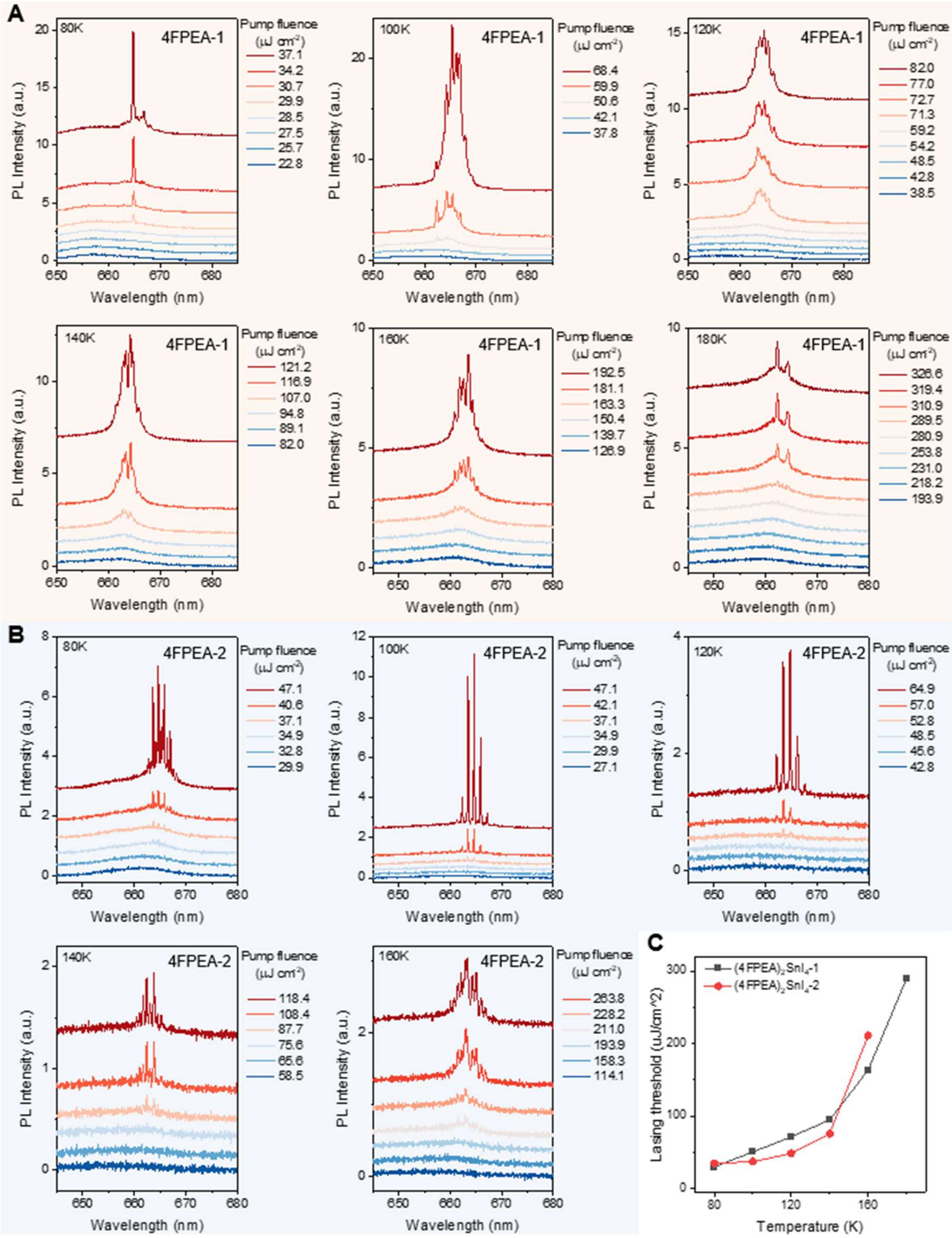

Fig. S28. Lasing performance of $(4FPEA)_2SnI_4$. Emission spectra of two $(4FPEA)_2SnI_4$ samples under increasing pump fluences at **(A)** 80-180 K and **(B)** 80-160 K. **(C)** Temperature-dependent lasing thresholds of the two samples we tested. The threshold of $(4FPEA)_2SnI_4$ in Fig. 4F comes from the average value of these two samples.

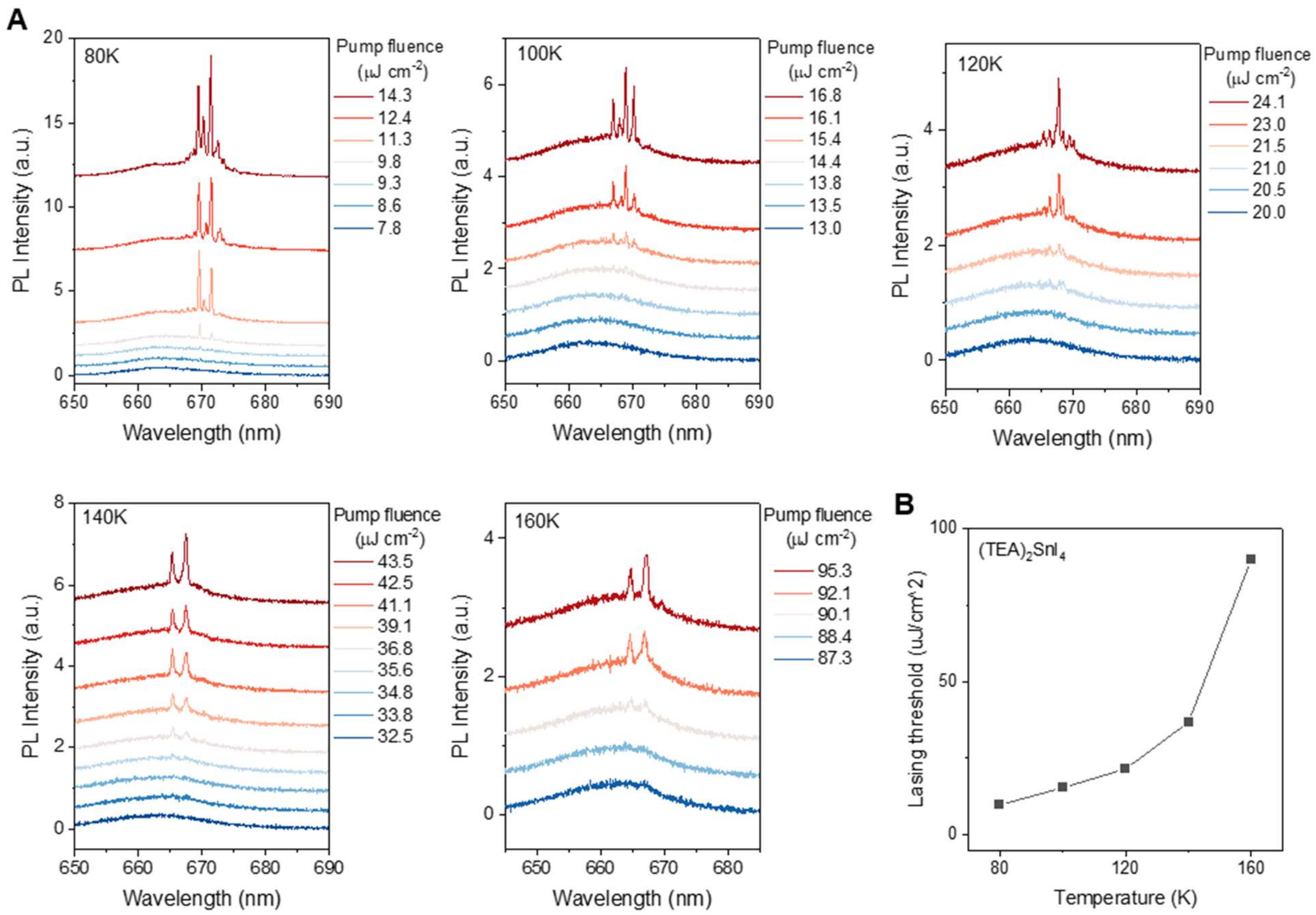

Fig. S29. Lasing performance of $(TEA)_2SnI_4$. **(A)** Emission spectra under increasing pump fluences at 80-160 K. **(B)** Temperature-dependent lasing thresholds.

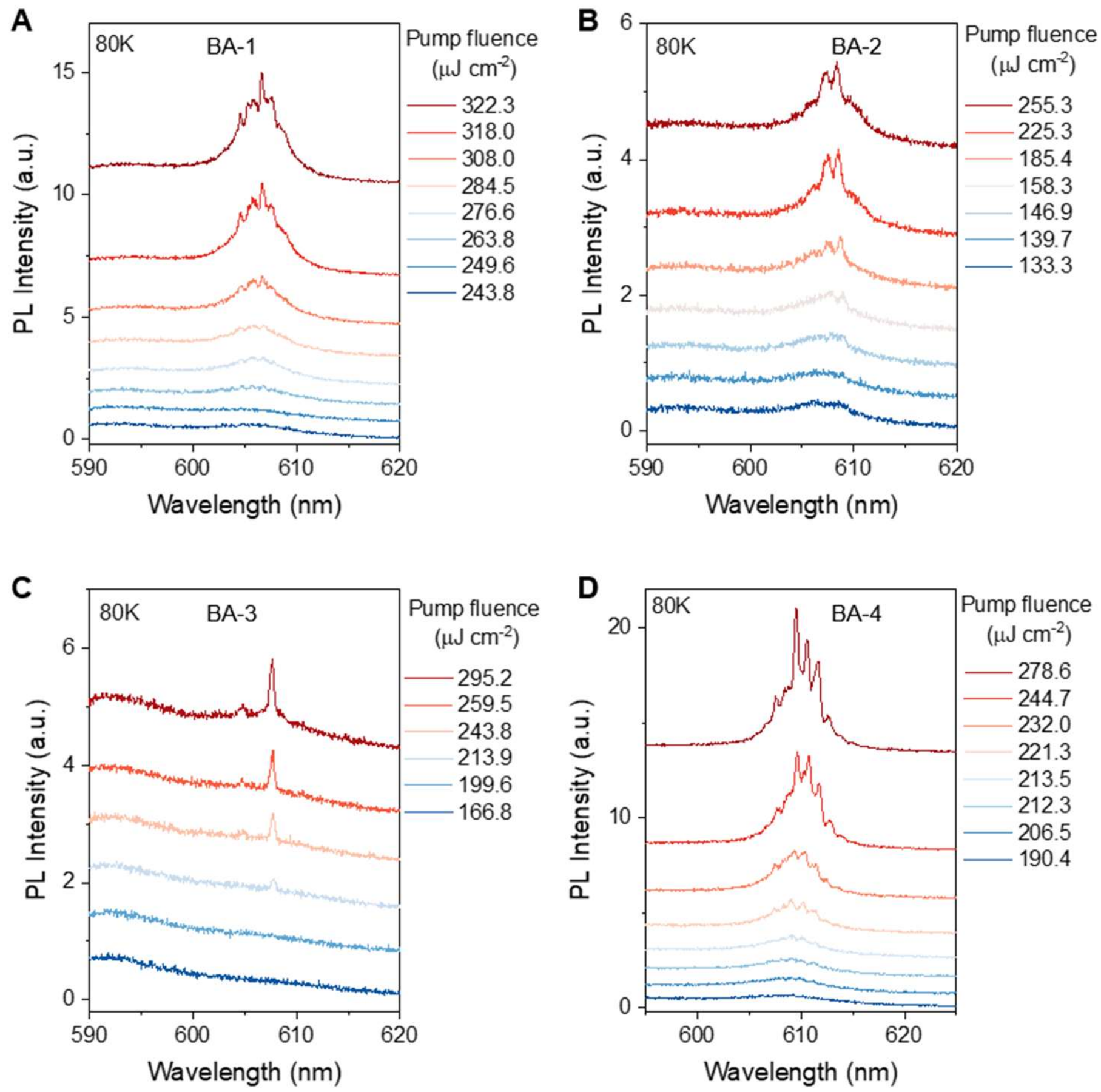

Fig. S30. Lasing performance of $(BA)_2SnI_4$. **(A-D)** Emission spectra of four $(BA)_2SnI_4$ samples under increasing pump fluences at 80K. The threshold of $(BA)_2SnI_4$ in Fig. 4F comes from the average value of these four samples.

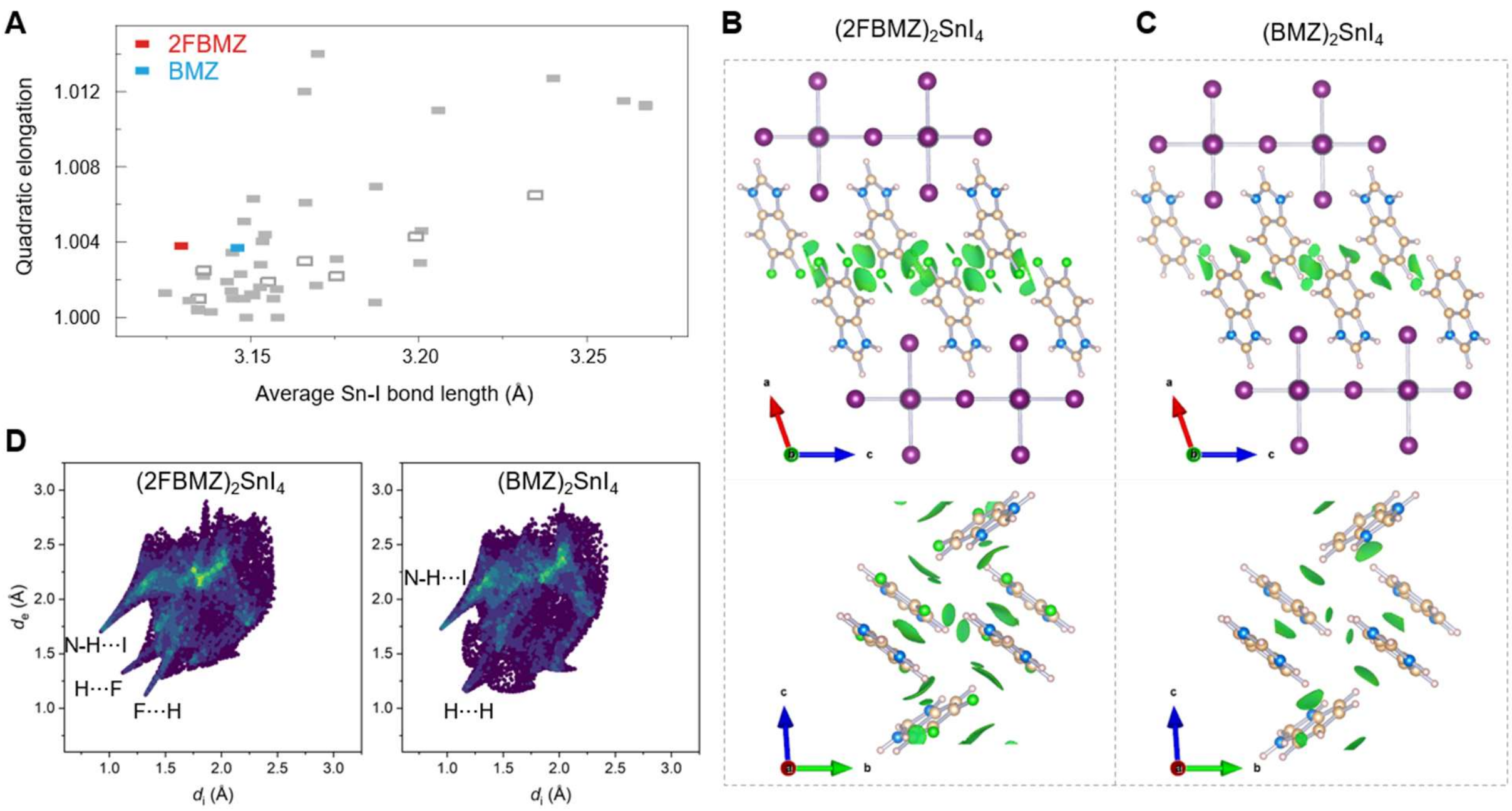

Fig. S31. Difference in distortion and intermolecular forces between $(2FBMZ)_2SnI_4$ and $(BMZ)_2SnI_4$. **(A)** Structural correlation map of quadratic elongation versus average bond length for all Sn-based structures studied, with the positions of $(2FBMZ)_2SnI_4$ and $(BMZ)_2SnI_4$ highlighted. **(B-C)** Intermolecular interactions between the spacer cations for **(B)** $(2FBMZ)_2SnI_4$ and **(C)** $(BMZ)_2SnI_4$, shown as green regions representing the independent gradient model based on Hirshfeld partition (IGMH) isosurfaces at $\delta g^{inter} = 0.005$ a.u. $(2FBMZ)_2SnI_4$ features multiple intermolecular interactions (F···π halogen bonds, F···H hydrogen bonds, and F···F dispersion forces), while $(BMZ)_2SnI_4$ exhibits H···H van der Waals contacts. **(D)** Hirshfeld surface fingerprint plots of $(2FBMZ)_2SnI_4$ and $(BMZ)_2SnI_4$, highlighting the differing intermolecular interactions. The *x*-axis (d_i) and the *y*-axis (d_e) represent the distances from a point on the Hirshfeld surface to the nearest nucleus internal and external to the surface, respectively. Each plot is color-mapped according to point density using a viridis color scale, with low-density regions appearing purple and high-density regions appearing yellow.

Table S1. Crystal structure refinement of newly synthesized 2D perovskites.

Structure	$(2FBMZ)_2PbI_4$	$(4tFmPMA)_2SnI_4$	$(4ClPMA)_2SnI_4$	$(4ClAn)_2SnI_4$
Empirical formula	$C_{14}H_{10}F_4PbI_4N_4$	$C_{16}H_{18}F_6SnI_4N_2$	$C_{13}H_{16}Cl_2SnI_4N_2$	$C_{12}H_{14}Cl_2SnI_4N_2$
Crystal system	monoclinic	monoclinic	monoclinic	monoclinic
Space group	*I2/a*	$P2_1/c$	$P2_1/c$	$P2_1/c$
Unit cell dimensions	a = 12.5966(3) Å, α = 90°	a = 18.2742(8) Å, α = 90°	a = 16.9029(8) Å, α = 90°	a = 15.4205(6) Å, α = 90°
	b = 6.2867(2) Å, β = 94.558(3) °	b = 8.5209(3) Å, β = 97.859(4) °	b = 8.7967(4) Å, β = 104.299(5) °	b = 8.1306(3) Å, β = 99.982(3) °
	c = 30.0956(9) Å, γ = 90°	c = 8.6155(3) Å, γ = 90°	c = 8.6482(4) Å, γ = 90°	c = 9.0386(3) Å, γ = 90°
Volume	2375.76(12) $Å^3$	1328.93(9) $Å^3$	1246.06(10) $Å^3$	1116.09(7) $Å^3$
Z	8	4	2	2
F000	1808.0	888.0	824.0	792.0
h,k,lmax	17,8,39	25,11,11	20,10,10	21,11,12
Nref	2952	3349	2197	2763
Tmin,Tmax	0.524,1.000	0.450,1.000	0.653,1.000	0.630,1.000
Data completeness	0.889	0.918	1.000	0.891
Theta(max)	29.521	29.332	25.000	29.459
R(reflections)	0.0271 (2543)	0.0307 (2590)	0.0196 (1930)	0.0286 (2288)
wR2(reflections)	0.0740 (2952)	0.0750 (3349)	0.0453 (2197)	0.0679 (2763)

Crystal structure refinement of newly synthesized 2D perovskites (continued)

Structure	$(4IAn)_2SnI_4$	$(4FPMA)_2SnI_4$	$(3ClPMA)_2SnI_4$	$(SBrMBA)_2SnI_4$
Empirical formula	$C_{12}H_{14}SnI_6N_2$	$C_{14}H_{18}F_2SnI_4N_2$	$C_{14}H_{18}Cl_2SnI_4N_2$	$C_{16}H_{22}Br_2SnI_4N_2$
Crystal system	monoclinic	monoclinic	monoclinic	tetragonal
Space group	*P*2₁/*c*	*P*2₁/*n*	*P*2₁/*c*	*P*4₁2₁2
Unit cell dimensions	a = 15.7290(8) Å, α = 90°	a = 8.7254(3) Å, α = 90°	a = 17.4986(8) Å, α = 90°	a = 9.0022(2) Å, α = 90°
	b = 8.2576(4) Å, β = 100.181(4) °	b = 9.2905(3) Å, β = 98.009(3) °	b = 8.2076(4) Å, β = 100.199(5) °	b = 9.0022(2) Å, β = 90°
	c = 9.1466(4) Å, γ = 90°	c = 27.8298(9) Å, γ = 90°	c = 8.7066(4) Å, γ = 90°	c = 65.4006(18) Å, γ = 90°
Volume	1169.30(10) $Å^3$	2233.97(13) $Å^3$	1230.70(10) $Å^3$	5300.0(3) $Å^3$
Z	4	4	2	8
F000	936.0	1584.0	824.0	3712.0
h,k,lmax	20,10,11	11,12,38	20,9,10	12,12,89
Nref	2618	5629	2171	6446
Tmin,Tmax	0.235,1.000	0.579,1.000	0.425,1.000	0.568,1.000
Data completeness	0.977	0.909	1.000	1.45/0.87
Theta(max)	27.489	29.434	25.000	29.467
R(reflections)	0.0457 (2193)	0.0268 (4350)	0.0237 (1927)	0.0602 (4939)
wR2(reflections)	0.1118 (2618)	0.0554 (5629)	0.0587 (2171)	0.1330 (6446)

Crystal structure refinement of newly synthesized 2D perovskites (continued)

Structure	$(4BrAn)_2GeI_4$	$(4ClAn)_2GeI_4$	$(Nm\text{-}4ClAn)_2GeI_4$	$(Nm\text{-}4BrAn)_2GeI_4$
Empirical formula	$C_{12}H_{14}Br_2GeI_4N_2$	$C_{12}H_{14}Cl_2GeI_4N_2$	$C_{14}H_{18}Cl_2GeI_4N_2$	$C_{14}H_{18}Br_2GeI_4N_2$
Crystal system	monoclinic	monoclinic	monoclinic	monoclinic
Space group	$P2_1/c$	$P2_1/c$	$P2_1/n$	$P2_1/n$
Unit cell dimensions	a = 30.6357(14) Å, α = 90°	a = 30.4134(10) Å, α = 90°	a = 8.6674(3) Å, α = 90°	a = 8.6936(5) Å, α = 90°
	b = 7.9257(3) Å, β = 96.793(4) °	b = 7.8574(3) Å, β = 96.751(3) °	b = 31.5650(13) Å, β = 89.997(3) °	b = 31.681(2) Å, β = 90.127(5) °
	c = 9.0659(3) Å, γ = 90°	c = 8.9748(3) Å, γ = 90°	c = 8.7338(3) Å, γ = 90°	c = 8.8080(4) Å, γ = 90°
Volume	2185.83(15) Å^3	2129.84(13) Å^3	2389.45(15) Å^3	2425.9(2) Å^3
Z	4	4	4	4
F000	1656.0	1512.0	1576.0	1720.0
h,k,lmax	40,10,12	41,10,12	10,37,10	10,37,10
Nref	5500	5352	4144	3750
Tmin,Tmax	0.661,1.000	0.411,1.000	0.711,1.000	0.729,1.000
Data completeness	0.904	0.899	0.983	0.877
Theta(max)	29.470	29.538	25.000	24.990
R(reflections)	0.0344 (4482)	0.0319 (4308)	0.0287 (3502)	0.0338 (2962)
wR2(reflections)	0.0855 (5500)	0.0755 (5352)	0.0620 (4144)	0.0812 (3750)

Crystal structure refinement of newly synthesized 2D perovskites (continued)

Structure	$(4iPPMA)_2GeI_4$	$(2FPEA)_2GeI_4$	$(R\text{-}BrMBA)_2GeI_4$	$(2PYEA)GeI_4$
Empirical formula	$C_{20}H_{32}GeI_4N_2$	$C_{16}H_{21}F_2GeI_4N_2$	$C_{16}H_{22}Br_2GeI_4N_2$	$C_7H_{12}GeI_4N_2$
Crystal system	orthorhombic	triclinic	orthorhombic	orthorhombic
Space group	*Pbcn*	*P*-1	$P2_12_12_1$	*Pbca*
Unit cell dimensions	a = 8.6949(3) Å, α = 90°	a = 12.0191(7) Å, α = 107.357(5) °	a = 9.1941(3) Å, α = 90°	a = 17.8532(6) Å, α = 90°
	b = 8.3483(3) Å, β = 90°	b = 12.0967(6) Å, β = 99.670(5) °	b = 25.5568(9) Å, β = 90°	b = 8.6761(3) Å, β = 90°
	c = 39.3081(18) Å, γ = 90°	c = 17.6365(11) Å, γ = 90.056(4) °	c = 33.6148(12) Å, γ = 90°	c = 20.4494(9) Å, γ = 90°
Volume	2853.28(19) $Å^3$	2409.1(2) $Å^3$	7898.5(5) $Å^3$	3167.5(2) $Å^3$
Z	4	2	4	8
F000	1640.0	1572.0	5352.0	2496.0
h,k,lmax	10,9,46	14,14,20	13,35,46	24,12,28
Nref	2510	8456	19936	4133
Tmin,Tmax	0.566,1.000	0.711,1.000	0.346,1.000	0.122,1.000
Data completeness	1.000	1.000	1.53/0.84	0.880
Theta(max)	25.000	25.000	30.396	30.178
R(reflections)	0.1274 (2220)	0.0499 (6126)	0.0385 (16079)	0.0310 (3162)
wR2(reflections)	0.2823 (2510)	0.1116 (8456)	0.0988 (19936)	0.0845 (4133)

Crystal structure refinement of newly synthesized 2D perovskites (continued)

Structure	(4aiPPD)GeI_4	(4aEtPD)GeI_4	(DmDPA)$_2GeI_4$	(2FBMZ)$_2GeI_4$
Empirical formula	$C_8H_{20}GeI_4N_2$	$C_7H_{18}GeI_4N_2$	$C_5H_{16}GeI_4N_2$	$C_{14}H_{10}F_4GeI_4N_4$
Crystal system	monoclinic	monoclinic	monoclinic	orthorhombic
Space group	*C2/c*	*C2/c*	*P*2$_1$/*c*	*Pnma*
Unit cell dimensions	a = 21.5484(4) Å, α = 90°	a = 21.0210(4) Å, α = 90°	a = 8.3304(2) Å, α = 90°	a = 12.1670(7) Å, α = 90°
	b = 12.6382(2) Å, β = 103.309(2) °	b = 12.4859(2) Å, β = 99.154(2) °	b = 20.7900(5) Å, β = 90.591(2) °	b = 30.5427(19) Å, β = 90°
	c = 13.5262(3) Å, γ = 90°	c = 12.8898(3) Å, γ = 90°	c = 18.2606(5) Å, γ = 90°	c = 6.0505(3) Å, γ = 90°
Volume	3584.70(12) Å^3	3340.05(11) Å^3	3162.37(14) Å^3	2248.4(2) Å^3
Z	8	8	4	8
F000	2608.0	2544.0	2432.0	1608.0
h,k,lmax	25,15,16	24,14,15	9,24,21	16,41,8
Nref	3144	2940	5316	2898
Tmin,Tmax	0.608,1.000	0.782,1.000	0.800,1.000	0.410,1.000
Data completeness	1.000	1.000	1.000	0.914
Theta(max)	25.000	25.000	25.000	29.409
R(reflections)	0.0173 (2930)	0.0191 (2758)	0.0242 (4704)	0.0289 (2472)
wR2(reflections)	0.0398 (3144)	0.0463 (2940)	0.0619 (5316)	0.0689 (2898)

$R = \Sigma||F_o|-|F_c|| / \Sigma|F_o|$, $wR = \{\Sigma[w(|F_o|^2 - |F_c|^2)^2] / \Sigma[w(|F_o|^2)^2]\}^{1/2}$ and $w=1/[\sigma^2(Fo^2)+(0.0833P)^2+3.5012P]$ where $P=(Fo^2+2Fc^2)/3$

Table S2. Summary of structural parameters, including the average B–I bond length ($\bar{\boldsymbol{d}}$), quadratic elongation ($\langle\boldsymbol{\lambda}\rangle$), bond angle variance ($\boldsymbol{\sigma_{\theta}^2}$), and I-B-B-I dihedral angle for the surveyed Pb-, Sn-, and Ge-based iodide perovskites. The data presented here correspond to those in Fig. 1C-E and Fig. S2.

B-cation	LA-cation	LA-Abbreviation	$\bar{d}$ (Å)	$\langle\lambda\rangle$	σ_{θ}^2 (deg^2)	dihedral angle (deg)
Pb	Acetone-[bis-(2-amino-ethyl)-dithioacetal	dSEA-Act	3.1671	1.0013	4.35	5.21
	5,6-difluoro-1H-benzimidazole	2FBMZ	3.1731	1.0036	12.07	1.04
	4-fluorophenylethylammonium	4FPEA	3.1752	1.0013	4.17	3.69
	Bis(2-aminoethylthio)methane	dSEA-Met	3.1759	1.0012	3.96	5.44
	Phenylethylammonium	PEA	3.1763	1.0009	2.98	3.90
	4-phenylphenethylammonium	4PhPEA	3.1787	1.0006	1.64	2.02
	2-bromophenylethylammonium	2BrPEA	3.1794	1.0047	16.15	13.06
	DL-histidine	Hist	3.1794	1.0072	25.13	0
	4-(trifluoromethyl)benzylammonium	4tFmPMA	3.1823	1.0015	5.53	14.27
	Thiophene-2-ethylammonium	TEA	3.1828	1.0012	3.94	4.48
	1,12-dodecanediammonium	C12DA	3.1844	1.0006	2.16	0.09
	2-(3,5-difluorophenyl)ethanammonium	35dFPEA	3.1850	1.0034	9.44	1.42
	2-thiophenemethylammonium	2ThioMA	3.1860	1.0019	6.62	7.12
	2-(3,5-dimethylphenyl)ethanammonium	35dMePEA	3.1868	1.0024	8.46	8.08
	4-isopropylbenzylammonium	4iPPMA	3.1877	1.0011	4.03	15.48
	2-iodopropylammonium	2IPA	3.1878	1.0001	0.16	1.56
	Amylammonium	AA	3.1878	1.0016	5.69	17.09
	Butylammonium	BA	3.1882	1.0016	5.64	7.46
	2-(2,3,5,6-tetrafluorophenoxy)-ethanammonium	2356-4FPhOEA	3.1885	1.0006	2.22	5.20
	Heptanammonium	HEA	3.1891	1.0014	4.56	7.71
	2-(2,4,5-trifluorophenyl)ethanammonium	245-3FPEA	3.1894	1.0032	9.38	1.46
	4-chlorophenylmethylammonium	4ClPMA	3.1907	1.0021	6.89	7.83
	Cyclohexanemethanammonium	CyHMA	3.1907	1.0014	4.78	5.11
	4-bromophenylmethylammonium	4BrPMA	3.1909	1.0025	8.40	8.06
	2-(2,3,4,5,6-pentafluorophenyl)ethanammonium	23456-5FPEA	3.1912	1.0000	0.003	0
	2-methylbutylammonium	2M-BA	3.1924	1.0005	1.88	4.36
	3-chloro-N-methylbenzenemethanammonium	Nm3ClPMA	3.1940	1.0071	23.03	8.39

B-cation	LA-cation	LA-Abbreviation	$\bar{d}$ (Å)	$\langle\lambda\rangle$	σ_θ^2 (deg^2)	dihedral angle (deg)
	2-(1-cyclohexenyl)ethylammonium	2-CyHe-EA	3.1941	1.0051	14.62	2.50
	2-fluorophenylethylammonium	2FPEA	3.1942	1.0008	2.72	2.02
	Hexylammonium	HA	3.1943	1.0019	6.67	7.26
	2-methylpropylammonium	2M-PA	3.1960	1.0020	6.89	8.36
	Octylammonium	OA	3.1963	1.0020	6.88	7.80
	4-methoxybenzylammonium	4MeOPMA	3.1964	1.0044	12.18	2.26
	Nonylammonium	NOA	3.1965	1.0020	6.99	9.02
	4-fluoro-N-methylaniline	Nm4FAn	3.1968	1.0072	23.90	15.15
	4-chloroaniline	4ClAn	3.1974	1.0029	9.61	16.87
	Morpholine	Morph	3.1981	1.0055	19.89	0
	4-bromoaniline	4BrAn	3.2000	1.0026	8.78	17.59
	Isoamylammonium	iBA	3.2001	1.0001	0.03	0.09
	3-fluoro-N-methylaniline	Nm3FAn	3.2008	1.0078	27.45	14.88
	2-ethyl-hexylammonium	2EtHA	3.2020	1.0013	4.70	5.64
	3-iodopropylammonium	3IPA	3.2060	1.0034	12.20	15.82
	2-iodoethanammonium	2IEA	3.2080	1.0009	2.74	20.92
	2-bromoethylammonium	2BrEA	3.2095	1.0007	1.74	0
	2-fluorophenylmethylammonium	2FPMA	3.2115	1.0033	11.50	9.10
	6-iodo-hexylammonium	6IHA	3.2115	1.0032	10.97	10.66
	1,2-diaminocyclohexane	CyHDA	3.2142	1.0039	12.62	3.47
	4-aminocyclohexane-1-carboxylic acid	4ACyHCOOH	3.2179	1.0025	7.94	6.70
	2-chloroethylammonium	2ClEA	3.2182	1.0026	8.68	2.37
	Benzotriazole-ethanammonium	BTZ-EA	3.2221	1.0064	17.62	2.52
	1-methyl-hexylammonium	1M-HA	3.2230	1.0031	10.60	6.19
	2-(5,6-difluorobenzotriazol-2-yl)ethanamine	2F-BTZ-EA	3.2242	1.0067	19.66	2.24
	Cysteammonium	HSEA	3.2250	1.0062	15.27	2.63
	3,3-difluoropyrrolidine	33dFPyd	3.2271	1.0025	9.10	10.01
	4,4-difluoropiperidine	44dFPid	3.2274	1.0041	13.27	9.79
	4-aminobutyric acid	GABA	3.2274	1.0046	14.58	5.07
	4-fluorophenylmethylammonium	4FPMA	3.2305	1.0042	13.45	9.97
	Cyclohexanammonium	CyHA	3.2323	1.0030	9.26	9.12
	3-aminopropionitrile	CNEA	3.2377	1.0091	28.56	3.71
	S-1-(4-bromophenyl)ethan-1-ammonium	SBrMBA	3.2434	1.0044	13.06	7.05
	4,4-difluorocyclohexammonium	44dFCyHA	3.2482	1.0131	43.73	7.80
	3-nipecotic acid	3PidCOOH	3.2558	1.0190	49.87	4.07
	4,4-difluoroazepane	44dFAzp	3.2580	1.0191	55.11	7.64

B-cation	LA-cation	LA-Abbreviation	$\bar{d}$ (Å)	$\langle\lambda\rangle$	σ_θ^2 (deg^2)	dihedral angle (deg)
	S-alpha-methylbenzylammonium	SMBA	3.2613	1.0061	18.93	8.09
	S-1-(4-chlorophenyl)ethan-1-ammonium	SClMBA	3.2378	1.0041	12.59	7.16
	R-1-(4-chlorophenyl)ethan-1-ammonium	RClMBA	3.2382	1.0041	12.70	7.20
	rac-1-(4-chlorophenyl)ethan-1-ammonium	RacClMBA	3.2293	1.0037	12.04	7.00
	R-1-(4-bromophenyl)ethan-1-ammonium	RBrMBA	3.2358	1.0041	12.34	6.40
Sn	5,6-difluoro-1H-benzimidazole	2FBMZ	3.1294	1.0038	13.04	1.66
	Phenylethylammonium	PEA	3.1344	1.0004	1.33	1.82
	4-(trifluoromethyl)benzylammonium	4tFmPMA	3.1345	1.0010	3.69	14.06
	3,5-difluorophenylmethylammonium	35dFPMA	3.1360	1.0022	7.68	14.06
	Butylammonium	BA	3.1443	1.0014	4.81	8.44
	2-(2-bromo-5-carboxyphenoxy)ethan-1-aminium	BrCA3	3.1446	1.0034	11.80	10.88
	Benzimidazole	BMZ	3.1461	1.0037	12.98	1.61
	2-bromophenylethylammonium	2BrPEA	3.1480	1.0010	3.45	16.01
	2-thiophenemethylammonium	2ThioMA	3.1487	1.0000	0.11	0.27
	4-fluorophenylethylammonium	4FPEA	3.1505	1.0012	3.82	3.93
	2-iodoethanammonium	2IEA	3.1530	1.0028	8.64	12.70
	4-chlorophenylmethylammonium	4ClPMA	3.1552	1.0019	6.43	7.01
	3T	3T	3.1536	1.0040	8.54	1.64
	Cyclohexanemethanammonium	CyHMA	3.1568	1.0010	3.24	4.80
	4-chloroaniline	4ClAn	3.1661	1.0030	9.91	16.40
	4-iodoaniline	4IAn	3.1755	1.0022	6.89	18.66
	Phenylmethylammonium	PMA	3.1756	1.0031	10.62	10.22
	4-fluorophenylmethylammonium	4FPMA	3.1991	1.0043	12.75	9.62
	1-methyl-hexylammonium	1M-HA	3.2004	1.0029	9.49	6.05
	Ethanolammonium	2OHEA	3.1870	1.0008	2.28	10.03
	2-tetrahydrofurfurylammonium	2THFMA	3.1470	1.0023	8.12	7.92
	Tranexamic acid	4AMCyHCOOH	3.1382	1.0003	0.91	0
	4-iodobutylammonium	4IBA	3.1578	1.0015	5.42	16.00
	Cysteammonium	HSEA	3.2400	1.0127	18.32	2.87
	3-chlorophenylmethylammonium	3ClPMA	3.1360	1.0025	8.68	12.77
	S-1-(4-bromophenyl)ethan-1-ammonium	SBrMBA	3.2346	1.0065	10.87	6.14

B-cation	LA-cation	LA-Abbreviation	$\bar{d}$ (Å)	$\langle\lambda\rangle$	σ_θ^2 (deg^2)	dihedral angle (deg)
	TT	TT	3.1543	1.0044	10.57	0.46
	Thiophene-2-ethylammonium	TEA	3.1318	1.0009	2.46	3.85
	1-(2-aminoethyl) pyridinium	NEAPY	3.1660	1.0120	29.59	11.70
	1,5-diamino-3-oxapentane	2EAOEA	3.1663	1.0061	14.73	9.64
	2-pyridylethylammonium	2PYEA	3.1700	1.0140	37.52	1.37
	4-aminobutyric acid	GABA	3.2008	1.0046	13.81	4.85
	4-(2-aminoethyl) pyridinium	4PYEA	3.1508	1.0063	17.83	2.06
	4-(aminomethyl)piperidinium	4AMPid	3.1872	1.0070	16.40	7.33
	4-aminomethylpyridinium	4AMPY	3.1580	1.0000	0.004	0
	4Tm	4Tm	3.1481	1.0051	10.42	2.07
	1,5-naphthalenediammonium	15DNEA	3.1695	1.0017	5.39	7.08
	Dodecaneammonium	C12	3.1430	1.0019	6.77	14.89
	Hexadecylammonium	C16	3.1246	1.0013	3.55	14.09
	1,5-diaminopentane	DAA	3.1448	1.0010	2.46	13.08
	Hexylammonium	HA	3.1528	1.0016	5.47	7.25
	N-(2-aminoethyl)piperidinium	NEAPid	3.2058	1.0110	26.30	0.68
	R-alpha-methylbenzylammonium	RMBA	3.2609	1.0115	13.03	5.92
	S-alpha-methylbenzylammonium	SMBA	3.2674	1.0112	10.38	5.99
	rac-alpha-methylbenzylammonium	RacMBA	3.2674	1.0113	10.35	5.99
Ge	Butylammonium	BA	3.0307	1.0061	11.78	11.50
	Amylammonium	AA	3.0442	1.0141	26.67	12.73
	Ethanolammonium	2OHEA	3.1456	1.0327	42.86	1.30
	2-iodoethanammonium	2IEA	3.0469	1.0171	33.85	11.62
	4-bromoaniline	4BrAn	3.0834	1.0192	36.70	15.14
	4-chloroaniline	4ClAn	3.0666	1.0189	36.97	14.24
	4-chloro-N-methylaniline	Nm4ClAn	3.0916	1.0155	21.98	4.38
	4-bromo-N-methylaniline	Nm4BrAn	3.1012	1.0169	23.05	5.03
	4-fluorophenylmethylammonium	4FPMA	2.9992	1.0008	2.81	11.35
	4-chlorophenylmethylammonium	4ClPMA	2.9855	1.0008	2.78	10.11
	4-bromophenylmethylammonium	4BrPMA	2.9779	1.0009	3.19	10.41
	4-iodophenylmethylammonium	4IPMA	2.9789	1.0019	6.55	11.08
	4-isopropylbenzylammonium	4iPPMA	3.0269	1.0028	7.91	13.30
	3-fluorophenylmethylammonium	3FPMA	2.9830	1.0009	3.05	10.88
	3-chlorophenylmethylammonium	3ClPMA	2.9940	1.0009	2.94	10.89
	4-(trifluoromethyl)benzylammonium	4tFmPMA	3.0145	1.0083	16.54	12.60
	Tyrammonium	4OHPEA	3.0680	1.0146	21.24	3.52
	4-fluorophenylethylammonium	4FPEA	3.0771	1.0143	20.38	3.56
	2-fluoro-4-bromo-	2F4BrPMA	3.0165	1.0011	3.36	14.16

B-cation	LA-cation	LA-Abbreviation	$\bar{d}$ (Å)	$\langle\lambda\rangle$	σ_θ^2 (deg^2)	dihedral angle (deg)
	phenylmethylammonium					
	S-1-(4-bromophenyl)ethan-1-ammonium	SBrMBA	3.1617	1.0319	48.71	3.93
	Benzimidazole	BMZ	3.0795	1.0204	38.24	0.60
	2-pyridylethylammonium	2PYEA	3.1942	1.0433	58.53	9.92
	4-aminomethylpyridinium	4AMPY	3.0882	1.0123	27.07	6.55
	3-aminomethylpyridinium	3AMPY	3.0699	1.0198	36.34	7.94
	1-isopropylpiperidin-4-ammonium	4aiPPid	3.2578	1.0617	90.54	13.19
	1-ethyl-4-piperidinammonium	4aEtPid	3.1721	1.0408	71.43	9.09
	3-dimethylaminopropylammonium	NNdmDPA	3.1248	1.0260	43.07	6.94
	Phenylethylammonium	PEA	3.0760	1.0129	17.99	3.45
	Morpholine	Morph	3.1439	1.0305	43.87	0
	R-1-(4-bromophenyl)ethan-1-ammonium	RBrMBA	3.1567	1.0325	49.87	3.92
	5,6-difluoro-1H-benzimidazole	2FBMZ	3.0546	1.0168	33.85	0.26

$\langle\lambda\rangle = \sum_{i=1}^{6}\left(d_i/\bar{d}\right)^2/6$, $\sigma_\theta^2 = \sum_{i=1}^{12}(\theta_i - 90°)^2/11$, where d_i is the individual B−I bond length, and θ_i is the I−B−I bond angle. $\langle\lambda\rangle$ is dimensionless by definition.

Table S3. The temperature-dependent PL linewidths in representative Pb-based structures used for exciton-phonon coupling strength fitting.

Temperature (K)	FWHM (meV)								
	2FBMZ	4FPEA	PEA	BA	4ClAn	4FPMA	SBrMBA	2FPMA	44dFCyHMA
80	14.44	18.50	14.07	18.31	15.42	22.30	15.52	30.06	26.79
100	17.10	22.07	16.44	20.76	18.88	26.36	20.99	33.04	31.71
120	19.85	23.89	20.03	23.78	23.07	31.03	23.84	36.96	36.97
140	22.47	26.39	23.80	26.85	28.74	36.82	30.49	41.95	43.43
160	25.98	27.16	27.60	30.29	34.42	43.97	36.74	48.90	50.58
180	30.79	32.91	31.42	35.21	40.42	49.72	54.23	55.93	60.30
200	35.11	34.76	35.87	40.06	45.58	61.46	66.06	67.53	68.76
220	37.55	43.28	40.20	46.68	50.86	69.51	79.80	92.00	81.06
240	41.57	43.44	42.18	54.56	58.68	96.37	88.02	96.62	93.10
260	47.87	49.05	46.63	62.67	67.80	91.06	89.35	105.84	104.53
280	53.01	59.75	51.51	60.87	78.91	101.71	95.07	108.89	116.61
300	60.37	63.89	55.15	77.10	92.10	112.69	106.44	126.23	126.88

Table S4. The temperature-dependent PL linewidths in representative Sn-based structures used for exciton-phonon coupling strength fitting.

Temperature (K)	FWHM (meV)					
	2FBMZ	PEA	BA	4FPEA	4ClAn	4FPMA
80	26.06	33.59	25.13	28.89	28.53	63.14
100	28.17	38.75	30.12	32.92	36.74	64.73
120	31.51	43.93	38.91	38.90	43.61	61.57
140	39.25	57.94	45.83	48.24	54.35	66.21
160	49.60	65.37	54.40	57.49	64.92	75.77
180	54.12	70.39	60.00	68.86	74.91	93.55
200	65.65	77.91	71.52	79.50	85.88	114.69
220	71.08	87.66	81.30	87.82	100.67	153.61
240	75.48	94.89	89.50	91.65	117.69	166.78
260	86.86	102.24	92.07	98.93	137.22	212.36
280	98.35	114.55	101.20	105.33	161.94	245.21
300	107.48	123.44	115.44	113.18	182.47	307.73

Table S5. Fitting parameters of exciton-phonon coupling strength ($\boldsymbol{\gamma_{LO}}$) extracted from the temperature dependence of PL linewidths in representative Pb- and Sn-based structures listed in Table S3-S4.

B	LA	$\bar{d}$ (Å)	γ_{LO}(meV)	E_{LO}(meV)	Γ_0(meV)	Reduced Chi-Sqr*	R-Square (COD)	Adj. R-Square*
Pb	2FBMZ	3.1677	104.73 ± 14.46	31.26 ± 2.32	13.88 ± 1.83	1.18E-05	0.99511	0.98558
	4FPEA	3.1752	104.68 ± 14.45	31.26 ± 2.32	17.14 ± 1.83		0.97822	
	PEA	3.1763	97.22 ± 13.70	31.26 ± 2.32	15.21 ± 1.82		0.99061	
	BA	3.1882	133.69 ± 17.50	31.26 ± 2.32	15.95 ± 1.90		0.98303	
	4ClAn	3.1974	172.40 ± 21.76	31.26 ± 2.32	13.38 ± 2.01		0.98981	
	4FPMA	3.2115	224.08 ± 27.62	31.26 ± 2.32	19.20 ± 2.19		0.97937	
	BrMBA	3.2305	228.51 ± 28.12	31.26 ± 2.32	15.32 ± 2.20		0.97007	
	2FPMA	3.2434	238.85 ± 29.31	31.26 ± 2.32	24.80 ± 2.24		0.98111	
	44dFCyHMA	3.2482	243.10 ± 29.80	31.26 ± 2.32	23.72 ± 2.26		0.99899	
Sn	2FBMZ	3.1294	218.32 ± 27.53	33.41 ± 2.42	24.90 ± 1.97	1.07E-05	0.9937	0.9913
	PEA	3.1344	232.95 ± 29.23	33.41 ± 2.42	37.23 ± 2.01		0.98633	
	BA	3.1443	264.01 ± 38.45	33.41 ± 2.42	26.19 ± 2.13		0.99181	
	4FPEA	3.1505	319.94 ± 53.56	33.41 ± 2.42	26.30 ± 2.33		0.99939	
	4ClAn	3.1661	396.99 ± 48.62	33.41 ± 2.42	24.86 ± 2.62		0.992	
	4FPMA	3.1991	4283.42 ± 865.52	75.85 ± 4.89	61.24 ± 3.37	3.96E-05	0.99526	0.99421

The fitting model is $\Gamma(T) = \Gamma_0 + \gamma_{LO}/\left(e^{E_{LO}/k_B T} - 1\right)$, where Γ_0 is inhomogeneous broadening due to structural disorder, and Γ_{LO} is the homogeneous broadening arising from longitudinal optical (LO) phonon scattering, characterized by γ_{LO} and average phonon energy (E_{LO}).
*A global fitting approach was used to extract the γ_{LO} for all Pb-based perovskites and Sn-based perovskites (except for $(4FPMA)_2SnI_4$, which was fitted individually).
COD is equivalent to R-square, which stands for coefficient of determination, indicating how well the regression model fits the observed data. Since we used a global fitting approach, R-square (COD) for each data can help proving the reliability of the global fitting model.

Table S6. Summary of the fitted exciton diffusion coefficients of $(PEA)_2PbI_4$, $(2FBMZ)_2PbI_4$, $(PEA)_2SnI_4$ and $(2FBMZ)_2SnI_4$ under different pump fluences at 80 K. For each condition, at least three set of parallel measurement data are listed.

$(PEA)_2PbI_4$		$(2FBMZ)_2PbI_4$		$(PEA)_2SnI_4$		$(2FBMZ)_2SnI_4$	
Pump Fluence (μJ/cm²)	*D* (cm²/s)	Pump Fluence (μJ/cm²)	*D* (cm²/s)	Pump Fluence (μJ/cm²)	*D* (cm²/s)	Pump Fluence (μJ/cm²)	*D* (cm²/s)
0.015	1.78 ± 0.02	0.053	4.54 ± 0.07	0.230	0.12 ± 0.03	0.481	1.61 ± 0.40
	1.68 ± 0.02		4.79 ± 0.06		0.14 ± 0.04		1.17 ± 0.11
	1.76 ± 0.02		5.10 ± 0.06		0.08 ± 0.03		1.25 ± 0.22
	1.62 ± 0.02		4.54 ± 0.06				1.43 ± 0.19
			4.35 ± 0.09				
0.062	2.01 ± 0.02	0.224	5.18 ± 0.05	0.890	0.25 ± 0.02	1.858	1.37 ± 0.12
	1.89 ± 0.01		4.95 ± 0.04		0.25 ± 0.03		0.89 ± 0.03
	1.72 ± 0.02		5.65 ± 0.05		0.28 ± 0.02		0.83 ± 0.08
	1.86 ± 0.01		5.14 ± 0.03		0.34 ± 0.03		1.10 ± 0.06
	2.02 ± 0.02				0.31 ± 0.02		
0.136	2.11 ± 0.01	0.426	5.65 ± 0.04	1.459	0.44 ± 0.03	4.257	1.05 ± 0.06
	2.02 ± 0.01		5.38 ± 0.05		0.31 ± 0.01		1.59 ± 0.02
	1.92 ± 0.01		5.46 ± 0.06		0.52 ± 0.03		1.40 ± 0.12
	2.02 ± 0.02		5.47 ± 0.08		0.69 ± 0.03		1.56 ± 0.10
					0.39 ± 0.02		
					0.46 ± 0.02		
average	1.88	average	5.09	average	0.33	average	1.27

Table S7. The excitation-density dependence of exciton diffusion coefficient of $(PEA)_2PbI_4$, $(2FBMZ)_2PbI_4$, $(PEA)_2SnI_4$ and $(2FBMZ)_2SnI_4$ at 80 K. The *D* under different pump fluences is averaged from Table S6.

Sample	Pump Fluence (μJ/cm^2)	*D* (cm^2/s)
$(PEA)_2PbI_4$	0.015	1.71
	0.062	1.90
	0.136	2.02
$(2FBMZ)_2PbI_4$	0.053	4.66
	0.224	5.23
	0.426	5.48
$(PEA)_2SnI_4$	0.230	0.11
	0.890	0.29
	1.459	0.47
$(2FBMZ)_2SnI_4$	0.481	1.36
	1.858	1.05
	4.257	1.40

Table S8. Calculated carrier effective masses for $(2FBMZ)_2PbI_4$, $(PEA)_2PbI_4$, $(2FBMZ)_2SnI_4$, and $(PEA)_2SnI_4$.

Structures	K-path	Electron effective mass	Hole effective mass
$(2FBMZ)_2PbI_4$	$V_2 \rightarrow D_2$	0.336 m_0	0.206 m_0
$(PEA)_2PbI_4$	$\Gamma \rightarrow V_2$	0.256 m_0	0.281 m_0
$(2FBMZ)_2SnI_4$	$V_2 \rightarrow D_2$	0.268 m_0	0.128 m_0
$(PEA)_2SnI_4$	$\Gamma \rightarrow X$	0.152 m_0	0.144 m_0

K-path is the band direction used to calculate the effective mass, m_0 is the free electron mass. Due to structural differences, the K-paths for each structure are not exactly the same. However, the K-paths reported in this table are along the in-plane direction of the inorganic layer.
Type or paste caption here. Create a page break and paste in the Table above the caption.

Table S9. Calculated dielectric constants for $(2FBMZ)_2PbI_4$, $(PEA)_2PbI_4$, $(2FBMZ)_2SnI_4$, and $(PEA)_2SnI_4$.

Structures	High frequency dielectric constant (in-plane, contributed by electron)	Static dielectric constant (in-plane, sum of electron and ion)
$(2FBMZ)_2PbI_4$	4.71	13.14
$(PEA)_2PbI_4$	3.93	8.00
$(2FBMZ)_2SnI_4$	8.67	50.85
$(PEA)_2SnI_4$	5.04	13.20

Table S10. The structural parameters and lasing results of $(LA)_2SnI_4$ at 80 K.

LA-Abbreviation	$\bar{d}$ (Å)	$\langle\lambda\rangle$	σ_θ^2	dihedral angle (deg)	Asymmetry factor	Lasing or not--evidence
2FBMZ	3.1294	1.0038	13.04	1.66	1.40	Yes—Fig. S25
PEA	3.1344	1.0004	1.33	1.82	1.62	Yes—Fig S23A
4tFmPMA	3.1345	1.0010	3.69	14.06	1.84	No—Fig S23D
35dFPMA	3.1360	1.0022	7.68	14.06	1.95	No—Fig S23D
BA	3.1443	1.0014	4.81	8.44	2.00	Yes—Fig S23A
BMZ	3.1461	1.0037	12.98	1.61	1.57	Yes—Fig S23A
2BrPEA	3.1480	1.0010	3.45	16.01	1.98	No—Fig S23D
2ThioMA	3.1487	1.0000	0.11	0.27	1.59	Yes—Fig S23A
4FPEA	3.1505	1.0012	3.82	3.93	1.51	Yes—Fig S23A
2IEA	3.1530	1.0028	8.64	12.70	2.35	No—Fig S23C
4ClPMA	3.1552	1.0019	6.43	7.01	1.63	Yes—Fig S23A
CyHMA	3.1568	1.0010	3.24	4.80	1.83	Yes—Fig S23A
2FPEA	3.1590	NA[1]	NA[1]	0.10	1.81	Yes—Fig S23A
4ClAn	3.1661	1.0030	9.91	16.40	2.33	No—Fig S23B
4IAn	3.1755	1.0022	6.89	18.66	2.33	No—Fig S23C
PMA	3.1756	1.0031	10.62	10.22	2.08	Yes—Fig S23A
4FPMA	3.1991	1.0043	12.75	9.62	4.27	No—Fig S23B
1M-HA	3.2004	1.0029	9.49	6.05	3.28	No—Fig S23B
HSEA	3.2400	1.0127	18.32	2.87	6.23	No—Fig S23B
3ClPMA	3.1360	1.0025	8.68	12.77	1.72	No—Fig S23D
SBrMBA	3.2346	1.0065	10.87	6.14	STE[2]	No—Fig S23B
TEA	3.1318	1.0009	2.46	3.85	1.41	Yes—Fig S23A
2PYEA	3.1700	1.0140	37.52	1.37	2.97	No—Fig S23C
4AMPid	3.1872	1.0070	16.40	7.33	2.48	No—Fig S23C
15DNEA	3.1695	1.0017	5.39	7.08	2.52	Yes—Fig S23A
C16	3.1246	1.0013	3.55	14.09	1.94	No—Fig S23C
DAA	3.1448	1.0010	2.46	13.08	3.29	No—Fig S23C
HA	3.1528	1.0016	5.47	7.25	2.01	Yes—Fig S23A
NEAPid	3.2058	1.0110	26.30	0.68	4.22	No—Fig S23B
RMBA	3.2609	1.0115	13.03	5.92	STE[2]	No—Fig S23B
SMBA	3.2674	1.0112	10.38	5.99	STE[2]	No—Fig S23B
racMBA	3.2674	1.0113	10.35	5.99	STE[2]	No—Fig S23B
BrCA3	3.1446	1.0034	11.80	10.88		Yes—ref 32
3T	3.1536	1.0040	8.54	1.64		Yes—ref 33

[1] due to the disorder of inorganic lattice in $(2FPEA)_2SnI_4$ structure, the octahedral distortion parameters cannot be determined.

[2] represents self-trapped exciton (STE) emission with broad PL spectra, and the asymmetry factor cannot be determined.

Data 1 in separate zip files

Data S1. Crystallographic data (CIF files) and validation reports (checkCIF) for the crystal structures reported in this study. This ZIP archive contains the CIF files and corresponding checkCIF validation reports for each of the 20 crystal structures. The same data have been deposited in the Cambridge Crystallographic Data Centre (CCDC deposition numbers 2474241–2474260) and are freely available via www.ccdc.cam.ac.uk/data_request/cif upon publication.
