# Supplementary material for: Unlock lasing in two-dimensional metal halide perovskites by tuning ns2 lone pairs
Source: Sci Adv. 2026 Aug 5;12(32):eaee5631. doi: 10.1126/sciadv.aee5631 (PMC13440396; doi:10.1126/sciadv.aee5631)

checkCIF/PLATON report

Structure factors have been supplied for datablock(s) 4aiPPDGeI4

THIS REPORT IS FOR GUIDANCE ONLY. IF USED AS PART OF A REVIEW PROCEDURE FOR PUBLICATION, IT SHOULD NOT REPLACE THE EXPERTISE OF AN EXPERIENCED CRYSTALLOGRAPHIC REFEREE.

No syntax errors found. CIF dictionary Interpreting this report

Datablock: 4aiPPDGeI4

Bond precision: C-C = 0.0052 A Wavelength=0.71073

Cell:	a=21.5484(4)	b=12.6382(2)	c=13.5262(3)
	alpha=90	beta=103.309(2)	gamma=90
Temperature:	290 K		

	Calculated	Reported
Volume	3584.70(12)	3584.70(12)
Space group	C 2/c	C 1 2/c 1
Hall group	-C 2yc	-C 2yc
Moiety formula	C8 H20 N2, Ge I3, I	Ge I3, I, C8 H20 N2
Sum formula	C8 H20 Ge I4 N2	C8 H20 Ge I4 N2
Mr	724.47	724.45
Dx,g cm-3	2.685	2.685
Z	8	8
Mu (mm-1)	8.580	8.580
F000	2608.0	2608.0
F000’	2595.08	
h,k,lmax		25,15,16
Nref		3144
Tmin,Tmax	0.221,0.431	0.608,1.000
Tmin’	0.094	

Correction method= # Reported T Limits: Tmin=0.608 Tmax=1.000
AbsCorr = MULTI-SCAN

Data completeness= Theta(max)= 25.000

R(reflections)= 0.0173(2930) wR2(reflections)= 0.0398(3144)

S = 1.097 Npar= 139

The following ALERTS were generated. Each ALERT has the format
test-name_ALERT_alert-type_alert-level.
Click on the hyperlinks for more details of the test.

Alert level C

```
PLAT042_ALERT_1_C Calc. and Reported MoietyFormula Strings  Differ     Please Check
              Calc: C8 H20 N2, Ge I3, I
              Rep.: Ge I3, I, C8 H20 N2
PLAT934_ALERT_3_C Number of (Iobs-Icalc)/Sigma(W) > 10 Outliers ..          1 Check
                6  0  0,
```

Alert level G

```
PLAT007_ALERT_5_G Number of Unrefined Donor-H Atoms .............          4 Report
              H6    H7A   H7B   H7C
PLAT012_ALERT_1_G No       _shelx_res_checksum Found in CIF ......     Please Check
PLAT093_ALERT_1_G No s.u.'s on H-positions, Refinement Reported as      mixed Check
PLAT432_ALERT_2_G Short Inter X...Y Contact  I5       ..Ge2      .       3.32 Ang.
                                                    x,y,z  =      1_555 Check
PLAT909_ALERT_3_G Percentage of I>2sig(I) Data at Theta(Max) Still        86% Note
PLAT910_ALERT_3_G Missing # of FCF Reflection(s) Below Theta(Min).          3 Note
                1  1  0,   2  0  0,  -1  1  1,
PLAT933_ALERT_2_G Number of HKL-OMIT Records in Embedded .res File          1 Note
               -1  1  1,
PLAT961_ALERT_5_G Dataset Contains no Negative Intensities .......     Please Check
PLAT967_ALERT_5_G Note: Two-Theta Cutoff Value in Embedded .res ..       50.0 Degree
PLAT978_ALERT_2_G Number C-C Bonds with Positive Residual Density.          1 Info
```

0 **ALERT level A** = Most likely a serious problem - resolve or explain
0 **ALERT level B** = A potentially serious problem, consider carefully
2 **ALERT level C** = Check. Ensure it is not caused by an omission or oversight
10 **ALERT level G** = General information/check it is not something unexpected

3 ALERT type 1 CIF construction/syntax error, inconsistent or missing data
3 ALERT type 2 Indicator that the structure model may be wrong or deficient
3 ALERT type 3 Indicator that the structure quality may be low
0 ALERT type 4 Improvement, methodology, query or suggestion
3 ALERT type 5 Informative message, check

It is advisable to attempt to resolve as many as possible of the alerts in all categories. Often the minor alerts point to easily fixed oversights, errors and omissions in your CIF or refinement strategy, so attention to these fine details can be worthwhile. In order to resolve some of the more serious problems it may be necessary to carry out additional measurements or structure refinements. However, the purpose of your study may justify the reported deviations and the more serious of these should normally be commented upon in the discussion or experimental section of a paper or in the "special_details" fields of the CIF. checkCIF was carefully designed to identify outliers and unusual parameters, but every test has its limitations and alerts that are not important in a particular case may appear. Conversely, the absence of alerts does not guarantee there are no aspects of the results needing attention. It is up to the individual to critically assess their own results and, if necessary, seek expert advice.

Publication of your CIF in IUCr journals

A basic structural check has been run on your CIF. These basic checks will be run on all CIFs submitted for publication in IUCr journals (*Acta Crystallographica*, *Journal of Applied Crystallography*, *Journal of Synchrotron Radiation*); however, if you intend to submit to *Acta Crystallographica Section C* or *E* or *IUCrData*, you should make sure that full publication checks are run on the final version of your CIF prior to submission.

Publication of your CIF in other journals

Please refer to the *Notes for Authors* of the relevant journal for any special instructions relating to CIF submission.

PLATON version of 14/11/2023; check.def file version of 14/09/2023

Datablock 4aiPPDGeI4 - ellipsoid plot

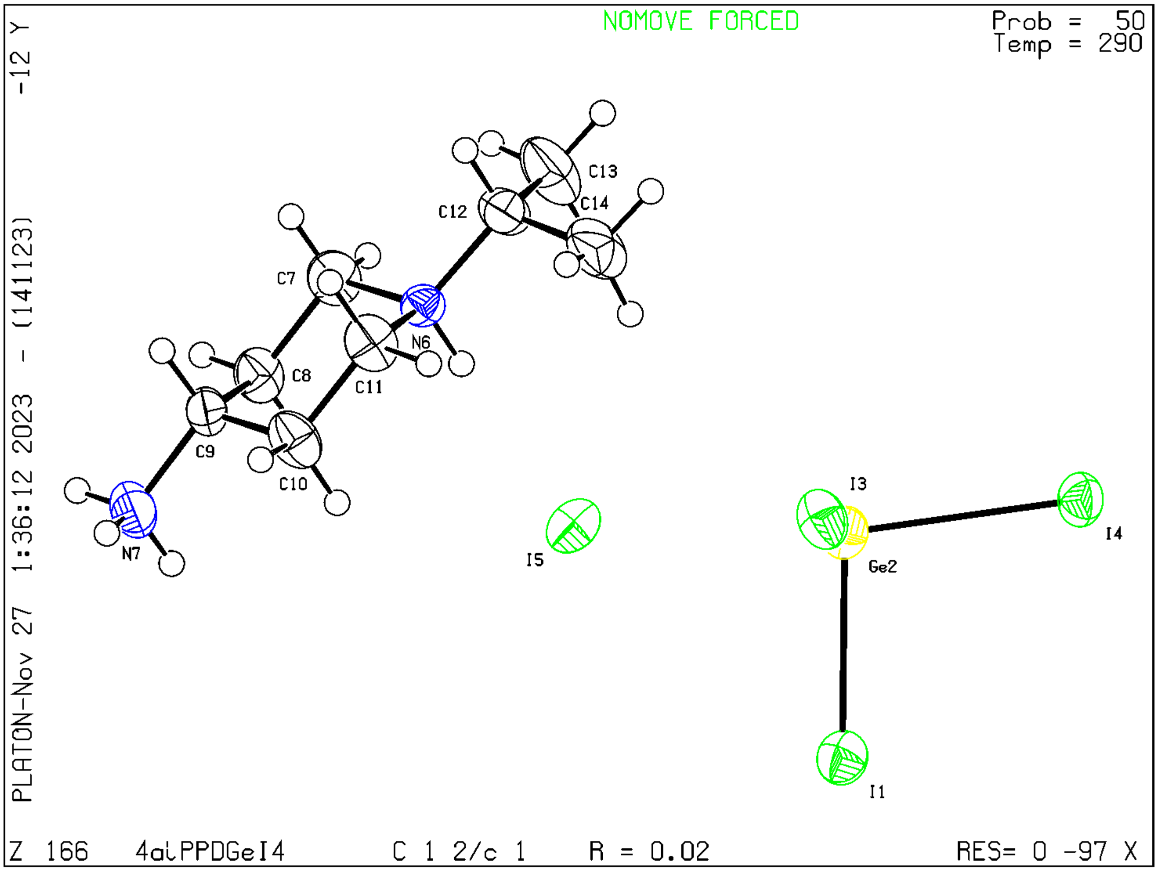

Supplement: Supplementary file 2 [file sciadv.aee5631_data_s1.zip › Ge-4aiPPD-checkcif.pdf]
