# Supplementary material for: Unlock lasing in two-dimensional metal halide perovskites by tuning ns2 lone pairs
Source: Sci Adv. 2026 Aug 5;12(32):eaee5631. doi: 10.1126/sciadv.aee5631 (PMC13440396; doi:10.1126/sciadv.aee5631)

checkCIF/PLATON report

Structure factors have been supplied for datablock(s) ge-4bran-abscorr

THIS REPORT IS FOR GUIDANCE ONLY. IF USED AS PART OF A REVIEW PROCEDURE FOR PUBLICATION, IT SHOULD NOT REPLACE THE EXPERTISE OF AN EXPERIENCED CRYSTALLOGRAPHIC REFEREE.

No syntax errors found. CIF dictionary Interpreting this report

Datablock: ge-4bran-abscorr

```
Bond precision:   C-C = 0.0043 A          Wavelength=0.71073

Cell:             a=30.6357(14)      b=7.9257(3)        c=9.0659(3)
                  alpha=90           beta=96.793(4)     gamma=90
Temperature:      290 K

                Calculated                    Reported
Volume          2185.83(15)                   2185.83(15)
Space group     P 21/c                        P 1 21/c 1
Hall group      -P 2ybc                       -P 2ybc
Moiety formula  2(C6 H7 Br N), Ge I4          Ge I4, 2(C6 H7 Br N)
Sum formula     C12 H14 Br2 Ge I4 N2          C12 H14 Br2 Ge I4 N2
Mr              926.26                        926.26
Dx,g cm-3       2.815                         2.815
Z               4                             4
Mu (mm-1)       10.703                        10.703
F000            1656.0                        1656.0
F000'           1647.65
h,k,lmax        42,10,12                      40,10,12
Nref            6081                          5500
Tmin,Tmax       0.260,0.592                   0.661,1.000
Tmin'           0.170

Correction method= # Reported T Limits: Tmin=0.661 Tmax=1.000
AbsCorr = GAUSSIAN

Data completeness= 0.904           Theta(max)= 29.470

R(reflections)= 0.0344( 4482)                         wR2(reflections)=
                                                      0.0855( 5500)
S = 1.060                    Npar= 169
```

The following ALERTS were generated. Each ALERT has the format
test-name_ALERT_alert-type_alert-level.
Click on the hyperlinks for more details of the test.

Alert level B

```
PLAT110_ALERT_2_B ADDSYM Detects Potential Lattice Translation ...          ? Check
PLAT112_ALERT_2_B ADDSYM Detects New (Pseudo) Symm. Elem         B        100 %Fit
PLAT113_ALERT_2_B ADDSYM Suggests Possible Pseudo/New  Space Group      P21/c Check
              Check Model Parameter Symmmetry for Reflection Data Support
```

Alert level C

```
PLAT042_ALERT_1_C Calc. and Reported MoietyFormula Strings  Differ     Please Check
PLAT906_ALERT_3_C Large K Value in the Analysis of Variance ......      5.847 Check
PLAT971_ALERT_2_C Check Calcd Resid. Dens.  0.88Ang From Ge1             1.64 eA-3
```

Alert level G

```
PLAT007_ALERT_5_G Number of Unrefined Donor-H Atoms .............           6 Report
PLAT012_ALERT_1_G No      _shelx_res_checksum Found in CIF ......     Please Check
PLAT083_ALERT_2_G SHELXL Second Parameter in WGHT  Unusually Large       6.52 Why ?
PLAT116_ALERT_2_G ADDSYM Included (Pseudo) Lattice Translation ...     Please Check
PLAT432_ALERT_2_G Short Inter X...Y Contact  I001     ..Ge1      .       3.45 Ang.
                                   x,1/2-y,-1/2+z  =      4_565 Check
PLAT432_ALERT_2_G Short Inter X...Y Contact  I002     ..Ge1      .       3.44 Ang.
                                   x,3/2-y,-1/2+z  =      4_575 Check
PLAT720_ALERT_4_G Number of Unusual/Non-Standard Labels .........          34 Note
PLAT910_ALERT_3_G Missing # of FCF Reflection(s) Below Theta(Min).          2 Note
PLAT912_ALERT_4_G Missing # of FCF Reflections Above STh/L=  0.600        563 Note
PLAT950_ALERT_5_G Calculated (ThMax) and CIF-Reported Hmax Differ           2 Units
PLAT956_ALERT_1_G Calculated (ThMax) and Actual (FCF) Hmax Differ           2 Units
PLAT978_ALERT_2_G Number C-C Bonds with Positive Residual Density.          1 Info
```

```
   0 ALERT level A = Most likely a serious problem - resolve or explain
   3 ALERT level B = A potentially serious problem, consider carefully
   3 ALERT level C = Check. Ensure it is not caused by an omission or oversight
  12 ALERT level G = General information/check it is not something unexpected

   3 ALERT type 1 CIF construction/syntax error, inconsistent or missing data
   9 ALERT type 2 Indicator that the structure model may be wrong or deficient
   2 ALERT type 3 Indicator that the structure quality may be low
   2 ALERT type 4 Improvement, methodology, query or suggestion
   2 ALERT type 5 Informative message, check
```

It is advisable to attempt to resolve as many as possible of the alerts in all categories. Often the minor alerts point to easily fixed oversights, errors and omissions in your CIF or refinement strategy, so attention to these fine details can be worthwhile. In order to resolve some of the more serious problems it may be necessary to carry out additional measurements or structure refinements. However, the purpose of your study may justify the reported deviations and the more serious of these should normally be commented upon in the discussion or experimental section of a paper or in the "special_details" fields of the CIF. checkCIF was carefully designed to identify outliers and unusual parameters, but every test has its limitations and alerts that are not important in a particular case may appear. Conversely, the absence of alerts does not guarantee there are no aspects of the results needing attention. It is up to the individual to critically assess their own results and, if necessary, seek expert advice.

Publication of your CIF in IUCr journals

A basic structural check has been run on your CIF. These basic checks will be run on all CIFs submitted for publication in IUCr journals (*Acta Crystallographica*, *Journal of Applied Crystallography*, *Journal of Synchrotron Radiation*); however, if you intend to submit to *Acta Crystallographica Section C* or *E* or *IUCrData*, you should make sure that full publication checks are run on the final version of your CIF prior to submission.

Publication of your CIF in other journals

Please refer to the *Notes for Authors* of the relevant journal for any special instructions relating to CIF submission.

PLATON version of 06/07/2023; check.def file version of 30/06/2023

Datablock ge-4bran-abscorr - ellipsoid plot

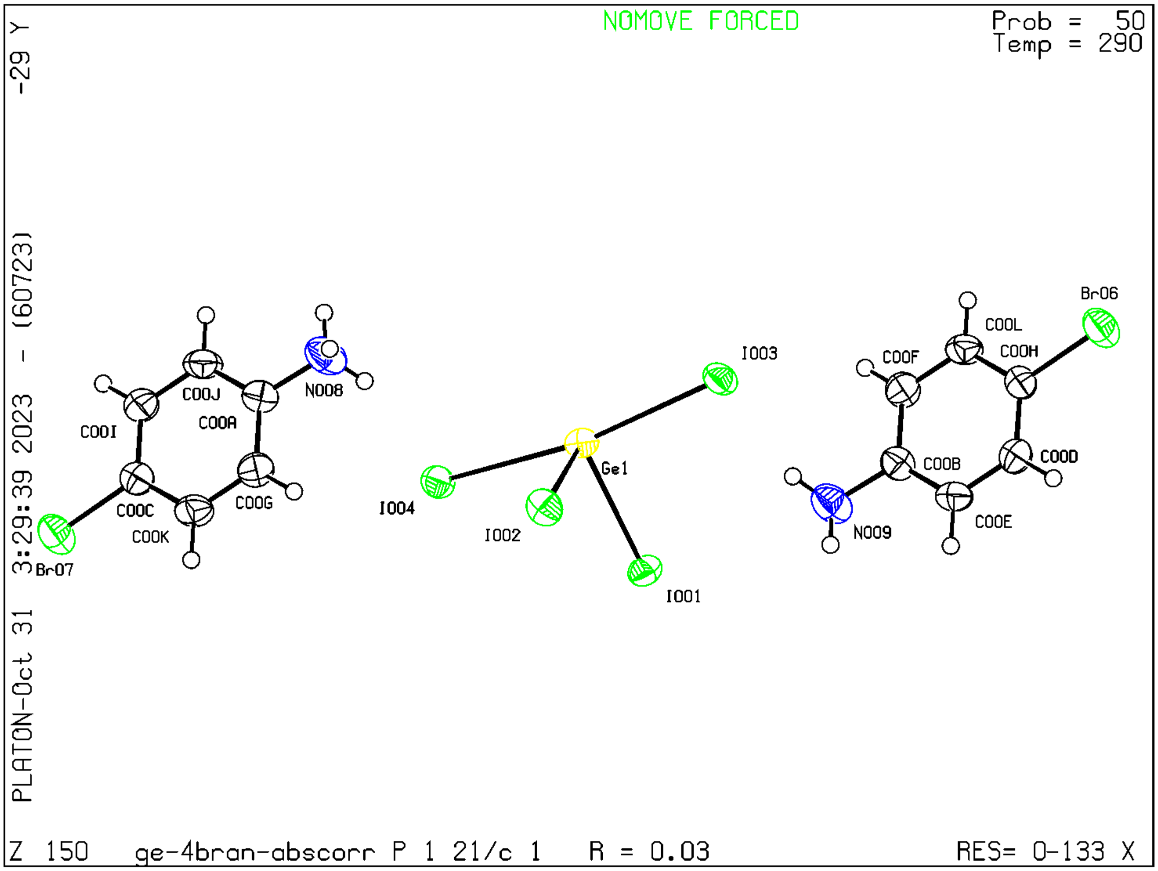

Supplement: Supplementary file 2 [file sciadv.aee5631_data_s1.zip › Ge-4BrAn-checkcif.pdf]
