# Supplementary material for: Unlock lasing in two-dimensional metal halide perovskites by tuning ns2 lone pairs
Source: Sci Adv. 2026 Aug 5;12(32):eaee5631. doi: 10.1126/sciadv.aee5631 (PMC13440396; doi:10.1126/sciadv.aee5631)

checkCIF/PLATON report

Structure factors have been supplied for datablock(s) exp_11567

THIS REPORT IS FOR GUIDANCE ONLY. IF USED AS PART OF A REVIEW PROCEDURE FOR PUBLICATION, IT SHOULD NOT REPLACE THE EXPERTISE OF AN EXPERIENCED CRYSTALLOGRAPHIC REFEREE.

No syntax errors found. CIF dictionary Interpreting this report

Datablock: exp_11567

Bond precision:	C-C = 0.0070 A	Wavelength=0.71073	
Cell:	a=30.4134(10)	b=7.8574(3)	c=8.9748(3)
	alpha=90	beta=96.751(3)	gamma=90
Temperature:	290 K		

	Calculated	Reported
Volume	2129.84(13)	2129.84(13)
Space group	P 21/c	P 1 21/c 1
Hall group	-P 2ybc	-P 2ybc
Moiety formula	2(C6 H7 Cl N), Ge I4	Ge I4, 2(C6 H7 Cl N)
Sum formula	C12 H14 Cl2 Ge I4 N2	C12 H14 Cl2 Ge I4 N2
Mr	837.36	837.34
Dx,g cm-3	2.611	2.611
Z	4	4
Mu (mm-1)	7.483	7.483
F000	1512.0	1512.0
F000'	1506.73	
h,k,lmax	42,10,12	41,10,12
Nref	5951	5352
Tmin,Tmax		0.411,1.000
Tmin'		

Correction method= # Reported T Limits: Tmin=0.411 Tmax=1.000
AbsCorr = MULTI-SCAN

Data completeness= 0.899 Theta(max)= 29.538

R(reflections)= 0.0319(4308) wR2(reflections)= 0.0755(5352)

S = 1.090 Npar= 193

The following ALERTS were generated. Each ALERT has the format
test-name_ALERT_alert-type_alert-level.
Click on the hyperlinks for more details of the test.

Alert level B

```
PLAT110_ALERT_2_B ADDSYM Detects Potential Lattice Translation ...           ? Check
PLAT112_ALERT_2_B ADDSYM Detects New (Pseudo) Symm. Elem         B        100 %Fit
PLAT113_ALERT_2_B ADDSYM Suggests Possible Pseudo/New  Space Group      P21/c Check
              Check Model Parameter Symmmetry for Reflection Data Support
PLAT196_ALERT_1_B No TEMP record and _measurement_temperature .NE.        293 Degree
PLAT934_ALERT_3_B Number of (Iobs-Icalc)/Sigma(W) > 10 Outliers ..          3 Check
```

Alert level C

```
PLAT042_ALERT_1_C Calc. and Reported MoietyFormula Strings  Differ     Please Check
PLAT053_ALERT_1_C Minimum Crystal Dimension Missing (or Error) ...     Please Check
PLAT054_ALERT_1_C Medium  Crystal Dimension Missing (or Error) ...     Please Check
PLAT055_ALERT_1_C Maximum Crystal Dimension Missing (or Error) ...     Please Check
PLAT334_ALERT_2_C Small <C-C> Benzene Dist.   C6       -C11      .       1.37 Ang.
PLAT334_ALERT_2_C Small <C-C> Benzene Dist.   C14      -C19      .       1.37 Ang.
PLAT767_ALERT_4_C INS Embedded LIST 6 Instruction Should be LIST 4     Please Check
PLAT906_ALERT_3_C Large K Value in the Analysis of Variance ......      4.117 Check
PLAT911_ALERT_3_C Missing FCF Refl Between Thmin & STh/L=    0.600          4 Report
```

Alert level G

```
PLAT007_ALERT_5_G Number of Unrefined Donor-H Atoms .............           6 Report
PLAT116_ALERT_2_G ADDSYM Included (Pseudo) Lattice Translation ...     Please Check
PLAT432_ALERT_2_G Short Inter X...Y Contact  I1       ..Ge2      .       3.41 Ang.
                                               x,1/2-y,1/2+z  =      4_566 Check
PLAT432_ALERT_2_G Short Inter X...Y Contact  I5       ..Ge2      .       3.42 Ang.
                                               x,3/2-y,1/2+z  =      4_576 Check
PLAT883_ALERT_1_G No Info/Value for _atom_sites_solution_primary .     Please Do !
PLAT910_ALERT_3_G Missing # of FCF Reflection(s) Below Theta(Min).          2 Note
PLAT912_ALERT_4_G Missing # of FCF Reflections Above STh/L=  0.600        592 Note
PLAT941_ALERT_3_G Average HKL Measurement Multiplicity ..........         2.9 Low
PLAT978_ALERT_2_G Number C-C Bonds with Positive Residual Density.          2 Info
```

0 **ALERT level A** = Most likely a serious problem - resolve or explain
5 **ALERT level B** = A potentially serious problem, consider carefully
9 **ALERT level C** = Check. Ensure it is not caused by an omission or oversight
9 **ALERT level G** = General information/check it is not something unexpected

6 ALERT type 1 CIF construction/syntax error, inconsistent or missing data
9 ALERT type 2 Indicator that the structure model may be wrong or deficient
5 ALERT type 3 Indicator that the structure quality may be low
2 ALERT type 4 Improvement, methodology, query or suggestion
1 ALERT type 5 Informative message, check

It is advisable to attempt to resolve as many as possible of the alerts in all categories. Often the minor alerts point to easily fixed oversights, errors and omissions in your CIF or refinement strategy, so attention to these fine details can be worthwhile. In order to resolve some of the more serious problems it may be necessary to carry out additional measurements or structure refinements. However, the purpose of your study may justify the reported deviations and the more serious of these should normally be commented upon in the discussion or experimental section of a paper or in the "special_details" fields of the CIF. checkCIF was carefully designed to identify outliers and unusual parameters, but every test has its limitations and alerts that are not important in a particular case may appear. Conversely, the absence of alerts does not guarantee there are no aspects of the results needing attention. It is up to the individual to critically assess their own results and, if necessary, seek expert advice.

Publication of your CIF in IUCr journals

A basic structural check has been run on your CIF. These basic checks will be run on all CIFs submitted for publication in IUCr journals (*Acta Crystallographica*, *Journal of Applied Crystallography*, *Journal of Synchrotron Radiation*); however, if you intend to submit to *Acta Crystallographica Section C* or *E* or *IUCrData*, you should make sure that full publication checks are run on the final version of your CIF prior to submission.

Publication of your CIF in other journals

Please refer to the *Notes for Authors* of the relevant journal for any special instructions relating to CIF submission.

PLATON version of 28/11/2022; check.def file version of 28/11/2022

Datablock exp_11567 - ellipsoid plot

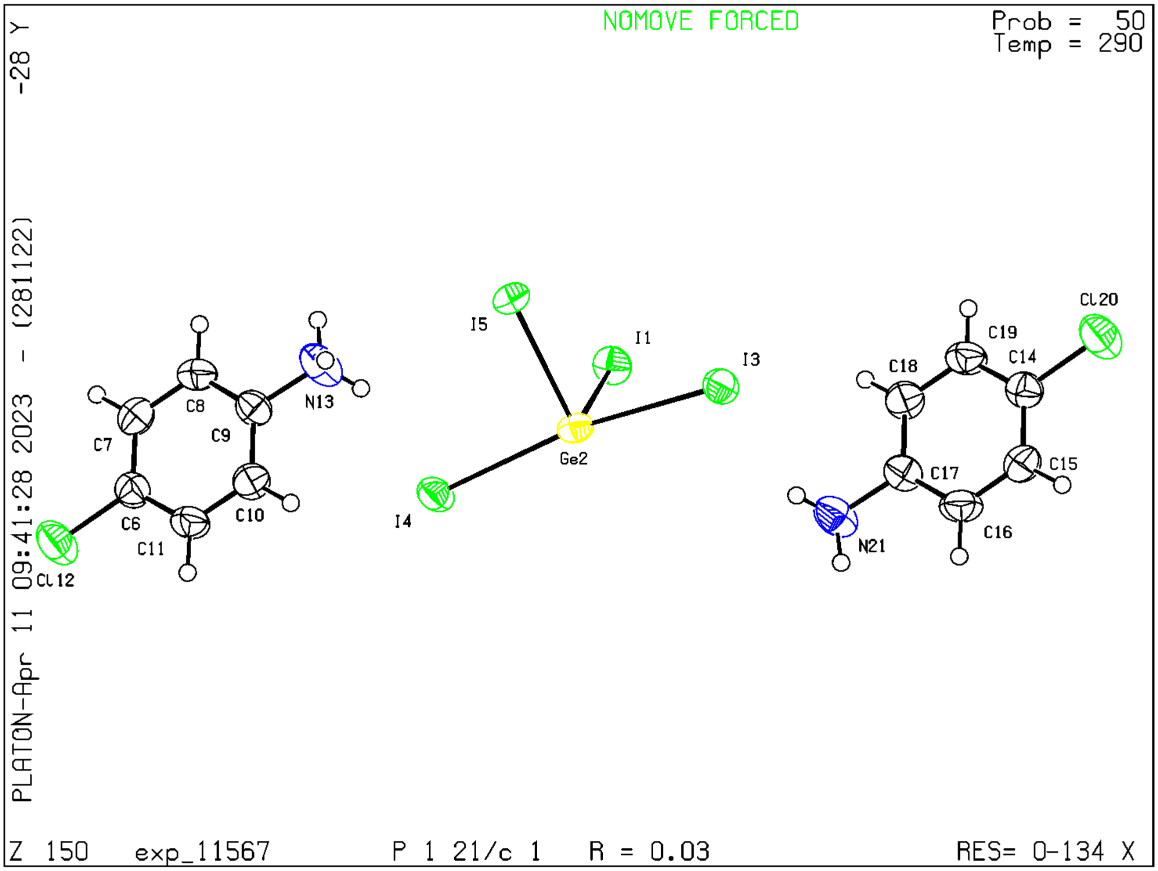

Supplement: Supplementary file 2 [file sciadv.aee5631_data_s1.zip › Ge-4ClAn-checkcif.pdf]
