# Supplementary material for: Unlock lasing in two-dimensional metal halide perovskites by tuning ns2 lone pairs
Source: Sci Adv. 2026 Aug 5;12(32):eaee5631. doi: 10.1126/sciadv.aee5631 (PMC13440396; doi:10.1126/sciadv.aee5631)

checkCIF/PLATON report

You have not supplied any structure factors. As a result the full set of tests cannot be run.

THIS REPORT IS FOR GUIDANCE ONLY. IF USED AS PART OF A REVIEW PROCEDURE FOR PUBLICATION, IT SHOULD NOT REPLACE THE EXPERTISE OF AN EXPERIENCED CRYSTALLOGRAPHIC REFEREE.

No syntax errors found.   CIF dictionary   Interpreting this report

Datablock: 4iPPMA2GeI4

```
Bond precision:   C-C = 0.0520 A          Wavelength=0.71073

Cell:               a=8.6949(3)      b=8.3483(3)      c=39.3081(18)
                    alpha=90         beta=90          gamma=90
Temperature:        290 K

                Calculated                   Reported
Volume          2853.28(19)                  2853.28(19)
Space group     P b c n                      P b c n
Hall group      -P 2n 2ab                    -P 2n 2ab
Moiety formula  Ge I4, 2(C10 H16 N)          2(C10 H16 N), Ge I4
Sum formula     C20 H32 Ge I4 N2             C20 H32 Ge I4 N2
Mr              880.69                       880.67
Dx,g cm-3       2.050                        2.050
Z               4                            4
Mu (mm-1)       5.410                        5.410
F000            1640.0                       1640.0
F000'           1633.56
h,k,lmax                                     10,9,46
Nref                                         2510
Tmin,Tmax       0.227,0.827                  0.566,1.000
Tmin'           0.171

Correction method= # Reported T Limits: Tmin=0.566 Tmax=1.000
AbsCorr = GAUSSIAN

Data completeness=                Theta(max)= 25.000

                                                        wR2(reflections)=
R(reflections)= 0.1274( 2220)                           0.2823( 2510)

S = 1.220                    Npar= 113
```

The following ALERTS were generated. Each ALERT has the format
test-name_ALERT_alert-type_alert-level.
Click on the hyperlinks for more details of the test.

Alert level B

```
PLAT342_ALERT_3_B Low Bond Precision on  C-C Bonds ...............      0.052 Ang.
PLAT412_ALERT_2_B Short Intra XH3 .. XHn     H18      ..H20A     .       1.75 Ang.
                                                  x,y,z  =      1_555 Check
```

Alert level C

```
DIFMX02_ALERT_1_C  The maximum difference density is > 0.1*ZMAX*0.75
            The relevant atom site should be identified.
PLAT042_ALERT_1_C Calc. and Reported MoietyFormula Strings  Differ     Please Check
              Calc: Ge I4, 2(C10 H16 N)
              Rep.: 2(C10 H16 N), Ge I4
PLAT082_ALERT_2_C High R1 Value ..................................       0.13 Report
PLAT084_ALERT_3_C High wR2 Value (i.e. > 0.25) ...................       0.28 Report
PLAT097_ALERT_2_C Large Reported Max.  (Positive) Residual Density       4.64 eA-3
PLAT213_ALERT_2_C Atom C11             has ADP max/min Ratio .....        3.1 oblate
PLAT213_ALERT_2_C Atom C20             has ADP max/min Ratio .....        3.1 prolat
PLAT220_ALERT_2_C NonSolvent   Resd 2  C   Ueq(max)/Ueq(min) Range        4.6 Ratio
PLAT222_ALERT_3_C NonSolvent Resd 2  H   Uiso(max)/Uiso(min) Range        5.0 Ratio
PLAT234_ALERT_4_C Large Hirshfeld Difference N2       --C11      .       0.22 Ang.
PLAT241_ALERT_2_C High   'MainMol' Ueq as Compared to Neighbors of        C17 Check
PLAT241_ALERT_2_C High   'MainMol' Ueq as Compared to Neighbors of        C18 Check
PLAT242_ALERT_2_C Low    'MainMol' Ueq as Compared to Neighbors of        C12 Check
PLAT242_ALERT_2_C Low    'MainMol' Ueq as Compared to Neighbors of        C15 Check
PLAT250_ALERT_2_C Large U3/U1 Ratio for Average U(i,j) Tensor ....        2.7 Note
PLAT260_ALERT_2_C Large Average Ueq of Residue Including        N2      0.112 Check
PLAT420_ALERT_2_C D-H Bond Without Acceptor  N2       --H2C      .     Please Check
```

Alert level G

```
PLAT003_ALERT_2_G Number of Uiso or Uij Restrained non-H Atoms ...         10 Report
PLAT004_ALERT_5_G Polymeric Structure Found with Maximum Dimension          2 Info
PLAT007_ALERT_5_G Number of Unrefined Donor-H Atoms .............           3 Report
              H2A   H2B   H2C
PLAT012_ALERT_1_G No       _shelx_res_checksum Found in CIF ......     Please Check
PLAT083_ALERT_2_G SHELXL Second Parameter in WGHT  Unusually Large     374.77 Why ?
PLAT093_ALERT_1_G No s.u.'s on H-positions, Refinement Reported as      mixed Check
PLAT177_ALERT_4_G The CIF-Embedded .res File Contains DELU Records          1 Report
PLAT343_ALERT_2_G Unusual sp?      Angle Range in Main Residue for        C18 Check
PLAT367_ALERT_2_G Long?  C(sp?)-C(sp?) Bond  C15      - C18      .       1.58 Ang.
PLAT860_ALERT_3_G Number of Least-Squares Restraints ............          23 Note
PLAT967_ALERT_5_G Note: Two-Theta Cutoff Value in Embedded .res ..       50.0 Degree
```

0 **ALERT level A** = Most likely a serious problem - resolve or explain
2 **ALERT level B** = A potentially serious problem, consider carefully
17 **ALERT level C** = Check. Ensure it is not caused by an omission or oversight
11 **ALERT level G** = General information/check it is not something unexpected

4 ALERT type 1 CIF construction/syntax error, inconsistent or missing data

```
17 ALERT type 2 Indicator that the structure model may be wrong or deficient
 4 ALERT type 3 Indicator that the structure quality may be low
 2 ALERT type 4 Improvement, methodology, query or suggestion
 3 ALERT type 5 Informative message, check
```

It is advisable to attempt to resolve as many as possible of the alerts in all categories. Often the minor alerts point to easily fixed oversights, errors and omissions in your CIF or refinement strategy, so attention to these fine details can be worthwhile. In order to resolve some of the more serious problems it may be necessary to carry out additional measurements or structure refinements. However, the purpose of your study may justify the reported deviations and the more serious of these should normally be commented upon in the discussion or experimental section of a paper or in the "special_details" fields of the CIF. checkCIF was carefully designed to identify outliers and unusual parameters, but every test has its limitations and alerts that are not important in a particular case may appear. Conversely, the absence of alerts does not guarantee there are no aspects of the results needing attention. It is up to the individual to critically assess their own results and, if necessary, seek expert advice.

Publication of your CIF in IUCr journals

A basic structural check has been run on your CIF. These basic checks will be run on all CIFs submitted for publication in IUCr journals (*Acta Crystallographica*, *Journal of Applied Crystallography*, *Journal of Synchrotron Radiation*); however, if you intend to submit to *Acta Crystallographica Section C* or *E* or *IUCrData*, you should make sure that full publication checks are run on the final version of your CIF prior to submission.

Publication of your CIF in other journals

Please refer to the *Notes for Authors* of the relevant journal for any special instructions relating to CIF submission.

PLATON version of 14/11/2023; check.def file version of 14/09/2023

Datablock 4iPPMA2GeI4 - ellipsoid plot

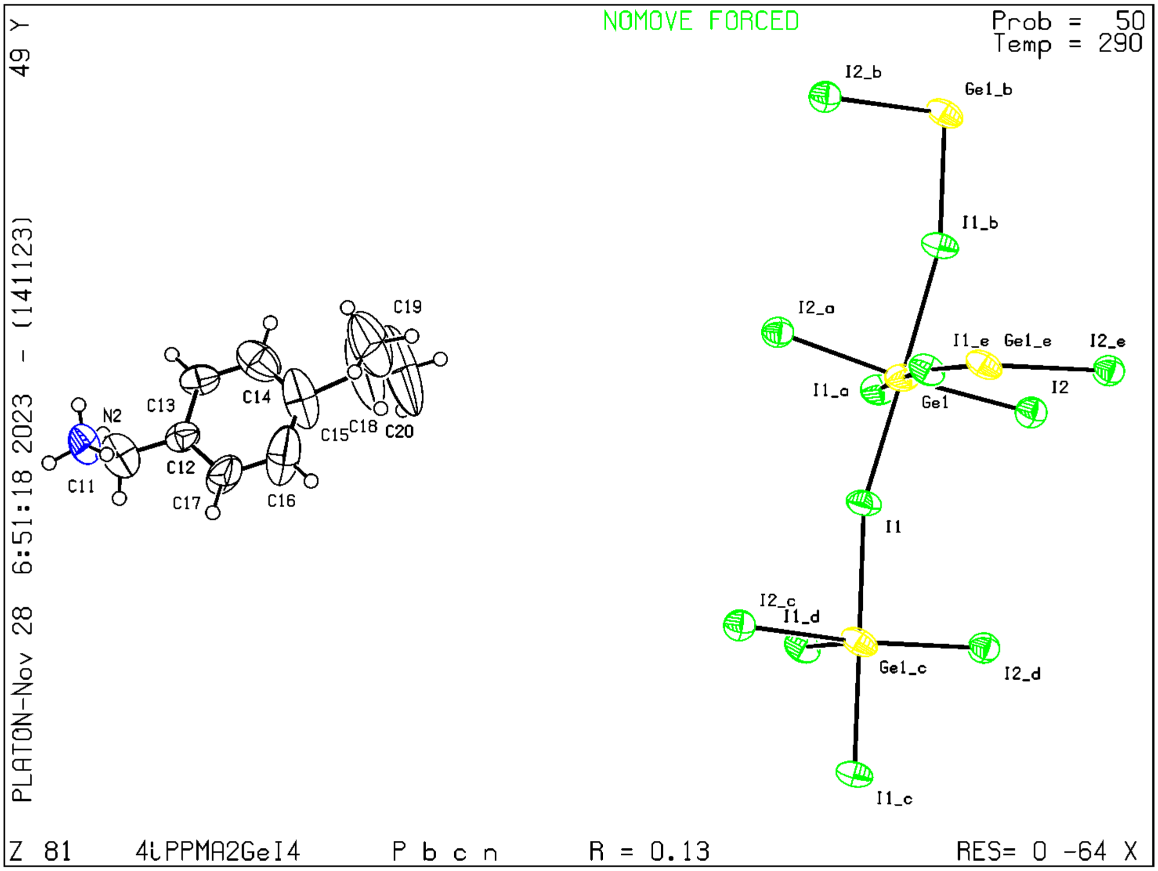

Supplement: Supplementary file 2 [file sciadv.aee5631_data_s1.zip › Ge-4iPPMA-checkcif.pdf]
