# Supplementary material for: Unlock lasing in two-dimensional metal halide perovskites by tuning ns2 lone pairs
Source: Sci Adv. 2026 Aug 5;12(32):eaee5631. doi: 10.1126/sciadv.aee5631 (PMC13440396; doi:10.1126/sciadv.aee5631)

checkCIF/PLATON report

Structure factors have been supplied for datablock(s) Nm4BrAn2GeI4

THIS REPORT IS FOR GUIDANCE ONLY. IF USED AS PART OF A REVIEW PROCEDURE FOR PUBLICATION, IT SHOULD NOT REPLACE THE EXPERTISE OF AN EXPERIENCED CRYSTALLOGRAPHIC REFEREE.

No syntax errors found. CIF dictionary Interpreting this report

Datablock: Nm4BrAn2GeI4

Bond precision:	C-C = 0.0063 A		Wavelength=0.71073
Cell:	a=8.6936(5)	b=31.681(2)	c=8.8080(4)
	alpha=90	beta=90.127(5)	gamma=90
Temperature:	290 K		

	Calculated	Reported
Volume	2425.9(2)	2425.9(2)
Space group	P 21/n	P 1 21/n 1
Hall group	-P 2yn	-P 2yn
Moiety formula	Ge I4, 2(C7 H9 Br N)	Ge I3, I, 2(C7 H9 Br N)
Sum formula	C14 H18 Br2 Ge I4 N2	C14 H18 Br2 Ge I4 N2
Mr	954.31	954.31
Dx,g cm-3	2.613	2.613
Z	4	4
Mu (mm-1)	9.648	9.648
F000	1720.0	1720.0
F000’	1711.63	
h,k,lmax	10,37,10	10,37,10
Nref	4278	3750
Tmin,Tmax	0.449,0.825	0.729,1.000
Tmin’	0.163	

Correction method= # Reported T Limits: Tmin=0.729 Tmax=1.000
AbsCorr = GAUSSIAN

Data completeness= 0.877 Theta(max)= 24.990

R(reflections)= 0.0338(2962) wR2(reflections)= 0.0812(3750)

S = 1.040 Npar= 186

The following ALERTS were generated. Each ALERT has the format
test-name_ALERT_alert-type_alert-level.
Click on the hyperlinks for more details of the test.

Alert level A

```
PLAT029_ALERT_3_A _diffrn_measured_fraction_theta_full value Low .      0.876 Why?
```

Alert level B

```
PLAT230_ALERT_2_B Hirshfeld Test Diff for    I002     --Ge8      .       36.2 s.u.
PLAT230_ALERT_2_B Hirshfeld Test Diff for    I003     --Ge8      .       33.4 s.u.
PLAT911_ALERT_3_B Missing FCF Refl Between Thmin & STh/L=    0.595        531 Report
                6  0  0,  10  0  0,   9  1  0,  10  1  0,   9  2  0,  10  2  0,
               10  3  0,   9  4  0,  10  4  0,   9  5  0,  10  5  0,   9  6  0,
               10  6  0,   8  7  0,   9  7  0,  10  7  0,   8  8  0,   9  8  0,
               10  8  0,   8  9  0,   9  9  0,  10  9  0,   9 10  0,   9 11  0,
                9 12  0,   6 21  0,   7 21  0,   6 22  0,   7 22  0,   6 23  0,
                   (  501 More Missing: see the .ckf listing file)
```

Alert level C

```
PLAT042_ALERT_1_C Calc. and Reported MoietyFormula Strings  Differ     Please Check
              Calc: Ge I4, 2(C7 H9 Br N)
              Rep.: Ge I3, I, 2(C7 H9 Br N)
PLAT242_ALERT_2_C Low    'MainMol' Ueq as Compared to Neighbors of       C00E Check
PLAT242_ALERT_2_C Low    'MainMol' Ueq as Compared to Neighbors of       C00D Check
PLAT242_ALERT_2_C Low    'MainMol' Ueq as Compared to Neighbors of       C00I Check
PLAT906_ALERT_3_C Large K Value in the Analysis of Variance ......      4.434 Check
```

Alert level G

```
PLAT004_ALERT_5_G Polymeric Structure Found with Maximum Dimension          2 Info
PLAT007_ALERT_5_G Number of Unrefined Donor-H Atoms .............           4 Report
              H00A  H00B  H00I  H00K
PLAT012_ALERT_1_G No       _shelx_res_checksum Found in CIF ......     Please Check
PLAT093_ALERT_1_G No s.u.'s on H-positions, Refinement Reported as      mixed Check
PLAT720_ALERT_4_G Number of Unusual/Non-Standard Labels ..........         39 Note
              I001    I002    I003    I004    Br06    N008    H00A    H00B
              N009    H00I    H00K    C00A    H00C    C00C    H00D    C00B
              C00G    H00G    C00J    H00J    C00E    C00D    C00K    H00L
              C00M    H00M    C00I    C00N    H00N    C00L    H00O    C00F
              H00P    H00Q    H00R    C00H    H00E    H00F    H00H
PLAT909_ALERT_3_G Percentage of I>2sig(I) Data at Theta(Max) Still        65% Note
PLAT910_ALERT_3_G Missing FCF Reflection(s) Below Theta(Min)[Deg]=       2.40 Note
                0  2  0,
PLAT961_ALERT_5_G Dataset Contains no Negative Intensities .......     Please Check
PLAT967_ALERT_5_G Note: Two-Theta Cutoff Value in Embedded .res ..       50.0 Degree
PLAT969_ALERT_5_G The 'Henn et al.' R-Factor-gap value ..........      1.787 Note
              Predicted wR2: Based on SigI**2  4.54 or SHELX Weight  7.81
PLAT978_ALERT_2_G Number C-C Bonds with Positive Residual Density.          0 Info
PLAT992_ALERT_5_G Repd & Actual _reflns_number_gt Values Differ by          2 Check
```

1 **ALERT level A** = Most likely a serious problem - resolve or explain

```
 3 ALERT level B = A potentially serious problem, consider carefully
 5 ALERT level C = Check. Ensure it is not caused by an omission or oversight
12 ALERT level G = General information/check it is not something unexpected

 3 ALERT type 1 CIF construction/syntax error, inconsistent or missing data
 6 ALERT type 2 Indicator that the structure model may be wrong or deficient
 5 ALERT type 3 Indicator that the structure quality may be low
 1 ALERT type 4 Improvement, methodology, query or suggestion
 6 ALERT type 5 Informative message, check
```

It is advisable to attempt to resolve as many as possible of the alerts in all categories. Often the minor alerts point to easily fixed oversights, errors and omissions in your CIF or refinement strategy, so attention to these fine details can be worthwhile. In order to resolve some of the more serious problems it may be necessary to carry out additional measurements or structure refinements. However, the purpose of your study may justify the reported deviations and the more serious of these should normally be commented upon in the discussion or experimental section of a paper or in the "special_details" fields of the CIF. checkCIF was carefully designed to identify outliers and unusual parameters, but every test has its limitations and alerts that are not important in a particular case may appear. Conversely, the absence of alerts does not guarantee there are no aspects of the results needing attention. It is up to the individual to critically assess their own results and, if necessary, seek expert advice.

Publication of your CIF in IUCr journals

A basic structural check has been run on your CIF. These basic checks will be run on all CIFs submitted for publication in IUCr journals (*Acta Crystallographica*, *Journal of Applied Crystallography*, *Journal of Synchrotron Radiation*); however, if you intend to submit to *Acta Crystallographica Section C* or *E* or *IUCrData*, you should make sure that full publication checks are run on the final version of your CIF prior to submission.

Publication of your CIF in other journals

Please refer to the *Notes for Authors* of the relevant journal for any special instructions relating to CIF submission.

PLATON version of 04/06/2025; check.def file version of 30/05/2025

Datablock Nm4BrAn2GeI4 - ellipsoid plot

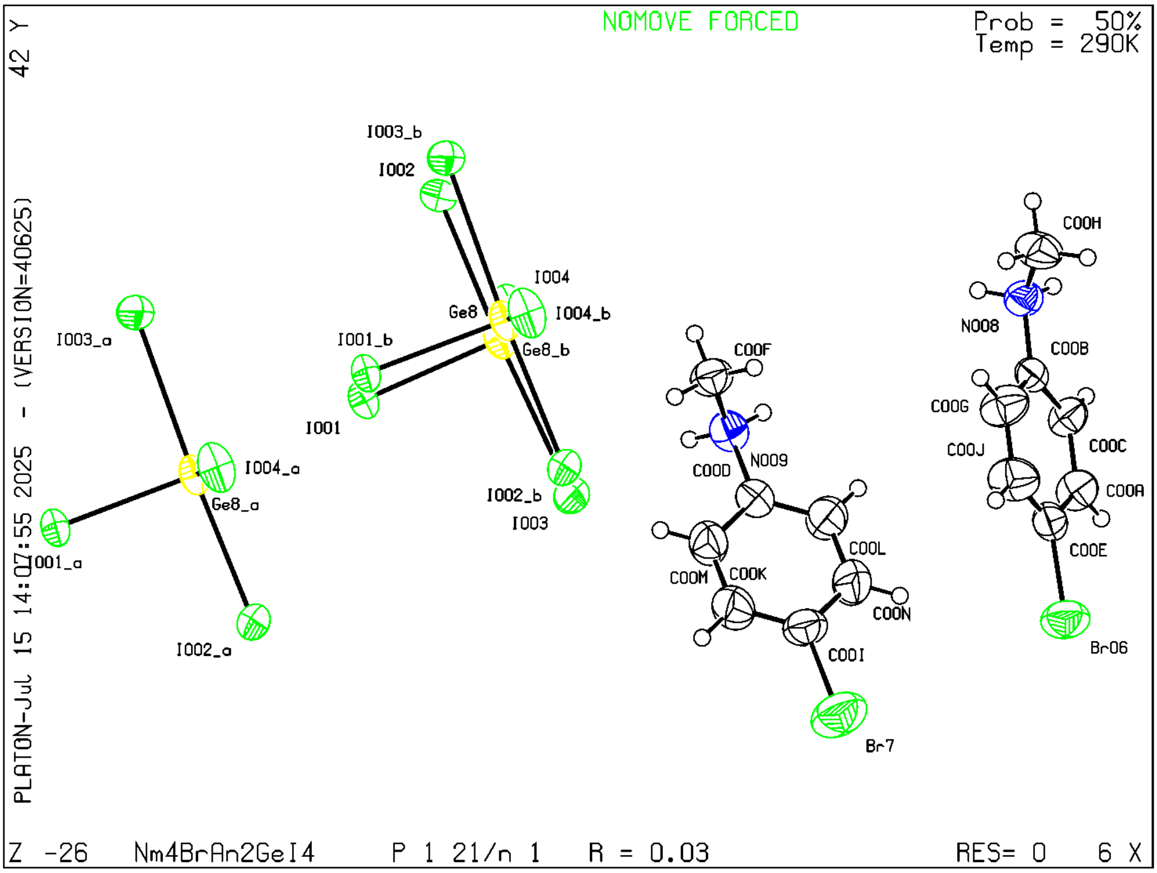

Supplement: Supplementary file 2 [file sciadv.aee5631_data_s1.zip › Ge-Nm4BrAn-checkcif.pdf]
