# Supplementary material for: Unlock lasing in two-dimensional metal halide perovskites by tuning ns2 lone pairs
Source: Sci Adv. 2026 Aug 5;12(32):eaee5631. doi: 10.1126/sciadv.aee5631 (PMC13440396; doi:10.1126/sciadv.aee5631)

checkCIF/PLATON report

Structure factors have been supplied for datablock(s) exp_13592

THIS REPORT IS FOR GUIDANCE ONLY. IF USED AS PART OF A REVIEW PROCEDURE FOR PUBLICATION, IT SHOULD NOT REPLACE THE EXPERTISE OF AN EXPERIENCED CRYSTALLOGRAPHIC REFEREE.

No syntax errors found. CIF dictionary Interpreting this report

Datablock: exp_13592

Bond precision:	C-C = 0.0053 A	Wavelength=0.71073	
Cell:	a=8.6674(3)	b=31.5650(13)	c=8.7338(3)
	alpha=90	beta=89.997(3)	gamma=90
Temperature:	290 K		

	Calculated	Reported
Volume	2389.45(15)	2389.45(15)
Space group	P 21/n	P 1 21/n 1
Hall group	-P 2yn	-P 2yn
Moiety formula	2(C7 H9 Cl N), Ge I4	Ge I3, I, 2(C7 H9 Cl N)
Sum formula	C14 H18 Cl2 Ge I4 N2	C14 H18 Cl2 Ge I4 N2
Mr	865.41	865.39
Dx,g cm-3	2.406	2.406
Z	4	4
Mu (mm-1)	6.674	6.674
F000	1576.0	1576.0
F000'	1570.73	
h,k,lmax	10,37,10	10,37,10
Nref	4216	4144
Tmin,Tmax	0.358,0.852	0.711,1.000
Tmin'	0.331	

Correction method= # Reported T Limits: Tmin=0.711 Tmax=1.000
AbsCorr = GAUSSIAN

Data completeness= 0.983 Theta(max)= 25.000

R(reflections)= 0.0287(3502) wR2(reflections)= 0.0620(4144)

S = 1.053 Npar= 186

The following ALERTS were generated. Each ALERT has the format
test-name_ALERT_alert-type_alert-level.
Click on the hyperlinks for more details of the test.

Alert level C

```
PLAT042_ALERT_1_C Calc. and Reported MoietyFormula Strings  Differ     Please Check
            Calc: 2(C7 H9 Cl N), Ge I4
            Rep.: Ge I3, I, 2(C7 H9 Cl N)
PLAT157_ALERT_4_C Non-standard Monoclinic Beta Angle less 90 Deg        90.00 Degree
PLAT241_ALERT_2_C High   'MainMol' Ueq as Compared to Neighbors of         C2 Check
PLAT242_ALERT_2_C Low    'MainMol' Ueq as Compared to Neighbors of       C00E Check
PLAT242_ALERT_2_C Low    'MainMol' Ueq as Compared to Neighbors of       C00B Check
PLAT242_ALERT_2_C Low    'MainMol' Ueq as Compared to Neighbors of       C00K Check
PLAT906_ALERT_3_C Large K Value in the Analysis of Variance ......      3.289 Check
PLAT911_ALERT_3_C Missing FCF Refl Between Thmin & STh/L=    0.595         69 Report
              3  0  1,   8 10  2,   8 11  2,   9 13  2,   9 14  2,  -2 30  2,
             -2 31  2,   9  0  3,   9  1  3,   9  2  3,   9  3  3,   5 25  5,
              5 26  5,   4 27  5,   5 27  5,   4 28  5,   4 29  5,  -4  0  6,
              0  0  6,  -5  1  6,  -4  1  6,  -5  2  6,   4 22  6,   4 23  6,
              4 24  6,   4 25  6,   4 26  6,  -5  0  7,   3 17  7,   3 18  7,
              3 19  7,   4 19  7,   3 20  7,   4 20  7,   5 20  7,   3 21  7,
              4 21  7,   3 22  7,   4 22  7,   3 23  7,   4 23  7,   3 24  7,
              3 25  7,   4 10  8,   5 10  8,   4 11  8,   5 11  8,   4 12  8,
              5 12  8,   4 13  8,   5 13  8,   4 14  8,   5 14  8,   4 15  8,
              5 15  8,   4 16  8,   3 17  8,   4 17  8,   3 18  8,   4 18  8,
              3 19  8,   3 20  8,   3 21  8,   5  0  9,   5  1  9,   4  8  9,
              4  9  9,   4 10  9,   4 11  9,
```

Alert level G

```
PLAT002_ALERT_2_G Number of Distance or Angle Restraints on AtSite          2 Note
PLAT007_ALERT_5_G Number of Unrefined Donor-H Atoms .............          4 Report
            H00A  H00B  H00I  H00J
PLAT012_ALERT_1_G No        _shelx_res_checksum Found in CIF ......     Please Check
PLAT093_ALERT_1_G No s.u.'s on H-positions, Refinement Reported as      mixed Check
PLAT172_ALERT_4_G The CIF-Embedded .res File Contains DFIX Records          1 Report
PLAT432_ALERT_2_G Short Inter X...Y Contact  I001      ..Ge05     .       3.41 Ang.
                                          1/2+x,1/2-y,1/2+z  =      4_666 Check
PLAT432_ALERT_2_G Short Inter X...Y Contact  I004      ..Ge05     .       3.48 Ang.
                                         -1/2+x,1/2-y,1/2+z  =      4_566 Check
PLAT720_ALERT_4_G Number of Unusual/Non-Standard Labels ..........         37 Note
            I001    I002    I003    I004    Ge05    Cl06    Cl07    N008
            H00A    H00B    N009    H00I    H00J    C00A    C00C    H00C
            C00D    H00D    C00E    C00L    H00L    C00H    H00H    C00B
            C00K    C00J    H00K    C00I    H00M    C00F    H00N    H00O
            H00P    C00G    H00E    H00F    H00G
PLAT860_ALERT_3_G Number of Least-Squares Restraints ............          1 Note
PLAT909_ALERT_3_G Percentage of I>2sig(I) Data at Theta(Max) Still        72% Note
PLAT910_ALERT_3_G Missing # of FCF Reflection(s) Below Theta(Min).          1 Note
              0  2  0,
PLAT933_ALERT_2_G Number of HKL-OMIT Records in Embedded .res File          1 Note
              0  0  6,
PLAT961_ALERT_5_G Dataset Contains no Negative Intensities .......     Please Check
PLAT967_ALERT_5_G Note: Two-Theta Cutoff Value in Embedded .res ..       50.0 Degree
PLAT978_ALERT_2_G Number C-C Bonds with Positive Residual Density.          0 Info
```

```
 0 ALERT level A = Most likely a serious problem - resolve or explain
 0 ALERT level B = A potentially serious problem, consider carefully
 8 ALERT level C = Check. Ensure it is not caused by an omission or oversight
15 ALERT level G = General information/check it is not something unexpected

 3 ALERT type 1 CIF construction/syntax error, inconsistent or missing data
 9 ALERT type 2 Indicator that the structure model may be wrong or deficient
 5 ALERT type 3 Indicator that the structure quality may be low
 3 ALERT type 4 Improvement, methodology, query or suggestion
 3 ALERT type 5 Informative message, check
```

It is advisable to attempt to resolve as many as possible of the alerts in all categories. Often the minor alerts point to easily fixed oversights, errors and omissions in your CIF or refinement strategy, so attention to these fine details can be worthwhile. In order to resolve some of the more serious problems it may be necessary to carry out additional measurements or structure refinements. However, the purpose of your study may justify the reported deviations and the more serious of these should normally be commented upon in the discussion or experimental section of a paper or in the "special_details" fields of the CIF. checkCIF was carefully designed to identify outliers and unusual parameters, but every test has its limitations and alerts that are not important in a particular case may appear. Conversely, the absence of alerts does not guarantee there are no aspects of the results needing attention. It is up to the individual to critically assess their own results and, if necessary, seek expert advice.

Publication of your CIF in IUCr journals

A basic structural check has been run on your CIF. These basic checks will be run on all CIFs submitted for publication in IUCr journals (*Acta Crystallographica*, *Journal of Applied Crystallography*, *Journal of Synchrotron Radiation*); however, if you intend to submit to *Acta Crystallographica Section C* or *E* or *IUCrData*, you should make sure that full publication checks are run on the final version of your CIF prior to submission.

Publication of your CIF in other journals

Please refer to the *Notes for Authors* of the relevant journal for any special instructions relating to CIF submission.

PLATON version of 29/11/2023; check.def file version of 14/09/2023

Datablock exp_13592 - ellipsoid plot

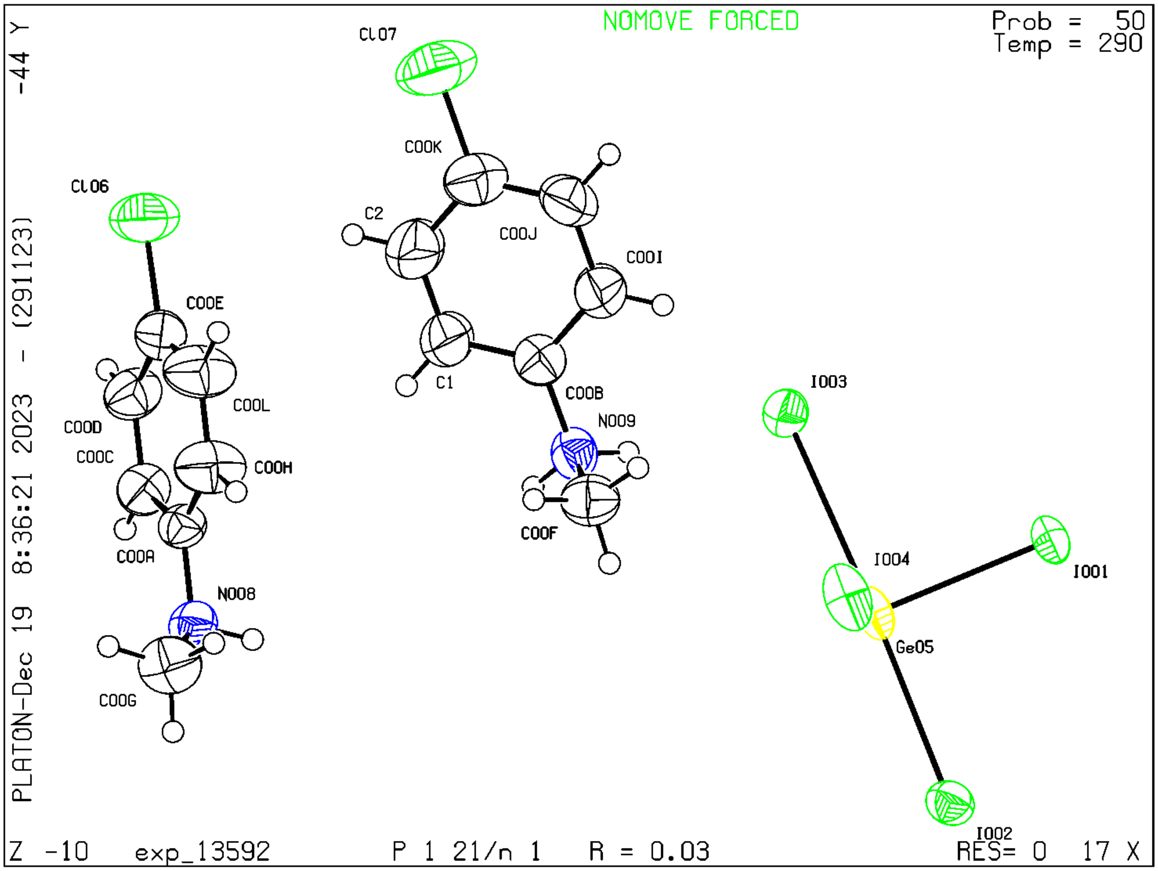

Supplement: Supplementary file 2 [file sciadv.aee5631_data_s1.zip › Ge-Nm4ClAn-checkcif.pdf]
