# Supplementary material for: Unlock lasing in two-dimensional metal halide perovskites by tuning ns2 lone pairs
Source: Sci Adv. 2026 Aug 5;12(32):eaee5631. doi: 10.1126/sciadv.aee5631 (PMC13440396; doi:10.1126/sciadv.aee5631)

checkCIF/PLATON report

Structure factors have been supplied for datablock(s) NNDmDPAGeI4

THIS REPORT IS FOR GUIDANCE ONLY. IF USED AS PART OF A REVIEW PROCEDURE FOR PUBLICATION, IT SHOULD NOT REPLACE THE EXPERTISE OF AN EXPERIENCED CRYSTALLOGRAPHIC REFEREE.

No syntax errors found. CIF dictionary Interpreting this report

Datablock: NNDmDPAGeI4

Bond precision: C-C = 0.0080 A Wavelength=0.71073

Cell:	a=8.3304(2)	b=20.7900(5)	c=18.2606(5)
	alpha=90	beta=90.591(2)	gamma=90
Temperature:	290 K		

	Calculated	Reported
Volume	3162.37(14)	3162.37(14)
Space group	P 21/c	P 1 21/c 1
Hall group	-P 2ybc	-P 2ybc
Moiety formula	C5 H16 N2, Ge I3, I	2(Ge I3), 2(I), 2(C5 H16 N2)
Sum formula	C5 H16 Ge I4 N2	C10 H32 Ge2 I8 N4
Mr	684.41	1368.78
Dx,g cm-3	2.875	2.875
Z	8	4
Mu (mm-1)	9.716	9.716
F000	2432.0	2432.0
F000'	2419.10	
h,k,lmax		9,24,21
Nref		5316
Tmin,Tmax	0.340,0.665	0.800,1.000
Tmin'	0.025	

Correction method= # Reported T Limits: Tmin=0.800 Tmax=1.000
AbsCorr = GAUSSIAN

Data completeness= Theta(max)= 25.000

R(reflections)= 0.0242(4704) wR2(reflections)= 0.0619(5316)

S = 1.076 Npar= 223

The following ALERTS were generated. Each ALERT has the format
test-name_ALERT_alert-type_alert-level.
Click on the hyperlinks for more details of the test.

Alert level B

```
PLAT029_ALERT_3_B _diffrn_measured_fraction_theta_full value Low .      0.955 Why?
```

Alert level C

```
PLAT042_ALERT_1_C Calc. and Reported MoietyFormula Strings  Differ     Please Check
              Calc: C5 H16 N2, Ge I3, I
              Rep.: 2(Ge I3), 2(I), 2(C5 H16 N2)
PLAT242_ALERT_2_C Low    'MainMol' Ueq as Compared to Neighbors of       N00B Check
PLAT242_ALERT_2_C Low    'MainMol' Ueq as Compared to Neighbors of       N00D Check
PLAT360_ALERT_2_C Short  C(sp3)-C(sp3) Bond  C00M     - C00N     .       1.43 Ang.
PLAT420_ALERT_2_C D-H Bond Without Acceptor  N00C     --H00R     .     Please Check
PLAT420_ALERT_2_C D-H Bond Without Acceptor  N00E     --H00A     .     Please Check
PLAT911_ALERT_3_C Missing FCF Refl Between Thmin & STh/L=    0.595        245 Report
               -1  0  2,  -1  2  2,  -1  3  2,  -1  7  2,  -1  1  3,  -1  2  3,
               -1  3  3,  -1  4  3,  -9  9  3,  -9  0  4,  -1  0  4,  -9  1  4,
               -1  1  4,  -9  5  4,  -9  1  5,  -1  1  5,  -2  4  5,  -2  5  5,
               -2  6  5,  -2 10  5,  -2 11  5,  -2 16  5,  -2 17  5,  -2  0  6,
                5  0  6,  -2  1  6,   5  1  6,  -2  2  6,   5  2  6,  -2  3  6,
               -2  4  6,  -2  5  6,  -2  6  6,  -2  9  6,  -2 10  6,  -2 11  6,
               -2 12  6,  -3 15  6,  -3 16  6,  -3 17  6,  -3 18  6,  -3 19  6,
               -3 20  6,  -2  1  7,   6  1  7,  -2  2  7,   6  2  7,  -2  3  7,
               -3 10  7,  -3 11  7,  -3 12  7,  -3 13  7,  -3 14  7,  -3 15  7,
               -3 16  7,  -3 17  7,  -3 18  7,  -3 19  7,  -3 20  7,  -3 21  7,
               -2  0  8,   6  0  8,   7  0  8,  -2  1  8,   6  1  8,  -2  2  8,
                6  2  8,   6  6  8,  -3  7  8,   6  7  8,  -3  8  8,  -3  9  8,
               -3 10  8,  -3 11  8,  -3 12  8,  -3 13  8,  -3 14  8,  -3 15  8,
               -3 16  8,  -3 17  8,  -3 18  8,   7  1  9,   7  2  9,  -3  3  9,
                7  3  9,  -3  4  9,  -3  5  9,   7  6  9,   6  7  9,  -3  8  9,
                6  8  9,  -3  9  9,   6  9  9,  -3 10  9,   6 10  9,  -3 11  9,
PLAT977_ALERT_2_C Check Negative Difference Density on H00Z      .      -0.37 eA-3
```

Alert level G

```
PLAT007_ALERT_5_G Number of Unrefined Donor-H Atoms .............           8 Report
              H00B  H00A  H00C  H00D  H00Q  H00R  H00S  H00T
PLAT012_ALERT_1_G No       _shelx_res_checksum Found in CIF ......     Please Check
PLAT045_ALERT_1_G Calculated and Reported Z Differ by a Factor ...          2 Check
PLAT093_ALERT_1_G No s.u.'s on H-positions, Refinement Reported as      mixed Check
PLAT432_ALERT_2_G Short Inter X...Y Contact  I001      ..Ge0A     .       3.44 Ang.
                                                 x,y,z  =      1_555 Check
PLAT432_ALERT_2_G Short Inter X...Y Contact  I005      ..Ge09     .       3.52 Ang.
                                    -1+x,1/2-y,-1/2+z  =      4_465 Check
PLAT432_ALERT_2_G Short Inter X...Y Contact  I006      ..Ge0A     .       3.52 Ang.
                                               1+x,y,z  =      1_655 Check
PLAT432_ALERT_2_G Short Inter X...Y Contact  I007      ..Ge0A     .       3.34 Ang.
                                                 x,y,z  =      1_555 Check
PLAT432_ALERT_2_G Short Inter X...Y Contact  I008      ..Ge09     .       3.32 Ang.
                                                 x,y,z  =      1_555 Check
PLAT720_ALERT_4_G Number of Unusual/Non-Standard Labels .........          51 Note
```

```
              I001    I002    I003    I004    I005    I006    I007    I008
              Ge09    Ge0A    N00B    H00B    N00C    H00Q    H00R    H00S
              N00D    H00T    N00E    H00A    H00C    H00D    C00F    H00U
              H00V    C00G    H00E    H00F    C00H    H00W    H00X    C00I
              H00G    H00H    H00I    C00J    H00Y    Ha      C00K    H00J
              H00K    H00L    C00L    H00Z    Hb      C00M    H00M    H00N
              C00N    H00O    H00P
PLAT790_ALERT_4_G Centre of Gravity not Within Unit Cell: Resd.  #          2 Note
              C5 H16 N2
PLAT909_ALERT_3_G Percentage of I>2sig(I) Data at Theta(Max) Still        78% Note
PLAT910_ALERT_3_G Missing # of FCF Reflection(s) Below Theta(Min).          4 Note
                0  2  0,   0  1  1,   0  2  1,   0  0  2,
PLAT933_ALERT_2_G Number of HKL-OMIT Records in Embedded .res File          2 Note
               -1  7  2,   0  2  1,
PLAT961_ALERT_5_G Dataset Contains no Negative Intensities .......     Please Check
PLAT967_ALERT_5_G Note: Two-Theta Cutoff Value in Embedded .res ..       50.0 Degree
PLAT978_ALERT_2_G Number C-C Bonds with Positive Residual Density.          0 Info
```

---

  0 **ALERT level A** = Most likely a serious problem - resolve or explain
  1 **ALERT level B** = A potentially serious problem, consider carefully
  8 **ALERT level C** = Check. Ensure it is not caused by an omission or oversight
 17 **ALERT level G** = General information/check it is not something unexpected

  4 ALERT type 1 CIF construction/syntax error, inconsistent or missing data
 13 ALERT type 2 Indicator that the structure model may be wrong or deficient
  4 ALERT type 3 Indicator that the structure quality may be low
  2 ALERT type 4 Improvement, methodology, query or suggestion
  3 ALERT type 5 Informative message, check

It is advisable to attempt to resolve as many as possible of the alerts in all categories. Often the minor alerts point to easily fixed oversights, errors and omissions in your CIF or refinement strategy, so attention to these fine details can be worthwhile. In order to resolve some of the more serious problems it may be necessary to carry out additional measurements or structure refinements. However, the purpose of your study may justify the reported deviations and the more serious of these should normally be commented upon in the discussion or experimental section of a paper or in the "special_details" fields of the CIF. checkCIF was carefully designed to identify outliers and unusual parameters, but every test has its limitations and alerts that are not important in a particular case may appear. Conversely, the absence of alerts does not guarantee there are no aspects of the results needing attention. It is up to the individual to critically assess their own results and, if necessary, seek expert advice.

Publication of your CIF in IUCr journals

A basic structural check has been run on your CIF. These basic checks will be run on all CIFs submitted for publication in IUCr journals (*Acta Crystallographica*, *Journal of Applied Crystallography*, *Journal of Synchrotron Radiation*); however, if you intend to submit to *Acta Crystallographica Section C* or *E* or *IUCrData*, you should make sure that full publication checks are run on the final version of your CIF prior to submission.

Publication of your CIF in other journals

Please refer to the *Notes for Authors* of the relevant journal for any special instructions relating to CIF submission.

PLATON version of 14/11/2023; check.def file version of 14/09/2023

Datablock NNDmDPAGeI4 - ellipsoid plot

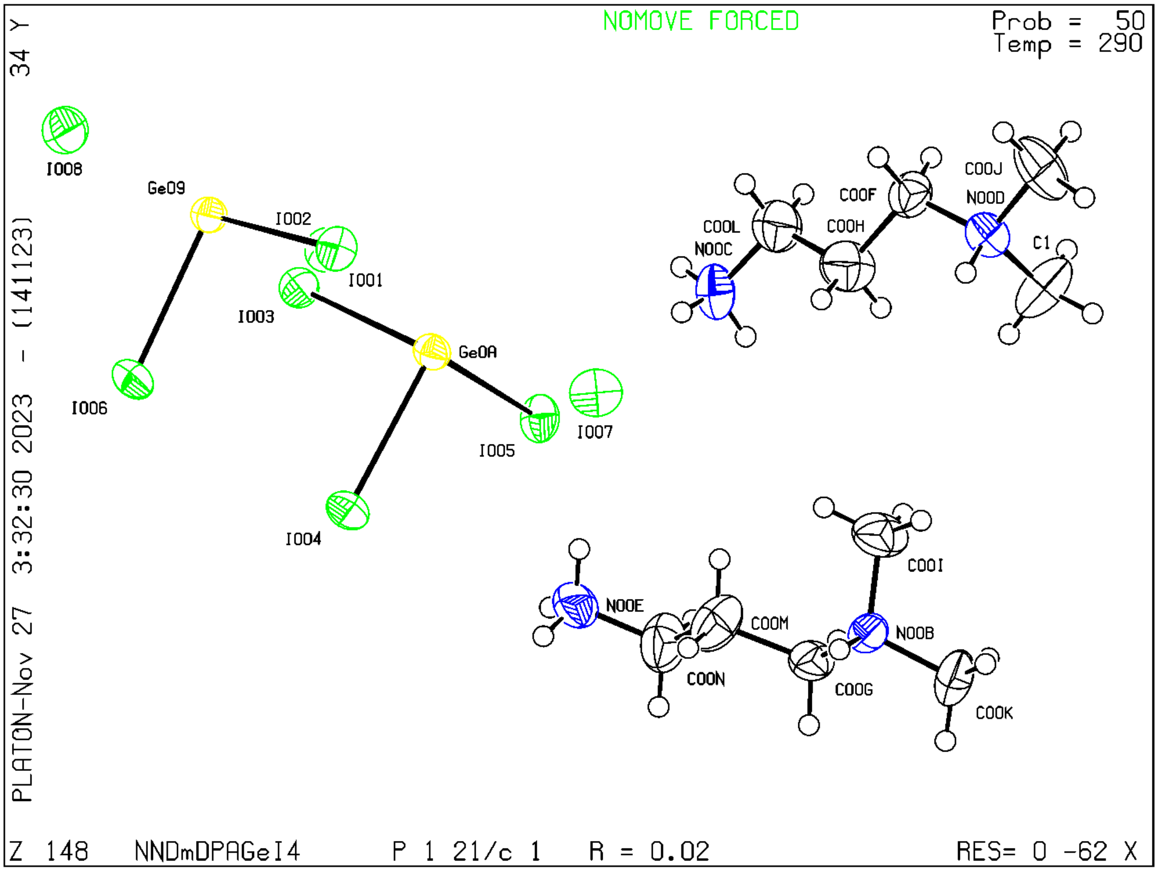

Supplement: Supplementary file 2 [file sciadv.aee5631_data_s1.zip › Ge-NNDmDPA-checkcif.pdf]
