# Supplementary material for: Unlock lasing in two-dimensional metal halide perovskites by tuning ns2 lone pairs
Source: Sci Adv. 2026 Aug 5;12(32):eaee5631. doi: 10.1126/sciadv.aee5631 (PMC13440396; doi:10.1126/sciadv.aee5631)

checkCIF/PLATON report

Structure factors have been supplied for datablock(s) exp_12325

THIS REPORT IS FOR GUIDANCE ONLY. IF USED AS PART OF A REVIEW PROCEDURE FOR PUBLICATION, IT SHOULD NOT REPLACE THE EXPERTISE OF AN EXPERIENCED CRYSTALLOGRAPHIC REFEREE.

No syntax errors found. CIF dictionary Interpreting this report

Datablock: exp_12325

Bond precision:	C-C = 0.0145 A		Wavelength=0.71073
Cell:	a=9.1941(3)	b=25.5568(9)	c=33.6148(12)
	alpha=90	beta=90	gamma=90
Temperature:	290 K		

	Calculated	Reported
Volume	7898.5(5)	7898.5(5)
Space group	P 21 21 21	P 21 21 21
Hall group	P 2ac 2ab	P 2ac 2ab
Moiety formula	6(C8 H11 Br N), 2(Ge I4), Ge I3, I	3(I), 3(Ge I3), 6(C8 H11 Br N)
Sum formula	C48 H66 Br6 Ge3 I12 N6	C48 H66 Br6 Ge3 I12 N6
Mr	2947.10	2947.09
Dx,g cm-3	2.478	2.478
Z	4	4
Mu (mm-1)	8.893	8.893
F000	5352.0	5352.0
F000'	5326.86	
h,k,lmax	13,36,47	13,35,46
Nref	23852[13018]	19936
Tmin,Tmax		0.346,1.000
Tmin'		

Correction method= # Reported T Limits: Tmin=0.346 Tmax=1.000
AbsCorr = MULTI-SCAN

Data completeness= 1.53/0.84 Theta(max)= 30.396

R(reflections)= 0.0385(16079) wR2(reflections)= 0.0988(19936)

S = 1.039 Npar= 692

The following ALERTS were generated. Each ALERT has the format
test-name_ALERT_alert-type_alert-level.
Click on the hyperlinks for more details of the test.

Alert level B

PLAT035_ALERT_1_B _chemical_absolute_configuration Info Not Given Please Do !
PLAT196_ALERT_1_B No TEMP record and _measurement_temperature .NE. 293 Degree
PLAT242_ALERT_2_B Low 'MainMol' Ueq as Compared to Neighbors of C77 Check
PLAT919_ALERT_3_B Reflection # Likely Affected by the Beamstop ... 2 Check
PLAT934_ALERT_3_B Number of (Iobs-Icalc)/Sigma(W) > 10 Outliers .. 3 Check
PLAT939_ALERT_3_B Large Value of Not (SHELXL) Weight Optimized S . 111.21 Check

Alert level C

PLAT042_ALERT_1_C Calc. and Reported MoietyFormula Strings Differ Please Check
PLAT053_ALERT_1_C Minimum Crystal Dimension Missing (or Error) ... Please Check
PLAT054_ALERT_1_C Medium Crystal Dimension Missing (or Error) ... Please Check
PLAT055_ALERT_1_C Maximum Crystal Dimension Missing (or Error) ... Please Check
PLAT213_ALERT_2_C Atom N65 has ADP max/min Ratio ..... 3.2 prolat
PLAT213_ALERT_2_C Atom C74 has ADP max/min Ratio ..... 3.2 prolat
PLAT220_ALERT_2_C NonSolvent Resd 5 C Ueq(max)/Ueq(min) Range 4.0 Ratio
PLAT222_ALERT_3_C NonSolvent Resd 5 H Uiso(max)/Uiso(min) Range 4.5 Ratio
PLAT234_ALERT_4_C Large Hirshfeld Difference N54 --C53 . 0.19 Ang.
PLAT234_ALERT_4_C Large Hirshfeld Difference N65 --C77 . 0.17 Ang.
PLAT242_ALERT_2_C Low 'MainMol' Ueq as Compared to Neighbors of C29 Check
PLAT242_ALERT_2_C Low 'MainMol' Ueq as Compared to Neighbors of C48 Check
PLAT242_ALERT_2_C Low 'MainMol' Ueq as Compared to Neighbors of C53 Check
PLAT242_ALERT_2_C Low 'MainMol' Ueq as Compared to Neighbors of C56 Check
PLAT242_ALERT_2_C Low 'MainMol' Ueq as Compared to Neighbors of C66 Check
PLAT334_ALERT_2_C Small <C-C> Benzene Dist. C56 -C61 . 1.37 Ang.
PLAT342_ALERT_3_C Low Bond Precision on C-C Bonds .... 0.01452 Ang.
PLAT420_ALERT_2_C D-H Bond Without Acceptor N24 --H24B . Please Check
PLAT420_ALERT_2_C D-H Bond Without Acceptor N34 --H34A . Please Check
PLAT420_ALERT_2_C D-H Bond Without Acceptor N34 --H34B . Please Check
PLAT420_ALERT_2_C D-H Bond Without Acceptor N34 --H34C . Please Check
PLAT420_ALERT_2_C D-H Bond Without Acceptor N54 --H54B . Please Check
PLAT420_ALERT_2_C D-H Bond Without Acceptor N66 --H66B . Please Check
PLAT767_ALERT_4_C INS Embedded LIST 6 Instruction Should be LIST 4 Please Check
PLAT910_ALERT_3_C Missing # of FCF Reflection(s) Below Theta(Min). 7 Note
PLAT911_ALERT_3_C Missing FCF Refl Between Thmin & STh/L= 0.600 2 Report
PLAT915_ALERT_3_C No Flack x Check Done: Low Friedel Pair Coverage 78 %
PLAT918_ALERT_3_C Reflection(s) with I(obs) much Smaller I(calc) . 1 Check
PLAT971_ALERT_2_C Check Calcd Resid. Dens. 0.82Ang From I15 1.71 eA-3
PLAT971_ALERT_2_C Check Calcd Resid. Dens. 0.80Ang From I15 1.61 eA-3
PLAT971_ALERT_2_C Check Calcd Resid. Dens. 0.90Ang From I7 1.55 eA-3

Alert level G

PLAT007_ALERT_5_G Number of Unrefined Donor-H Atoms .... 18 Report
PLAT083_ALERT_2_G SHELXL Second Parameter in WGHT Unusually Large 9.52 Why ?
PLAT115_ALERT_5_G ADDSYM Detects Noncrystallographic Inversion ... 82% Check
PLAT164_ALERT_4_G Nr. of Refined C-H H-Atoms in Heavy-Atom Struct. 1 Note
PLAT343_ALERT_2_G Unusual sp? Angle Range in Main Residue for C76 Check
PLAT432_ALERT_2_G Short Inter X...Y Contact I2 ..Ge73 . 3.35 Ang.

```
                                                 x,y,z  =      1_555 Check
PLAT432_ALERT_2_G Short Inter X...Y Contact  I9       ..Ge74     .       3.57 Ang.
                                      -1/2+x,1/2-y,1-z  =      4_456 Check
PLAT432_ALERT_2_G Short Inter X...Y Contact  I10      ..Ge73     .       3.50 Ang.
                                                 x,y,z  =      1_555 Check
PLAT790_ALERT_4_G Centre of Gravity not Within Unit Cell: Resd.  #          5 Note
              C8 H11 Br N
PLAT790_ALERT_4_G Centre of Gravity not Within Unit Cell: Resd.  #          7 Note
              Ge I4
PLAT883_ALERT_1_G No Info/Value for _atom_sites_solution_primary .     Please Do !
PLAT912_ALERT_4_G Missing # of FCF Reflections Above STh/L=  0.600       1526 Note
PLAT978_ALERT_2_G Number C-C Bonds with Positive Residual Density.          0 Info
PLAT992_ALERT_5_G Repd & Actual _reflns_number_gt Values Differ by          2 Check
```

---

   0 **ALERT level A** = Most likely a serious problem - resolve or explain
   6 **ALERT level B** = A potentially serious problem, consider carefully
  31 **ALERT level C** = Check. Ensure it is not caused by an omission or oversight
  14 **ALERT level G** = General information/check it is not something unexpected

   7 ALERT type 1 CIF construction/syntax error, inconsistent or missing data
  25 ALERT type 2 Indicator that the structure model may be wrong or deficient
   9 ALERT type 3 Indicator that the structure quality may be low
   7 ALERT type 4 Improvement, methodology, query or suggestion
   3 ALERT type 5 Informative message, check

It is advisable to attempt to resolve as many as possible of the alerts in all categories. Often the minor alerts point to easily fixed oversights, errors and omissions in your CIF or refinement strategy, so attention to these fine details can be worthwhile. In order to resolve some of the more serious problems it may be necessary to carry out additional measurements or structure refinements. However, the purpose of your study may justify the reported deviations and the more serious of these should normally be commented upon in the discussion or experimental section of a paper or in the "special_details" fields of the CIF. checkCIF was carefully designed to identify outliers and unusual parameters, but every test has its limitations and alerts that are not important in a particular case may appear. Conversely, the absence of alerts does not guarantee there are no aspects of the results needing attention. It is up to the individual to critically assess their own results and, if necessary, seek expert advice.

Publication of your CIF in IUCr journals

A basic structural check has been run on your CIF. These basic checks will be run on all CIFs submitted for publication in IUCr journals (*Acta Crystallographica*, *Journal of Applied Crystallography*, *Journal of Synchrotron Radiation*); however, if you intend to submit to *Acta Crystallographica Section C* or *E* or *IUCrData*, you should make sure that full publication checks are run on the final version of your CIF prior to submission.

Publication of your CIF in other journals

Please refer to the *Notes for Authors* of the relevant journal for any special instructions relating to CIF submission.

PLATON version of 10/05/2023; check.def file version of 10/05/2023

Datablock exp_12325 - ellipsoid plot

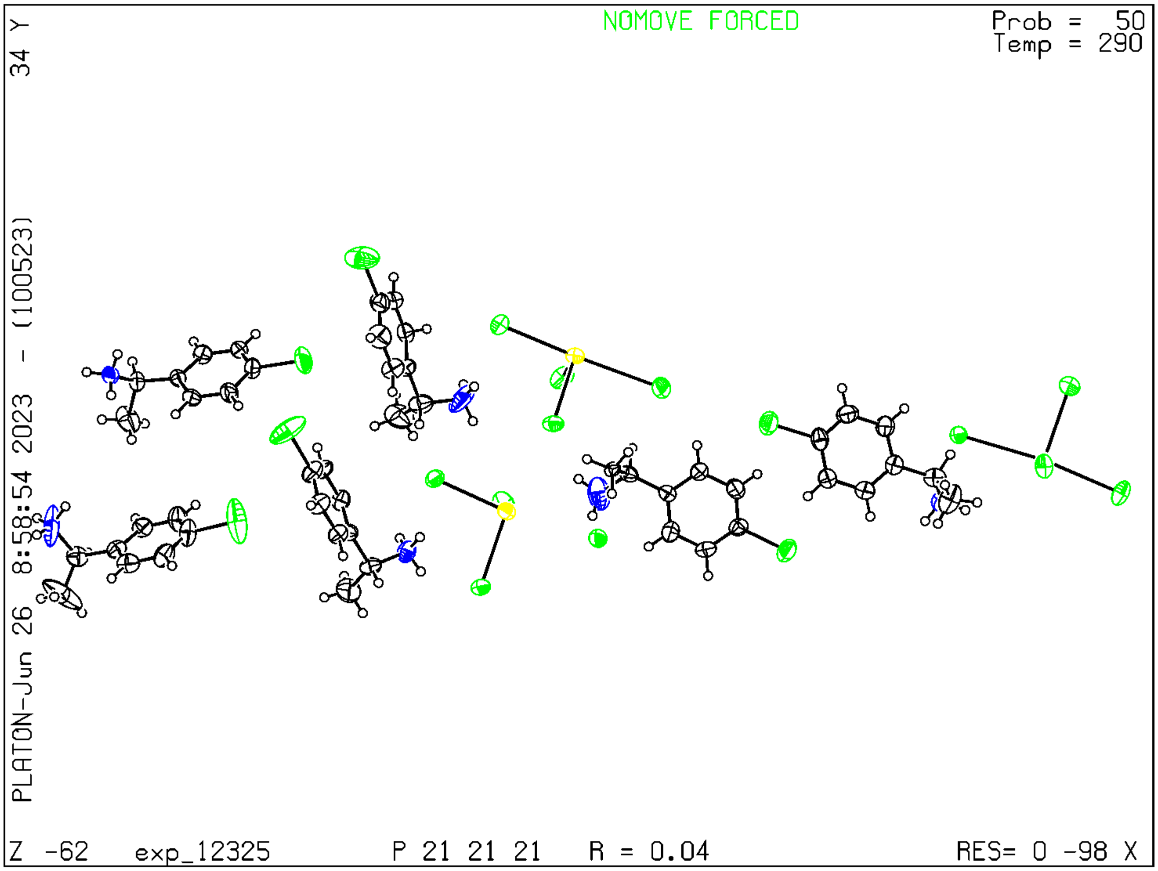

Supplement: Supplementary file 2 [file sciadv.aee5631_data_s1.zip › Ge-RBrMBA_cifreport.pdf]
