# Supplementary material for: Unlock lasing in two-dimensional metal halide perovskites by tuning ns2 lone pairs
Source: Sci Adv. 2026 Aug 5;12(32):eaee5631. doi: 10.1126/sciadv.aee5631 (PMC13440396; doi:10.1126/sciadv.aee5631)

checkCIF/PLATON report

Structure factors have been supplied for datablock(s) 2FBMZ2PbI4

THIS REPORT IS FOR GUIDANCE ONLY. IF USED AS PART OF A REVIEW PROCEDURE FOR PUBLICATION, IT SHOULD NOT REPLACE THE EXPERTISE OF AN EXPERIENCED CRYSTALLOGRAPHIC REFEREE.

No syntax errors found.   CIF dictionary   Interpreting this report

Datablock: 2FBMZ2PbI4

Bond precision: C-C = 0.0085 A   Wavelength=0.71073

Cell:	a=12.5966(3)	b=6.2867(2)	c=30.0956(9)
	alpha=90	beta=94.558(3)	gamma=90
Temperature:	290 K		

	Calculated	Reported
Volume	2375.76(12)	2375.76(12)
Space group	I 2/a	I 1 2/a 1
Hall group	-I 2ya	-I 2ya
Moiety formula	I4 Pb, 2(C7 H5 F2 N2)	I2 Pb0.5, C7 H5 F2 N2
Sum formula	C14 H10 F4 I4 N4 Pb	C7 H5 F2 I2 N2 Pb0.50
Mr	1025.06	512.52
Dx,g cm-3	2.866	2.866
Z	4	8
Mu (mm-1)	12.337	12.337
F000	1808.0	1808.0
F000'	1788.41	
h,k,lmax	17,8,41	17,8,39
Nref	3320	2952
Tmin,Tmax	0.150,0.682	0.524,1.000
Tmin'	0.049	

Correction method= # Reported T Limits: Tmin=0.524 Tmax=1.000
AbsCorr = GAUSSIAN

Data completeness= 0.889   Theta(max)= 29.521

R(reflections)= 0.0271(2543)   wR2(reflections)= 0.0740(2952)

S = 0.999   Npar= 125

The following ALERTS were generated. Each ALERT has the format
test-name_ALERT_alert-type_alert-level.
Click on the hyperlinks for more details of the test.

Alert level C

```
PLAT041_ALERT_1_C Calc. and Reported SumFormula    Strings  Differ     Please Check
              Calc: C14 H10 F4 I4 N4 Pb
              Rep.: C7 H5 F2 I2 N2 Pb0.50
PLAT042_ALERT_1_C Calc. and Reported MoietyFormula Strings  Differ     Please Check
              Calc: I4 Pb, 2(C7 H5 F2 N2)
              Rep.: I2 Pb0.5, C7 H5 F2 N2
PLAT242_ALERT_2_C Low    'MainMol' Ueq as Compared to Neighbors of       Pb01 Check
PLAT342_ALERT_3_C Low Bond Precision on  C-C Bonds ...............     0.0085 Ang.
PLAT972_ALERT_2_C Check Calcd Resid. Dens.  1.13Ang From Pb01           -1.74 eA-3
```

Alert level G

```
PLAT004_ALERT_5_G Polymeric Structure Found with Maximum Dimension          2 Info
PLAT007_ALERT_5_G Number of Unrefined Donor-H Atoms .............           2 Report
              H005  H006
PLAT012_ALERT_1_G No       _shelx_res_checksum Found in CIF ......     Please Check
PLAT045_ALERT_1_G Calculated and Reported Z Differ by a Factor ...      0.500 Check
PLAT232_ALERT_2_G Hirshfeld Test Diff (M-X)  Pb01     --I002     .        9.7 s.u.
PLAT232_ALERT_2_G Hirshfeld Test Diff (M-X)  Pb01     --I003     .       10.0 s.u.
PLAT232_ALERT_2_G Hirshfeld Test Diff (M-X)  Pb01     --I004     .       16.5 s.u.
PLAT720_ALERT_4_G Number of Unusual/Non-Standard Labels .........          18 Note
              Pb01    I002    I003    I004    N005    H005    N006    H006
              C007    F008    C009    C00A    H00A    F00B    C00C    H00C
              C00D    H00D
PLAT794_ALERT_5_G Tentative Bond Valency for Pb01      (II)      .       2.38 Info
PLAT910_ALERT_3_G Missing FCF Reflection(s) Below Theta(Min)[Deg]=       2.72 Note
                0  0  2,
PLAT912_ALERT_4_G Missing # of FCF Reflections Above STh/L=  0.600        351 Note
PLAT933_ALERT_2_G Number of HKL-OMIT Records in Embedded .res File          1 Note
                4  0 26,
PLAT952_ALERT_5_G Calculated (ThMax) and CIF-Reported Lmax Differ.          2 Units
PLAT958_ALERT_1_G Calculated (ThMax) and Actual (FCF) Lmax Differ.          2 Units
PLAT969_ALERT_5_G The 'Henn et al.' R-Factor-gap value ..........       2.089 Note
              Predicted wR2: Based on SigI**2  3.54 or SHELX Weight  7.40
PLAT978_ALERT_2_G Number C-C Bonds with Positive Residual Density.          0 Info
```

0 **ALERT level A** = Most likely a serious problem - resolve or explain
0 **ALERT level B** = A potentially serious problem, consider carefully
5 **ALERT level C** = Check. Ensure it is not caused by an omission or oversight
16 **ALERT level G** = General information/check it is not something unexpected

5 ALERT type 1 CIF construction/syntax error, inconsistent or missing data
7 ALERT type 2 Indicator that the structure model may be wrong or deficient
2 ALERT type 3 Indicator that the structure quality may be low
2 ALERT type 4 Improvement, methodology, query or suggestion
5 ALERT type 5 Informative message, check

It is advisable to attempt to resolve as many as possible of the alerts in all categories. Often the minor alerts point to easily fixed oversights, errors and omissions in your CIF or refinement strategy, so attention to these fine details can be worthwhile. In order to resolve some of the more serious problems it may be necessary to carry out additional measurements or structure refinements. However, the purpose of your study may justify the reported deviations and the more serious of these should normally be commented upon in the discussion or experimental section of a paper or in the "special_details" fields of the CIF. checkCIF was carefully designed to identify outliers and unusual parameters, but every test has its limitations and alerts that are not important in a particular case may appear. Conversely, the absence of alerts does not guarantee there are no aspects of the results needing attention. It is up to the individual to critically assess their own results and, if necessary, seek expert advice.

Publication of your CIF in IUCr journals

A basic structural check has been run on your CIF. These basic checks will be run on all CIFs submitted for publication in IUCr journals (*Acta Crystallographica*, *Journal of Applied Crystallography*, *Journal of Synchrotron Radiation*); however, if you intend to submit to *Acta Crystallographica Section C* or *E* or *IUCrData*, you should make sure that full publication checks are run on the final version of your CIF prior to submission.

Publication of your CIF in other journals

Please refer to the *Notes for Authors* of the relevant journal for any special instructions relating to CIF submission.

PLATON version of 04/06/2025; check.def file version of 30/05/2025

Datablock 2FBMZ2PbI4 - ellipsoid plot

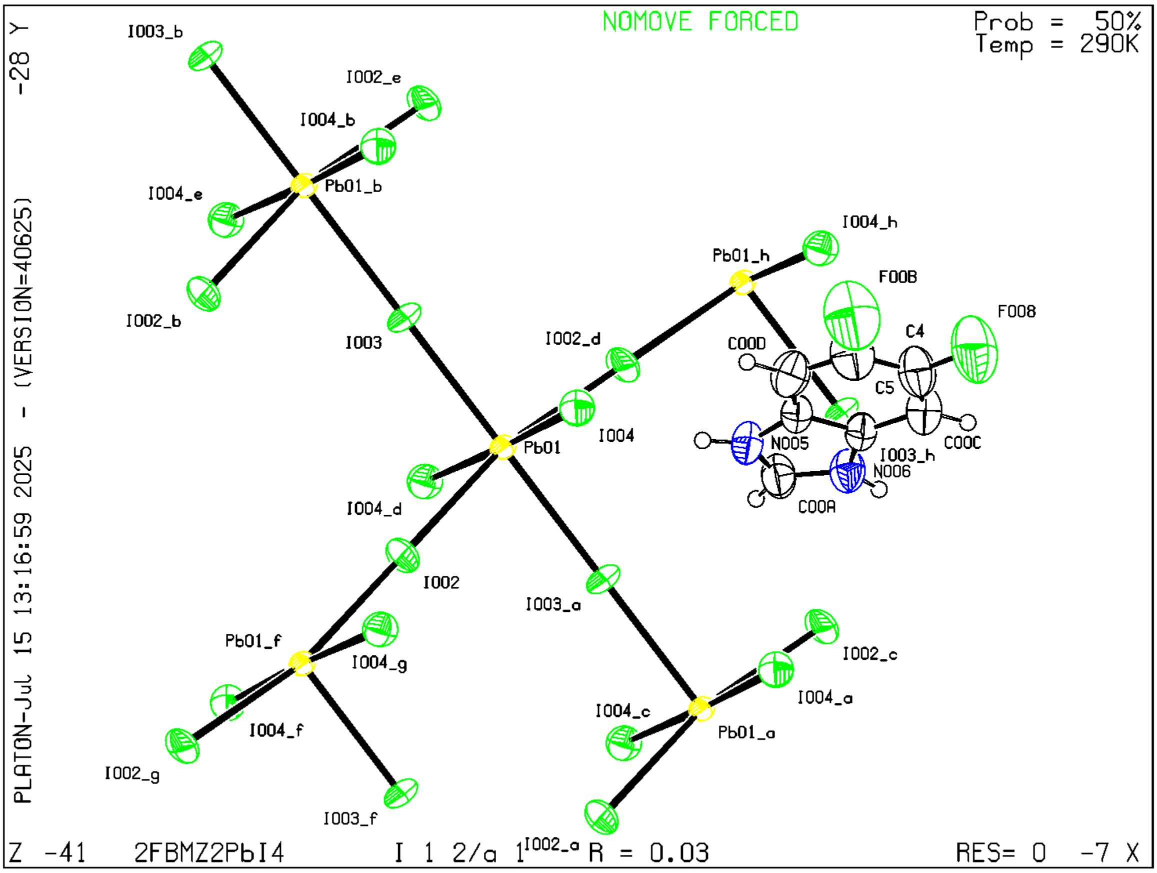

Supplement: Supplementary file 2 [file sciadv.aee5631_data_s1.zip › Pb-2FBMZ-checkcif.pdf]
