# Supplementary material for: Unlock lasing in two-dimensional metal halide perovskites by tuning ns2 lone pairs
Source: Sci Adv. 2026 Aug 5;12(32):eaee5631. doi: 10.1126/sciadv.aee5631 (PMC13440396; doi:10.1126/sciadv.aee5631)

checkCIF/PLATON report

Structure factors have been supplied for datablock(s) 4ClPMA2SnI4

THIS REPORT IS FOR GUIDANCE ONLY. IF USED AS PART OF A REVIEW PROCEDURE FOR PUBLICATION, IT SHOULD NOT REPLACE THE EXPERTISE OF AN EXPERIENCED CRYSTALLOGRAPHIC REFEREE.

No syntax errors found. CIF dictionary Interpreting this report

Datablock: 4ClPMA2SnI4

```
Bond precision:    C-C = 0.0051 A           Wavelength=0.71073

Cell:              a=16.9029(8)     b=8.7967(4)          c=8.6482(4)
                   alpha=90         beta=104.299(5)      gamma=90
Temperature:       290 K

                 Calculated                  Reported
Volume           1246.06(10)                 1246.06(10)
Space group      P 21/c                      P 1 21/c 1
Hall group       -P 2ybc                     -P 2ybc
Moiety formula   I4 Sn, 2(C7 H9 Cl N)        I4 Sn, 2(C7 H9 Cl N)
Sum formula      C14 H18 Cl2 I4 N2 Sn        C14 H18 Cl2 I4 N2 Sn
Mr               911.51                      911.49
Dx,g cm-3        2.429                       2.429
Z                2                           2
Mu (mm-1)        6.195                       6.195
F000             824.0                       824.0
F000'            819.76
h,k,lmax         20,10,10                    20,10,10
Nref             2198                        2197
Tmin,Tmax        0.487,0.928                 0.653,1.000
Tmin'            0.389

Correction method= # Reported T Limits: Tmin=0.653 Tmax=1.000
AbsCorr = GAUSSIAN

Data completeness= 1.000            Theta(max)= 25.000

                                                      wR2(reflections)=
R(reflections)= 0.0196( 1930)                         0.0453( 2197)

S = 1.052                    Npar= 95
```

The following ALERTS were generated. Each ALERT has the format
test-name_ALERT_alert-type_alert-level.
Click on the hyperlinks for more details of the test.

Alert level C

```
PLAT242_ALERT_2_C Low    'MainMol' Ueq as Compared to Neighbors of       C00B Check
PLAT242_ALERT_2_C Low    'MainMol' Ueq as Compared to Neighbors of        C10 Check
PLAT420_ALERT_2_C D-H Bond Without Acceptor  N005     --H00A     .     Please Check
```

Alert level G

```
PLAT004_ALERT_5_G Polymeric Structure Found with Maximum Dimension          2 Info
PLAT007_ALERT_5_G Number of Unrefined Donor-H Atoms .............          3 Report
              H00A  H00B  H00C
PLAT012_ALERT_1_G No       _shelx_res_checksum Found in CIF ......     Please Check
PLAT093_ALERT_1_G No s.u.'s on H-positions, Refinement Reported as      mixed Check
PLAT232_ALERT_2_G Hirshfeld Test Diff (M-X)  I002     --Sn1      .        6.5 s.u.
PLAT710_ALERT_4_G Delete 1-2-3 or 2-3-4 Linear Torsion Angle ... #          1 Do !
              I002-SN1 -I002-SN1   -73.39  0.08   3_576   1_555   1_555   2_546
PLAT720_ALERT_4_G Number of Unusual/Non-Standard Labels ..........         19 Note
              I002    I003    Cl04    N005    H00A    H00B    H00C    C009
              H009    C00C    H00D    C00B    C00A    H00E    C008    H008
              C007    H00F    H00G
PLAT764_ALERT_4_G Overcomplete CIF Bond List Detected (Rep/Expd) .       1.33 Ratio
PLAT794_ALERT_5_G Tentative Bond Valency for Sn1       (II)      .       2.36 Info
PLAT909_ALERT_3_G Percentage of I>2sig(I) Data at Theta(Max) Still        70% Note
PLAT910_ALERT_3_G Missing # of FCF Reflection(s) Below Theta(Min).          1 Note
                1  0  0,
PLAT961_ALERT_5_G Dataset Contains no Negative Intensities .......     Please Check
PLAT967_ALERT_5_G Note: Two-Theta Cutoff Value in Embedded .res ..       50.0 Degree
PLAT969_ALERT_5_G The 'Henn et al.' R-Factor-gap value ..........       1.53 Note
              Predicted wR2: Based on SigI**2  2.97 or SHELX Weight  4.41
PLAT978_ALERT_2_G Number C-C Bonds with Positive Residual Density.          1 Info
```

---

0 **ALERT level A** = Most likely a serious problem - resolve or explain
0 **ALERT level B** = A potentially serious problem, consider carefully
3 **ALERT level C** = Check. Ensure it is not caused by an omission or oversight
15 **ALERT level G** = General information/check it is not something unexpected

2 ALERT type 1 CIF construction/syntax error, inconsistent or missing data
5 ALERT type 2 Indicator that the structure model may be wrong or deficient
2 ALERT type 3 Indicator that the structure quality may be low
3 ALERT type 4 Improvement, methodology, query or suggestion
6 ALERT type 5 Informative message, check

It is advisable to attempt to resolve as many as possible of the alerts in all categories. Often the minor alerts point to easily fixed oversights, errors and omissions in your CIF or refinement strategy, so attention to these fine details can be worthwhile. In order to resolve some of the more serious problems it may be necessary to carry out additional measurements or structure refinements. However, the purpose of your study may justify the reported deviations and the more serious of these should normally be commented upon in the discussion or experimental section of a paper or in the "special_details" fields of the CIF. checkCIF was carefully designed to identify outliers and unusual parameters, but every test has its limitations and alerts that are not important in a particular case may appear. Conversely, the absence of alerts does not guarantee there are no aspects of the results needing attention. It is up to the individual to critically assess their own results and, if necessary, seek expert advice.

Publication of your CIF in IUCr journals

A basic structural check has been run on your CIF. These basic checks will be run on all CIFs submitted for publication in IUCr journals (*Acta Crystallographica*, *Journal of Applied Crystallography*, *Journal of Synchrotron Radiation*); however, if you intend to submit to *Acta Crystallographica Section C* or *E* or *IUCrData*, you should make sure that full publication checks are run on the final version of your CIF prior to submission.

Publication of your CIF in other journals

Please refer to the *Notes for Authors* of the relevant journal for any special instructions relating to CIF submission.

PLATON version of 06/01/2024; check.def file version of 05/01/2024

Datablock 4ClPMA2SnI4 - ellipsoid plot

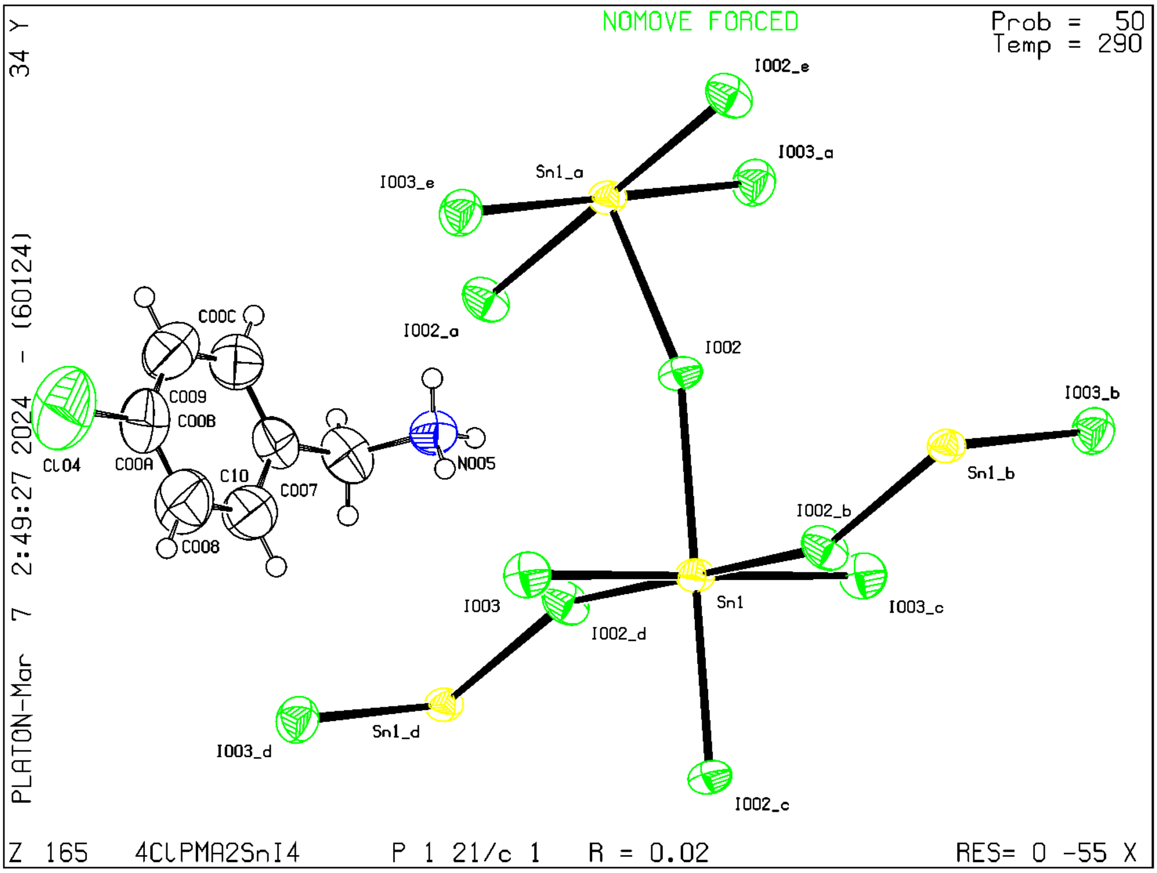

Supplement: Supplementary file 2 [file sciadv.aee5631_data_s1.zip › Sn-4ClPMA-checkcif.pdf]
