# Supplementary material for: Unlock lasing in two-dimensional metal halide perovskites by tuning ns2 lone pairs
Source: Sci Adv. 2026 Aug 5;12(32):eaee5631. doi: 10.1126/sciadv.aee5631 (PMC13440396; doi:10.1126/sciadv.aee5631)

checkCIF/PLATON report

Structure factors have been supplied for datablock(s) exp_11565

THIS REPORT IS FOR GUIDANCE ONLY. IF USED AS PART OF A REVIEW PROCEDURE FOR PUBLICATION, IT SHOULD NOT REPLACE THE EXPERTISE OF AN EXPERIENCED CRYSTALLOGRAPHIC REFEREE.

No syntax errors found. CIF dictionary Interpreting this report

Datablock: exp_11565

```
Bond precision:   C-C = 0.0055 A          Wavelength=0.71073

Cell:              a=8.7254(3)     b=9.2905(3)         c=27.8298(9)
                   alpha=90        beta=98.009(3)      gamma=90
Temperature:       290 K

                 Calculated                        Reported
Volume           2233.97(13)                       2233.97(13)
Space group      P 21/n                            P 1 21/n 1
Hall group       -P 2yn                            -P 2yn
Moiety formula   I4 Sn, 2(C7 H9 F N)               I4 Sn, 2(C7 H9 F N)
Sum formula      C14 H18 F2 I4 N2 Sn               C14 H18 F2 I4 N2 Sn
Mr               878.61                            878.59
Dx,g cm-3        2.612                             2.612
Z                4                                 4
Mu (mm-1)        6.687                             6.687
F000             1584.0                            1584.0
F000'            1574.45
h,k,lmax         12,12,38                          11,12,38
Nref             6190                              5629
Tmin,Tmax                                          0.579,1.000
Tmin'

Correction method= # Reported T Limits: Tmin=0.579 Tmax=1.000
AbsCorr = MULTI-SCAN

Data completeness= 0.909          Theta(max)= 29.434

R(reflections)= 0.0268( 4350)                      wR2(reflections)=
                                                   0.0554( 5629)
S = 1.035                    Npar= 210
```

The following ALERTS were generated. Each ALERT has the format
test-name_ALERT_alert-type_alert-level.
Click on the hyperlinks for more details of the test.

Alert level B

```
PLAT196_ALERT_1_B No TEMP record and _measurement_temperature .NE.        293 Degree
PLAT919_ALERT_3_B Reflection # Likely Affected by the Beamstop ...          1 Check
```

Alert level C

```
PLAT053_ALERT_1_C Minimum Crystal Dimension Missing (or Error) ...     Please Check
PLAT054_ALERT_1_C Medium  Crystal Dimension Missing (or Error) ...     Please Check
PLAT055_ALERT_1_C Maximum Crystal Dimension Missing (or Error) ...     Please Check
PLAT334_ALERT_2_C Small <C-C> Benzene Dist.   C6       -C11      .       1.37 Ang.
PLAT334_ALERT_2_C Small <C-C> Benzene Dist.   C15      -C20      .       1.37 Ang.
PLAT420_ALERT_2_C D-H Bond Without Acceptor  N1       --H1B      .     Please Check
PLAT420_ALERT_2_C D-H Bond Without Acceptor  N2       --H2A      .     Please Check
PLAT767_ALERT_4_C INS Embedded LIST 6 Instruction Should be LIST 4     Please Check
PLAT906_ALERT_3_C Large K Value in the Analysis of Variance ......      2.221 Check
PLAT911_ALERT_3_C Missing FCF Refl Between Thmin & STh/L=    0.600          2 Report
PLAT934_ALERT_3_C Number of (Iobs-Icalc)/Sigma(W) > 10 Outliers ..          1 Check
```

Alert level G

```
PLAT004_ALERT_5_G Polymeric Structure Found with Maximum Dimension          2 Info
PLAT007_ALERT_5_G Number of Unrefined Donor-H Atoms .............           6 Report
PLAT232_ALERT_2_G Hirshfeld Test Diff (M-X)  I1       --Sn1      .       15.0 s.u.
PLAT232_ALERT_2_G Hirshfeld Test Diff (M-X)  I3       --Sn1      .       47.5 s.u.
PLAT232_ALERT_2_G Hirshfeld Test Diff (M-X)  I3       --Sn1_c    .       58.0 s.u.
PLAT232_ALERT_2_G Hirshfeld Test Diff (M-X)  I4       --Sn1      .       32.0 s.u.
PLAT232_ALERT_2_G Hirshfeld Test Diff (M-X)  I4       --Sn1_b    .       49.5 s.u.
PLAT232_ALERT_2_G Hirshfeld Test Diff (M-X)  I5       --Sn1      .       32.1 s.u.
PLAT794_ALERT_5_G Tentative Bond Valency for Sn1       (II)      .       2.13 Info
PLAT883_ALERT_1_G No Info/Value for _atom_sites_solution_primary .     Please Do !
PLAT910_ALERT_3_G Missing # of FCF Reflection(s) Below Theta(Min).          1 Note
PLAT912_ALERT_4_G Missing # of FCF Reflections Above STh/L=  0.600        551 Note
PLAT941_ALERT_3_G Average HKL Measurement Multiplicity ..........         3.1 Low
PLAT978_ALERT_2_G Number C-C Bonds with Positive Residual Density.          2 Info
```

0 **ALERT level A** = Most likely a serious problem - resolve or explain
2 **ALERT level B** = A potentially serious problem, consider carefully
11 **ALERT level C** = Check. Ensure it is not caused by an omission or oversight
14 **ALERT level G** = General information/check it is not something unexpected

5 ALERT type 1 CIF construction/syntax error, inconsistent or missing data
11 ALERT type 2 Indicator that the structure model may be wrong or deficient
6 ALERT type 3 Indicator that the structure quality may be low
2 ALERT type 4 Improvement, methodology, query or suggestion
3 ALERT type 5 Informative message, check

It is advisable to attempt to resolve as many as possible of the alerts in all categories. Often the minor alerts point to easily fixed oversights, errors and omissions in your CIF or refinement strategy, so attention to these fine details can be worthwhile. In order to resolve some of the more serious problems it may be necessary to carry out additional measurements or structure refinements. However, the purpose of your study may justify the reported deviations and the more serious of these should normally be commented upon in the discussion or experimental section of a paper or in the "special_details" fields of the CIF. checkCIF was carefully designed to identify outliers and unusual parameters, but every test has its limitations and alerts that are not important in a particular case may appear. Conversely, the absence of alerts does not guarantee there are no aspects of the results needing attention. It is up to the individual to critically assess their own results and, if necessary, seek expert advice.

Publication of your CIF in IUCr journals

A basic structural check has been run on your CIF. These basic checks will be run on all CIFs submitted for publication in IUCr journals (*Acta Crystallographica*, *Journal of Applied Crystallography*, *Journal of Synchrotron Radiation*); however, if you intend to submit to *Acta Crystallographica Section C* or *E* or *IUCrData*, you should make sure that full publication checks are run on the final version of your CIF prior to submission.

Publication of your CIF in other journals

Please refer to the *Notes for Authors* of the relevant journal for any special instructions relating to CIF submission.

PLATON version of 28/11/2022; check.def file version of 28/11/2022

Datablock exp_11565 - ellipsoid plot

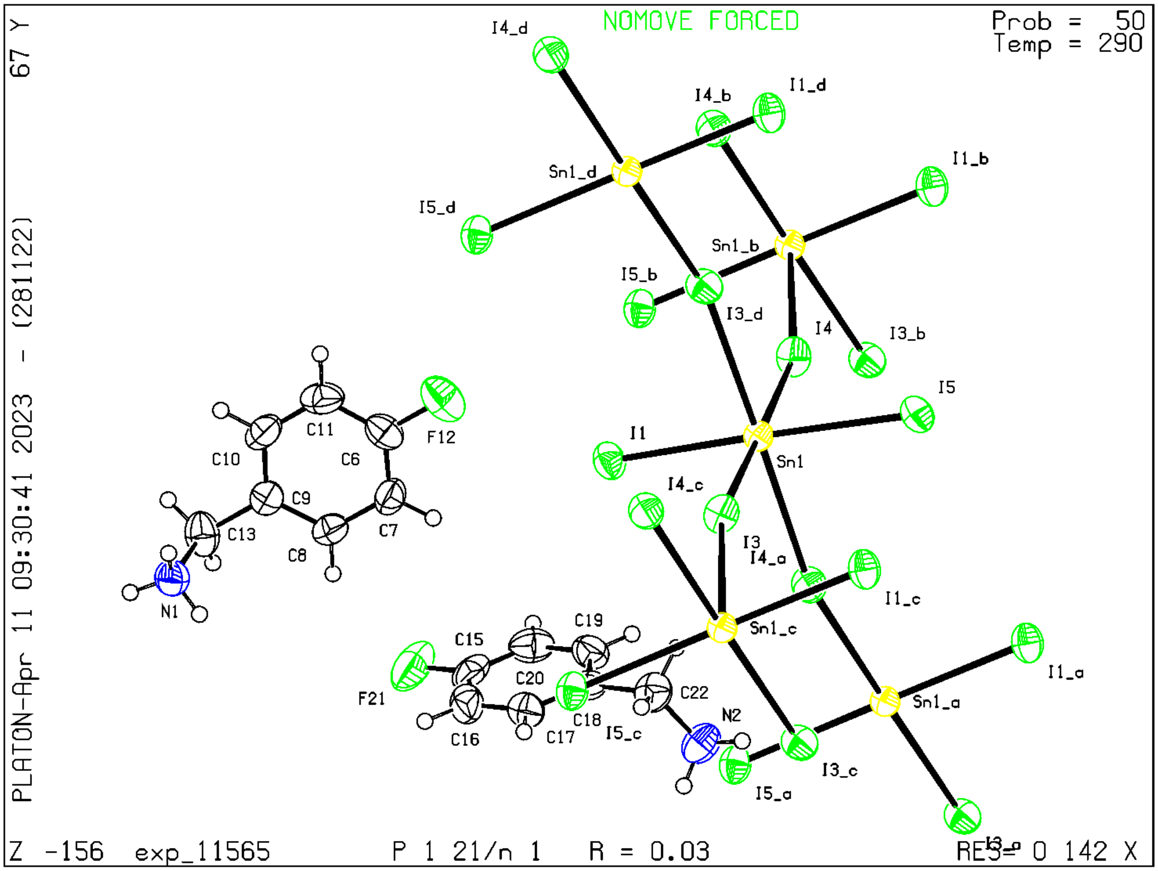

Supplement: Supplementary file 2 [file sciadv.aee5631_data_s1.zip › Sn-4FPMA-checkcif.pdf]
