# Supplementary material for: Unlock lasing in two-dimensional metal halide perovskites by tuning ns2 lone pairs
Source: Sci Adv. 2026 Aug 5;12(32):eaee5631. doi: 10.1126/sciadv.aee5631 (PMC13440396; doi:10.1126/sciadv.aee5631)

checkCIF/PLATON report

Structure factors have been supplied for datablock(s) Sn-4IAn

THIS REPORT IS FOR GUIDANCE ONLY. IF USED AS PART OF A REVIEW PROCEDURE FOR PUBLICATION, IT SHOULD NOT REPLACE THE EXPERTISE OF AN EXPERIENCED CRYSTALLOGRAPHIC REFEREE.

No syntax errors found.   CIF dictionary   Interpreting this report

Datablock: Sn-4IAn

Bond precision: C-C = 0.0052 A   Wavelength=0.71073

Cell:	a=15.7290(8)	b=8.2576(4)	c=9.1466(4)
	alpha=90	beta=100.181(4)	gamma=90
Temperature:	290 K		

	Calculated	Reported
Volume	1169.29(10)	1169.30(10)
Space group	P 21/c	P 1 21/c 1
Hall group	-P 2ybc	-P 2ybc
Moiety formula	I4 Sn, 2(C6 H7 I N)	I2 Sn0.5, C6 H7 I N
Sum formula	C12 H14 I6 N2 Sn	C6 H7 I3 N Sn0.50
Mr	1066.36	533.17
Dx,g cm-3	3.029	3.029
Z	2	4
Mu (mm-1)	9.010	9.010
F000	936.0	936.0
F000’	929.39	
h,k,lmax	20,10,11	20,10,11
Nref	2679	2618
Tmin,Tmax	0.135,0.679	0.235,1.000
Tmin’	0.103	

Correction method= # Reported T Limits: Tmin=0.235 Tmax=1.000
AbsCorr = GAUSSIAN

Data completeness= 0.977   Theta(max)= 27.489

R(reflections)= 0.0457(2193)   wR2(reflections)= 0.1118(2618)

S = 1.020   Npar= 86

The following ALERTS were generated. Each ALERT has the format
test-name_ALERT_alert-type_alert-level.
Click on the hyperlinks for more details of the test.

Alert level C

```
PLAT042_ALERT_1_C Calc. and Reported MoietyFormula Strings  Differ     Please Check
              Calc: I4 Sn, 2(C6 H7 I N)
              Rep.: I2 Sn0.5, C6 H7 I N
PLAT911_ALERT_3_C Missing FCF Refl Between Thmin & STh/L=    0.600         38 Report
               13  0  0,  13  1  0,  14  0  0,  14  1  0,  15  0  0,  15  1  0,
               16  0  0,  16  1  0,  17  0  0,  17  1  0,  18  0  0,  18  1  0,
              -18  1  1, -17  1  1, -16  1  1, -15  1  1, -14  1  1, -13  1  1,
               13  1  1,  14  1  1,  14  3  1,  15  1  1,  15  3  1,  16  1  1,
               16  3  1,  17  1  1,  18  1  1,  14  0  2,  15  0  2,  15  1  2,
               16  0  2,  16  1  2,  16  3  2,  17  0  2,   6  6  6,   4  5  7,
                5  5  7,   6  6  7,
PLAT972_ALERT_2_C Check Calcd Resid. Dens.  0.70Ang From I4             -1.59 eA-3
PLAT972_ALERT_2_C Check Calcd Resid. Dens.  0.93Ang From I4             -1.59 eA-3
PLAT972_ALERT_2_C Check Calcd Resid. Dens.  0.75Ang From I1             -1.54 eA-3
```

Alert level G

```
PLAT004_ALERT_5_G Polymeric Structure Found with Maximum Dimension          2 Info
PLAT007_ALERT_5_G Number of Unrefined Donor-H Atoms .............           3 Report
              H3A   H3B   H3C
PLAT012_ALERT_1_G No       _shelx_res_checksum Found in CIF ......     Please Check
PLAT045_ALERT_1_G Calculated and Reported Z Differ by a Factor ...      0.500 Check
PLAT764_ALERT_4_G Overcomplete CIF Bond List Detected (Rep/Expd) .       1.27 Ratio
PLAT794_ALERT_5_G Tentative Bond Valency for Sn1       (II)      .       2.25 Info
PLAT910_ALERT_3_G Missing # of FCF Reflection(s) Below Theta(Min).          1 Note
                1  0  0,
PLAT912_ALERT_4_G Missing # of FCF Reflections Above STh/L=  0.600         22 Note
PLAT969_ALERT_5_G The 'Henn et al.' R-Factor-gap value ..........        1.56 Note
              Predicted wR2: Based on SigI**2  7.18 or SHELX Weight 11.14
PLAT978_ALERT_2_G Number C-C Bonds with Positive Residual Density.          0 Info
```

```
   0 ALERT level A = Most likely a serious problem - resolve or explain
   0 ALERT level B = A potentially serious problem, consider carefully
   5 ALERT level C = Check. Ensure it is not caused by an omission or oversight
  10 ALERT level G = General information/check it is not something unexpected

   3 ALERT type 1 CIF construction/syntax error, inconsistent or missing data
   4 ALERT type 2 Indicator that the structure model may be wrong or deficient
   2 ALERT type 3 Indicator that the structure quality may be low
   2 ALERT type 4 Improvement, methodology, query or suggestion
   4 ALERT type 5 Informative message, check
```

It is advisable to attempt to resolve as many as possible of the alerts in all categories. Often the minor alerts point to easily fixed oversights, errors and omissions in your CIF or refinement strategy, so attention to these fine details can be worthwhile. In order to resolve some of the more serious problems it may be necessary to carry out additional measurements or structure refinements. However, the purpose of your study may justify the reported deviations and the more serious of these should normally be commented upon in the discussion or experimental section of a paper or in the "special_details" fields of the CIF. checkCIF was carefully designed to identify outliers and unusual parameters, but every test has its limitations and alerts that are not important in a particular case may appear. Conversely, the absence of alerts does not guarantee there are no aspects of the results needing attention. It is up to the individual to critically assess their own results and, if necessary, seek expert advice.

Publication of your CIF in IUCr journals

A basic structural check has been run on your CIF. These basic checks will be run on all CIFs submitted for publication in IUCr journals (*Acta Crystallographica*, *Journal of Applied Crystallography*, *Journal of Synchrotron Radiation*); however, if you intend to submit to *Acta Crystallographica Section C* or *E* or *IUCrData*, you should make sure that full publication checks are run on the final version of your CIF prior to submission.

Publication of your CIF in other journals

Please refer to the *Notes for Authors* of the relevant journal for any special instructions relating to CIF submission.

PLATON version of 06/01/2024; check.def file version of 05/01/2024

Datablock Sn-4IAn - ellipsoid plot

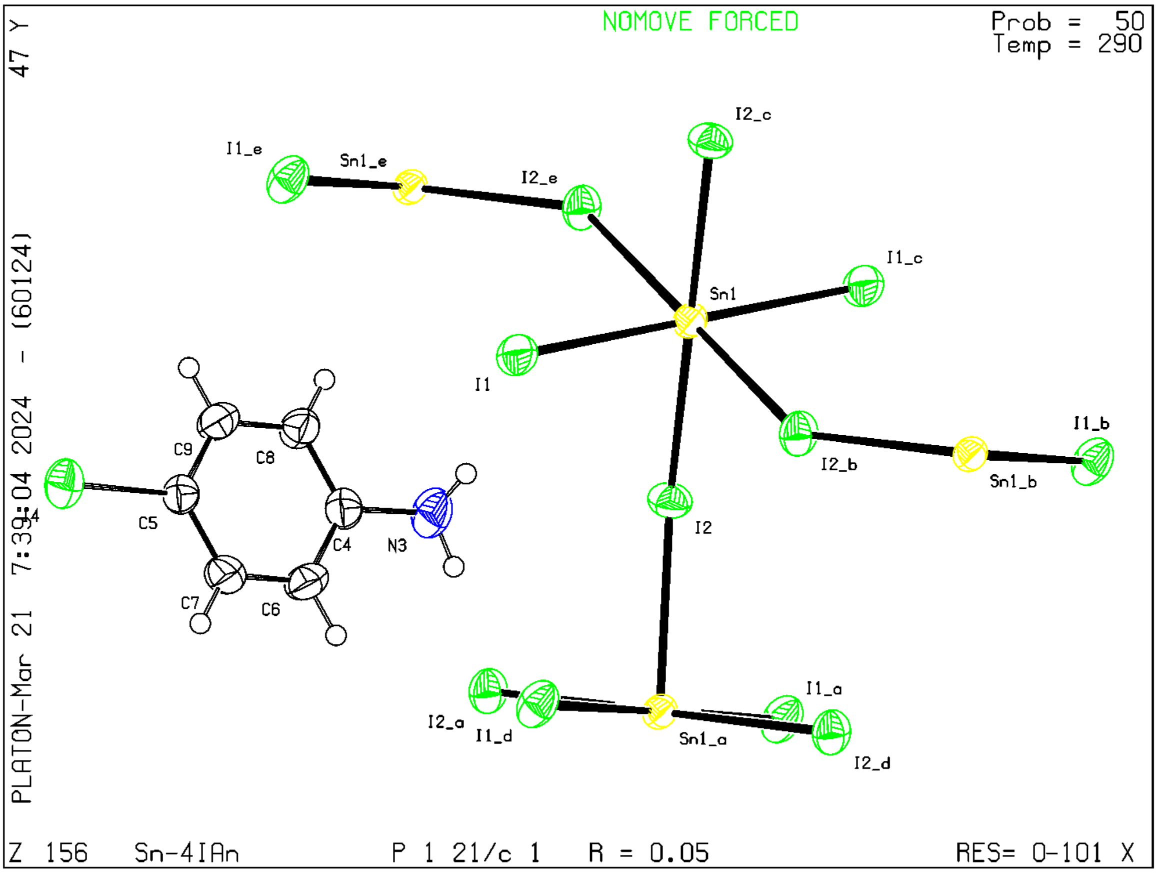

Supplement: Supplementary file 2 [file sciadv.aee5631_data_s1.zip › Sn-4IAn-checkcif.pdf]
