# Supplementary material for: Unlock lasing in two-dimensional metal halide perovskites by tuning ns2 lone pairs
Source: Sci Adv. 2026 Aug 5;12(32):eaee5631. doi: 10.1126/sciadv.aee5631 (PMC13440396; doi:10.1126/sciadv.aee5631)

checkCIF/PLATON report

Structure factors have been supplied for datablock(s) exp_11556

THIS REPORT IS FOR GUIDANCE ONLY. IF USED AS PART OF A REVIEW PROCEDURE FOR PUBLICATION, IT SHOULD NOT REPLACE THE EXPERTISE OF AN EXPERIENCED CRYSTALLOGRAPHIC REFEREE.

No syntax errors found. CIF dictionary Interpreting this report

Datablock: exp_11556

Bond precision: C-C = 0.0288 A Wavelength=0.71073

Cell:	a=9.0022(2)	b=9.0022(2)	c=65.4006(18)
	alpha=90	beta=90	gamma=90
Temperature:	290 K		

	Calculated	Reported
Volume	5300.0(3)	5300.0(3)
Space group	P 41 21 2	P 41 21 2
Hall group	P 4abw 2nw	P 4abw 2nw
Moiety formula	I4 Sn, 2(C8 H11 Br N)	I3 Sn, I, 2(C8 H11 Br N)
Sum formula	C16 H22 Br2 I4 N2 Sn	C16 H22 Br2 I4 N2 Sn
Mr	1028.47	1028.46
Dx,g cm-3	2.578	2.578
Z	8	8
Mu (mm-1)	8.644	8.644
F000	3712.0	3712.0
F000’	3688.77	
h,k,lmax	12,12,90	12,12,89
Nref	7381[4439]	6446
Tmin,Tmax		0.568,1.000
Tmin’		

Correction method= # Reported T Limits: Tmin=0.568 Tmax=1.000
AbsCorr = MULTI-SCAN

Data completeness= 1.45/0.87 Theta(max)= 29.467

R(reflections)= 0.0602(4939) wR2(reflections)= 0.1330(6446)

S = 1.134 Npar= 231

The following ALERTS were generated. Each ALERT has the format
test-name_ALERT_alert-type_alert-level.
Click on the hyperlinks for more details of the test.

Alert level B

```
PLAT035_ALERT_1_B _chemical_absolute_configuration Info  Not Given     Please Do !
PLAT196_ALERT_1_B No TEMP record and _measurement_temperature .NE.        293 Degree
PLAT234_ALERT_4_B Large Hirshfeld Difference C18      --C23      .       0.26 Ang.
PLAT342_ALERT_3_B Low Bond Precision on  C-C Bonds ..............    0.02875 Ang.
PLAT919_ALERT_3_B Reflection # Likely Affected by the Beamstop ...          1 Check
PLAT934_ALERT_3_B Number of (Iobs-Icalc)/Sigma(W) > 10 Outliers ..          3 Check
PLAT987_ALERT_1_B The Flack x is >> 0 -  Do a BASF/TWIN Refinement     Please Check
```

Alert level C

```
PLAT042_ALERT_1_C Calc. and Reported MoietyFormula Strings  Differ     Please Check
PLAT053_ALERT_1_C Minimum Crystal Dimension Missing (or Error) ...     Please Check
PLAT054_ALERT_1_C Medium  Crystal Dimension Missing (or Error) ...     Please Check
PLAT055_ALERT_1_C Maximum Crystal Dimension Missing (or Error) ...     Please Check
PLAT213_ALERT_2_C Atom N23             has ADP max/min Ratio .....        3.3 oblate
PLAT213_ALERT_2_C Atom C25             has ADP max/min Ratio .....        3.5 oblate
PLAT234_ALERT_4_C Large Hirshfeld Difference C24      --C25      .       0.19 Ang.
PLAT241_ALERT_2_C High   'MainMol' Ueq as Compared to Neighbors of         C9 Check
PLAT241_ALERT_2_C High   'MainMol' Ueq as Compared to Neighbors of        C23 Check
PLAT242_ALERT_2_C Low    'MainMol' Ueq as Compared to Neighbors of        C14 Check
PLAT242_ALERT_2_C Low    'MainMol' Ueq as Compared to Neighbors of        C18 Check
PLAT242_ALERT_2_C Low    'MainMol' Ueq as Compared to Neighbors of        C21 Check
PLAT250_ALERT_2_C Large U3/U1 Ratio for Average U(i,j) Tensor ....        2.8 Note
PLAT334_ALERT_2_C Small <C-C> Benzene Dist.   C18       -C23      .       1.37 Ang.
PLAT414_ALERT_2_C Short Intra D-H..H-X       H23B      ..H25A     .       1.95 Ang.
                                                       x,y,z  =      1_555 Check
PLAT420_ALERT_2_C D-H Bond Without Acceptor  N24      --H24B     .     Please Check
PLAT767_ALERT_4_C INS Embedded LIST 6 Instruction Should be LIST 4     Please Check
PLAT906_ALERT_3_C Large K Value in the Analysis of Variance ......      4.867 Check
PLAT911_ALERT_3_C Missing FCF Refl Between Thmin & STh/L=    0.600          5 Report
PLAT972_ALERT_2_C Check Calcd Resid. Dens.  2.20Ang From I6             -1.99 eA-3
PLAT972_ALERT_2_C Check Calcd Resid. Dens.  2.50Ang From I3             -1.71 eA-3
PLAT977_ALERT_2_C Check Negative Difference Density on H12       .      -0.35 eA-3
PLAT977_ALERT_2_C Check Negative Difference Density on H14       .      -0.40 eA-3
PLAT977_ALERT_2_C Check Negative Difference Density on H24       .      -0.41 eA-3
PLAT977_ALERT_2_C Check Negative Difference Density on H24C      .      -0.37 eA-3
```

Alert level G

```
PLAT003_ALERT_2_G Number of Uiso or Uij Restrained non-H Atoms ...          1 Report
PLAT004_ALERT_5_G Polymeric Structure Found with Maximum Dimension          2 Info
PLAT007_ALERT_5_G Number of Unrefined Donor-H Atoms ..............          6 Report
PLAT033_ALERT_4_G Flack x Value Deviates > 3.0 * sigma from Zero .      0.048 Note
PLAT083_ALERT_2_G SHELXL Second Parameter in WGHT  Unusually Large      61.53 Why ?
PLAT186_ALERT_4_G The CIF-Embedded .res File Contains ISOR Records          1 Report
PLAT232_ALERT_2_G Hirshfeld Test Diff (M-X)  I1       --Sn1      .        5.5 s.u.
PLAT232_ALERT_2_G Hirshfeld Test Diff (M-X)  I6       --Sn1      .       20.7 s.u.
PLAT794_ALERT_5_G Tentative Bond Valency for Sn1       (II)      .       2.14 Info
PLAT860_ALERT_3_G Number of Least-Squares Restraints ............           6 Note
```

```
PLAT883_ALERT_1_G No Info/Value for _atom_sites_solution_primary .     Please Do !
PLAT910_ALERT_3_G Missing # of FCF Reflection(s) Below Theta(Min).          1 Note
PLAT912_ALERT_4_G Missing # of FCF Reflections Above STh/L=  0.600        349 Note
PLAT941_ALERT_3_G Average HKL Measurement Multiplicity ..........         4.4 Low
PLAT978_ALERT_2_G Number C-C Bonds with Positive Residual Density.          0 Info
```

```
   0 ALERT level A = Most likely a serious problem - resolve or explain
   7 ALERT level B = A potentially serious problem, consider carefully
  25 ALERT level C = Check. Ensure it is not caused by an omission or oversight
  15 ALERT level G = General information/check it is not something unexpected

   8 ALERT type 1 CIF construction/syntax error, inconsistent or missing data
  22 ALERT type 2 Indicator that the structure model may be wrong or deficient
   8 ALERT type 3 Indicator that the structure quality may be low
   6 ALERT type 4 Improvement, methodology, query or suggestion
   3 ALERT type 5 Informative message, check
```

It is advisable to attempt to resolve as many as possible of the alerts in all categories. Often the minor alerts point to easily fixed oversights, errors and omissions in your CIF or refinement strategy, so attention to these fine details can be worthwhile. In order to resolve some of the more serious problems it may be necessary to carry out additional measurements or structure refinements. However, the purpose of your study may justify the reported deviations and the more serious of these should normally be commented upon in the discussion or experimental section of a paper or in the "special_details" fields of the CIF. checkCIF was carefully designed to identify outliers and unusual parameters, but every test has its limitations and alerts that are not important in a particular case may appear. Conversely, the absence of alerts does not guarantee there are no aspects of the results needing attention. It is up to the individual to critically assess their own results and, if necessary, seek expert advice.

Publication of your CIF in IUCr journals

A basic structural check has been run on your CIF. These basic checks will be run on all CIFs submitted for publication in IUCr journals (*Acta Crystallographica*, *Journal of Applied Crystallography*, *Journal of Synchrotron Radiation*); however, if you intend to submit to *Acta Crystallographica Section C* or *E* or *IUCrData*, you should make sure that full publication checks are run on the final version of your CIF prior to submission.

Publication of your CIF in other journals

Please refer to the *Notes for Authors* of the relevant journal for any special instructions relating to CIF submission.

PLATON version of 28/11/2022; check.def file version of 28/11/2022

Datablock exp_11556 - ellipsoid plot

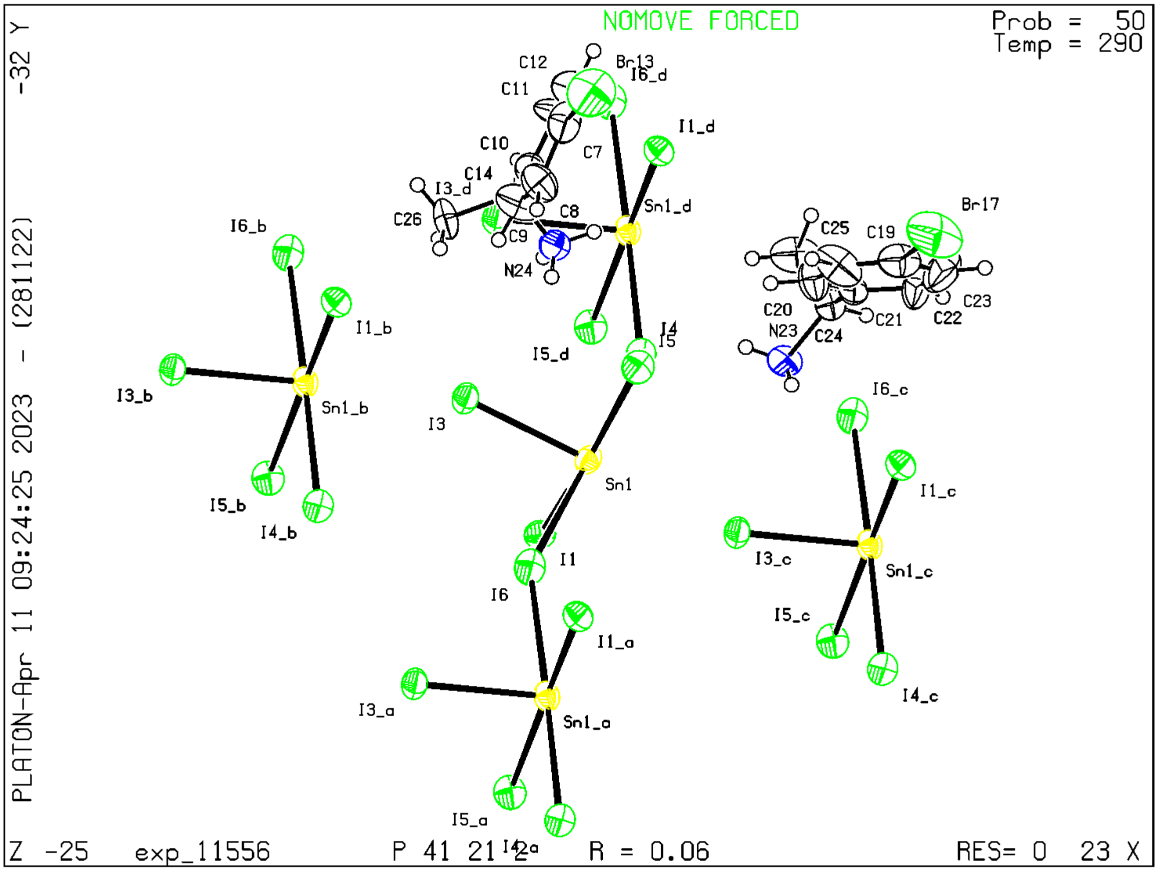

Supplement: Supplementary file 2 [file sciadv.aee5631_data_s1.zip › Sn-BrMBA-checkcif.pdf]
