# Supplementary material for: Unlock lasing in two-dimensional metal halide perovskites by tuning ns2 lone pairs
Source: Sci Adv. 2026 Aug 5;12(32):eaee5631. doi: 10.1126/sciadv.aee5631 (PMC13440396; doi:10.1126/sciadv.aee5631)

checkCIF/PLATON report

Structure factors have been supplied for datablock(s) 2FPEA2GeI4

THIS REPORT IS FOR GUIDANCE ONLY. IF USED AS PART OF A REVIEW PROCEDURE FOR PUBLICATION, IT SHOULD NOT REPLACE THE EXPERTISE OF AN EXPERIENCED CRYSTALLOGRAPHIC REFEREE.

No syntax errors found. CIF dictionary Interpreting this report

Datablock: 2FPEA2GeI4

```
Bond precision:   C-C = 0.0144 A          Wavelength=0.71073

Cell:           a=12.0191(7)        b=12.0967(6)      c=17.6365(11)
                alpha=107.357(5)    beta=99.670(5)    gamma=90.056(4)
Temperature:    290 K
```

	Calculated	Reported
Volume	2409.1(3)	2409.1(2)
Space group	P -1	P -1
Hall group	-P 1	-P 1
Moiety formula	C16 H21 F2 Ge2 I6 N2, 2(C8 H10.50 F N), 2(I)	3.72(I), 2(Ge I2.142), 2(C8 H10.5 F N), C8 H11 F N, C8 H10 F N
Sum formula	C32 H42 F4 Ge2 I8 N4	C32 H42 F4 Ge2 I8 N4
Mr	1719.12	1719.08
Dx,g cm-3	2.370	2.370
Z	2	2
Mu (mm-1)	6.416	6.416
F000	1572.0	1572.0
F000’	1565.67	
h,k,lmax		14,14,20
Nref		8456
Tmin,Tmax	0.417,0.863	0.711,1.000
Tmin’	0.408	

```
Correction method= # Reported T Limits: Tmin=0.711 Tmax=1.000
AbsCorr = GAUSSIAN

Data completeness=                 Theta(max)= 25.000
```

R(reflections)= 0.0499(6126)   wR2(reflections)= 0.1116(8456)

S = 1.049   Npar= 465

The following ALERTS were generated. Each ALERT has the format
test-name_ALERT_alert-type_alert-level.
Click on the hyperlinks for more details of the test.

Alert level B

```
PLAT230_ALERT_2_B Hirshfeld Test Diff for    I002     --Ge0A     .       25.3 s.u.
PLAT230_ALERT_2_B Hirshfeld Test Diff for    I004     --Ge09     .       26.1 s.u.
PLAT234_ALERT_4_B Large Hirshfeld Difference C00Y     --C00Z     .       0.28 Ang.
PLAT780_ALERT_1_B Coordinates do not Form a Properly Connected Set     Please Do !
```

Alert level C

```
PLAT042_ALERT_1_C Calc. and Reported MoietyFormula Strings  Differ     Please Check
              Calc: C16 H21 F2 Ge2 I6 N2, 2(C8 H10.50 F N), 2(I)
              Rep.: 3.72(I), 2(Ge I2.142), 2(C8 H10.5 F N), C8 H11 F N, C8 H10 F
PLAT112_ALERT_2_C ADDSYM Detects New (Pseudo) Symm. Elem       a/2         86 %Fit
PLAT220_ALERT_2_C NonSolvent   Resd 1  C   Ueq(max)/Ueq(min) Range        3.5 Ratio
PLAT230_ALERT_2_C Hirshfeld Test Diff for    C011     --C20      .        6.0 s.u.
PLAT234_ALERT_4_C Large Hirshfeld Difference F1       --C00T     .       0.19 Ang.
PLAT234_ALERT_4_C Large Hirshfeld Difference F2       --C00U     .       0.21 Ang.
PLAT234_ALERT_4_C Large Hirshfeld Difference N17      --C19      .       0.16 Ang.
PLAT234_ALERT_4_C Large Hirshfeld Difference C00J     --C00Z     .       0.24 Ang.
PLAT234_ALERT_4_C Large Hirshfeld Difference C013     --C00Y     .       0.24 Ang.
PLAT234_ALERT_4_C Large Hirshfeld Difference C00N     --C011     .       0.20 Ang.
PLAT234_ALERT_4_C Large Hirshfeld Difference C00R     --C19      .       0.21 Ang.
PLAT234_ALERT_4_C Large Hirshfeld Difference N18      --C00S     .       0.16 Ang.
PLAT241_ALERT_2_C High   'MainMol' Ueq as Compared to Neighbors of       C00Z Check
PLAT241_ALERT_2_C High   'MainMol' Ueq as Compared to Neighbors of        C19 Check
PLAT241_ALERT_2_C High   'MainMol' Ueq as Compared to Neighbors of        C20 Check
PLAT241_ALERT_2_C High   'MainMol' Ueq as Compared to Neighbors of        C26 Check
PLAT241_ALERT_2_C High   'MainMol' Ueq as Compared to Neighbors of        C24 Check
PLAT241_ALERT_2_C High   'MainMol' Ueq as Compared to Neighbors of        C31 Check
PLAT241_ALERT_2_C High   'MainMol' Ueq as Compared to Neighbors of        C32 Check
PLAT242_ALERT_2_C Low    'MainMol' Ueq as Compared to Neighbors of       C00J Check
PLAT242_ALERT_2_C Low    'MainMol' Ueq as Compared to Neighbors of       C00T Check
PLAT242_ALERT_2_C Low    'MainMol' Ueq as Compared to Neighbors of       C00N Check
PLAT242_ALERT_2_C Low    'MainMol' Ueq as Compared to Neighbors of       C00U Check
PLAT242_ALERT_2_C Low    'MainMol' Ueq as Compared to Neighbors of       C00L Check
PLAT242_ALERT_2_C Low    'MainMol' Ueq as Compared to Neighbors of       C016 Check
PLAT242_ALERT_2_C Low    'MainMol' Ueq as Compared to Neighbors of       C00O Check
PLAT242_ALERT_2_C Low    'MainMol' Ueq as Compared to Neighbors of        C28 Check
PLAT250_ALERT_2_C Large U3/U1 Ratio for Average U(i,j) Tensor ....        2.4 Note
PLAT303_ALERT_2_C Full Occupancy Atom H15B      with # Connections       1.14 Check
PLAT303_ALERT_2_C Full Occupancy Atom H17B      with # Connections       1.14 Check
PLAT342_ALERT_3_C Low Bond Precision on  C-C Bonds ...............    0.01441 Ang.
PLAT906_ALERT_3_C Large K Value in the Analysis of Variance ......     22.265 Check
PLAT906_ALERT_3_C Large K Value in the Analysis of Variance ......      3.093 Check
PLAT910_ALERT_3_C Missing # of FCF Reflection(s) Below Theta(Min).          5 Note
                 1  0  0,   0  1  0,   0 -1  1,  -1  0  1,   0  0  1,
PLAT911_ALERT_3_C Missing FCF Refl Between Thmin & STh/L=    0.595         25 Report
```

```
              -9 -9  5,  -9 -8  5,  -9 -7  5, -10-10  6, -11 -9  6, -10 -9  6,
              -9 -9  6, -11 -8  6, -10 -8  6,  -9 -8  6, -11 -7  6, -10 -7  6,
              -9 -7  6, -11 -6  6, -11 -9  7, -11 -8  7, -12 -7  7, -12 -6  7,
             -11 -6  7, -13 -5  7,  11  3  7, -13 -5  8,  -8 -2 18,  -7 -2 18,
              -7 -2 19,
PLAT934_ALERT_3_C Number of (Iobs-Icalc)/Sigma(W) > 10 Outliers ..          1 Check
             -11 -7  7,
PLAT976_ALERT_2_C Check Calcd Resid. Dens.  0.76Ang From N15     .      -0.56 eA-3
PLAT976_ALERT_2_C Check Calcd Resid. Dens.  1.05Ang From N18     .      -0.53 eA-3
```

Alert level G

```
PLAT002_ALERT_2_G Number of Distance or Angle Restraints on AtSite          6 Note
PLAT003_ALERT_2_G Number of Uiso or Uij Restrained non-H Atoms ...          9 Report
PLAT004_ALERT_5_G Polymeric Structure Found with Maximum Dimension          1 Info
PLAT007_ALERT_5_G Number of Unrefined Donor-H Atoms .............          11 Report
              H15A  H15B  H15C  H17A  H17B  H16A  H16B  H16C  H18A  H18B  H18C
PLAT012_ALERT_1_G No       _shelx_res_checksum Found in CIF ......     Please Check
PLAT083_ALERT_2_G SHELXL Second Parameter in WGHT  Unusually Large      17.50 Why ?
PLAT093_ALERT_1_G No s.u.'s on H-positions, Refinement Reported as      mixed Check
PLAT176_ALERT_4_G The CIF-Embedded .res File Contains SADI Records          2 Report
PLAT177_ALERT_4_G The CIF-Embedded .res File Contains DELU Records          4 Report
PLAT186_ALERT_4_G The CIF-Embedded .res File Contains ISOR Records          1 Report
PLAT192_ALERT_3_G A Non-default DELU Restraint Value for SecondPar     0.0200 Report
PLAT192_ALERT_3_G A Non-default DELU Restraint Value for SecondPar     0.0200 Report
PLAT192_ALERT_3_G A Non-default DELU Restraint Value for SecondPar     0.0200 Report
PLAT192_ALERT_3_G A Non-default DELU Restraint Value for SecondPar     0.0200 Report
PLAT230_ALERT_2_G Hirshfeld Test Diff for    I005     --Ge09     .        6.0 s.u.
PLAT230_ALERT_2_G Hirshfeld Test Diff for    I006     --Ge0A     .        5.7 s.u.
PLAT230_ALERT_2_G Hirshfeld Test Diff for    I1       --Ge09     .       12.4 s.u.
PLAT230_ALERT_2_G Hirshfeld Test Diff for    I1       --Ge0A     .       12.0 s.u.
PLAT230_ALERT_2_G Hirshfeld Test Diff for    I3       --Ge0A     .       12.0 s.u.
PLAT230_ALERT_2_G Hirshfeld Test Diff for    I3       --Ge09_b   .       12.4 s.u.
PLAT230_ALERT_2_G Hirshfeld Test Diff for    F3       --C00V     .        6.6 s.u.
PLAT230_ALERT_2_G Hirshfeld Test Diff for    F5       --C29      .        7.6 s.u.
PLAT300_ALERT_4_G Atom Site Occupancy of F3         Constrained at        0.5 Check
PLAT300_ALERT_4_G Atom Site Occupancy of F7         Constrained at        0.5 Check
PLAT300_ALERT_4_G Atom Site Occupancy of H00V       Constrained at        0.5 Check
PLAT300_ALERT_4_G Atom Site Occupancy of F4         Constrained at        0.5 Check
PLAT300_ALERT_4_G Atom Site Occupancy of F5         Constrained at        0.5 Check
PLAT300_ALERT_4_G Atom Site Occupancy of H28        Constrained at        0.5 Check
PLAT301_ALERT_3_G Main Residue  Disorder .............(Resd  1  )        14% Note
PLAT302_ALERT_4_G Anion/Solvent/Minor-Residue Disorder (Resd  2  )        10% Note
PLAT302_ALERT_4_G Anion/Solvent/Minor-Residue Disorder (Resd  3  )        10% Note
PLAT432_ALERT_2_G Short Inter X...Y Contact  I001     ..Ge0A     .       3.35 Ang.
                                                      x,y,z  =      1_555 Check
PLAT432_ALERT_2_G Short Inter X...Y Contact  I003     ..Ge09     .       3.34 Ang.
                                                      x,y,z  =      1_555 Check
PLAT720_ALERT_4_G Number of Unusual/Non-Standard Labels ..........         48 Note
              I001    I002    I003    I004    I005    I006    I007    I008
              Ge09    Ge0A    C00J    C00T    C017    H017    C013    H013
              C00Y    H00Y    C00Z    H00Z    C00K    H00E    H00F    C00L
              C016    C00V    H00V    C00M    H00G    H00H    C00N    C011
              H011    C00U    C00O    C00P    H00I    H00J    C00Q    H00A
              H00B    C00R    H00K    H00L    C00S    H00C    H00D    I0
PLAT779_ALERT_4_G Suspect or Irrelevant (Bond) Angle(s) in CIF ...      24.73 Deg.
              I005 -GE09 -I0      1_555   1_555   1_555 ......     #    3 Check
```

```
PLAT779_ALERT_4_G Suspect or Irrelevant (Bond) Angle(s) in CIF ...      24.45 Deg.
              I006 -GE0A -I2      1_555   1_555   1_555 ......     #    9 Check
PLAT779_ALERT_4_G Suspect or Irrelevant (Bond) Angle(s) in CIF ...       5.40 Deg.
              F3   -C00V -H00V    1_555   1_555   1_555 ......     #   90 Check
PLAT779_ALERT_4_G Suspect or Irrelevant (Bond) Angle(s) in CIF ...       6.90 Deg.
              F4   -C28  -H28     1_555   1_555   1_555 ......     #  122 Check
PLAT789_ALERT_4_G Atoms with Negative _atom_site_disorder_group  #          2 Check
PLAT822_ALERT_4_G CIF-embedded .res Contains Negative PART Numbers          1 Check
PLAT860_ALERT_3_G Number of Least-Squares Restraints ............          12 Note
PLAT909_ALERT_3_G Percentage of I>2sig(I) Data at Theta(Max) Still        58% Note
PLAT961_ALERT_5_G Dataset Contains no Negative Intensities .......     Please Check
PLAT967_ALERT_5_G Note: Two-Theta Cutoff Value in Embedded .res ..       50.0 Degree
PLAT978_ALERT_2_G Number C-C Bonds with Positive Residual Density.          0 Info
```

---

0 **ALERT level A** = Most likely a serious problem - resolve or explain
4 **ALERT level B** = A potentially serious problem, consider carefully
38 **ALERT level C** = Check. Ensure it is not caused by an omission or oversight
45 **ALERT level G** = General information/check it is not something unexpected

4 ALERT type 1 CIF construction/syntax error, inconsistent or missing data
39 ALERT type 2 Indicator that the structure model may be wrong or deficient
13 ALERT type 3 Indicator that the structure quality may be low
27 ALERT type 4 Improvement, methodology, query or suggestion
4 ALERT type 5 Informative message, check

It is advisable to attempt to resolve as many as possible of the alerts in all categories. Often the minor alerts point to easily fixed oversights, errors and omissions in your CIF or refinement strategy, so attention to these fine details can be worthwhile. In order to resolve some of the more serious problems it may be necessary to carry out additional measurements or structure refinements. However, the purpose of your study may justify the reported deviations and the more serious of these should normally be commented upon in the discussion or experimental section of a paper or in the "special_details" fields of the CIF. checkCIF was carefully designed to identify outliers and unusual parameters, but every test has its limitations and alerts that are not important in a particular case may appear. Conversely, the absence of alerts does not guarantee there are no aspects of the results needing attention. It is up to the individual to critically assess their own results and, if necessary, seek expert advice.

Publication of your CIF in IUCr journals

A basic structural check has been run on your CIF. These basic checks will be run on all CIFs submitted for publication in IUCr journals (*Acta Crystallographica*, *Journal of Applied Crystallography*, *Journal of Synchrotron Radiation*); however, if you intend to submit to *Acta Crystallographica Section C* or *E* or *IUCrData*, you should make sure that full publication checks are run on the final version of your CIF prior to submission.

Publication of your CIF in other journals

Please refer to the *Notes for Authors* of the relevant journal for any special instructions relating to CIF submission.

PLATON version of 14/11/2023; check.def file version of 14/09/2023

Datablock 2FPEA2GeI4 - ellipsoid plot

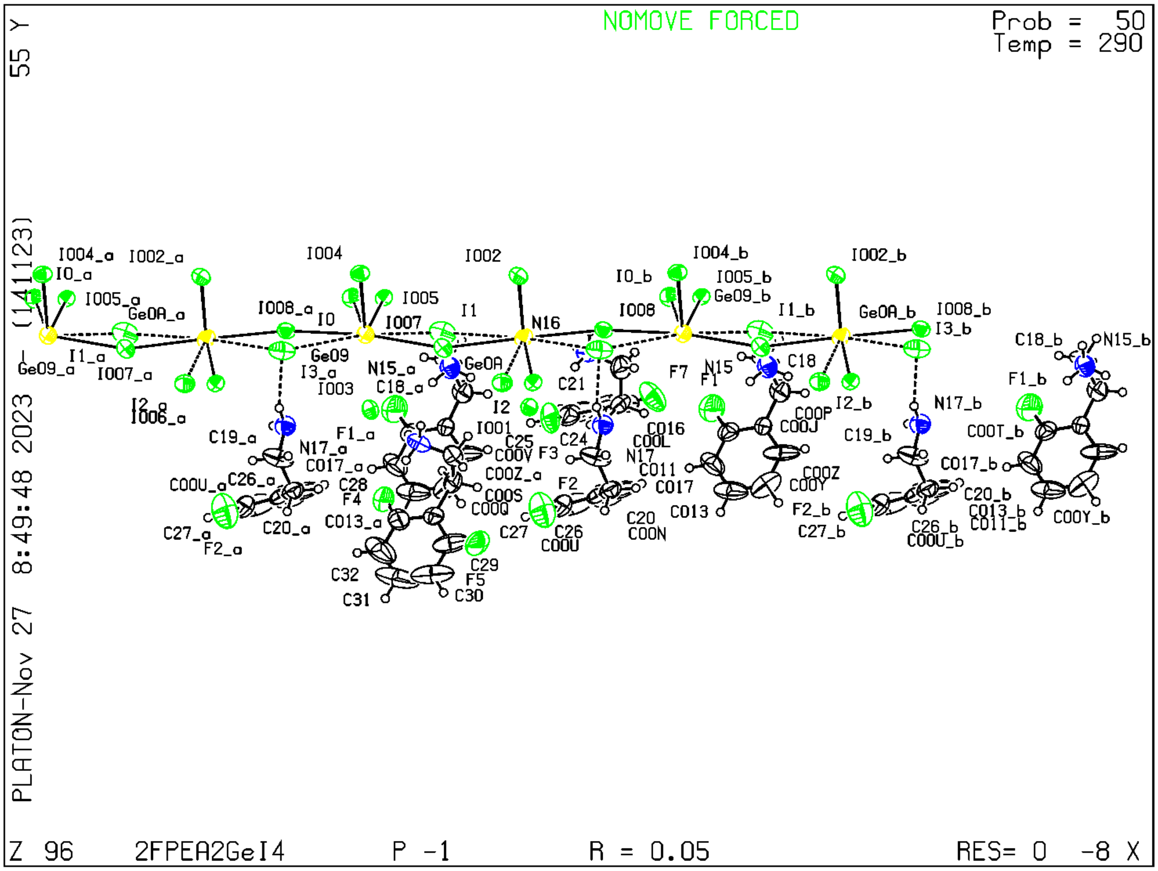

Supplement: Supplementary file 2 [file sciadv.aee5631_data_s1.zip › Ge-2FPEA-checkcif.pdf]
