# Supplementary material for: Unlock lasing in two-dimensional metal halide perovskites by tuning ns2 lone pairs
Source: Sci Adv. 2026 Aug 5;12(32):eaee5631. doi: 10.1126/sciadv.aee5631 (PMC13440396; doi:10.1126/sciadv.aee5631)

checkCIF/PLATON report

Structure factors have been supplied for datablock(s) exp_13468

THIS REPORT IS FOR GUIDANCE ONLY. IF USED AS PART OF A REVIEW PROCEDURE FOR PUBLICATION, IT SHOULD NOT REPLACE THE EXPERTISE OF AN EXPERIENCED CRYSTALLOGRAPHIC REFEREE.

No syntax errors found. CIF dictionary Interpreting this report

Datablock: exp_13468

Bond precision:	C-C = 0.0056 A		Wavelength=0.71073
Cell:	a=21.0210(4)	b=12.4859(2)	c=12.8898(3)
	alpha=90	beta=99.154(2)	gamma=90
Temperature:	290 K		

	Calculated	Reported
Volume	3340.05(12)	3340.05(11)
Space group	C 2/c	C 1 2/c 1
Hall group	-C 2yc	-C 2yc
Moiety formula	C7 H18 N2, Ge I4	Ge I3, I, C7 H18 N2
Sum formula	C7 H18 Ge I4 N2	C7 H18 Ge I4 N2
Mr	710.44	710.42
Dx,g cm-3	2.826	2.826
Z	8	8
Mu (mm-1)	9.205	9.205
F000	2544.0	2544.0
F000’	2531.09	
h,k,lmax		24,14,15
Nref		2940
Tmin,Tmax	0.167,0.479	0.782,1.000
Tmin’	0.053	

Correction method= # Reported T Limits: Tmin=0.782 Tmax=1.000
AbsCorr = GAUSSIAN

Data completeness= Theta(max)= 25.000

R(reflections)= 0.0191(2758) wR2(reflections)= 0.0463(2940)

S = 1.062 Npar= 129

The following ALERTS were generated. Each ALERT has the format
test-name_ALERT_alert-type_alert-level.
Click on the hyperlinks for more details of the test.

Alert level C

```
PLAT042_ALERT_1_C Calc. and Reported MoietyFormula Strings  Differ     Please Check
              Calc: C7 H18 N2, Ge I4
              Rep.: Ge I3, I, C7 H18 N2
PLAT420_ALERT_2_C D-H Bond Without Acceptor  N007     --H00B     .     Please Check
PLAT911_ALERT_3_C Missing FCF Refl Between Thmin & STh/L=    0.595          7 Report
              -21  3  1,  22  6  1, -12 12  4,  16  6  7,  10  0 10,  12  0 10,
                2  0 14,
```

Alert level G

```
PLAT007_ALERT_5_G Number of Unrefined Donor-H Atoms .............          4 Report
              H006  H00A  H00B  H00C
PLAT012_ALERT_1_G No       _shelx_res_checksum Found in CIF ......     Please Check
PLAT083_ALERT_2_G SHELXL Second Parameter in WGHT  Unusually Large       6.13 Why ?
PLAT093_ALERT_1_G No s.u.'s on H-positions, Refinement Reported as      mixed Check
PLAT720_ALERT_4_G Number of Unusual/Non-Standard Labels ..........         32 Note
              I001    I002    I003    I004    Ge05    N006    H006    N007
              H00A    H00B    H00C    C008    H00D    H00E    C009    H00F
              H00G    C00A    H00H    H00I    C00B    H00J    H00K    C00C
              H00L    C00D    H00M    H00N    H00O    C00E    H00P    H00Q
PLAT909_ALERT_3_G Percentage of I>2sig(I) Data at Theta(Max) Still        86% Note
PLAT910_ALERT_3_G Missing # of FCF Reflection(s) Below Theta(Min).          3 Note
                1  1  0,   2  0  0,  -1  1  1,
PLAT933_ALERT_2_G Number of HKL-OMIT Records in Embedded .res File          1 Note
               -1  1  1,
PLAT961_ALERT_5_G Dataset Contains no Negative Intensities .......     Please Check
PLAT967_ALERT_5_G Note: Two-Theta Cutoff Value in Embedded .res ..       50.0 Degree
PLAT978_ALERT_2_G Number C-C Bonds with Positive Residual Density.          0 Info
```

0 **ALERT level A** = Most likely a serious problem - resolve or explain
0 **ALERT level B** = A potentially serious problem, consider carefully
3 **ALERT level C** = Check. Ensure it is not caused by an omission or oversight
11 **ALERT level G** = General information/check it is not something unexpected

3 ALERT type 1 CIF construction/syntax error, inconsistent or missing data
4 ALERT type 2 Indicator that the structure model may be wrong or deficient
3 ALERT type 3 Indicator that the structure quality may be low
1 ALERT type 4 Improvement, methodology, query or suggestion
3 ALERT type 5 Informative message, check

It is advisable to attempt to resolve as many as possible of the alerts in all categories. Often the minor alerts point to easily fixed oversights, errors and omissions in your CIF or refinement strategy, so attention to these fine details can be worthwhile. In order to resolve some of the more serious problems it may be necessary to carry out additional measurements or structure refinements. However, the purpose of your study may justify the reported deviations and the more serious of these should normally be commented upon in the discussion or experimental section of a paper or in the "special_details" fields of the CIF. checkCIF was carefully designed to identify outliers and unusual parameters, but every test has its limitations and alerts that are not important in a particular case may appear. Conversely, the absence of alerts does not guarantee there are no aspects of the results needing attention. It is up to the individual to critically assess their own results and, if necessary, seek expert advice.

Publication of your CIF in IUCr journals

A basic structural check has been run on your CIF. These basic checks will be run on all CIFs submitted for publication in IUCr journals (*Acta Crystallographica*, *Journal of Applied Crystallography*, *Journal of Synchrotron Radiation*); however, if you intend to submit to *Acta Crystallographica Section C* or *E* or *IUCrData*, you should make sure that full publication checks are run on the final version of your CIF prior to submission.

Publication of your CIF in other journals

Please refer to the *Notes for Authors* of the relevant journal for any special instructions relating to CIF submission.

PLATON version of 14/11/2023; check.def file version of 14/09/2023

Datablock exp_13468 - ellipsoid plot

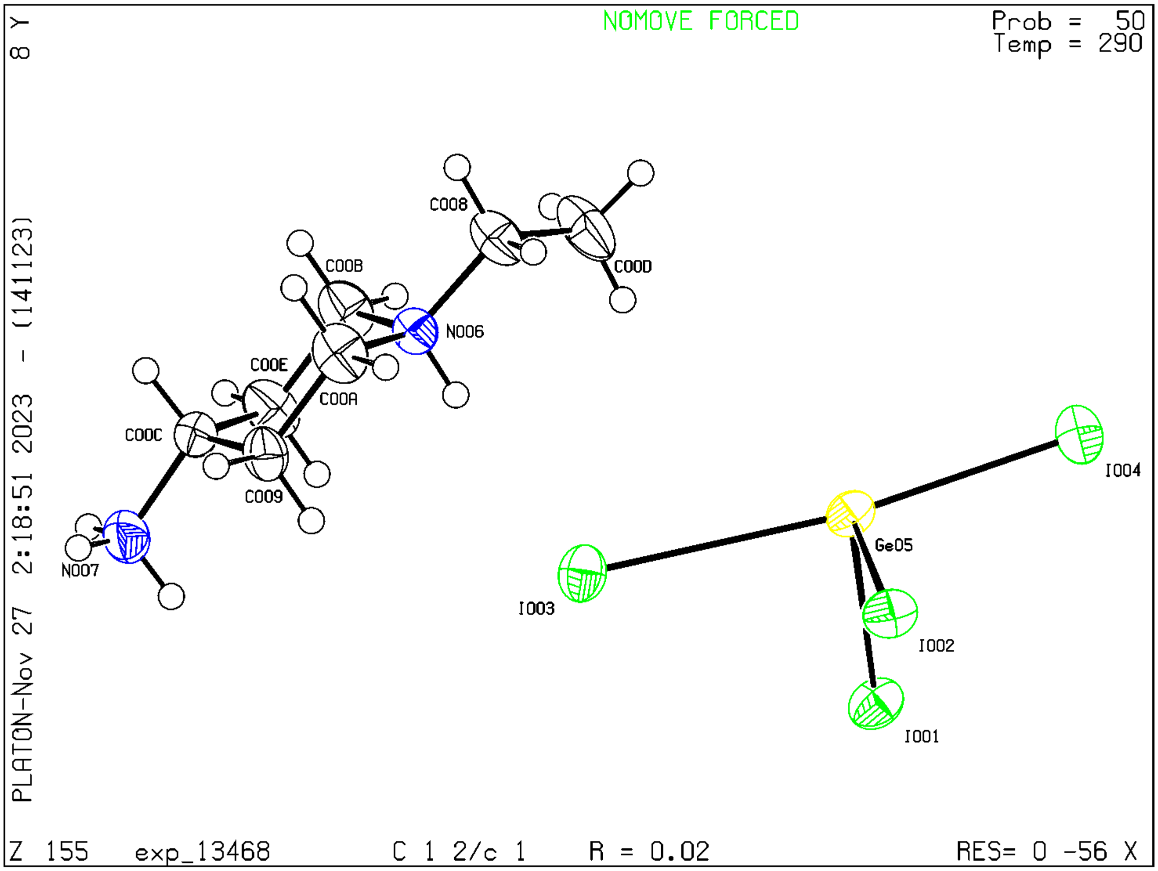

Supplement: Supplementary file 2 [file sciadv.aee5631_data_s1.zip › Ge-4aEtPD-checkcif.pdf]
